# Supplementary material for: Clinical and cost-effectiveness, safety and acceptability of community intravenous antibiotic service models: CIVAS systematic review
Source: BMJ Open. 2017 Apr 20;7(4):e013560. doi: 10.1136/bmjopen-2016-013560 (PMC5775457; doi:10.1136/bmjopen-2016-013560)
Supplement: supplementary appendix [file bmjopen-2016-013560supp001.pdf]

CIVAS SYSTEMATIC REVIEW: DATABASES SEARCHED AND SAMPLE SEARCHES

DATABASE	DATES
British Society for Antimicrobial Chemotherapy	Accessed March 2015 http://bsac.org.uk/
CINAHL (EBSCOHost)	1981 to March 2015
The Cochrane Library (Wiley)	Accessed March 25, 2015
Embase Classic + Embase (Ovid)	1947 to March 25, 2015
Health Business Elite (HDAS, NHS Evidence)	1922 to March 25, 2015
Health Management Information Consortium – HMIC (Ovid)	1983 to March 25, 2015
International Pharmaceutical Abstracts (Ovid)	1970 to March 2015
Ovid MEDLINE(R)	1946 to March Week 4, 2015
Ovid MEDLINE(R) In-Process & Other Non-Indexed Citations	March 25, 2015
Research Papers in Economics – RePEc	Accessed March 2015 http://ideas.repec.org/
CEA Registry	Accessed March 2015 https://research.tufts-nemc.org/cear4/Default.aspx
Web of Science Conference Proceedings Citation Index – Science (Thomson Reuters)	1990 to March 2015

Sample Search A: Studies of IV antibiotics and known models of care

Ovid MEDLINE(R) <1946 to March Week 4, 2015>

----

1 exp Anti-Bacterial Agents/ (497300)

2 Anti-Infective Agents/ (36221)

3 exp Anti-infective agents, Urinary/ (28162)

4 exp antifungal agents/ (126501)

5 or/1-4 (612854)

6 Administration, Intravenous/ (151)

7 infusions, intravenous/ (45941)

8 injections, intravenous/ (75646)

9 Home infusion therapy/ (579)

10 or/6-9 (120138)

11 ((parenteral or intravenous or IV or inject* or infusion*) adj5 (antibiotic* or anti-biotic* or antimicrobial* or anti-microbial* or antifungal* or anti-fungal* or anti-infective* or antiinfective* or antibiotherap*or anti-biotherap*)).tw. (10477)

12 (5 and 10) or 11 (20711)

13 exp Ambulatory Care Facilities/ (41613)

14 exp Delivery of healthcare/ (739029)

15 Critical Pathways/ (4202)

16 Ambulatory Care/ (34170)

17 Emergency Service, Hospital/ (39312)

18 outpatients/ (7955)

19 inpatients/ (11488)

20 day care/ (4526)

21 inpatients/ (11488)

22 hospitalization/ (66091)

23 community health services/ (25779)

24 community health nursing/ (17982)

25 home care services/ (26544)

26 home care services, hospital-based/ (1552)

27 home nursing/ (7802)

28 home infusion therapy/ (579)

29 or/13-28 (936219)

30 12 and 29 (1550)

31 ("emergency ward*" or "emergency room*" or "accident and emergency" or ED or "emergency department*" or A&E).tw. (77291)

32 outpatient*.tw. (95608)

33 (home or homes).tw. (141950)

34 (self adj3 (treat* or care or regime*)).tw. (14552)

35 ((clinic or clinics) adj3 (treat* or care or regime*)).tw. (11443)

36 (community adj3 (treat* or care or regime*)).tw. (15994)

37 (ambulatory adj3 (treat* or care or regime*)).tw. (11257)

38 "district nurs*".tw. (1351)

39 ("specialist nurs*" or "nurse specialist*").tw. (3498)

40 "community nurs*".tw. (2437)

41 ((hospital* or ward or clinic) adj2 (patient or patients)).tw. (58637)

42 inpatient*.tw. (54156)

43 ("care pathway*" or "care model*" or "model* of care").tw. (6275)

44 or/31-43 (438938)

45 44 and 12 (2102)

46 "outpatient parenteral antibiotic* therapy".tw. (103)

47 "outpatient antibiotic* therapy".tw. (64)

48 (ohpat or opat).tw. (116)

49 or/45-48 (2140)

50 49 or 30 (2678)

Sample Search B: Reviews of IV antibiotic use in cellulitis or cystic fibrosis

Ovid MEDLINE(R) <1946 to March Week 4, 2015>

----

1 exp *Anti-Bacterial Agents/ or *Anti-Infective Agents/ or exp *antifungal agents/ or exp *Anti-infective agents, Urinary/ (380181)

2 *Administration, Intravenous/ or *infusions, intravenous/ or *injections, intravenous/ or *Home infusion therapy/ (5005)

3 1 and 2 (324)

4 ((parenteral or intravenous or IV or inject* or infusion*) adj5 (antibiotic* or anti-biotic* or antimicrobial* or anti-microbial* or antifungal* or anti-fungal* or anti-infective* or antiinfective* or antibiotherap*or anti-biotherap*)).ti. (1464)

5 3 or 4 (1649)

6 exp Anti-Bacterial Agents/ (498780)

7 Anti-Infective Agents/ (36330)

8 exp Anti-infective agents, Urinary/ (28211)

9 exp antifungal agents/ (126949)

10 or/6-9 (614703)

11 Administration, Intravenous/ (177)

12 infusions, intravenous/ (46068)

13 injections, intravenous/ (75786)

14 Home infusion therapy/ (580)

15 or/11-14 (120429)

16 ((parenteral or intravenous or IV or inject* or infusion*) adj5 (antibiotic* or anti-biotic* or antimicrobial* or anti-microbial* or antifungal* or anti-fungal* or anti-infective* or antiinfective* or antibiotherap*or anti-biotherap*)).tw. (10513)

17 (10 and 15) or 16 (20758)

18 "outpatient* parenteral".tw. [finds more] (189)

19 "outpatient parenteral antibiotic* therapy".tw. (103)

20 "outpatient antibiotic* therapy".tw. (64)

21 (ohpat or opat).tw. (116)

22 Cellulitis/ (6046)

23 cellulitis.tw. (5439)

24 Cystic Fibrosis/ (27005)

25 (cystic adj fibrosis).tw. (29491)

26 "fibrocystic disease*".tw. (807)

27 17 or 19 or 20 or 21 (20796)

28 or/22-26 (43980)

29 (5 or (18 or 19 or 20 or 21)) and 28 [major headings or title occurence of IV Antibiotic terms AND MINOR Cell/CF terms] (125)

30 17 and 28 [most sensitive search] (1037)

31 limit 30 to "review articles" (150)

32 29 or 31 (254)
