# Supplementary material for: Clinical and cost-effectiveness, safety and acceptability of community intravenous antibiotic service models: CIVAS systematic review
Source: BMJ Open. 2017 Apr 20;7(4):e013560. doi: 10.1136/bmjopen-2016-013560 (PMC5775457; doi:10.1136/bmjopen-2016-013560)
Supplement: supplementary appendix [file bmjopen-2016-013560supp002.pdf]

# CIVAS SYSTEMATIC REVIEW:
# QUALITY ASSESSMENT – OBSERVATIONAL STUDIES

| **Population** (tick one box) | | **PAPER ID:** |
|---|---|---|
| 1. Sample size: | | |
| a) Strong – power calculation provided | ✪ | ☐ |
| b) Strong – included all OPAT patients | ✪ | ☐ |
| c) Strong – appropriate size for qualitative study | ✪ | ☐ |
| d) Insufficient – no power calculation or selective/small study | | ☐ |
| e) Unclear – not enough information provided | | ☐ |
| **Methodology:** (tick one box) | | |
| 2. Method to ascertain data: | | |
| a) Strong – not open to selection bias* | ✪ | ☐ |
| b) Strong – appropriate qualitative methodology | ✪ | ☐ |
| c) Moderate – open to some bias but appropriate method used | ○ | ☐ |
| d) Insufficient – inappropriate method used to ascertain data | | ☐ |
| e) Unclear – not enough information provided | | ☐ |
| **Analysis:** (tick one box) | | |
| 3. Data analysis: | | |
| a) Strong – reports statistically significant differences in outcomes | ✪ | ☐ |
| b) Strong – relevant qualitative analytic techniques | ✪ | ☐ |
| c) Moderate – used only comparative analysis with no statistical testing of differences | ○ | ☐ |
| d) Moderate – reported only descriptive statistics | ○ | ☐ |

* Systematic differences between comparison groups in terms of selection, prognosis or treatment; differences in group characteristics.

## ASSESSMENT OF THE RISK OF BIAS:

| | | | | |
|---|---|---|---|---|
| **Potential bias:** | Selection | ☐ | Analysis | ☐ |
| | Method | ☐ | **ASSESSMENT** | ☐ |
