# Supplementary material for: Clinical and cost-effectiveness, safety and acceptability of community intravenous antibiotic service models: CIVAS systematic review
Source: BMJ Open. 2017 Apr 20;7(4):e013560. doi: 10.1136/bmjopen-2016-013560 (PMC5775457; doi:10.1136/bmjopen-2016-013560)
Supplement: Author's manuscript [file bmjopen-2016-013560.draft_revisions.pdf]

BMJ Open

Clinical and cost-effectiveness, safety and acceptability of community intravenous antibiotic service models: CIVAS systematic review

Journal:	*BMJ Open*
Manuscript ID	bmjopen-2016-013560
Article Type:	Research
Date Submitted by the Author:	20-Jul-2016
Complete List of Authors:	Mitchell, Elizabeth; University of Leeds, Leeds Institute of Health Sciences<br>Czoski Murray, Carolyn; University of Leeds, Leeds Institute of Health Sciences<br>Meads, David; University of Leeds, Leeds Institute of Health Sciences<br>Minton, Jane; Leeds Teaching Hospitals NHS Trust,<br>Wright, Judy; University of Leeds, Leeds Institute of Health Sciences<br>Twiddy, Maureen; University of Leeds, Leeds Institute of Health Sciences
**Primary Subject Heading**:	Health services research
Secondary Subject Heading:	Infectious diseases, Evidence based practice
Keywords:	Organisation of health services < HEALTH SERVICES ADMINISTRATION & MANAGEMENT, Quality in health care < HEALTH SERVICES ADMINISTRATION & MANAGEMENT, Infection control < INFECTIOUS DISEASES

SCHOLARONE™
Manuscripts

For peer review only

For peer review only - http://bmjopen.bmj.com/site/about/guidelines.xhtml

Clinical and cost-effectiveness, safety and acceptability of community intravenous antibiotic service models: CIVAS systematic review

ED Mitchell, C Czoski Murray, D Meads, J Minton, J Wright, M Twiddy

Centre for Health Services Research, Leeds Institute of Health Sciences, University of Leeds, Charles Thackrah Building, 101 Clarendon Road, Leeds UK LS2 9LJ

ED Mitchell, *Senior Research Fellow*; C Czoski Murray, *Senior Research Fellow*; M Twiddy, *Senior Research Fellow*

Academic Unit of Health Economics, Leeds Institute of Health Sciences, University of Leeds, Charles Thackrah Building, 101 Clarendon Road, Leeds UK LS2 9LJ

D Meads, *Associate Professor*; J Wright, *Senior Information Specialist*

Leeds Teaching Hospitals NHS Trust, Infection and Travel Medicine, St James's Hospital, Beckett Street, Leeds UK LS9 7TF Road, Leeds UK LS2 9LJ

J Minton, *Consultant in Infectious Diseases*

For peer review only

Correspondence to:

Dr Liz Mitchell, Leeds Institute of Health Sciences, University of Leeds, Charles Thackrah Building, 101 Clarendon Road, LEEDS UK LS2 9LJ; *Email*: e.d.mitchell@leeds.ac.uk

WORD COUNT: 5,102

For peer review only - http://bmjopen.bmj.com/site/about/guidelines.xhtml

ARTICLE SUMMARY

Strengths and limitations of this study

- This review provides the most comprehensive picture of OPAT-related research in recent years, bringing together available evidence on the clinical and cost-effectiveness, safety and acceptability of community-based antibiotic delivery
- Much of the work in this area is based around service evaluation and many studies provided only basic descriptive findings, making it difficult to grade the quality and robustness of the evidence
- Although many studies were identified and included in the review, the definitiveness of its conclusions related to the clinical benefit of OPAT over traditional inpatient treatment is limited by the lack of good quality, comparative studies
- Nonetheless, the synthesis provided by this review provides much needed evidence to inform the design and commissioning of OPAT services

For peer review only

ABSTRACT

Objective: Evaluate evidence of the efficacy, safety, acceptability and cost-effectiveness of outpatient parenteral antimicrobial therapy (OPAT) models.

Design: Systematic review.

Data sources: MEDLINE, EMBASE, CINAHL, Cochrane Library, NHS EED, RePEc, Tufts CEA Registry, Health Business Elite, HMIC, Web of Science Proceedings, International Pharmaceutical Abstracts, British Society for Antimicrobial Chemotherapy website. Searches were undertaken from 1993-2015.

Study selection: All studies, except case reports, considering adult patients or practitioners involved in the delivery of OPAT were included. Studies combining outcomes for adults and children or non-IV and IV antibiotic groups were excluded, as were those focused on process of delivery or clinical effectiveness of one antibiotic over another. Titles/abstracts were screened by one reviewer (20% verified). Two authors independently screened studies for inclusion.

Results: 128 studies involving >60,000 OPAT episodes were included. 22 studies (17%) did not indicate the OPAT model used; only 29 involved a comparator (23%). There was little difference in duration of OPAT treatment compared to inpatient therapy, and overall OPAT appeared to produce superior cure/improvement rates. However, when models were considered individually, outpatient delivery appeared to be less effective, and self-administration and specialist nurse delivery more effective. Drug side-effects, deaths and hospital re-admissions were similar to those for inpatient treatment, but there were more line-related complications. Patient satisfaction was high, with advantages seen in being able to resume daily activities and having greater freedom and control. However, most professionals perceived challenges in providing OPAT.

Conclusions: There were no systematic differences related to the impact of OPAT on treatment duration or adverse events. However, evidence of its clinical benefit compared with traditional inpatient treatment is lacking, primarily due to the dearth of good quality, comparative studies. There was high patient satisfaction with OPAT use but the few studies considering practitioner acceptability highlighted organisational and logistic barriers to its delivery.

For peer review only - http://bmjopen.bmj.com/site/about/guidelines.xhtml

INTRODUCTION

Delivery of intravenous (IV) antibiotics outside of the hospital setting (often termed outpatient parenteral antimicrobial therapy (OPAT)) is widely accepted as standard practice in many countries, including the USA and Australia.[1] In the USA alone, an estimated quarter of a million patients receive IV antibiotics on an outpatient basis each year,[2] and this method of delivery has been used to treat a wide variety of infections, among them skin and soft tissue infections, bone and joint infections, pneumonia, and endocarditis.

In recent years OPAT services have also been developed in the UK, both in the NHS and private sectors.[3] It has the potential to deliver cost efficiency savings and improved patient experience[4] through fewer in-patient bed days, antimicrobial agents that need less frequent administration and choice of treatment setting. Despite this, implementation of OPAT is patchy and there is significant geographical variation in both the setting and the health care professions involved. Four primary models of OPAT delivery are in use: outpatient attendance at a healthcare facility, patient (or carer) self-administration, administration by a visiting general nurse, and administration by a visiting specialist nurse.

The failure to realise the full potential of OPAT in the UK is due in no small part to the paucity of information related to optimal delivery, most notably evidence of effectiveness, associated risks, and patient preferences for this form of treatment. The purpose of this review was to evaluate existing evidence in relation to the efficacy, safety, acceptability and cost-effectiveness of different models of OPAT delivery.

METHODS

Identification of studies

A comprehensive search strategy was developed by an experienced information specialist (JW) and a range of bibliographic sources were searched for the period 1993 to March 2015: Medline, Medline in process, EMBASE, CINAHL, International Pharmaceutical Abstracts, the Cochrane Library, the NHS Economic Evaluation Database (EED), Research Papers in Economics (RePEc), Tufts Cost-Effectiveness Analysis (CEA) Registry, and Health Business Elite (HBE). This timeframe was selected to take account of the substantial changes in the organisation of the NHS and the shift towards more community involvement that have taken place following publication of National Health Service and Community Care Act 1990.[5] Two separate searches were run. The first identified studies of IV antibiotics and known models of delivery, while the second identified reviews of antibiotic use in cellulitis or cystic fibrosis (infections where OPAT is frequently used) to allow for the identification of delivery models that

For peer review only - http://bmjopen.bmj.com/site/about/guidelines.xhtml

were unknown to us and subsequently not considered when identifying terms for the first search.

Supplementary searches of Web of Science Proceedings, the Health Information Management Consortium (HMIC), and the website of the British Society for Antimicrobial Chemotherapy were conducted to identify relevant unpublished work. In addition, the reference lists of included studies were reviewed for potentially relevant papers (see Appendix 1 for sample search strategies and list of databases searched).

Criteria for inclusion

Studies evaluating the clinical effectiveness or cost-effectiveness of an OPAT model, describing or evaluating patient safety issues associated with OPAT, or considering the acceptability of OPAT were included. The population included adult patients treated for any condition (and/or their carer in the case of acceptability studies) or practitioners involved in the delivery of OPAT. Any form of intravenous antibiotic drug delivery system (e.g. infusion or bolus) was included. Studies of any research design were considered (with the exception of single case reports), and no language restrictions were applied.

Studies were excluded if they considered the costs related to a model of delivery but did not consider patient benefit alongside these, or if they made reference to costs and benefits but did not report specific cost-effectiveness data (such as cost per Quality Adjusted Life Year (QALY)). Similarly, studies that made reference to clinical effectiveness without reporting specific patient outcomes were also excluded. Studies that included children or that involved multiple routes of antibiotic delivery were reviewed but excluded if they did not differentiate between outcomes for adult patients or for patients receiving IV treatment, and those of other participants. Studies that focused only on the method or process of delivery or on the clinical effectiveness of a single treatment or of one class of antibiotic over another were excluded, as were abstract only, descriptive or commentary pieces and guidance documents.

For peer review only

Selection of studies

Titles and abstracts of all identified studies were screened for eligibility by one reviewer (EDM) with a random selection (20%) independently screened by a second. Full text versions of papers not excluded at this stage were obtained for detailed review and independently assessed by two reviewers (EDM with CCM, DM or MT) to determine whether they met the inclusion criteria. Differences of opinion were discussed until a consensus was reached, with validation sought from a third reviewer where necessary.

For peer review only - http://bmjopen.bmj.com/site/about/guidelines.xhtml

Data extraction

Data extraction was carried out by one experienced reviewer (EDM) using a standardised proforma. Data for a sample of studies were extracted independently by a second reviewer (CCM) in order to validate the items being collected. Information was collected on study purpose and design (including factors related to quality assessment), setting, duration, population and clinical characteristics, models of delivery (outpatient, self-administration, general nurse, specialist nurse), outcome area (clinical effectiveness, cost-effectiveness, safety, acceptability), antibiotic parameters (type, delivery route, treatment dose), outcome measures, follow-up and key findings.

Assessment of bias

Studies were assessed for bias by two reviewers (EDM and CCM), where possible using previously developed scoring systems. The Cochrane Risk of Bias assessment tool was used for experimental studies (randomised controlled trials, clinical trials, controlled before and after studies), and the Newcastle-Ottawa scales for cohort and case-control studies.[6-7]

A method of assessing the strength of evidence of other observational studies – developed for previous reviews on early diagnosis of cancer[8-10] – was modified for this topic area and applied to relevant studies. In this system, papers were evaluated on three key areas: Population, Methodology and Analysis (Appendix 2). Population assesses the method used to ensure that a study is appropriately powered/produces generalizable results, with use of a sample size calculation or inclusion of all possible patients or providers rated more highly than selective recruitment. Methodology assesses procedures for obtaining study data, with use of a rigorous approach designed to reduce systematic differences between groups (selection, characteristics, treatment etc.) rated more highly than other methods. Analysis assesses use of analytic techniques, with reporting of relevant significant comparisons or differences (or use of appropriate analytic techniques if qualitative) rated more highly than non-statistical comparisons or descriptive data.

Four categories were then used to the weight the evidence: *STRONG+* (study graded strong in all three areas); *STRONG–* (study graded strong in two areas and moderate in the third); *MODERATE* (study graded strong in one area and moderate in two, or moderate in all three areas); *INSUFFICIENT* (study used a selective study population and/or an inappropriate method to ascertain data, or did not provide enough information to be able to determine a grading).

For peer review only - http://bmjopen.bmj.com/site/about/guidelines.xhtml

Many of the papers included in this review used methodologies that did not lend themselves to the scoring systems outlined. Many studies included all patients in receipt of OPAT since its establishment at a particular institution or over a specified time period, and simply reported conditions treated and therapies used, along with limited outcomes data. Case-series such as these, which were to all intents and purposes service evaluations that included little or no analytic content, were not subject to formal quality assessment.

In keeping with accepted good practice, studies at risk of bias were not excluded from the review, but an appraisal of the strength of existing evidence has been reported, and findings interpreted in light of this.

Data synthesis

The main characteristics of included studies and findings relating to clinical effectiveness, cost-effectiveness, patient safety, acceptability, and study quality have been summarised in narrative and tabular form. Substantial heterogeneity in clinical characteristics – condition treated, treatment duration, definition of a successful outcome (cure, improvement, deterioration etc.) – and methodology precluded pooling of study data for meta-analysis.

Patient involvement

This review formed one work stream of a larger NIHR-funded study that included qualitative interviews and economic modelling. The research team included a lay co-applicant who commented on study design prior to submission of the funding proposal, including the focus of the systematic review and the outcomes to be included. In addition, a Patient Advisory Group (PAG) was established to input at key phases throughout the project including the design of patient recruitment materials, interview topic guides and health economics aspects of the study. An Expert Panel was convened to discuss emerging findings from each work stream, and a representative of the PAG was a member of the panel. A project dissemination event was held in April 2016 to which both patients and professionals were invited.

RESULTS

The search strategy identified 7,214 articles of which 589 met the inclusion criteria for detailed review (Figure 1). The full-text of an additional 17 papers, identified from the reference lists of previous reviews and identified references, was also obtained giving a total of 606 potentially relevant papers. In a change to the initial protocol, non-English language papers were identified but were not assessed for inclusion (n=69), and we were unable to obtain one article from the library document supply service. One hundred and twenty-eight papers involving more than 60,000 OPAT episodes were included in the final analysis (Tables 1-2).[11-138]

For peer review only - http://bmjopen.bmj.com/site/about/guidelines.xhtml

Population and setting

Three-quarters of studies were undertaken in Europe (n=53; 41%) and North America (n=45; 35%). Almost two-thirds of the European studies were UK based. Two studies involved centres in more than one country,[41, 101] and one was a systematic review of world literature.[60] Studies were comparatively small in size , involving between 6 and 11,427 participants or episodes of care (mean 476; median 100); almost two-thirds (63%) had fewer than 150 participants. It was not possible to determine total participant numbers in two studies.[32, 138] The period under study ranged from 6 weeks to 15 years. In general, those studies with the largest numbers of subjects (>1000) either analysed all cases included in an OPAT registry and/or reviewed cases over a more substantial time period.[18, 32, 37, 41, 45, 66, 76, 84, 112, 130, 133]

The most commonly reported reason for treatment was osteomyelitis (n=68, 53%), followed by endocarditis (n=53, 41%), skin and soft tissue infection (n=41, 32%), cellulitis (n=32, 25%), and septic arthritis (n=29, 23%). Most studies involved multiple conditions (n=72, 56%); seven did not specify indications for treatment. These included two qualitative studies and one survey of patient acceptability,[16, 26, 67] three surveys of practitioner acceptability,[63, 68, 107] and one secondary analysis of data from an OPAT database.[17]

Outcomes and OPAT models studied

Of the five outcome areas evaluated in the review, the most commonly considered were patient safety (n=109; 85%) and clinical effectiveness (n=89; 70%). Just over a quarter of studies involved some aspect of patient acceptability (n=37; 29%), but few determined cost-effectiveness (n=5) or practitioner acceptability (n=6). Most investigated multiple areas (70%).

Twenty two of the 128 studies (17%) either did not indicate the type of OPAT delivery model used, or reported that home treatment was used without providing any additional detail. In the remainder, the most frequently reported model across studies was self (or carer) administration (n=66; 52%), followed by visits from a specialist nurse (n=44; 34%), outpatient treatment (n=35; 27%), and visits from a general nurse (n=14; 11%). Just over half of these studies evaluated a single model of delivery (n=59; 55%). Other, less common delivery methods and locations included infusion centres, home infusion or home care companies, Hospital in the Home Units (HHU), prison, and doctor visits (Tables S1–S5).

Quality assessment

Few studies employed an experimental or quasi-experimental design (Tables S1–S5). Most (67%) were observational, primarily case-series, and many involved retrospective data

For peer review only - http://bmjopen.bmj.com/site/about/guidelines.xhtml

collection (n=57; 45%). Of the 12 included randomised controlled trials, five reported on subgroup analyses from the main study and all failed to provide details of the trial methodology.[15, 77, 99-100, 102] One of two included clinical trials did not specify the delivery model under evaluation.[39]

Many studies analysed data obtained from medical records or prospectively held OPAT databases (42%); a small number of others carried out secondary analysis of national or international OPAT Registries. Satisfaction surveys, either by questionnaire or telephone completion, were frequently used, while interviews (face-to-face, telephone, focus group) and visual analogue scales were also well represented. Less common methods of data collection included clinic or ward diaries, direct observation, and data from previous studies or the published literature. It was often unclear who collected data, and few studies reported on how this was carried out. A substantial number provided little or no detail on the methods used. Twenty one of the 128 (16%) studies received full or partial funding by pharmaceutical companies, 72 others (56%) did not report the funding source.

Risk of bias within studies

Three of the 14 included trials were assessed as having a low risk of bias,[30, 103, 131] and one a high risk of bias (pilot study where patients self-selected hospital or home treatment and were recruited consecutively to each arm after this decision was made).[40] In the remaining ten studies, the level of potential bias was unclear. In five cases, it was uncertain whether randomisation or controlling had taken place. Four of these studies reported on subgroup analyses from a single open label trial, and none provided details of the original study methodology.[15, 77, 99-100] The fifth reported on two related trials comparing IV with oral treatment for neutropenia in cancer patients, and again reported no methodological details of the parent studies.[39] In the remaining five trials, details of randomisation, allocation concealment and blinding (especially in relation to assessment of outcome measures) were poorly reported.[101-102, 111, 116, 132] In addition, one the trials closed early due to poor recruitment,[87] whilst population bias due to age and treatment duration differences could not be excluded from another.[132]

All five included case-control studies (three of them retrospective) rated low for potential bias (median 8/9; range 6-9).[44, 78, 98, 136-137] Those studies scoring lowest did not provide details on the methods used to determine outcomes.[44, 78] Similarly, four cohort studies (two retrospective) all had low potential for bias (median 8/9; range 7-9)[12, 20, 76, 112]; lower scores related to possible failure to control for potential differences.[20, 76]

For peer review only - http://bmjopen.bmj.com/site/about/guidelines.xhtml

In the majority of observational studies, the data analysed were derived from OPAT databases or medical records review. Many studies also involved questionnaire surveys. Only five included a comparator (inpatient care),[23, 61, 75, 81, 122] with many simply including all OPAT patients over a selected time period. Most studies reported descriptive results only, with no statistical testing of differences.

Impact on clinical outcomes

Only 21 of the 89 studies evaluating the clinical effectiveness of OPAT included a comparator, which with few exceptions, was inpatient treatment (Table 3; Table S1). Five of the studies did not specify the OPAT delivery model used,[39, 75, 122, 132, 136] while two others reported combined results for multiple models.[102, 137]

Regardless of the particular OPAT model used, most studies assessing duration of treatment found little difference compared to inpatient therapy[30, 32, 40, 44, 81, 98, 101, 103, 131] (Table 3). Two studies (one using specialist nurses[18] the other self-administration[74]) found that the average length of OPAT treatment was shorter. A third study evaluating a Hospital at Home Unit (HHU) found a significant reduction in treatment duration for patients with cellulitis (median 6 days vs. 8.6 days; 95% CI 0.24-4.85) but not for patients with pyelonephritis (4.6 days vs. 4 days; 95% CI -1.86-0.72).[81]

Evidence of the effect of OPAT on cure rate however, was less conclusive. When findings from all delivery models were considered, OPAT appeared to produce superior results to those seen for inpatient treatment. This may reflect heterogeneity in the population groups, with inpatients being thus because they required more intensive care. However, it is also undoubtedly influenced by the inclusion of positive studies that reported on multiple or unspecified OPAT treatment models.[75, 102, 137] When these were removed and specific models considered individually, outpatient delivery appeared to have a lower rate of cure or improvement than inpatient treatment (89% vs. 95%)[101] and self-administration[20] and OPAT by specialist nurse a higher rate.[44, 81]. One study found that duration of neutropenia-related fever in cancer patients was significantly reduced with specialist nurse delivered OPAT (2 days vs. 5 days; $p<0.001$),[44] and another that rates of recovery for pyelonephritis were higher (93% vs. 90%)[62], although this was not the case for cellulitis (93% vs. 100%).[81] OPAT delivered by general nurse however, had little or no impact on rates of recovery. One study of treatment for community-acquired pneumonia[103] found no significant difference in time to resolution of fever, tachycardia and tachypnoea, while another found no significant difference in days to no advancement of cellulitis[30] (mean 1.50 vs. 1.49; 95% CI -0.3 to 0.28; $p=0.90$). Results from studies assessing

For peer review only - http://bmjopen.bmj.com/site/about/guidelines.xhtml

the impact of OPAT specifically on lung function in patients with cystic fibrosis were either inconclusive or showed no differential impact.[23, 40, 98, 131]

In the remaining studies that considered clinical effectiveness for OPAT models only, rates of cure or improvement ranged from 61.1-100% (mean 89.6%; median 92.5%). When the various OPAT models were considered individually, the highest mean cure/improvement rate was found for self-administration (91.3%), followed by specialist nurse (90.6%), general nurse (90.0%) and outpatient treatment (88.3%). Few studies reported on bacterial eradication, but those that did saw rates of between 57.1-100% (mean 86.2%; median 90.0%) (Table S1).

Patient safety and adverse events

Only 24 of the 109 studies evaluating OPAT-related safety included a comparator, which in the majority of cases was inpatient treatment (Table 3; Table S2). Five studies did not specify the OPAT model being used[39, 61, 75, 132, 136] and three others reported combined results for multiple models.[76, 102, 137]

There was little evidence of impact on either drug-related side effects or number of deaths in OPAT patients compared to patients receiving treatment in hospital[20, 32, 40, 44, 76, 81, 98, 101, 103, 109, 131] (Table 3). One study evaluating OPAT via outpatient treatment[101] reported a higher death rate (1 patient vs. 0 patients), but this was a small study and the overall rate of side-effects was lower in the OPAT group (15% vs. 18%). There also appeared to be no conclusive evidence of benefit either in relation to hospital re-admissions overall or in relation to those who self-administered therapy.[20, 131] There were conflicting results for OPAT delivered by nurses, with specialist nurses[32, 78, 81, 109] appearing to have superior results to those of general nurses.[30, 103] Perhaps unsurprisingly, overall there appeared to be more line-related complications in IV therapy administered outside of hospital.

Across all studies, the most commonly reported adverse events were rash, fever, nausea/vomiting, diarrhoea, allergic reaction or anaphylaxis, phlebitis, leucopenia, and line complications (including line infection, occlusion, breakage, and dislodgement) (Table S2).

Cost-effectiveness of OPAT

While many of the identified studies reported on the cost of OPAT, only five considered cost-effectiveness (Table S3). Three studies applied decision tree models to OPAT provided by specialist nurses,[95, 119, 138] with one also determining the cost-effectiveness of self-administration.[138] The remaining studies (one literature review, one retrospective observational study) did not specify the OPAT model(s) used.[60, 122]

For peer review only - http://bmjopen.bmj.com/site/about/guidelines.xhtml

In two of the three decision tree analyses, IV OPAT was found to be more cost-effective than IV inpatient therapy.[119, 138] In one case, it was also more cost-effective than early discharge with oral therapy and oral outpatient therapy.[119] In the other, its dominance was only maintained when the IV success rate was greater than 55%.[138] Conversely, in the third study IV OPAT was found to be less cost-effective than both IV to oral switch therapy, and oral treatment both during and after hospitalisation (which was the most cost-effective option).[95] However, the probability estimates used in two of the three scenarios in this study were derived from different data sources (published research and institutional data). In addition, there were no published data related to the baseline probability for cure in the third scenario, and so this was assumed to be at some point between the estimates used in the other two.

Studies included in the literature review predominantly concluded that home IV antibacterial therapy would lead to significant cost reductions, both from a societal and third-party payer perspective.[60] In five of the 11 studies, inpatient therapy was 2-3 times as expensive as home care therapy. However, there was considerable variation in the way in which costs were determined and calculated across studies (e.g. incremental costs, costs for selected components only etc.), while the review itself lacked considerable detail on the methodology used and the criteria for study inclusion and exclusion.

Findings from the observational study (based on 435 courses of IV antibiotic treatment for respiratory exacerbations in 116 adult patients with cystic fibrosis) indicated that for both one course and one year of treatment, IV antibiotic treatment administered mostly in hospital was more effective but more costly than treatment administered mostly at home.[122] This improved clinical effectiveness could only be achieved with the input of considerable additional resources (between £46 and £73k per patient at 2002 prices). However, when the strictest definition of effectiveness was applied (≤0% decline in lung function), hospital treatment was unlikely to ever be cost-effective.

Acceptability of OPAT

Patient preference

Only four of the 36 studies considering patients' acceptability of OPAT involved a comparison of inpatient and outpatient therapy (Table S4), two where OPAT delivery was by general nurses[30, 103] and two where delivery was by specialist nurses.[32, 44] In each case, satisfaction was high, with home treatment seen as beneficial. One of the two general nurse studies found that only 5% of home group patients would have preferred hospital treatment, compared with 35% of hospital patients who would have preferred treatment at home (p<0.001).[30] The other found

For peer review only - http://bmjopen.bmj.com/site/about/guidelines.xhtml

that patients in the home group were significantly happier with the location of their care than those receiving inpatient therapy ($p<0.001$).[103] Similarly, in one of the studies where patients received home care by specialist nurses, almost all (97%) indicated that they would chose to receive at-home therapy in future and would recommend it to others.[44] The main reasons given for this were quiet and increased home comfort, familiar environment, and free choice of activity. However, some patients in this study did report disadvantages to receiving therapy at home, primarily related to patient and caregiver anxiety.[44]

In the remaining studies that considered acceptability in OPAT patients only, most involved multiple OPAT models (and did not differentiate between them in their findings), or did not specify the model(s) under study. For the most part, satisfaction with treatment was very high,[11, 26, 28, 42, 45, 48, 51-52, 55-57, 59, 74, 89-90, 104-105, 108, 113, 120, 123-124] including where patients had to have frequent attendance at hospital.[22, 135] Commonly perceived advantages of OPAT included the ability to resume daily activities,[45, 56, 59, 74] feelings of improved self-esteem or greater freedom and control,[48, 56, 74, 82, 93] and not having to remain in or attend hospital.[11, 56, 59, 82, 118] The main disadvantages were most commonly related to infusion equipment, and included anxiety about the device and its sterility,[16, 67, 97] Two studies found that younger patients were better able to use infusion devices, and required less support to do so than older patients.[31, 67]

Two studies determined patients' willingness to pay to have treatment in their preferred location, and although differences did not reach statistical significance, patients reported that they would pay more for home than hospital-based treatment, including giving up slightly more of their remaining life to ensure this.[73, 120]

Provider preference

Only six studies included assessment of practitioner acceptability; one involving GPs, one involving nurses, and four involving infection specialists (Table S5). In most cases, professionals saw advantages (or a need) for patients receiving IV antibiotic therapy outside of hospital.[68, 86, 93, 107] However, there were also negative perceptions in relation to practitioner involvement. Most GPs saw no advantage to themselves in home treatment, and many thought distance from hospital was an issue for patients.[93] Similarly, nurses perceived that there were challenges in providing this model of care, mainly around the technical nature of the devices used, and dealing with patients' understanding of the technology and its related risks.[68] Finally, many specialists saw organisational barriers to the use of OPAT, in terms of funding, leadership, and the links between primary and secondary care.[63, 86-87, 107] In addition, there was no consensus regarding who should assume the cost or take clinical responsibility for patients.[87, 107]

For peer review only - http://bmjopen.bmj.com/site/about/guidelines.xhtml

DISCUSSION

Principal findings

This review provides the most comprehensive picture of the research published on OPAT in recent years. It brings together available evidence on the clinical and cost-effectiveness, safety and acceptability of community-based antibiotic delivery, and as such it is a valuable addition to the literature in this area. It has established that there are no systematic differences in relation to the impact of OPAT on duration of therapy, or on adverse events associated with IV antibiotic treatment, and that on the whole, OPAT is more cost-effective than inpatient care. However, conclusive evidence as to the clinical benefit (or otherwise) of this mode of therapy compared with traditional inpatient IV treatment is lacking. Acceptability of OPAT appears to be high among patients, who appreciate the greater freedom that it provides, particularly in relation to being able to resume daily activities (such as going to work or school), having greater control over their illness, and not having to attend hospital but being able to stay at home with family. Disadvantages identified most commonly related to the use of infusion equipment. Few studies considered practitioner acceptability, but those that did found some concerns related to the logistics involved in providing an OPAT service, including cost and who would assume clinical responsibility for patients.

Limitations of the study

Although many studies were identified and included in this review, its conclusions are limited by the lack of studies involving a usual care comparison, or comparison with other models of OPAT delivery. In addition, few studies employed a rigorous study design. Much of the work in this area appears to be based around service evaluation, and as such many of the studies provided only basic descriptive findings, with no estimates of variance and limited data related to patient outcomes. As a result, it is difficult to grade the quality and robustness of the evidence, even in the few randomised controlled trials that have been conducted. Similarly, substantial clinical and methodological heterogeneity precluded pooling of data for meta-analysis. The majority of studies included a varied case-mix and did not differentiate their results between conditions treated. In addition, there was variation across studies in relation to what constituted a successful outcome (cure, improvement, deterioration etc.) as well as a lack of consistent treatment duration. Although this undoubtedly reflects real-world practise, it meant that it was not possible to pool the results of individual studies to provide estimates of true effect size when using OPAT for different patient groups, or even for comparing OPAT as a whole with inpatient treatment. Although identified as part of the search strategy, time constraints meant that Non-English language papers were not assessed beyond the abstract screening stage, and it is possible that this may have resulted in some language bias (through

For peer review only - http://bmjopen.bmj.com/site/about/guidelines.xhtml

exclusion of negative findings that may be more likely to have been published in non-international journals). However, given the lack of robust, conclusive studies that were included, this is unlikely to have significantly influenced the findings of the review.

Implications for clinicians and policymakers

It is likely that the increased use of OPAT in the UK in recent years is due, in no small part, to clinician's expectations that it should increase hospital inpatient capacity whilst delivering better patient care and improved patient experience. However, this review has demonstrated that the evidence for better care is not strong. Few studies reported on the different levels of service required to account for the complexity of patient cases encountered, including those with co-morbidity and those requiring 'one off' or longer term treatment. In addition, many studies provided aggregated results and it was not possible to disentangle these either for individual OPAT models or for the specific conditions treated. This, combined with a lack of detail on the delivery models actually used, makes it difficult for clinicians and policymakers to be able to replicate existing practice (and consequently potential outcomes) even from positive studies. OPAT services have the potential to deliver significant costs savings and increased patient satisfaction for the NHS, and it is crucial that this information is reported in future studies in order to identify best practice and help support decision making at a local level.

Future research

Commissioning healthcare services in England is increasingly complex and has been subject to much change in recent years.[139] The NHS England service specification for Infectious Diseases services[140] includes a requirement to provide OPAT, but at present there is no clear commissioning mechanism to achieve this. This review has established that there is currently a dearth of information around key aspects of OPAT, and it is therefore clear that where Clinical Commissioning Groups have opted to commission OPAT services, they are working without the support of a robust evidence-base. It is also clear that there is little evidence upon which recommendations for best practice can be based. Indeed, the main guidance currently in use is the British Society for Antimicrobial Chemotherapy OPAT standards document,[141] which is a consensus statement largely derived from expert opinion.

There is a need for further research to address significant gaps in knowledge about the cost-effectiveness of different IV antibiotic services, and to ascertain which services patients prefer and which aspects of the services are most important to them. This would help identify the most appropriate configuration of services in terms of value for money and patient preference, as well as future research priorities. Since OPAT services are now established in many areas

For peer review only - http://bmjopen.bmj.com/site/about/guidelines.xhtml

of the UK, conducting a definite randomised controlled trial of different models of care is unlikely to be feasible. Individual centres would not be able to provide all of the necessary comparator services for randomisation, and there would be difficulties related to the heterogeneous nature of the patient population, and consequent decisions about the most relevant outcome measure. Other, more pragmatic, study designs should therefore be considered, including large-scale, prospective observational studies designed to collect robust data on outcomes, adverse events, and the costs of OPAT (including mixed models of care). This, together with high standards of reporting to ensure that delivery models and their results are fully described and reported individually, would go some way to bridging the current information gap.

For peer review only

For peer review only - http://bmjopen.bmj.com/site/about/guidelines.xhtml

Acknowledgements: The authors would like to thank Thomas Veale for help in obtaining papers, Jill Edwards for help with screening abstracts, and Kate Hill for assistance with data extraction. We would also like to thank the members of the CIVAS Patient Advisory Group and Expert Panel.

Contributors: The review was designed by CCM, JM, JW and MT as part of a larger study. JW developed and ran the electronic searches. Data extraction tools were designed by EDM. EDM, CCM, DM and MT were involved in record screening and study selection. EDM and CCM undertook data abstraction and carried out assessment of risk of bias. EDM synthesized the data. The article was drafted by EDM, and all authors contributed to subsequent drafts. EDM is guarantor.

Funding: This work was supported by NIHR Health Services Research and Delivery (HSDR), Project reference 11/2003/60. NIHR had no role in design, conduct, analysis, interpretation of data, or the decision to submit for publication.

Competing interests: All authors have completed the Unified Competing Interest form at www.icmje.org/coi_disclosure.pdf (available on request from the corresponding author) and declare: this work was funded by The National Institute for Health Research; no financial relationships with any organization that might have an interest in the submitted work in the previous three years; no other relationships or activities that could appear to have influenced the submitted work.

Ethical approval: Not required: systematic review.

Data sharing: No additional data are available.

Review registration: This study is registered as PROSPERO CRD42013006374 (available at www.crd.york.ac.uk/PROSPERO/).

For peer review only

REFERENCES

1. Paladino J, Portez D. Outpatient parenteral antimicrobial therapy today. Clinical Infectious Diseases 2010; 51: S198-208.

2. Chary A, Tice AD, Martinelli LP, Liedtke LA, Plantenga MS, Strausbaugh LJ. Experience of infectious diseases consultants with outpatient parenteral antimicrobial therapy: results of an emerging infections network survey. Clinical Infectious Diseases 2006; 43(10):1290-95.

3. Torok M, Chapman A, Lessing M, Sanderson F, Seaton R. Outpatient parenteral antimicrobial therapy: Recent developments and future prospects. Current Opinion in Investigational Drugs 2010; 11: 929-39.

4. Chapman ALN. Outpatient parenteral antimicrobial therapy. BMJ 2013; 346: f1585.

5. Department of Health. National Health Service and Community Care Act 1990. The Stationary Office: London, 1990.

6. Higgins JPT, Green S (eds.). Cochrane handbook for systematic reviews of interventions. Version 5.1.0. Cochrane Collaboration, 2008. Available at: http://handbook.cochrane.org (accessed 11 July 2016).

7. Wells GA, Shea B, O'Connell D, et al. The Newcastle-Ottawa Scale (NOS) for assessing the quality of nonrandomised studies in meta-analyses. Available at: http://www.ohri.ca/programs/clinical_epidemiology/oxford.htm (accessed 11 July 2016).

8. Mitchell ED, Pickwell-Smith B, Macleod U. Risk factors for emergency presentation with lung and colorectal cancers: a systematic review. BMJ Open 2015; 5: 4 e006965.

9. Macleod U, Mitchell ED, Burgess C, Macdonald S, Ramirez AJ. Risk factors for delayed presentation and referral of symptomatic cancer: evidence for common cancers. Br J Cancer 2009; 101: S92-S101.

10. Mitchell E, Macdonald S, Campbell NC, Weller D, Macleod U. Influences on pre-hospital delay in the diagnosis of colorectal cancer: a systematic review. Br J Cancer 2008; 98: 60-70.

11. Al Ansari A, Al Alawi S, Al Qahtani M, Darwish A. Outpatient Parenteral Antimicrobial Therapy (OPAT) in the Kingdom of Bahrain: efficacy, patient satisfaction and cost effectiveness. The Open Infectious Diseases Journal 2013; 7(1): 90-95.

12. Allison GM, Muldoon EG, Kent DM, et al. Prediction model for 30-day hospital readmissions among patients discharged receiving outpatient parenteral antibiotic therapy. Clinical Infectious Diseases 2014; 58(6): 812-19.

13. Amodeo MR, Clulow T, Lainchbury J, et al. Outpatient intravenous treatment for infective endocarditis: safety, effectiveness and one-year outcomes. Journal of Infection 2009; 59(6): 387-93.

14. Anand V, Levine H, Friedman M, et al. Intravenous antibiotics for refractory rhinosinusitis in nonsurgical patients: preliminary findings of a prospective study. American Journal of Rhinology 2003; 17(6): 363-68.

15. Angel JV. Outpatient antibiotic therapy for elderly patients. HIAT Study Group. American Journal of Medicine 1994; 97(2A): 43-49.

16. Bamford KB, Desai M, Aruede MJ, Lawson W, Jacklin A, Franklin BD. Patients' views and experience of intravenous and oral antimicrobial therapy: room for change. Injury 2011; 42(Suppl 5): S24-27.

17. Barr DA, Semple L, Seaton RA. Self-administration of outpatient parenteral antibiotic therapy and risk of catheter-related adverse events: a retrospective cohort study. European Journal of Clinical Microbiology & Infectious Diseases 2012; 31(10): 2611-19.

18. Barr DA, Semple L, Seaton RA. Outpatient parenteral antimicrobial therapy (OPAT) in a teaching hospital-based practice: a retrospective cohort study describing experience and evolution over 10 years. International Journal of Antimicrobial Agents 2012; 39(5): 407-13.

19. Barr DA, Irvine S, Ritchie NS, McCutcheon J, Seaton RA. Risk of venous thromboembolism in patients treated for bacterial infection in the community with outpatient parenteral antimicrobial therapy. QJM 2014; 107(3): 207-11.

For peer review only - http://bmjopen.bmj.com/site/about/guidelines.xhtml

20. Bedi P, Sidhu MK, Donaldson LS, et al. A prospective cohort study of the use of domiciliary intravenous antibiotics in bronchiectasis. Primary Care Respiratory Medicine 2014; 24: 14090.

21. Berman SJ, Johnson EW. Out-patient parenteral antibiotic therapy (OPAT): clinical outcomes and adverse events. Hawaii Medical Journal 2001; 60(2): 31-33.

22. Bernard L, El H, Pron B, et al. Outpatient parenteral antimicrobial therapy (OPAT) for the treatment of osteomyelitis: evaluation of efficacy, tolerance and cost. Journal of Clinical Pharmacy & Therapeutics 2001; 26(6): 445-51.

23. Bradley JM, Wallace ES, Elborn JS, Howard JL, McCoy MP. An audit of the effect of intravenous antibiotic treatment on spirometric measures of pulmonary function in cystic fibrosis. Irish Journal of Medical Science 1999; 168(1): 25-28.

24. Cervera C, Del Rio A, Garcia L, et al. Efficacy and safety of outpatient parenteral antibiotic therapy for infective endocarditis: a ten-year prospective study. Enfermedades Infecciosas y Microbiologia Clinica 2011; 29(8): 587-92.

25. Chambers S, Gallagher K, Metcalf S, Pithie A. Home intravenous antimicrobial service--twelve months experience in Christchurch. New Zealand Medical Journal 2002; 115: 216-18.

26. Chambers S, Gallagher K, Pithie A. Patient acceptability of home intravenous antibiotic therapy. New Zealand Medical Journal 2004; 117: U865.

27. Chan DSG, Archuleta S, Llorin RM, Lye DC, Fisher D. Standardized outpatient management of Klebsiella pneumoniae liver abscesses. International Journal of Infectious Diseases 2013; 17(3): e185-e188.

28. Chapman ALN, Dixon S, Andrews D, Lillie PJ, Bazaz R, Patchett JD. Clinical efficacy and cost-effectiveness of outpatient parenteral antibiotic therapy (OPAT): a UK perspective. Journal of Antimicrobial Chemotherapy 2009; 64(6): 1316-24.

29. Cheong EA, Katelaris CH, Sisson CM, Anderson EA, Byth K. Adverse drug reactions associated with home parenteral therapy. Journal of Pharmacy Practice and Research 2008; 38(4): 267-70.

30. Corwin P, Toop L, McGeoch G, et al. Randomised controlled trial of intravenous antibiotic treatment for cellulitis at home compared with hospital. BMJ 2005; 330: 129-34..

31. Cox AM, Malani PN, Wiseman SW, Kauffman CA. Home intravenous antimicrobial infusion therapy: a viable option in older adults. Journal of the American Geriatrics Society 2007; 55(5): 645-50.

32. Dall L, Peddicord T, Peterson S, Simmons T, Dall T. Hospitalist treatment of CAP and cellulitis using objective criteria to select patients. Infections in Medicine 2003; 20(8): 379-90.

33. Dalovisio JR, Juneau J, Baumgarten K, Kateiva J. Financial impact of a home intravenous antibiotic program on a medicare managed care program. Clinical Infectious Diseases 2000; 30(4): 639-42.

34. Dargan S, Zvonar RK, Saginur R. A review of outpatient parenteral antimicrobial therapy practices and experience at the Ottawa Hospital. Canadian Journal of Hospital Pharmacy 2007; 60(3): 177-83.

35. Dobson PM, Boyle M, Loewenthal M. Home intravenous antibiotic therapy and allergic drug reactions: is there a case for routine supply of anaphylaxis kits? Journal of Infusion Nursing 2004; 27(6): 425-30.

36. Donald M, Marlow N, Swinburn E, Wu M. Emergency department management of home intravenous antibiotic therapy for cellulitis. Emergency Medicine Journal 2005; 22(10): 715-17.

37. Duncan CJA, Barr DA, Seaton RA. Outpatient parenteral antimicrobial therapy with ceftriaxone, a review. International Journal of Clinical Pharmacy 2012; 34(3): 410-17.

38. Duncan CJA, Barr DA, Ho A, Sharp E, Semple L, Seaton RA. Risk factors for failure of outpatient parenteral antibiotic therapy (OPAT) in infective endocarditis. Journal of Antimicrobial Chemotherapy 2013; 68(7): 1650-54.

39. Escalante CP, Rubenstein EB, Rolston KV. Outpatient antibiotic therapy for febrile episodes in low-risk neutropenic patients with cancer. Cancer Investigation 1997; 15(3): 237-42.

40. Esmond G, Butler M, McCormack AM. Comparison of hospital and home intravenous antibiotic therapy in adults with cystic fibrosis. Journal of Clinical Nursing 2006; 15(1): 52-60.

For peer review only

For peer review only - http://bmjopen.bmj.com/site/about/guidelines.xhtml

41. Esposito S, Noviello S, Leone S, et al. Outpatient parenteral antibiotic therapy (OPAT) in different countries: a comparison. International Journal of Antimicrobial Agents 2004; 24(5): 473-78.

42. Esposito S, Leone S, Noviello S, et al. Outpatient parenteral antibiotic therapy for bone and joint infections: an Italian multicenter study. Journal of Chemotherapy 2007; 19(4): 417-22.

43. Esposito S, Leone S, Noviello S, et al. Outpatient parenteral antibiotic therapy in the elderly: an Italian observational multicenter study. Journal of Chemotherapy 2009; 21(2): 193-98.

44. Fernandez-Aviles F, Carreras E, Urbano-Ispizua A, et al. Case-control comparison of at-home to total hospital care for autologous stem-cell transplantation for hematologic malignancies. Journal of Clinical Oncology 2006; 24(30): 4855-61.

45. Goodwin DD, Hanson JC, Berry CP. The changing face of Canadian home parenteral therapy. Journal of Infusion Nursing 2002; 25(6): 372-78.

46. Gourdeau M, Deschenes L, Caron M, Desmarais M. Home iv antibiotic therapy through a medical day care unit. Canadian Journal of Infectious Diseases 1993; 4(3): 158-62.

47. Graninger W, Presterl E, Wenisch C, Schwameis E, Breyer S, Vukovich T. Management of serious staphylococcal infections in the outpatient setting. Drugs 1997; 54 (Suppl 6): 21-28.

48. Grayson ML, Silvers J, Turnidge J. Home intravenous antibiotic therapy. A safe and effective alternative to inpatient care. Medical Journal of Australia 1995; 162(5): 249-53.

49. Gross R, Graziani AL, Laufer D, Turner JL, Ondercin JP, Macgregor RR. Adverse effects of the use of intravenous pentamidine in the home. Infectious Diseases in Clinical Practice 1996; 5(7): 456-58.

50. Heintz BH, Halilovic J, Christensen CL. Impact of a multidisciplinary team review of potential outpatient parenteral antimicrobial therapy prior to discharge from an academic medical center. Annals of Pharmacotherapy 2011; 45(11): 1329-37.

51. Hindes R, Winkler C, Kane P, Kunkel M, Poretz DM. Outpatient intravenous antibiotic therapy in Medicare patients: Cost-savings analysis. Infectious Diseases in Clinical Practice 1995; 4(3): 211-18.

52. Hitchcock J, Jepson AP, Main J, Wickens HJ. Establishment of an outpatient and home parenteral antimicrobial therapy service at a London teaching hospital: a case series. Journal of Antimicrobial Chemotherapy 2009; 64(3): 630-34.

53. Ho J, Archuleta S, Sulaiman Z, Fisher D. Safe and successful treatment of intravenous drug users with a peripherally inserted central catheter in an outpatient parenteral antibiotic treatment service. Journal of Antimicrobial Chemotherapy 2010; 65(12): 2641-44.

54. Htin KF, Friedman ND, Hughes A, et al. Outpatient parenteral antimicrobial therapy is safe and effective for the treatment of infective endocarditis: a retrospective cohort study. Internal Medicine Journal 2013; 43(6): 700-05.

55. Huminer D, Bishara J, Pitlik S. Home intravenous antibiotic therapy for patients with infective endocarditis. European Journal of Clinical Microbiology & Infectious Diseases 1999; 18(5): 330-34.

56. Johansson E, Bjorkholm M, Wredling R, Kalin M, Engervall P. Outpatient parenteral antibiotic therapy in patients with haematological malignancies. A pilot study of an early discharge strategy. Supportive Care in Cancer 2001; 9(8): 619-24.

57. Kayley J, Berendt AR, Snelling MJ, et al. Safe intravenous antibiotic therapy at home: experience of a UK based programme. Journal of Antimicrobial Chemotherapy 1996; 37(5): 1023-29.

58. Keller SC, Ciuffetelli D, Bilker W, et al. The impact of an Infectious Diseases Transition Service on the care of outpatients on parenteral antimicrobial therapy. Journal of Pharmacy Technology 2013; 29(5): 205-14.

59. Kieran J, O'Reilly A, Parker J, Clarke S, Bergin C. Self-administered outpatient parenteral antimicrobial therapy: a report of three year's experience in the Irish healthcare setting. European Journal of Clinical Microbiology & Infectious Diseases 2009; 28(11): 1369-74.

60. Krauth C, Jalilvand N, Welte T, Busse R. Cystic fibrosis: cost of illness and considerations for the economic evaluation of potential therapies. Pharmacoeconomics 2003; 21(14): 1001-24.

61. Lacroix A, Revesta M, Patrat-Delon S, et al. Outpatient parenteral antimicrobial therapy for infective endocarditis: A cost-effective strategy. Medecine et Maladies Infectieuses 2014; 44(7): 327-30.

For peer review only - http://bmjopen.bmj.com/site/about/guidelines.xhtml

62. Lai A, Tran T, Nguyen HM, Fleischmann J, Beenhouwer DO, Graber CJ. Outpatient parenteral antimicrobial therapy at large Veterans Administration medical center. The American Journal of Managed Care 2013; 19(9): e317-e324.

63. Lane MA, Marschall J, Beekmann SE, et al. Outpatient parenteral antimicrobial therapy practices among adult infectious disease physicians. Infection Control and Hospital Epidemiology 2014; 35(7): 839-44.

64. Larioza J, Heung L, Girard A, Brown RB. Management of infective endocarditis in outpatients: clinical experience with outpatient parenteral antibiotic therapy. Southern Medical Journal 2009; 102(6): 575-79.

65. Larioza J, Girard A, Brown RB. Clinical experience with daptomycin for outpatient parenteral antibiotic therapy. American Journal of the Medical Sciences 2011; 342(6): 486-88.

66. Laupland KB, Gill MJ, Schenk L, Goodwin D, Davies HD. Outpatient parenteral antibiotic therapy: evolution of the Calgary adult home parenteral therapy program. Clinical & Investigative Medicine - Medecine Clinique et Experimentale 2002; 25(5): 185-90.

67. Lehoux P. Patients' perspectives on high-tech home care: a qualitative inquiry into the user-friendliness of four technologies. BMC Health Services Research 2004; 4(1): 28.

68. Lehoux P, Richard L, Pineault R, Saint-Arnaud J. Delivery of high-tech home care by hospital-based nursing units in Quebec: clinical and technical challenges. Nursing leadership (Toronto, Ont.) 2006; 19(1): 44-55.

69. Lillie PJ, Andrews D, Eaves K, Darton TC, Chapman ALN. Baseline factors predicting the duration of intravenous antibiotic therapy for cellulitis in an outpatient setting. European Journal of Clinical Microbiology & Infectious Diseases 2010; 29(3): 347-49.

70. Lin JW, Kacker A, Anand VK, Levine H. Catheter- and antibiotic-related complications of ambulatory intravenous antibiotic therapy for chronic refractory rhinosinusitis. American Journal of Rhinology 2005; 19(4): 365-69.

71. Lopardo G. Management of endocarditis: outpatient parenteral antibiotic treatment in Argentina. Chemotherapy 2001; 47(Suppl 1): 24-32.

72. Mackintosh CL, White HA, Seaton RA. Outpatient parenteral antibiotic therapy (OPAT) for bone and joint infections: experience from a UK teaching hospital-based service. Journal of Antimicrobial Chemotherapy 2011; 66(2): 408-15.

73. Marra CA, Frighetto L, Goodfellow AF, et al. Willingness to pay to assess patient preferences for therapy in a Canadian setting. BMC Health Services Research 2005; 5: 43.

74. Martel AY. Home intravenous self-injection of antibiotic therapy. Canadian Journal of Infectious Diseases 1994; 5(Suppl. C): 51C-55C.

75. Martone WJ, Lindfield KC, Katz DE. Outpatient parenteral antibiotic therapy with daptomycin: insights from a patient registry. International Journal of Clinical Practice 2008; 62(8): 1183-87.

76. Matthews PC, Conlon CP, Berendt AR, et al. Outpatient parenteral antimicrobial therapy (OPAT): is it safe for selected patients to self-administer at home? A retrospective analysis of a large cohort over 13 years. Journal of Antimicrobial Chemotherapy 2007; 60(2): 356-62.

77. Mauceri AA. Treatment of bone and joint infections utilizing a third-generation cephalosporin with an outpatient drug delivery device. HIAT Study Group. American Journal of Medicine 1994; 97(2A): 14-22.

78. Mazo S, Emparan C, Vallejo M, Soriano P. Hospital-in-the-home treatment of surgical infectious diseases: an economic analysis. Surgical Infections 2007; 8(6): 567-74.

79. McMahon JH, O'Keeffe JM, Victorian Hith Outcomes Study G, Grayson ML. Is hospital-in-the-home (HITH) treatment of bacterial endocarditis safe and effective? Scandinavian Journal of Infectious Diseases 2008; 40(1): 40-43.

80. Mohammadi S, MacKay K, Ward TT, Forrest GN. Clinical outcomes of a Veterans Affairs outpatient antimicrobial treatment program. Southern Medical Journal 2013; 106(6): 345-49.

81. Montalto M, Dunt D. Home and hospital intravenous therapy for two acute infections: an early study. Australian & New Zealand Journal of Medicine 1997; 27(1): 19-23.

For peer review only - http://bmjopen.bmj.com/site/about/guidelines.xhtml

82. Montalto M. Patients' and carers' satisfaction with hospital-in-the-home care. International Journal for Quality in Health Care 1996; 8(3): 243-51.

83. Montalto M. An audit of patients admitted for home intravenous therapy directly from the emergency department. International Journal of Clinical Practice 1997; 51(7): 433-37.

84. Montalto M, Lui B, Mullins A, Woodmason K. Medically-managed Hospital in the Home: 7 year study of mortality and unplanned interruption. Australian Health Review 2010; 34(3): 269-75.

85. Morales JO, von Behren L. Secondary bacterial infections in HIV-infected patients: an alternative ambulatory outpatient treatment utilizing intravenous cefotaxime. American Journal of Medicine 1994; 97(2A): 9-13.

86. Muldoon EG, Allison GM, Gallagher D, Snydman DR, Bergin C. Outpatient parenteral antimicrobial therapy (OPAT) in the republic of Ireland: results of a national survey. European Journal of Clinical Microbiology & Infectious Diseases 2013; 32(11): 1465-70.

87. Muldoon EG, Switkowski K, Tice A, Snydman DR, Allison GM. A national survey of infectious disease practitioners on their use of outpatient parenteral antimicrobial therapy (OPAT). Infectious Diseases 2015; 47(1): 39-45.

88. Murray H, Stiell I, Wells G. Treatment failure in emergency department patients with cellulitis. Canadian Journal of Emergency Medicine 2005; 7(4): 228-34.

89. Nathwani D, Morrison J, Seaton RA, France AJ, Davey P. Out-patient and home-parental antibiotic therapy (OHPAT): Evaluation of the impact of one year's experience in Tayside. Health Bulletin 1999; 57(5): 332-37.

90. Nathwani D. The management of skin and soft tissue infections: outpatient parenteral antibiotic therapy in the United Kingdom. Chemotherapy 2001; 47(Suppl 1): 17-23.

91. Nathwani D, Barlow GD, Ajdukiewicz K, et al. Cost-minimization analysis and audit of antibiotic management of bone and joint infections with ambulatory teicoplanin, in-patient care or outpatient oral linezolid therapy. Journal of Antimicrobial Chemotherapy 2003; 51(2): 391-96.

92. Pajarón M, Fernández-Miera MF, Allende I, et al. Self-administered outpatient parenteral antimicrobial therapy (S-OPAT) for infective endocarditis: A safe and effective model. European Journal of Internal Medicine 2015; 26(2): 131-36.

93. Parker SE, Nathwani D, O'Reilly D, Parkinson S, Davey PG. Evaluation of the impact of non-inpatient IV antibiotic treatment for acute infections on the hospital, primary care services and the patient. Journal of Antimicrobial Chemotherapy 1998; 42(3): 373-80.

94. Partridge DG, O'Brien E, Chapman ALN. Outpatient parenteral antibiotic therapy for infective endocarditis: a review of 4 years' experience at a UK centre. Postgraduate Medical Journal 2012; 88: 377-81.

95. Patanwala AE, Erstad BL, Nix DE. Cost-effectiveness of linezolid and vancomycin in the treatment of surgical site infections. Current Medical Research and Opinion 2007; 23(1): 185-93.

96. Perez-Lopez J, San Jose Laporte A, Pardos-Gea J, et al. Safety and efficacy of home intravenous antimicrobial infusion therapy in older patients: a comparative study with younger patients. International Journal of Clinical Practice 2008; 62(8): 1188-92.

97. Pilling M, Walley T. Parenteral antibiotics at home in cystic fibrosis: experiences and attitudes of recipients. Health & Social Care in the Community 1997; 5(3): 209-12.

98. Pond MN, Newport M, Joanes D, Conway SP. Home versus hospital intravenous antibiotic therapy in the treatment of young adults with cystic fibrosis. European Respiratory Journal 1994; 7(9): 1640-44.

99. Poretz DM. Treatment of serious infections with cefotaxime utilizing an outpatient drug delivery device: global analysis of a large-scale, multicenter trial. HIAT Study Group. American Journal of Medicine 1994; 97(2A): 34-42.

100. Poretz DM. Treatment of skin and soft-tissue infections utilizing an outpatient parenteral drug delivery device: a multicenter trial. HIAT Study Group. American Journal of Medicine 1994; 97(2A): 23-27.

For peer review only - http://bmjopen.bmj.com/site/about/guidelines.xhtml

101. Rapoport BL, Sussmann O, Herrera MV, et al. Ceftriaxone plus once daily aminoglycoside with filgrastim for treatment of febrile neutropenia: Early hospital discharge vs. standard in-patient care. Chemotherapy 1999; 45(6): 466-76.

102. Rehm S, Campion M, Katz DE, Russo R, Boucher HW. Community-based outpatient parenteral antimicrobial therapy (CoPAT) for Staphylococcus aureus bacteraemia with or without infective endocarditis: analysis of the randomized trial comparing daptomycin with standard therapy. Journal of Antimicrobial Chemotherapy 2009; 63(5): 1034-42.

103. Richards DA, Toop LJ, Epton MJ, et al. Home management of mild to moderately severe community-acquired pneumonia: a randomised controlled trial. Medical Journal of Australia 2005; 183(5): 235-38.

104. Rodriguez-Cerrillo M, Poza-Montoro A, Fernandez-Diaz E, Romero AI. Patients with uncomplicated diverticulitis and comorbidity can be treated at home. European Journal of Internal Medicine 2010; 21(6): 553-54.

105. Rodriguez-Cerrillo M, Poza-Montoro A, Fernandez-Diaz E, Inurrieta-Romero A, Matesanz-David M. Home treatment of patients with acute cholecystitis. European Journal of Internal Medicine 2012; 23(1): e10-e13.

106. Seaton RA, MacConnachie AA. Experience with daptomycin in an infectious diseases service over 1 year: utility in an outpatient parenteral antibiotic programme. International Journal of Antimicrobial Agents 2008; 31(5): 492-97.

107. Seaton RA, Nathwani D. Outpatient and home parenteral antibiotic therapy (OHPAT) in the UK: survey of infection specialists' experience and views. Clinical Microbiology & Infection 2000; 6(7): 387-90.

108. Seaton RA, Nathwani D, Williams FL, Boyter AC. Feasibility of an outpatient and home parenteral antibiotic therapy (OHPAT) programme in Tayside, Scotland. Journal of Infection 1999; 39(2): 129-33.

109. Seaton RA, Bell E, Gourlay Y, Semple L. Nurse-led management of uncomplicated cellulitis in the community: evaluation of a protocol incorporating intravenous ceftriaxone. Journal of Antimicrobial Chemotherapy 2005; 55(5): 764-67.

110. Seaton RA, Sharp E, Bezlyak V, Weir CJ. Factors associated with outcome and duration of therapy in outpatient parenteral antibiotic therapy (OPAT) patients with skin and soft-tissue infections. International Journal of Antimicrobial Agents 2011; 38(3): 243-48.

111. Sebban C, Dussart S, Fuhrmann C, et al. Oral moxifloxacin or intravenous ceftriaxone for the treatment of low-risk neutropenic fever in cancer patients suitable for early hospital discharge. Supportive Care in Cancer 2008; 16(9): 1017-23.

112. Seetoh T, Lye DC, Cook AR, et al. An outcomes analysis of outpatient parenteral antibiotic therapy (OPAT) in a large Asian cohort. International Journal of Antimicrobial Agents 2013; 41(6): 569-73.

113. Sims AL, Baker P, Bellamy R, McMurtry IA. Outpatient parenteral antibiotic therapy in primary hip and knee arthroplasty infection managed with debridement and retention of prosthesis: a retrospective cohort study. Surgical Infections 2013; 14(3): 293-96.

114. Smego RA, Khan MA, Khowaja K, Rafique R, Datoo F. A university-sponsored Home Health Nursing Program in Karachi, Pakistan. Home Healthcare Nurse 2005; 23(11): 710-16.

115. South R. Retrospective study of teicoplanin as home continuation of hospital-initiated therapy. International Journal of Antimicrobial Agents 1998; 9(4): 219-25.

116. Stein GE, Schooley SL, Havlichek DH, Nix DE. Outpatient intravenous antibiotic therapy compared with oral linezolid in patients with skin and soft tissue infections: A pharmacoeconomic analysis. Infectious Diseases in Clinical Practice 2008; 16(4): 235-39.

117. Subedi S, Looke DFM, McDougall DA, Sehu MM, Playford EG. Supervised self-administration of outpatient parenteral antibiotic therapy: a report from a large tertiary hospital in Australia. International Journal of Infectious Diseases 2015; 30: 161-65.

118. Talcott JA, Whalen A, Clark J, Rieker PP, Finberg R. Home antibiotic therapy for low-risk cancer patients with fever and neutropenia: a pilot study of 30 patients based on a validated prediction rule. Journal of Clinical Oncology 1994; 12(1): 107-14.

For peer review only

119. Teuffel O, Amir E, Alibhai S, Beyene J, Sung L. Cost effectiveness of outpatient treatment for febrile neutropaenia in adult cancer patients. British Journal of Cancer 2011; 104(9): 1377-83.

120. Teuffel O, Cheng S, Ethier MC, et al. Health-related quality of life anticipated with different management strategies for febrile neutropenia in adult cancer patients. Supportive Care in Cancer 2012; 20(11): 2755-64.

121. Theocharis G, Rafailidis PI, Rodis D, Kontopidis I, Barbas SG, Falagas ME. Outpatient parenteral antibiotic therapy (OPAT) at home in Attica, Greece. European Journal of Clinical Microbiology & Infectious Diseases 2012; 31(11): 2957-61.

122. Thornton J, Elliott RA, Tully MP, Dodd M, Webb AK. Clinical and economic choices in the treatment of respiratory infections in cystic fibrosis: comparing hospital and home care. Journal of Cystic Fibrosis 2005; 4(4): 239-47.

123. Tice AD. Experience with a physician-directed, clinic-based program for outpatient parenteral antibiotic therapy in the USA. European Journal of Clinical Microbiology & Infectious Diseases 1995; 14(7): 655-61.

124. Tice A. The use of outpatient parenteral antimicrobial therapy in the management of osteomyelitis: data from the Outpatient Parenteral Antimicrobial Therapy Outcomes Registries. Chemotherapy 2001; 47(Suppl 1): 5-16.

125. Tice AD, Hoaglund PA, Nolet B, Mckinnon PS, Mozaffari E. Cost perspectives for outpatient intravenous antimicrobial therapy. Pharmacotherapy: The Journal of Human Pharmacology & Drug Therapy 2002; 22(2 Pt 2): 63S-70S.

126. Upton A, Ellis-Pegler RB, Woodhouse AF. Outpatient parenteral antimicrobial therapy (OPAT): A review of experience at Auckland Hospital. New Zealand Medical Journal 2004; 117: 1200.

127. Walton AL, Howden BP, Grayson LM, Korman TM. Continuous-infusion penicillin home-based therapy for serious infections due to penicillin-susceptible pathogens. International Journal of Antimicrobial Agents 2007; 29(5): 544-48.

128. White HA, Davis JS, Kittler P, Currie BJ. Outpatient parenteral antimicrobial therapy-treated bone and joint infections in a tropical setting. Internal Medicine Journal 2011; 41(9): 668-73.

129. White B, Seaton RA, Evans TJ. Management of suspected lyme borreliosis: Experience from an outpatient parenteral antibiotic therapy service. QJM 2013; 106(2): 133-38.

130. Williams DN. Home intravenous antibiotic therapy (HIVAT): Indications, patients and antimicrobial agents. International Journal of Antimicrobial Agents 1995; 5(1): 3-8.

131. Wolter JM, Bowler SD, Nolan PJ, McCormack JG. Home intravenous therapy in cystic fibrosis: a prospective randomized trial examining clinical, quality of life and cost aspects. European Respiratory Journal 1997; 10(4): 896-900.

132. Wolter JM, Cagney RA, McCormack JG. A randomized trial of home vs hospital intravenous antibiotic therapy in adults with infectious diseases. Journal of Infection 2004; 48(3): 263-68.

133. Wynn M, Dalovisio JR, Tice AD, Jiang X. Evaluation of the efficacy and safety of outpatient parenteral antimicrobial therapy for infections with methicillin-sensitive Staphylococcus aureus. Southern Medical Journal 2005; 98(6): 590-95.

134. Yadlapalli NG, Vaishnav A, Sheehan P. Conservative management of diabetic foot ulcers complicated by osteomyelitis. Wounds: A Compendium of Clinical Research & Practice 2002; 14(1): 31-35.

135. Yan YM, Singh M, Tonks K, Kavi J, Langford NJ. Delivering outpatient antibiotic therapy (OPAT) in an Acute Medical Unit. Acute Medicine 2011; 10(1): 22-25.

136. Yang A, Fung R, Brunton J, Dresser L. Outpatient parenteral antimicrobial therapy for surgery patients: A comparison with previous standard of care. The Canadian Journal of Infectious Diseases & Medical Microbiology 2014; 24(2): 74-78.

137. Yong C, Fisher DA, Sklar GE, Li S-C. A cost analysis of Outpatient Parenteral Antibiotic Therapy (OPAT): an Asian perspective. International Journal of Antimicrobial Agents 2009; 33(1): 46-51.

138. You JHS, Lee GCH, So RKH, Cheung KW, Hui M. Linezolid versus vancomycin for prosthetic joint infections: a cost analysis. Infection 2007; 35(4): 265-70.

For peer review only

For peer review only - http://bmjopen.bmj.com/site/about/guidelines.xhtml

139. Wye L, Brangan E, Cameron A, Gabbay J, Klein J, Pope C. Knowledge exchange in health-care commissioning: case studies of the use of commercial, not-for-profit and public sector agencies, 2011-14. Health Serv Deliv Res 2015; 3(19).

140. NHS England. NHS standard contract for specialised services for infectious diseases (adult), 2013/14. Available at: https://www.england.nhs.uk/wp-content/uploads/2013/06/b07-infec-diseases-adu.pdf (accessed 13 July 2016).

141. Chapman ALN, Seaton RA, Cooper MA et al. Good practice recommendations for outpatient parenteral antibiotic therapy (OPAT) in adults in the UK: a consensus statement. J Antimicrob Chemother 2012; 67(5): 1053-1062.

For peer review only

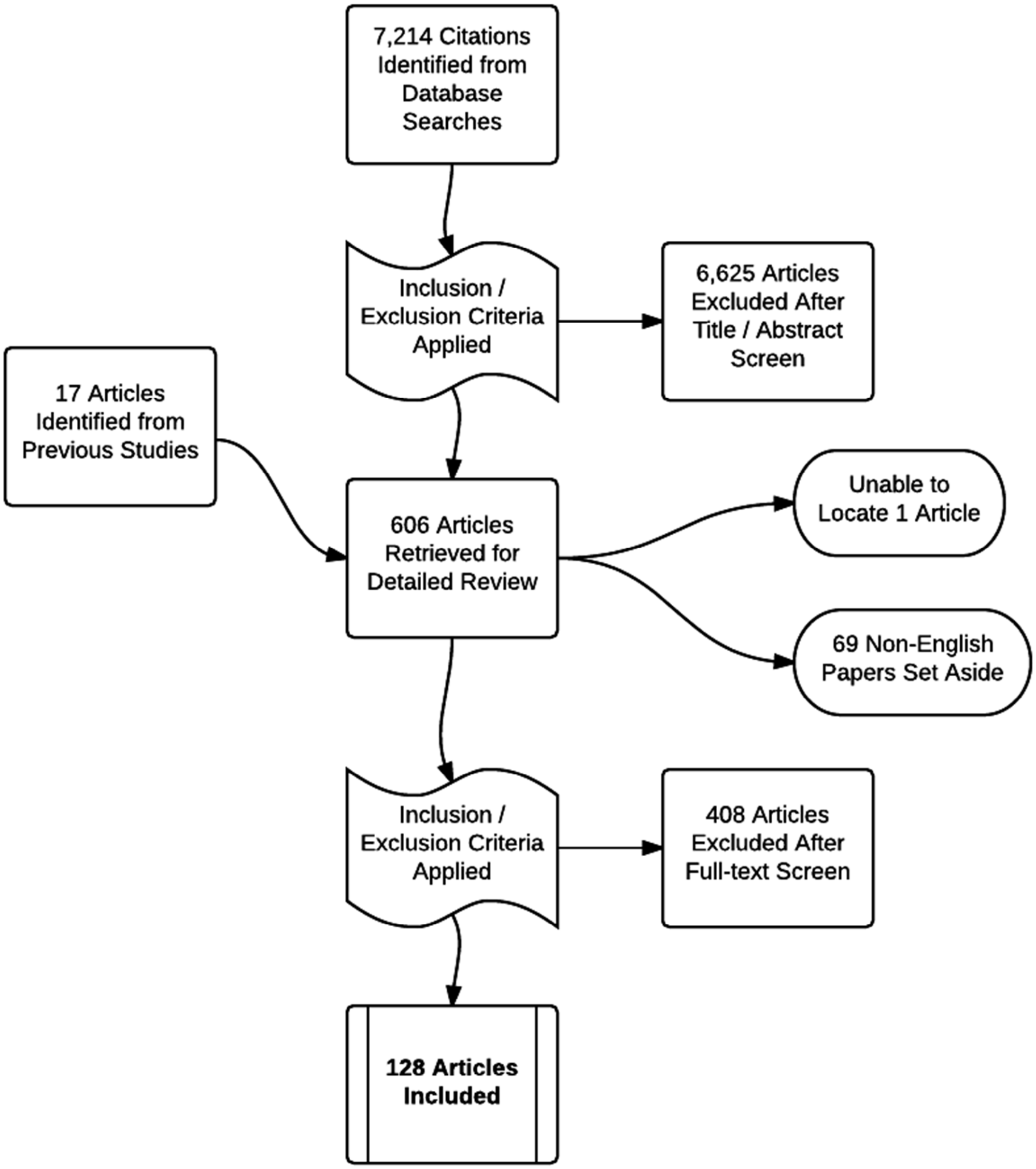

Figure 1 Flow of studies into the review

For peer review only - http://bmjopen.bmj.com/site/about/guidelines.xhtml

TABLE 1 Results for study exclusion

REASON FOR EXCLUSION	NUMBER
Descriptive or commentary piece	118
Non-English language (set aside)	69
Did not consider an OPAT model	50
Abstract only	34
Duplicate paper or study	27
Evidence summary*	26
Focus on effectiveness of a specific antibiotic	26
Reported non-IV antibiotics	20
No distinction between IV antibiotic/other treatments	19
No distinction between adults/children	16
Reported costs only	14
Focus on method or process of delivery	10
Survey but no acceptability data reported	10
Unclear if OPAT model involved IV	8
Lacked outcomes data	6
Studied children only	6
Studied other antimicrobial	6
Guidance document	5
Reported non-specific effectiveness	5
Focus on adherence to guidelines	1
Focus on retention of patient training knowledge	1
Unable to locate article	1

* Relates to reviews that included studies already identified, or were out with the timescale or inclusion criteria for this review. One Cochrane review had no included studies.

For peer review only - http://bmjopen.bmj.com/site/about/guidelines.xhtml

TABLE 2 Characteristics of included studies

Study (location)	Infection(s) treated	OPAT model(s)	Comparator	Population	Outcomes
Al Ansari *et al*, 2013 (Bahrain)[11]	Cellulitis; osteomyelitis; respiratory †	OP	None	101 patients	Clinical effectiveness<br>Patient safety<br>Patient acceptability
Allison *et al*, 2014 (USA)[12]	Bacteraemia; cellulitis; endocarditis; osteomyelitis; septic arthritis †	SN	None	782 patients	Patient safety
Amodeo *et al*, 2009 (New Zealand)[13]	Endocarditis	SA + GN	None	100 treatment courses	Patient safety
Anand *et al*, 2003 (USA)[14]	Sinusitis	Home therapy (unspecified)	None	52 patients	Clinical effectiveness<br>Patient safety
Angel, 1994 (USA)[15]	Osteomyelitis; pneumonia; sepsis; SSTI	SA	None	62 patients	Clinical effectiveness<br>Patient safety
Bamford *et al*, 2011 (UK)[16]	Unspecified	Not specified	None	12 interviewees	Patient acceptability
Barr *et al*, 2012 (UK)[17]	Unspecified	OP + SA	None	854 treatment courses	Patient safety
Barr *et al*, 2012 (UK)[18]	Bacteraemia; endocarditis; osteomyelitis; septic arthritis; SSTI †	OP + SA + SN + GN	None	2,233 patients	Clinical effectiveness<br>Patient safety
Barr *et al*, 2014 (UK)[19]	Abscess; bacteraemia; osteomyelitis; respiratory; septic arthritis; SSTI †	Not specified	None	780 patients	Patient safety
Bedi *et al*, 2014 (UK)[20]	Respiratory	SA	Inpatient therapy	111 patients	Clinical effectiveness<br>Patient safety
Berman & Johnson, 2001 (USA)[21]	Endocarditis; osteomyelitis; respiratory; septic arthritis; SSTI †	SA	None	221 patients	Clinical effectiveness<br>Patient safety
Bernard *et al*, 2001 (France)[22]	Osteomyelitis	SA + SN	None	39 patients	Clinical effectiveness<br>Patient safety<br>Patient acceptability
Bradley *et al*, 1999 (UK)[23]	Cystic fibrosis	SA	Combined therapy<br>Inpatient therapy	51 patients	Clinical effectiveness
Cervera *et al*, 2011 (Spain)[24]	Endocarditis	SA + SN	None	73 patients	Patient safety
Chambers *et al*, 2002 (New Zealand)[25]	Cellulitis; endocarditis; osteomyelitis; septic arthritis †	SA + SN	None	153 patients	Patient safety
Chambers *et al*, 2004 (New Zealand)[26]	Unspecified	SA	None	100 eligible patients	Patient acceptability
Chan *et al*, 2013 (Singapore)[27]	Abscess (liver)	Not specified	None	109 patients	Clinical effectiveness<br>Patient safety
Chapman *et al*, 2009 (UK)[28]	Bacteraemia; cellulitis; endocarditis; osteomyelitis; respiratory; SSTI †	SA + infusion centre	None	334 treatment courses	Clinical effectiveness<br>Patient safety<br>Patient acceptability
Cheong *et al*, 2008 (Australia)[29]	Abscess; cellulitis; osteomyelitis; pneumonia; wound infection	GN	None	714 treatment courses	Patient safety

For peer review only - http://bmjopen.bmj.com/site/about/guidelines.xhtml

Study (location)	Infection(s) treated	OPAT model(s)	Comparator	Population	Outcomes
Corwin *et al*, 2005 (New Zealand)[30]	Cellulitis	GN	Inpatient therapy	194 patients	Clinical effectiveness<br>Patient safety<br>Patient acceptability
Cox *et al*, 2007 (USA)[31]	Bacteraemia; cellulitis; endocarditis; osteomyelitis; respiratory †	SA	None	205 patients	Clinical effectiveness<br>Patient safety<br>Patient acceptability
Dall *et al*, 2003 (USA)[32]	Cellulitis; pneumonia	SN	Inpatient therapy	10,884 patients	Clinical effectiveness<br>Patient safety<br>Patient acceptability
Dalovisio *et al*, 2000 (USA)[33]	Cellulitis; endocarditis; osteomyelitis; respiratory †	SA + SN	None	62 patients	Clinical effectiveness<br>Patient safety
Dargan *et al*, 2007 (Canada)[34]	Cellulitis; endocarditis; osteomyelitis †	OP + home therapy (unspecified)	None	66 patients	Clinical effectiveness<br>Patient safety
Dobson *et al*, 2004 (Australia)[35]	Cellulitis; endocarditis; osteomyelitis; respiratory; septic arthritis †	SA + SN + GN	None	770 patients	Patient safety
Donald *et al*, 2005 (Australia)[36]	Cellulitis	SN	None	124 patients	Clinical effectiveness<br>Patient safety
Duncan *et al*, 2012 (UK)[37]	Bacteraemia; endocarditis; osteomyelitis; SSTI †	Not specified	None	1,377 treatment courses	Patient safety
Duncan *et al*, 2013 (UK)[38]	Endocarditis	Not specified	None	80 patients	Clinical effectiveness<br>Patient safety
Escalante *et al*, 1997 (USA)[39]	Neutropenia	Not specified	Oral therapy	83 patients	Clinical effectiveness<br>Patient safety
Esmond *et al*, 2006 (UK)[40]	Cystic fibrosis	SA	Inpatient therapy	30 patients	Clinical effectiveness<br>Patient safety
Esposito *et al*, 2004 (Multinational)[41]	Endocarditis; osteomyelitis; respiratory; SSTI †	OP + SA + SN + GN + ED + Infusion centre	None	11,427 registry patients	Clinical effectiveness
Esposito *et al*, 2007 (Italy)[42]	Discitis; osteomyelitis; prosthetic joint infection; septic arthritis	OP + SA + visit by doctor or nurse (also care facility)	None	239 patients	Clinical effectiveness<br>Patient safety<br>Patient acceptability
Esposito *et al*, 2009 (Italy)[43]	osteomyelitis; respiratory; SSTI †	OP + SA + visit by doctor or nurse (also care facility)	None	176 patients	Clinical effectiveness<br>Patient safety
Fernandez-Aviles *et al*, 2006 (Spain)[44]	Neutropenia	SN	Inpatient therapy	100 patients	Clinical effectiveness<br>Patient safety<br>Patient acceptability
Goodwin *et al*, 2002 (Canada)[45]	Cellulitis; endocarditis; osteomyelitis; respiratory; septic arthritis; SSTI †	SN + GN	None	2,405 patients	Clinical effectiveness<br>Patient safety<br>Patient acceptability
Gourdeau *et al*, 1993 (Canada)[46]	Bacteraemia; osteomyelitis; respiratory; SSTI †	OP + SA	None	124 patients	Patient safety
Graninger *et al*, 1997 (Austria)[47]	Endocarditis; osteomyelitis	OP	None	54 patients	Clinical effectiveness<br>Patient safety
Grayson *et al*, 1995 (Australia)[48]	Cellulitis; endocarditis; osteomyelitis; sepsis; vascular graft infection	GN	None	20 patients	Clinical effectiveness<br>Patient safety<br>Patient acceptability
Gross *et al*, 1996 (USA)[49]	HIV/AIDS	Home therapy (unspecified)	None	13 patients	Clinical effectiveness<br>Patient safety

For peer review only - http://bmjopen.bmj.com/site/about/guidelines.xhtml

Study (location)	Infection(s) treated	OPAT model(s)	Comparator	Population	Outcomes
Heintz *et al*, 2011 (USA)[50]	Bacteraemia; osteomyelitis; respiratory; SSTI †	OP + SA + SN + infusion centre (also long-term care facility, interim-care facility, prison)	None	494 treatment courses	Patient safety
Hindes *et al*, 1995 (USA)[51]	Bacteraemia; cellulitis; endocarditis; osteomyelitis; respiratory; septic arthritis †	SA	None	48 patients	Clinical effectiveness<br>Patient safety<br>Patient acceptability
Hitchcock *et al*, 2009 (UK)[52]	Cellulitis; endocarditis; osteomyelitis; respiratory; septic arthritis †	OP + SA + GN + private home healthcare co.	None	273 patients	Clinical effectiveness<br>Patient safety<br>Patient acceptability
Ho *et al*, 2010 (Singapore)[53]	Bacteraemia; endocarditis; osteomyelitis; SSTI	OP	None	29 patients	Clinical effectiveness<br>Patient safety
Htin *et al*, 2013 (Australia)[54]	Endocarditis	SN	None	68 patients	Clinical effectiveness<br>Patient safety
Huminer *et al*, 1999 (Israel)[55]	Endocarditis	SA + SN (also nursing home, kibbutz)	None	37 patients	Clinical effectiveness<br>Patient safety<br>Patient acceptability
Johansson *et al*, 2001 (Sweden)[56]	Leukaemia; non-Hodgkin's lymphoma	SA	None	11 patients	Clinical effectiveness<br>Patient safety<br>Patient acceptability
Kayley *et al*, 1996 (UK)[57]	Endocarditis; osteomyelitis; septic arthritis †	SA + GN + doctor administered	None	67 patients	Patient safety<br>Patient acceptability
Keller *et al*, 2013 (USA)[58]	Abscess; bacteraemia; endocarditis; osteomyelitis; septic arthritis; SSTI †	Home health agency; skilled nursing facility	Pre-infectious disease transition service	488 patients	Patient safety
Kieran *et al*, 2009 (Ireland)[59]	Endocarditis; osteomyelitis; septic arthritis; SSTI †	SA + nurses attached to commercial co.	None	56 patients	Clinical effectiveness<br>Patient safety<br>Patient acceptability
Krauth *et al*, 2003 (Worldwide)[60]	Cystic fibrosis	Not specified	Inpatient therapy (for cost comparison only)	11 studies	Clinical effectiveness<br>Cost effectiveness
Lacroix *et al*, 2014 (France)[61]	Endocarditis	Not specified	Inpatient therapy	39 patients	Patient safety
Lai *et al*, 2013 (USA)[62]	Abscess; bacteraemia; endocarditis; osteomyelitis; respiratory; SSTI †	SA + SN	None	333 patients	Clinical effectiveness<br>Patient safety
Lane *et al*, 2014 (USA)[63]	Unspecified	OP + SA	None	555 infectious disease physicians	Patient safety<br>Provider acceptability
Larioza *et al*, 2009 (USA)[64]	Endocarditis	Home infusion co.	None	43 patients	Clinical effectiveness<br>Patient safety
Larioza *et al*, 2011 (USA)[65]	Bacteraemia; endocarditis; osteomyelitis; pyelonephritis; SSTI	Home infusion co.	None	33 patients	Clinical effectiveness<br>Patient safety
Laupland *et al*, 2002 (Canada)[66]	Odontogenic; osteomyelitis; respiratory; SSTI; UTI	OP + SA + SN	None	2,405 patients	Patient safety
Lehoux, 2004 (Canada)[67]	Unspecified	SA	None	6 interviewees	Patient acceptability
Lehoux *et al*, 2006 (Canada)[68]	Unspecified	SA	None	51 nurses	Provider acceptability

Study (location)	Infection(s) treated	OPAT model(s)	Comparator	Population	Outcomes
Lillie *et al*, 2010 (UK)[69]	Cellulitis	SN	None	98 patients	Clinical effectiveness<br>Patient safety
Lin *et al*, 2005 (USA)[70]	Sinusitis	Not specified	None	177 patients	Patient safety
Lopardo, 2001 (Argentina)[71]	Endocarditis	OP + SA + SN	None	48 patients	Clinical effectiveness<br>Patient safety
Mackintosh *et al*, 2011 (UK)[72]	Osteomyelitis; septic arthritis †	OP + SA + SN	None	198 patients	Clinical effectiveness<br>Patient safety
Marra *et al*, 2005 (Canada)[73]	Bacteraemia; cellulitis; endocarditis; osteomyelitis; septic arthritis †	SA + SN	None	91 patients	Patient acceptability
Martel, 1994 (France)[74]	Cellulitis; endocarditis; osteomyelitis; septic arthritis; SSTI †	SA	None	116 patients	Patient acceptability
Martone *et al*, 2008 (USA)[75]	Bacteraemia; endocarditis; osteomyelitis; SSTI	Not specified	Inpatient therapy	949 patients	Clinical effectiveness<br>Patient safety
Matthews *et al*, 2007 (UK)[76]	Bacteraemia; endocarditis; osteomyelitis; septic arthritis; SSTI †	SA vs. GN + Community hospital staff	Professional-administered OPAT	2,134 episodes	Patient safety
Mauceri, 1994 (USA)[77]	Bursitis (septic); discitis; osteomyelitis; septic arthritis	SA	None	27 patients	Clinical effectiveness<br>Patient safety
Mazo *et al*, 2007 (Spain)[78]	Osteomyelitis; septic arthritis; SSTI †	Hospital in the Home unit	Inpatient therapy	300 patients	Patient safety
McMahon *et al*, 2008 (Australia)[79]	Endocarditis	SN	None	40 patients	Clinical effectiveness<br>Patient safety
Mohammadi *et al,* 2015 (USA)[80]	Bacteraemia; endocarditis; osteomyelitis; SSTI †	SA + SN	None	190 patients	Clinical effectiveness<br>Patient safety
Montalto & Dunt, 1997 (Australia)[81]	Cellulitis; pyelonephritis	SN (HHU)	Inpatient therapy	101 patients	Clinical effectiveness<br>Patient safety
Montalto, 1996 (Australia)[82]	Osteomyelitis; respiratory; SSTI †	SN (HHU)	None	67 patients<br>65 carers	Patient acceptability
Montalto, 1997 (Australia)[83]	Cellulitis; osteomyelitis; respiratory †	SN (HHU)	None	133 patients	Clinical effectiveness<br>Patient safety
Montalto *et al*, 2010 (Australia)[84]	Endocarditis; osteomyelitis; respiratory; SSTI †	SN (HHU)	None	3,423 treatment courses	Patient safety
Morales & von Behren, 1994 (Puerto Rico)[85]	HIV/AIDS; pneumonia; sepsis; SSTI	SA	None	22 patients	Clinical effectiveness<br>Patient safety
Muldoon *et al,* 2013 (Ireland)[86]	Endocarditis; osteomyelitis; respiratory; SSTI †	Not specified	None	55 consultant physicians	Patient safety<br>Provider acceptability
Muldoon *et al,* 2015 (USA)[87]	Endocarditis; osteomyelitis; respiratory; SSTI	Not specified	None	316 infectious disease physicians	Patient safety<br>Provider acceptability
Murray *et al*, 2005 (Canada)[88]	Cellulitis	ED	None	75 patients	Clinical effectiveness

For peer review only - http://bmjopen.bmj.com/site/about/guidelines.xhtml

Study (location)	Infection(s) treated	OPAT model(s)	Comparator	Population	Outcomes
Nathwani *et al*, 1999 (UK)[89]	Endocarditis; osteomyelitis; respiratory; septic arthritis; SSTI †	OP + SA	None	101 patients	Clinical effectiveness<br>Patient safety<br>Patient acceptability
Nathwani, 2001 (UK)[90]	SSTI	OP + SA + SN	None	125 patients	Clinical effectiveness<br>Patient safety<br>Patient acceptability
Nathwani *et al*, 2003 (UK)[91]	Osteomyelitis; septic arthritis	OP + SA	None	50 patients	Clinical effectiveness
Pajaron *et al*, 2015 (Spain)[92]	Endocarditis	SA	None	45 patients	Patient safety
Parker *et al*, 1998 (UK)[93]	Cellulitis; osteomyelitis; septic arthritis †	OP + SA	None	29 patients<br>38 GP practices	Clinical effectiveness<br>Patient safety<br>Patient acceptability<br>Provider acceptability
Partridge *et al*, 2012 (UK)[94]	Endocarditis	OP + SA	None	34 patients	Clinical effectiveness<br>Patient safety
Patanwala *et al*, 2007 (USA)[95]	Wound infection	SN	Switch (IV-oral) or oral therapy (for cost comparison only)	41 patients	Clinical effectiveness<br>Cost effectiveness<br>Patient safety
Perez-Lopez *et al*, 2008 (Spain)[96]	Abscess (abdominal); bacteraemia; cellulitis; GU infection; respiratory	SN	None	145 patients	Clinical effectiveness<br>Patient safety
Pilling & Walley, 1997 (UK)[97]	Cystic fibrosis	SA + commercial home care organisation	None	11 patients<br>14 parents	Patient acceptability
Pond *et al*, 1994 (UK)[98]	Cystic fibrosis	SA	Inpatient therapy	50 patients	Clinical effectiveness<br>Patient safety
Poretz, 1994 (USA)[99]	Bacteraemia; osteomyelitis; pneumonia; sepsis; SSTI	SA	None	238 patients	Clinical effectiveness<br>Patient safety
Poretz, 1994 (USA)[100]	SSTI	SA	None	130 patients	Clinical effectiveness<br>Patient safety
Rapoport *et al*, 1999 (Multinational)[101]	Neutropenia	OP	Inpatient therapy	88 patients	Clinical effectiveness<br>Patient safety
Rehm *et al*, 2009 (USA)[102]	Bacteraemia; endocarditis	SA + SN + (also skilled nursing facility + assisted-living + nursing home + rehab centre)	Inpatient therapy	200 patients	Clinical effectiveness<br>Patient safety
Richards *et al*, 2005 (New Zealand)[103]	Pneumonia	GN	Inpatient therapy	49 patients	Clinical effectiveness<br>Patient safety<br>Patient acceptability
Rodriguez-Cerrillo *et al*, 2010 (Spain)[104]	Diverticulitis	SN (HHU)	None	24 patients	Clinical effectiveness<br>Patient safety<br>Patient acceptability
Rodriguez-Cerrillo *et al*, 2012 (Spain)[105]	Cholecystitis	SN (HHU)	None	25 patients	Clinical effectiveness<br>Patient safety<br>Patient acceptability
Seaton & MacConnachie, 2008 (UK)[106]	Cellulitis; endocarditis; osteomyelitis; septic arthritis †	SA + other (unspecified)	None	19 patients	Clinical effectiveness<br>Patient safety
Seaton & Nathwani, 2000 (UK)[107]	Unspecified	Not specified	None	157 infection specialists	Provider acceptability

Study (location)	Infection(s) treated	OPAT model(s)	Comparator	Population	Outcomes
Seaton *et al*, 1999 (UK)[108]	Bacteraemia; osteomyelitis; respiratory; SSTI †	Not specified	None	205 eligible patients	Patient acceptability
Seaton *et al*, 2005 (UK)[109]	Cellulitis	SN	Nurse-led OPAT (vs. standard)	342 patients	Clinical effectiveness<br>Patient safety
Seaton *et al*, 2011 (UK)[110]	SSTI	OP	None	963 patients	Clinical effectiveness<br>Patient safety
Sebban *et al*, 2008 (France)[111]	Neutropenia	OP	Oral therapy	96 patients	Clinical effectiveness<br>Patient safety
Seetoh *et al*, 2013 (Singapore)[112]	Endocarditis; osteomyelitis; diabetic foot disease	OP + SA + SN	None	2,229 patients	Clinical effectiveness<br>Patient safety
Sims *et al*, 2013 (UK)[113]	Prosthetic joint infection	OP	None	14 patients	Clinical effectiveness<br>Patient safety<br>Patient acceptability
Smego *et al*, 2005 (Pakistan)[114]	Osteomyelitis; septic arthritis; SSTI †	SA	None	316 patients	Patient safety
South, 1998 (UK)[115]	Bacteraemia; endocarditis; osteomyelitis; respiratory; SSTI †	OP + SA + nurse (unspecified)	None	57 patients	Clinical effectiveness<br>Patient safety
Stein *et al*, 2008 (USA)[116]	Cellulitis	OP	Oral therapy	20 patients	Clinical effectiveness<br>Patient safety
Subedi *et al*, 2015 (Australia)[117]	Abscess; bacteraemia; endocarditis; osteomyelitis; SSTI †	SA + SN	None	144 patients	Clinical effectiveness<br>Patient safety
Talcott *et al*, 1994 (USA)[118]	Neutropenia	SA + SN	None	30 patients	Clinical effectiveness<br>Patient safety<br>Patient acceptability
Teufel *et al*, 2011 (Canada)[119]	Neutropenia	SN	Early discharge with oral therapy<br>Inpatient therapy<br>Oral outpatient therapy	77 patients	Cost effectiveness
Teufel *et al*, 2012 (Canada)[120]	Neutropenia	Not specified	None	78 participants	Patient acceptability
Theocharis *et al*, 2012 (Greece)[121]	GI tract infection; pneumonia; sepsis; SSTI; UTI	SN	None	91 patients	Clinical effectiveness<br>Patient safety
Thornton *et al*, 2005 (UK)[122]	Cystic fibrosis	Not specified	Combined therapy<br>Inpatient therapy	116 patients	Clinical effectiveness<br>Cost effectiveness
Tice, 1995 (USA)[123]	Bacteraemia; osteomyelitis; respiratory; SSTI †	OP + SA + SN	None	538 patients	Clinical effectiveness<br>Patient safety<br>Patient acceptability
Tice, 2001 (USA)[124]	Osteomyelitis	OP + SA + SN +infusion centre.	None	500 patients	Clinical effectiveness<br>Patient safety<br>Patient acceptability
Tice *et al*, 2002 (USA)[125]	Bacteraemia; endocarditis; osteomyelitis; SSTI †	OP + office based + pharmacy based	None	971 patients	Patient safety
Upton *et al*, 2004 (New Zealand)[126]	Bacteraemia; endocarditis; osteomyelitis; septic arthritis; SSTI †	SA	None	100 patients	Clinical effectiveness<br>Patient safety

For peer review only - http://bmjopen.bmj.com/site/about/guidelines.xhtml

Study (location)	Infection(s) treated	OPAT model(s)	Comparator	Population	Outcomes
Walton *et al*, 2007 (Australia)[127]	Bacteraemia; cellulitis; endocarditis; osteomyelitis; septic arthritis †	SN	None	35 patients	Clinical effectiveness<br>Patient safety
White *et al*, 2011 (Australia)[128]	Osteomyelitis; prosthetic joint infection; septic arthritis	OP + SN + GN	None	55 patients	Clinical effectiveness<br>Patient safety
White *et al*, 2013 (UK)[129]	Lyme disease	SA	None	72 patients	Clinical effectiveness<br>Patient safety
Williams, 1995 (USA)[130]	Cellulitis; osteomyelitis; septic arthritis †	SA	None	1,045 patients	Patient safety
Wolter *et al*, 1997 (Australia)[131]	Cystic fibrosis	SA	Inpatient therapy	31 admissions	Clinical effectiveness<br>Patient safety
Wolter *et al*, 2004 (Australia)[132]	Cellulitis; osteomyelitis; respiratory †	Not specified	Inpatient therapy	82 admissions	Clinical effectiveness<br>Patient safety
Wynn *et al*, 2005 (USA)[133]	Bacteraemia; endocarditis; osteomyelitis; septic arthritis; SSTI †	Physician based + home health organisation + hospital based (no details)	None	1,252 patients	Clinical effectiveness<br>Patient safety
Yadlapalli *et al*, 2002 (USA)[134]	Osteomyelitis	Not specified	None	58 patients	Clinical effectiveness<br>Patient safety
Yan *et al*, 2011 (UK)[135]	Cellulitis; osteomyelitis †	OP + GN	None	140 patients	Clinical effectiveness<br>Patient safety<br>Patient acceptability
Yang *et al*, 2014 (Canada)[136]	Abscess; endocarditis; osteomyelitis; prosthetic joint infection; vascular graft	Not specified	Previous standard care	42 patients	Clinical effectiveness<br>Patient safety
Yong *et al*, 2009 (Singapore)[137]	Cellulitis; endocarditis; osteomyelitis †	OP + SA	Inpatient therapy	162 patients	Clinical effectiveness<br>Patient safety
You *et al*, 2007 (China)[138]	Prosthetic joint infection	SA + SN	Oral therapy<br>Inpatient therapy	All patients (no numbers provided)	Cost effectiveness

ED: emergency department; GU: genitourinary; GI: gastrointestinal; GN: general nurse administered; HHU: hospital at home unit; OP: outpatient; SA: self-administered; SN: specialist nurse administered; UTI: urinary tract infection.

† For studies where ≥5 conditions were treated, only the most commonly reported across studies (included in ≥20%) have been presented.

only

For peer review only - http://bmjopen.bmj.com/site/about/guidelines.xhtml

TABLE 3 Effect of OPAT on clinical success and safety

OPAT model(s) Study ID	Inferior	Superior	No difference	Inconclusive
**Duration of treatment (all models)**			◉	
Outpatient attendance vs. inpatient treatment [101]			●	
Self-administration vs. inpatient treatment [40, 98, 131]			●	
General nurse vs. inpatient treatment [30, 103]			●	
Specialist nurse vs. inpatient treatment [32, 44, 81]			◉	
**Rate of cure or improvement (all models)**		◉		
Outpatient attendance vs. inpatient treatment [101]	●			
Self-administration vs. inpatient treatment [20]		●		
General nurse vs. inpatient treatment [30,103]			●	
Specialist nurse vs. inpatient treatment [44, 81]		◉		
**Improved lung function in CF (all models)**				○
Self-administration vs. inpatient treatment for FEV_1 [23, 40, 98, 131]			●	
Self-administration vs. inpatient treatment for FVC [23, 40, 98, 131]				○
Self-administration vs. inpatient treatment for PEFR [23, 98]				○
**Drug-related side effects (all models)**			◉	
Outpatient attendance vs. inpatient treatment [101]		●		
Self-administration vs. inpatient treatment [20, 40, 98, 131]			●	
General nurse vs. inpatient treatment [103]			●	
Specialist nurse vs. inpatient treatment [44, 78, 81, 109]				○
**Venous access complications (all models)**	◉			
Self-administration vs. inpatient treatment [20, 40, 131]			◉	
General nurse vs. inpatient treatment [103]	●			
**Hospital admission (all models)**				○
Self-administration vs. inpatient treatment [20, 131]			●	
General nurse vs. inpatient treatment [30, 103]	●			
Specialist nurse vs. inpatient treatment [32, 78, 81, 109]		◉		
**Deaths (all models)**			●	
Outpatient attendance vs. inpatient treatment [101]	●			
Self-administration vs. inpatient treatment [20, 131]			●	
General nurse vs. inpatient treatment [103]			●	
Specialist nurse vs. inpatient treatment [32, 44]			●	

(dark shading) Reports the overall effect on an outcome for all OPAT models combined. This includes findings from studies where the OPAT model was not specified or where results for multiple models were not reported separately.

(light shading) Reports the effect only for studies that specify the model under study and report on individual findings.

Key: ● found in ≥75% studies considering outcome; ◉ found in ≥50% studies considering outcome; ○ evidence of effect supported by <50% studies considering outcome.

APPENDIX 1

CIVAS SYSTEMATIC REVIEW:
DATABASES SEARCHED AND SAMPLE SEARCHES

DATABASE	DATES
British Society for Antimicrobial Chemotherapy	Accessed March 2015 http://bsac.org.uk/
CINAHL (EBSCOHost)	1981 to March 2015
The Cochrane Library (Wiley)	Accessed March 25, 2015
Embase Classic + Embase (Ovid)	1947 to March 25, 2015
Health Business Elite (HDAS, NHS Evidence)	1922 to March 25, 2015
Health Management Information Consortium – HMIC (Ovid)	1983 to March 25, 2015
International Pharmaceutical Abstracts (Ovid)	1970 to March 2015
Ovid MEDLINE(R)	1946 to March Week 4, 2015
Ovid MEDLINE(R) In-Process & Other Non-Indexed Citations	March 25, 2015
Research Papers in Economics – RePEc	Accessed March 2015 http://ideas.repec.org/
CEA Registry	Accessed March 2015 https://research.tufts-nemc.org/cear4/Default.aspx
Web of Science Conference Proceedings Citation Index – Science (Thomson Reuters)	1990 to March 2015

For peer review only

APPENDIX 1

Sample Search A: Studies of IV antibiotics and known models of care

Ovid MEDLINE(R) <1946 to March Week 4, 2015>

----

1 exp Anti-Bacterial Agents/ (497300)

2 Anti-Infective Agents/ (36221)

3 exp Anti-infective agents, Urinary/ (28162)

4 exp antifungal agents/ (126501)

5 or/1-4 (612854)

6 Administration, Intravenous/ (151)

7 infusions, intravenous/ (45941)

8 injections, intravenous/ (75646)

9 Home infusion therapy/ (579)

10 or/6-9 (120138)

11 ((parenteral or intravenous or IV or inject* or infusion*) adj5 (antibiotic* or anti-biotic* or antimicrobial* or anti-microbial* or antifungal* or anti-fungal* or anti-infective* or antiinfective* or antibiotherap*or anti-biotherap*)).tw. (10477)

12 (5 and 10) or 11 (20711)

13 exp Ambulatory Care Facilities/ (41613)

14 exp Delivery of healthcare/ (739029)

15 Critical Pathways/ (4202)

16 Ambulatory Care/ (34170)

17 Emergency Service, Hospital/ (39312)

18 outpatients/ (7955)

19 inpatients/ (11488)

20 day care/ (4526)

21 inpatients/ (11488)

22 hospitalization/ (66091)

23 community health services/ (25779)

24 community health nursing/ (17982)

25 home care services/ (26544)

26 home care services, hospital-based/ (1552)

27 home nursing/ (7802)

28 home infusion therapy/ (579)

29 or/13-28 (936219)

30 12 and 29 (1550)

31 ("emergency ward*" or "emergency room*" or "accident and emergency" or ED or "emergency department*" or A&E).tw. (77291)

32 outpatient*.tw. (95608)

For peer review only

APPENDIX 1

33 (home or homes).tw. (141950)

34 (self adj3 (treat* or care or regime*)).tw. (14552)

35 ((clinic or clinics) adj3 (treat* or care or regime*)).tw. (11443)

36 (community adj3 (treat* or care or regime*)).tw. (15994)

37 (ambulatory adj3 (treat* or care or regime*)).tw. (11257)

38 "district nurs*".tw. (1351)

39 ("specialist nurs*" or "nurse specialist*").tw. (3498)

40 "community nurs*".tw. (2437)

41 ((hospital* or ward or clinic) adj2 (patient or patients)).tw. (58637)

42 inpatient*.tw. (54156)

43 ("care pathway*" or "care model*" or "model* of care").tw. (6275)

44 or/31-43 (438938)

45 44 and 12 (2102)

46 "outpatient parenteral antibiotic* therapy".tw. (103)

47 "outpatient antibiotic* therapy".tw. (64)

48 (ohpat or opat).tw. (116)

49 or/45-48 (2140)

50 49 or 30 (2678)

Sample Search B: Reviews of IV antibiotic use in cellulitis or cystic fibrosis

Ovid MEDLINE(R) <1946 to March Week 4, 2015>

----

1 exp *Anti-Bacterial Agents/ or *Anti-Infective Agents/ or exp *antifungal agents/ or exp *Anti-infective agents, Urinary/ (380181)

2 *Administration, Intravenous/ or *infusions, intravenous/ or *injections, intravenous/ or *Home infusion therapy/ (5005)

3 1 and 2 (324)

4 ((parenteral or intravenous or IV or inject* or infusion*) adj5 (antibiotic* or anti-biotic* or antimicrobial* or anti-microbial* or antifungal* or anti-fungal* or anti-infective* or antiinfective* or antibiotherap*or anti-biotherap*)).ti. (1464)

5 3 or 4 (1649)

6 exp Anti-Bacterial Agents/ (498780)

7 Anti-Infective Agents/ (36330)

8 exp Anti-infective agents, Urinary/ (28211)

9 exp antifungal agents/ (126949)

10 or/6-9 (614703)

11 Administration, Intravenous/ (177)

12 infusions, intravenous/ (46068)

For peer review only

APPENDIX 1

13 injections, intravenous/ (75786)

14 Home infusion therapy/ (580)

15 or/11-14 (120429)

16 ((parenteral or intravenous or IV or inject* or infusion*) adj5 (antibiotic* or anti-biotic* or antimicrobial* or anti-microbial* or antifungal* or anti-fungal* or anti-infective* or antiinfective* or antibiotherap*or anti-biotherap*)).tw. (10513)

17 (10 and 15) or 16 (20758)

18 "outpatient* parenteral".tw. [finds more] (189)

19 "outpatient parenteral antibiotic* therapy".tw. (103)

20 "outpatient antibiotic* therapy".tw. (64)

21 (ohpat or opat).tw. (116)

22 Cellulitis/ (6046)

23 cellulitis.tw. (5439)

24 Cystic Fibrosis/ (27005)

25 (cystic adj fibrosis).tw. (29491)

26 "fibrocystic disease*".tw. (807)

27 17 or 19 or 20 or 21 (20796)

28 or/22-26 (43980)

29 (5 or (18 or 19 or 20 or 21)) and 28 [major headings or title occurence of IV Antibiotic terms AND MINOR Cell/CF terms] (125)

30 17 and 28 [most sensitive search] (1037)

31 limit 30 to "review articles" (150)

32 29 or 31 (254)

For peer review only

APPENDIX 2

CIVAS SYSTEMATIC REVIEW:
QUALITY ASSESSMENT – OBSERVATIONAL STUDIES

**Population** (tick one box) **PAPER ID:**		
1. Sample size:		
a) Strong – power calculation provided	✪	☐
b) Strong – included all OPAT patients	✪	☐
c) Strong – appropriate size for qualitative study	✪	☐
d) Insufficient – no power calculation or selective/small study		☐
e) Unclear – not enough information provided		☐
**Methodology:** (tick one box)		
2. Method to ascertain data:		
a) Strong – not open to selection bias*	✪	☐
b) Strong – appropriate qualitative methodology	✪	☐
c) Moderate – open to some bias but appropriate method used	○	☐
d) Insufficient – inappropriate method used to ascertain data		☐
e) Unclear – not enough information provided		☐
**Analysis:** (tick one box)		
3. Data analysis:		
a) Strong – reports statistically significant differences in outcomes	✪	☐
b) Strong – relevant qualitative analytic techniques	✪	☐
c) Moderate – used only comparative analysis with no statistical testing of differences	○	☐
d) Moderate – reported only descriptive statistics	○	☐

* Systematic differences between comparison groups in terms of selection, prognosis or treatment; differences in group characteristics.

ASSESSMENT OF THE RISK OF BIAS:

**Potential bias:**	Selection		Analysis	
	Method		**ASSESSMENT**	

SUPPLEMENTARY TABLES

TABLE S1: Clinical effectiveness of outpatient parenteral antibiotic treatment

ID	Author (country)	Study design	OPAT Model(s)	OPAT group(s)†	Comparator(s)†	Findings
**COMPARATOR STUDIES**						
20	Bedi et al (UK)	Cohort	SA	Home therapy (domiciliary IV); 52 patients (median age 61); 58% male<br>Home therapy (early supported discharge); 23 patients (median age 65); 35% male	Inpatient therapy; 36 patients (median age 71); 36% male	There was resolution of infection in 76% of the inpatient group, 80% of the ESD group and 80% of the domiciliary IV group.<br>Within group comparisons showed significant (p<0.05) improvements in FEV1, FVC, incremental shuttle walking test, 24-h sputum volume, sputum bacterial clearance, and parameters of inflammation in all groups.
23	Bradley et al (UK)	Observational (retrospective)	SA	Home therapy; 8 patients (mean age 26); 50% male	Combined therapy; 14 patients (mean age 24); 57% male<br>Inpatient therapy; 29 patients (mean age 24); 83% male	On completion of therapy, there was no significant difference between the groups in FEV_1 (51 vs. 66 vs. 60), FVC (63 vs. 73 vs. 71) or $FEF_{25\text{-}75}$ (32 vs. 43 vs. 29).<br>PEFR was significantly greater in the OPAT and Combined groups than in the Hospital group (84 vs. 73 vs. 67; p<0.05).<br>When change was expressed as a percentage of the baseline value, $FEF_{25\text{-}75}$ showed a large improvement in each group (approx. 15-48%). There was a significantly greater improvement in FVC, FEV_1 and $FEF^{25\text{-}75}$ in the Hospital group than in the other two groups.
30	Corwin et al (New Zealand)	RCT	GN	Home therapy; 98 patients (mean age 55); 62% male	Inpatient therapy; 96 patients (mean age 48); 73% male	There was no significant difference in mean days to no advancement of cellulitis between Home and Hospital groups (1.50 days vs. 1.49 days; 95% CI -0.3 to 0.28)).<br>This was also the case for days on IV antibiotics (HR 0.84, 95% CI 0.63-1.12; p=0.23), and days to discharge (HR 0.93, 95% CI 0.70-1.23; p=0.60).
32	Dall et al (USA)	Before and after	SN	Home therapy; 92 pneumonia patients (97% aged >50 years); 44% male / 64 cellulitis patients (mean age 51); 70% male	Inpatient therapy; 10728 pneumonia patients (83% aged >50 years); 51% male / cellulitis patients (no numbers provided)	Most OPAT cellulitis patients (45/64; 70%) required only two day's treatment (range 2-6d). Among pneumonia patients, 58% (53/92) required ≤3 days of treatment (range 2-9d).<br>Median length of stay was shorter for OPAT pneumonia patients than for historical comparators (0.07-1.07 days vs. 2-4 days vs. 6-9 days; p<0.05). This was also the case for SSTI patients ((0.1 day vs. 1.3 days; p<.001).
39	Escalante et al (USA)	Clinical trial (unspecified)	Not specified	IV therapy; 43 patients (median age 39); 42% male	Oral therapy; 40 patients (median age 52); 50% male	There was a response in 41/43 (95%) patients receiving IV therapy compared with 35/40 (88%) patients receiving oral treatment (p=0.19).<br>Preliminary results of a second trial (altered oral regime) showed a response rate of 87% (78/90 patients) in the IV arm and 90% (80/89 patients) in the oral arm.

SUPPLEMENTARY TABLES

ID	Author (country)	Study design	OPAT Model(s)	OPAT group(s)†	Comparator(s)†	Findings
40	Esmond et al (UK)	Controlled trial	SA	Home therapy; 15 patients (mean age 27); 40% male	Inpatient therapy; 15 patients (mean age 23); 60% male	There was no significant difference between groups in mean duration of treatment: Home group 14 days (range 10-18d), Hospital group 15 days (range 10-25d).<br>Improved lung function was significantly greater in the Hospital group than in the Home group (FEV_1 2.0 vs. 5.2; p=0.05 and FVC 2.1 vs. 12.3; p=0.01). When change in the groups was directly compared, only the difference in FVC was significant (p=0.01).
44	Fernandez-Aviles et al (Spain)	Case-control	SN	Home therapy; 50 patients (mean age 47); 62% male	Inpatient therapy; 50 patients (median age 50); 54% male	There was no significant difference between Home and Hospital patients in relation to median duration of treatment (8 days vs. 9 days).<br>Median duration of fever was significantly shorter in Home patients (2 days vs. 5 days; p=0.00003).
75	Martone et al (USA)	Observational (retrospective)	Not specified	OPAT; 539 patients (57% aged >50 years); 56% male	Inpatient therapy; 410 patients (63% aged >50 years); 45% male	Median duration of OPAT only treatment was 17 days (range 3-144d). Median duration of hospital treatment was 7 days (range 1-153d).<br>Success rates were higher in OPAT (510/539) than in hospital (354/41) patients (94.6% vs. 86.3%; p<0.001).
81	Montalto & Dunt (Australia)	Observational (retrospective)	SN (HHU)	HHU therapy; 55 cellulitis patients (mean age 52); 60% male / 14 pyelonephritis patients (mean age 30); 0% male	Inpatient therapy; 22 cellulitis patients (mean age 51); 73% male / 10 pyelonephritis patients (mean age 35); 30% male	Mean duration of stay was lower for HHU cellulitis patients (6.00 days vs. 8.55 days; 95% CI 0.24-4.85) but not for pyelonephritis patients (4.57 days vs. 4.00 days; 95% CI -1.86-0.72).<br>Full recovery was expected in 51/55 HHU cellulitis patients (93%); the remaining four showed recovery back to a stable pre-existent condition. Recovery was expected in all 22 Hospital patients.<br>Recovery was expected in 13/14 HHU pyelonephritis patients (93%), and in 9/10 Hospital patients (90%). The remaining patient in each group was expected to return to a stable pre-existent condition.<br>Mean time to febrifuge was lower for HHU cellulitis (1.96 days vs. 2.00 days) and pyelonephritis patients (1.79 days vs. 2.40 days), although the difference was not statistically significant.
98	Pond et al (UK)	Case-control (retrospective)	SA	Home therapy; 25 patients (mean age 21); 56% male	Inpatient therapy; 25 patients (mean age 22); 56% male	Mean duration of treatment was similar in the two groups (OPAT 14.1 days vs. Hospital 16.7 days).<br>There was no difference between the OPAT and Hospital groups in relation to adjusted mean improvement in FVC (0.567 vs. 0.644), FEV1 (0.456 vs. 0.403), PEFR (66.1 vs. 57.5) or other outcome variables. The only variable to show a significant difference was total white cell count (-3.64 vs. -4.72; p<0.05).

SUPPLEMENTARY TABLES

ID	Author (country)	Study design	OPAT Model(s)	OPAT group(s)†	Comparator(s)†	Findings
101	Rapoport et al (Multinational)	RCT	OP	Outpatient therapy; 40 patients (median age 45); 52% male	Inpatient therapy; 44 patients (median age 48); 32% male	Median duration of treatment was similar for both OPAT and inpatients groups (6.0 days vs. 6.3 days).<br>Treatment was successful in 34/38 OPAT patients (89%) and 40/42 inpatients (95%).
102	Rehm et al (USA)	RCT	SA; SN; (also skilled nursing facility; assisted-living; nursing home; rehab centre)	Home therapy; 103 patients (median age 50); 58% male	Inpatient therapy; 97 patients (median age 54); 61% male	OPAT patients received longer courses of antibiotics than hospital patients (mean 25.4 days vs. 13.5 days; p<0.001). OPAT patients received a mean of 14.9 days of therapy outside the hospital setting.<br>There was a higher rate of clinical success among OPAT patients (89/103; 86.4%) than hospital patients (54/97; 55.7%) (p<0.001).
103	Richards et al (New Zealand)	RCT	GN	Home therapy; 24 patients (mean age 50); 54% male	Inpatient therapy; 25 patients (mean age 50); 52% male	Median time to discharge was 4 days (range 1-14d) in the Home group and 2 days (range 0-10d) in the Hospital group (p=0.004).<br>There was no difference in the number of days on IV antibiotics (3 days vs. 2 days; p=0.22).<br>At 2 weeks, there was no difference in patient-rated symptoms. There was a significant difference in sleep disturbance (Home median "occasional"; Hospital median "never"; p<0.01), but this did not persist at 6 weeks. There was no difference in time to resolution of fever, tachycardia or tachypnoea.
109	Seaton et al (UK)	Before and after	SN	Standard OPAT; 230 patients (median age 49); 52% male	Nurse-led OPAT; 112 patients (median age 50); 61% male	Total median duration of IV therapy was reduced from 5 days (range 1-37d) to 4 days (range 1-23d) (p=0.01)<br>Median duration of outpatient therapy was reduced from 4 days (range 1-37d) to 3 days (range 1-22d) (p=0.02).<br>Cure or improvement was similar for pre (225/228) and post-protocol (106/108) patients (99% vs. 97%).
111	Sebban et al (France)	RCT	OP	IV therapy; 47 patients (mean age 52); 40% male	Oral therapy; 49 patients (mean age 52); 31% male	Success was observed for 34/46 (73.9%) patients in the IV group and 38/48 (79.2%) patients in the Oral group (calculated risk difference was 5.3%; 95% CI 11.9 to 22.4%).
116	Stein et al (USA)	RCT	OP	IV therapy; 10 patients (mean age 54); 60% male	Oral therapy; 10 patients (mean age 53); 40% male	Average number of OPAT doses was eight (range 1-30). Average number of Oral doses was 19 (range 9-40).<br>Cure and improvement rates were higher for Oral group patients (10/10; 100%) than for OPAT patients (6/10; 60%), although this was not statistically significant (p=0.09).
122	Thornton et al (UK)	Observational (retrospective)	Not specified	Home therapy; 47 patients (mean age 26); 36% male	Combined therapy; 18 patients (mean age 25); 61% male<br>Inpatient therapy; 51 patients (mean age 26); 59% male	Mean treatment duration was 63 days for Home patients 54 days for Hospital patients and 66 days for Combined patients.<br>Mean FEV_1 improvement from baseline ''best'' was significantly higher for Hospital patients (mean diff. 4.6%, 95% CI 1.8-7.4; p=0.001).

SUPPLEMENTARY TABLES

ID	Author (country)	Study design	OPAT Model(s)	OPAT group(s)†	Comparator(s)†	Findings
						More Hospital (41/236; 17%) than Home courses (18/199; 9%) were classified as effective (p=0.001). At the ≤0% level, there was a mean decline in FEV_1 for Home patients after 1 year, but an improvement for Hospital patients (mean diff. 10.1%, 95% CI 2.9-17.2; p=0.003). The number of patients in whom treatment was classified as effective was higher in the Hospital (n=30; 59%) than Home group (n=20; 43%). When effectiveness was defined as an FEV_1 decline of ≤2%, the difference became statistically significant (43% vs. 63%; p=0.045).
131	Wolter et al (Australia)	RCT	SA	Home care; 13 admissions (median age 22); 28% male	Inpatient therapy; 18 admissions (median age 22); 39% male	There were no significant differences in the duration of treatment or use of antibiotics. Median duration of treatment was 12 days (range 10-24d) in the Home group and 11 days (range 7-26d) in the Hospital group (p=0.2). Home patients had fewer investigations performed than inpatients (p=0.002). There was no significant difference between groups in improved lung function (FEV_1 p=0.27; FVC p=0.30). There was no significant difference in time to next admission between the groups (p=0.68).
132	Wolter et al (Australia)	RCT	Not specified	Home care; 44 admissions (median patient age 43); 45% male	Inpatient therapy; 38 admissions (median patient age 49); 34% male	Median duration of treatment was similar between the groups (Home 11.5 days, range 3-57d; Hospital 11.0 days, range 4-126d; p=0.002). Median number of investigations was significantly lower in the Home group (12.5 vs. 19.0; p<0.001). There was no difference in numbers with an "Improved" outcome following treatment (Home 84.1% vs. Hospital 92.1%; p=0.32). Average time to the next admission was longer in the Hospital group (12 days vs. 32 days; p=0.006).
136	Yang et al (Canada)	Case-control	Not specified	OPAT; 21 patients (mean age 59); 57% male	Previous standard care; 21 patients (mean age 59); 57% male	There was a better overall cure rate in the OPAT group (61.9% vs. 57.1%, p=0.1), For a composite end point of clinical success (i.e. combined cures and controlled/improved cases) the results were 95% in the OPAT group and 86% in the previous care group (p=0.62). There were three (14.3%) treatment failures in the control group and one (4.8%) in the OPAT group
137	Yong et al (Singapore)	Case-control (retrospective)	OP; SA	OPAT ; 69 patients (mean age 53); 53% male	Inpatient therapy; 93 patients (mean age 56); 51% male	Average length of OPAT only care was 24.3 days (range 4-93d). Mean duration of treatment for OPAT patients was 42.5 days (range 4-145d), significantly longer than for hospital patients (mean 19 days; range 4-183d) (p<0.001). At the end of the 4-month follow-up period, 59/72 OPAT

SUPPLEMENTARY TABLES

ID	Author (country)	Study design	OPAT Model(s)	OPAT group(s)†	Comparator(s)†	Findings
						episodes (81.9%) were considered cured compared with 75/93 hospital patients (80.6%).
**OPAT ONLY STUDIES**						
11	Al Ansari et al (Bahrain)	Case-series (retrospective)	OP	101 patients; 57% male	—	Average duration of treatment ranged from three to 10 days.<br>Total cure was achieved in 97 (96%) treated patients. Two patients were lost to follow-up with the OPAT clinic.
14	Anand et al (USA)	Case-series	Home IV therapy (unspecified)	52 patients	—	Patients showed a significant improvement in all symptoms post-treatment (p<0.001).
15	Angel (USA)	RCT	SA	62 patients (mean age 71); 53% male	—	Mean length of inpatient treatment was 3.6 days less than expected from DRGs.<br>Documented cure was seen in 35/50 evaluable patients (70%), while 14 (28%) had improved. There was one clinical failure.<br>Bacteriologic eradication was seen in 16/28 (57%) evaluable patients; 10 others (36%) were clinically improved (no culture could be obtained). Two patients had persistent pathogens.
18	Barr et al (UK)	Observational (retrospective)	OP; SA; SN; GN	2233 patients (median age 51); 58% male	—	Median duration of treatment was 5 days (range <1-328d).<br>Most patients were cured (1,501/2233; 67%) or improved (n=562; 25%). Small numbers showed no change (n=52; 2%) or deteriorated (n=91; 4%).
21	Berman & Johnson (USA)	Case-series (retrospective)	SA	221 patients (median age 41); 66% male	—	Median duration of treatment was 18 days (range 3-307d).<br>OPAT was successful in 283/302 episodes (94%). Treatment failed in 19 episodes (6%) of which 16 (84%) required hospitalisation.
22	Bernard et al (France)	Observational (?)	SA; SN;	39 patients (mean age 44); 64% male	—	Mean duration of treatment was four months (range 1.5 to 12m).<br>Twenty-eight of 30 patients (93%) available for follow-up were considered cured with a mean delay of 24 months after completion of therapy. There was one clinical failure and one relapse within one month after treatment.
27	Chan et al (Singapore)	Observational	Not specified	109 patients (mean age 57); 66% male	—	Average duration of IV therapy was 32 days (range 11-98d). Mean length of OPAT was 16 days (range 2-54d).<br>Eighty of 109 patients (74%) achieved a clinical response at four weeks. All patients achieved cure.

SUPPLEMENTARY TABLES

ID	Author (country)	Study design	OPAT Model(s)	OPAT group(s)†	Comparator(s)†	Findings
28	Chapman et al (UK)	Observational (retrospective)	SA; Infusion centre	334 treatment courses (mean patient age 46); 59% male	—	Mean duration of treatment for SSTI (n=198) was 4.9 days (range 1-46d). Mean duration of treatment for other infections (n=136) was 22.5 days (range 1-163d).<br>A total of 291/334 patients (87%) were cured or improved on completion of therapy; 11 were recorded as "no change".<br>Sixty two records of SSTI patients were selected at random for follow-up data. Mean duration of follow-up was 27.8 days (range 4-122d); 61 (98.4%) were cured or improved.
31	Cox et al (USA)	Observational (retrospective)	SA	205 patients (mean age 59); 99% male	—	Median duration of treatment was 22 days (range 2-105d) for older adults (≥60 years) and 29.5 days (range 4-450d) for younger adults.<br>A total of 54/231 (23%) courses resulted in cure at the end of home IV treatment, while 159 courses (69%) were deemed to have stable or improved infection. In 16 courses (7%) treatment was considered a failure (in all cases attributed to a difficult-to-treat infection rather than adverse effects).
33	Dalovisio et al (USA)	Observational (retrospective)	SA; SN	62 patients (mean age 69)	—	Average duration of treatment was 23.4 days.<br>Average number of home care visits per course was 18.8.<br>The majority of treatment courses (60/66; 91%) met the goals of therapy.
34	Dargan et al (Canada)	Case-series (retrospective)	OP; Home therapy (unspecified)	66 patients (mean age 57); 50% male	—	Mean total duration of therapy was 32.8 days (range 3-169d). Mean duration of OPAT treatment was 27.3 days (range 2-169d).<br>Most patients (56/66; 85%) had a successful outcome; 10 patients experienced treatment failure.
36	Donald et al (Australia)	Case-series (retrospective)	SN	124 patients (aged 16-97); 60% male	—	Mean duration of treatment was 6.24 days.<br>The majority of patients were treated successfully (105/124; 85%).
38	Duncan et al (UK)	Observational (retrospective)	Not specified	55 completed OPAT patients (median age 59); 80% male / 25 failed OPAT patients (median age 67); 60% male	—	Median duration of OPAT was 28 days (IQR 20-38 days); all patients had a prior period of inpatient management (median 22 days, IQR 14–30 days).<br>OPAT failure occurred in 25/80 (31.3%) episodes. Increased probability of failure was associated with co-existing cardiac or renal failure (OR 7.29, 95% CI 1.84-29.66, p=0.005), specialist referral (OR 0.25, 95% CI 2.01-37.47, p=0.00), and use of Teicoplanin (OR 8.69, 95% CI 0.06-1.11, p=0.068).
41	Esposito et al (Multinational)	Observational (retrospective)	OP; SA; SN; GN; Emergency room; Infusion centre	9826 US registry patients (aged 1-80+); 57% male / 981 UK registry patients (aged 11-80+); 63% male / 620 Italian registry	—	Mean duration of treatment was longer in Italy (56.0 days) compared with the USA (22.5 days) and UK (19.9 days).<br>Most patients (10629/11427; 93%) achieved clinical cure or improvement (USA 93%; UK 97%; Italy 95%).

SUPPLEMENTARY TABLES

ID	Author (country)	Study design	OPAT Model(s)	OPAT group(s)†	Comparator(s)†	Findings
				patients (aged 1-80+); 57% male		
42	Esposito et al (Italy)	Observational (retrospective)	OP; SA; Visit by doctor or nurse (also care facility)	239 patients (aged 11-80+); 62% male	—	Mean duration of treatment was 71.2 days (SD 39.3d).<br>Clinical results were satisfactory, with only 20 failures observed at the end of therapy (8.4%).<br>Of 120 patients available for follow-up (30 days after end of therapy), 72 (60%) were cured and 35 (29%) were improved.
43	Esposito et al (Italy)	Observational	OP; SA; Visit by doctor or nurse (also care facility)	176 patients (aged >65 years); 47% male	—	At the end of therapy, almost 90% of cases were as cured or improved (156/176).<br>Seventy one of 176 patients were available for follow-up (30 days after the end of therapy); cure or improvement was recorded in 81% (56/71).
45	Goodwin et al (Canada)	Case-series	SN; GN	2405 patients (mean age 46); 55% male	—	Duration of treatment ranged from one to >42 days (longest was 209 days).<br>Five patients had a resolution of their infection earlier than expected
47	Graninger et al (Austria)	Case-series	OP	54 patients	—	Mean duration of treatment for osteomyelitis was 62 days (range 28-150d).<br>Cure was achieved in 18 (41%) and improvement in 19 (43%) of 44 patients. Seven patients failed to respond even after the minimum treatment period of 1 month (range 25-84d).<br>Average duration of treatment for endocarditis was 49 days (range 28-88d).<br>Eight of 10 patients were cured (80%), and there were two treatment failures.
48	Grayson et al (Australia)	Case-series	GN	20 patients (mean age 58)	—	Mean duration of treatment was 26 days (range 11-44d).<br>Eighteen of 20 patients (90%) were clinically and microbiologically cured after therapy.
49	Gross et al (USA)	Case-series (retrospective)	Home IV therapy (unspecified)	13 patients (mean age 38); 100% male	—	There were no treatment failures.
51	Hindes et al (USA)	Case-series	SA	48 patients (mean age 65)	—	Mean duration of therapy was 22 days.<br>All patients (48/48) had eradication or arrest of their infection.

SUPPLEMENTARY TABLES

ID	Author (country)	Study design	OPAT Model(s)	OPAT group(s)†	Comparator(s)†	Findings
52	Hitchcock et al (UK)	Case-series	OP; SA; GN; Private home healthcare company	273 patients (mean age 60); 54% male	—	Mean length of treatment was 24 days (range 1-165d). In 278/303 episodes (92%), patients were successfully treated as per plan.
53	Ho et al (Singapore)	Case-series	OP	29 patients (median age 41); 90% male	—	Median length of hospital stay prior to OPAT was 15 days (range 2-48d) Median duration of treatment was 18 days (range 1-85d). All but one patient (28/29; 96.6%) completed the intended duration of OPAT therapy.
54	Htin et al (Australia)	Case-series (retrospective)	SN	68 patients (median age 68); 87% male	—	Median duration of antimicrobial therapy was 24 days (range 4 to 42 days). Treatment success rate was 94% (64/68). There were two relapses.
55	Huminer et al (Israel)	Case-series	SA; SN (also nursing home; kibbutz)	37 patients (mean age 64); 57% male	—	Mean duration of treatment was 26.2 days (range 14-49d). Cure after a first course of treatment was documented in 34/37 patients (92%).
56	Johansson et al (Sweden)	Case-series	SA	11 patients (mean age 51); 73% male	—	Median duration of OPAT was 4 days (range 1-12 days). Patients received therapy during 16 episodes of neutropenic fever. None of the patients developed recurrent fever.
59	Kieran et al (Ireland)	Case-series (retrospective)	SA; Nurses attached to commercial company	56 patients (median age 50); 57% male	—	Median duration of treatment was 16 days (range 2-84d). Clinical cure was documented in 52/56 patients (93%). There was no significant difference in outcomes between self-administered courses (n=48) and those given by a nurse (n=12); (p=0.61).
60	Krauth et al (Worldwide)	Literature review	Not specified	11 studies	—	Only one of seven studies evaluating efficacy of home IV therapy for CF found significant differences between home and inpatient care with regard to short-term outcomes such as VC% and FEV_1% etc. The only randomised study supported the (short-term) equivalence of home care and inpatient therapy. One trial performed under everyday conditions found that home care therapy produced significantly poorer VC and FEV_1 results.
62	Lai et al (USA)	Case-series (retrospective)	SA; SN	333 patients (mean age 62); 98% male	—	Mean duration of treatment was 21.1 days (range, 0-88 days; interquartile ratio, 9-30 days). Overall completion rate was 79.6% (313/393 courses). Factors associated with non-completion were bacteraemia (OR 1.82; p=0.040), concomitant CHF (OR 1.64; p=0.051), and concomitant end stage renal disease (ESRD) (OR 2.59; p=0.015). Only ESRD remained significant when the three variables were combined in a multivariable model (OR 2.20;

SUPPLEMENTARY TABLES

ID	Author (country)	Study design	OPAT Model(s)	OPAT group(s)†	Comparator(s)†	Findings
						p=0.047).
64	Larioza et al (USA)	Case-series (retrospective)	Home infusion company	43 patients (56% aged >50 years); 67% male	—	Patients were treated for 4-8 weeks; duration of OPAT was 70% for patients with surgery and 80% for patients without (p=0.0561).<br>All patients survived for one year following therapy without infective endocarditis relapse (43/43).
65	Larioza et al (USA)	Case-series (retrospective)	Home infusion company	33 patients (76% aged ≥50 years); 61% male	—	Mean duration of treatment was 29 days.<br>Thirty one of 33 patients (94%) were successfully treated.
69	Lillie et al (UK)	Case-series	SN	98 patients (mean age 55); 61% male	—	Average duration of treatment was 6.3 days.<br>Elevated baseline CRP (p=0.002), male sex (p=0.004), prolonged duration of symptoms prior to OPAT (p=0.001) and higher antibiotic dose (p=0.006) were associated with prolonged treatment via OPAT.
71	Lopardo (Argentina)	Case-series (retrospective)	OP; SA; SN	48 patients (median age 55); 62% male	—	Clinical and microbiologic cure was achieved in 100% of cases (48/48).
72	Mackintosh et al (UK)	Case-series (retrospective)	OP; SA; SN	198 patients (aged <40-89); 64% male	—	Success rate following initial OPAT was 86.4% (171/198), ranging from 71.8% for diabetic foot or stump infection to 100% for metalwork-related infection.<br>Median duration of follow-up was 60 weeks (range 6-104w). A total of 59/198 patients (29.8%) failed initial treatment, re-presented with a recurrence or relapse of their infection, or died during the follow-up period. Of those who failed, 55 (93%) did so within 12 months.
77	Mauceri (USA)	RCT	SA	27 patients	—	Mean duration of OPAT treatment was 30.50 days. Mean duration of prior hospitalisation was 12.17 days (1.77 days longer than allotted by DRGs).<br>Cure was achieved in 11/18 evaluable patients (61%) and improvement in four patients (22%).<br>Bacteriologic success rate was 79% (11/14 patients).
79	McMahon et al (Australia)	Observational	SN	40 patients (mean age 59 (M), 49 (F)); 75% male	—	Mean duration of treatment was 47.7 days, with the mean inpatient treatment duration being 23.5 days (range 8-75d), and the mean HITH treatment duration being 24.2 days (range 2-63d).<br>Cure was achieved in 37/40 (93%) patients, with the three remaining patients considered as failures.

SUPPLEMENTARY TABLES

ID	Author (country)	Study design	OPAT Model(s)	OPAT group(s)†	Comparator(s)†	Findings
80	Mohammadi et al (USA)	Case-series (retrospective)	SA; SN	190 patients (mean age 63); 98% male	—	Median duration of OPAT was 30 days (range 5-56 days).<br>The overall cure rate for all infections treated was 78% at end of treatment, decreasing to 58% at 90 days post-OPAT (p<0.001).
83	Montalto (Australia)	Case-series (retrospective)	SN (HHU)	133 patients (mean age 46); 52% male	—	Mean duration of HHU stay was 4.8 days (range 1-28d).<br>Full recovery was expected in 132/133 patients (99%).
85	Morales & von Behren (Puerto Rico)	Case-series	SA	22 patients (mean age 33-41); 82% male	—	Mean length of inpatient treatment was 4.5-6.5 days shorter than allotted by DRGs.<br>On completion of treatment, 19/20 evaluable patients (95%) were cured or improved.<br>Bacteriologic eradication was seen in 15/17 (88%) evaluable patients. There were no super infections or reinfections.
88	Murray et al (Canada)	Observational	Emergency department	75 patients (mean age 48); 57% male	—	Two of 29 patients in the oral group failed treatment (6.8%), compared with 12/46 patients in the IV group (26%).
89	Nathwani et al (UK)	Case-series	OP; SA	101 patients	—	Most patients were cured or improved (95/101; 94%), while two patients were unchanged (4%), and the remaining four were worse (4%).
90	Nathwani (UK)	Case-series	OP; SA; SN	125 patients	—	Mean duration of treatment was 5.32 days.<br>The majority of patients (123/125; 98%) were cured or improved. Two patients were worse and required surgery.
91	Nathwani et al (UK)	Observational (retrospective)	OP; SA	50 patients	—	Mean duration treatment was 37.7 days.<br>All patients 50/50 were deemed improved or cured at the end of treatment.
93	Parker et al (UK)	Observational	OP; SA	29 patients; 38 GP practices	—	Of the 29 enrolled patients, 23 (79%) had complete resolution of their symptoms. The remaining six were indeterminate.
94	Partridge et al (UK)	Case-series (retrospective)	OP; SA	34 patients (mean age 55); 79% male	—	Median duration of treatment was 27 days (range 7-65d).<br>Most episodes (34/36; 94%) were treated successfully, with no evidence of recurrence at a median of 30 months following completion of OPAT (range 6-57m).

For peer review only - http://bmjopen.bmj.com/site/about/guidelines.xhtml

SUPPLEMENTARY TABLES

ID	Author (country)	Study design	OPAT Model(s)	OPAT group(s)†	Comparator(s)†	Findings
95	Patanwala et al (USA)	Decision tree analysis	SN	41 patients (mean age 53); 44% male	—	Cure was documented in 18/41 patients (44%) and presumed in 11/41 patients (27%). Twelve patients were treatment failures.
96	Perez-Lopez et al (Spain)	Observational	SN	145 patients (mean age 68); 48% male	—	Mean duration of treatment was 8.8 days (range 2-60d).<br>Definitive discharge was given to 83/90 (92%) elderly group patients (≥70 years) due to satisfactory clinical evolution (no data reported for younger patients).
99	Poretz (USA)	RCT	SA	238 patients (mean age 45-52); 57% male†	—	Of 211 evaluable patients, 158 (75%) were cured and 43 (20%) were improvement. Response was unsatisfactory in the remaining 10 patients (5%).<br>Bacteriologic eradication was seen in 125/134 (93%) evaluable patients. There was persistence in 8 patients (6%).
100	Poretz (USA)	RCT	SA	130 patients (mean age 45); 59% male†	—	For patients with initial inpatient treatment, length of stay was 2.4 days shorter than allotted by DRGs. Mean duration of treatment completed entirely at home was 10.4 days.<br>Clinical cure or improvement was seen in 115/118 (98%) evaluable patients.<br>Bacteriologic eradication was noted in 78 (94%) of the 83 evaluable patients.
104	Rodriguez-Cerrillo et al (Spain)	Case-series	SN (HHU)	24 patients (mean age 73)	—	Mean duration of Hospital at Home Unit (HHU) stay was 9 days.<br>All patients had a favourable clinical outcome (24/24).
105	Rodriguez-Cerrillo et al (Spain)	Case-series	SN (HHU)	25 patients (mean age 59); 48% male	—	Mean duration of HHU stay was 11 days.<br>All patients (25/25) had a favourable clinical outcome.
106	Seaton & MacConnachie (UK)	Case-series (retrospective)	SA; Other (unspecified)	19 patients (mean age 55); 53% male	—	Average duration of OPAT was 34 days (range 3-128d).<br>Twelve of 19 patients (63%) were cured, while four (21%) were improved).
110	Seaton et al (UK)	Observational (retrospective)	OP	963 patients (median age 48); 59% male	—	Median duration of treatment was 3 days (IQR 2-5d).<br>The success rate (no progression of infection, no re-admission and no significant adverse events) was 87.1% (839/963 patients).<br>Progression of infection was seen in 27/963 patients (2.8%).

SUPPLEMENTARY TABLES

ID	Author (country)	Study design	OPAT Model(s)	OPAT group(s)†	Comparator(s)†	Findings
112	Seetoh et al (Singapore)	Cohort	OP; SA; SN	2,229 patients (median age 56); 64% male	—	Treatment was completed as planned in 1,874 (84.1%) of 2,229 episodes.<br>Median time to treatment completion was 16 days (IQR 8-27 days).
113	Sims et al (UK)	Case-series (retrospective)	OP	14 patients (mean age 63); 71% male	—	Average duration of OPAT treatment was 58 days.<br>Overall, 11/14 joints (79%) were salvaged. Of the three failures, one patient improved initially but had a recurrence 5-months later (different organism identified recurrence), one had an excellent initial response but infection recurred 18-months later, and one developed infection with an atypical organism 3-years after primary surgery.
115	South (UK)	Case-series (retrospective)	OP; SA; Nurse (unspecified)	57 patients (mean age 41); 77% male	—	Median duration of non-inpatient treatment was 5 days (range 1-14d) for haematological malignancies, and 24 days (range 9-78d) for bone and joint infections.<br>Cure or improvement was achieved in 60/64 evaluable episodes (94.8%; 95% CI 88.2-99.8). There were two clinical failures (both Hickman line infections).<br>Bacteriological success was achieved in 33/40 evaluable episodes (82.5%; 95% CI 71.4-94.6).
117	Subedi et al (Australia)	Case-series	SA; SN	144 patients (median age 55); 74% male	—	Median duration of OPAT treatment was 22 days (range 4-106 days).<br>Overall cure rate was 93% (140 episodes).
118	Talcott et al (USA)	Observational (pilot)	SA; SN	30 patients (median age 38); 43% male	—	Median duration of treatment was 3.5 days (range 1-24d).<br>Twenty-one of 30 patients (70%) completed home therapy without complication or readmission. Medically eligible patients who did not enrol in the trial had a shorter duration of neutropenia (median 4 days vs. 6 days; p<0.005) than enrolled patients.
121	Theocharis et al (Greece)	Case-series (retrospective)	SN	91 patients (mean age 85); 31% male	—	Cure rate was 72.5% (66/91).<br>Mean duration of treatment was 4.7 days (range 1-18d).
123	Tice (USA)	Case-series	OP; SA; SN	538 patients (mean age 45); 52% male	—	Clinical improvement was recorded in 484/491 patients who could be evaluated, (98.6%); failure was recorded in seven patients.<br>Eradication was reported in 244/265 cases (92%) where bacteriological outcome could be assessed. There was persistence in 17 cases (6.4%) and superinfection in four (1.5%).

SUPPLEMENTARY TABLES

ID	Author (country)	Study design	OPAT Model(s)	OPAT group(s)†	Comparator(s)†	Findings
124	Tice (USA)	Observational (retrospective)	OP; SA; SN; Infusion centre.	500 patients	—	Most patients (269/424; 56%) were treated for at least four weeks, although treatment duration varied from <1 week to 11 weeks.<br>Most patients assessed for clinical outcome were improved (259/266; 97%). There were failures in three patients and no change in four others. Most failures occurred in the first few months (65% in ≤3m); the majority (95%) in the first year.
126	Upton et al (New Zealand)	Case-series	SA	100 patients (mean age 51); 59% male	—	Median duration of treatment was 22 days (range 3-160d).<br>Cure was achieved in 94/107 treatment courses in 88 patients (88%). Ten patients relapsed, one did not respond, and one died.
127	Walton et al (Australia)	Case-series	SN	35 patients (mean age 50); 71% male	—	Mean duration of treatment was 24 days (range 6-47d).<br>Of 31 patients who were assessable after treatment completion, all were clinically and bacteriologically cured (31/31).<br>Twenty of 31 patients (65%) were reviewed ≥2 months after completion of treatment (median 6.5m; range 2-21m), and all remained free of relapse for the duration of follow-up.
128	White et al (Australia)	Case-series (retrospective)	OP; SN; GN	55 patients (mean age 50); 65% male	—	Median duration of IV therapy, inc. time spent as an inpatient, was 42 days (range 4-79d).<br>Median duration of treatment was 22 days (range 3-56d).<br>Forty six of 55 patients (84%) were considered to have had a successful outcome at completion of IV OPAT.
129	White et al (UK)	Case-series (retrospective)	SA	72 patients (mean age 42); 42% male	—	Median duration of treatment was 21 days (range 1-43d).<br>Definite improvement was seen in 20/72 patients (28%), modest/slight/transient improvement in 24 patients (33%), and no improvement was seen in 26 patients (36%).
133	Wynn et al (USA)	Observational (retrospective)	Physician-based program; Home health organisation; Hospital-based program (no other details)	1252 patients (mean age 52); 65% male	—	A total of 1202/1252 patients (96%) were treated successfully for their condition.
134	Yadlapalli et al (USA)	Case-series (retrospective)	Not specified	58 patients (mean age 60); 83% male	—	Mean duration of antibiotic therapy was 40.3 days (range 19-90d).<br>At the end of 12-months follow-up, 46/58 patients (79.3%) were cured (mean healing time 15.4 weeks). Three of the 12 patients (5.2%) who failed to heal had an amputation, and nine (15.5%) had persistence of ulceration after a year.

For peer review only - http://bmjopen.bmj.com/site/about/guidelines.xhtml

SUPPLEMENTARY TABLES

ID	Author (country)	Study design	OPAT Model(s)	OPAT group(s)†	Comparator(s)†	Findings
135	Yan et al (UK)	Case-series (retrospective)	OP; GN	140 patients (aged 17-89); 64% male	—	Mean duration of treatment for patients with cellulitis (n=128) was 4.4 days.<br>Duration of treatment ranged from 0 to >15 days. Around 60% of patients received IV antibiotics for ≤3 days.<br>The failure/complication rate among patients was low (8/140; 5.7%).

OP: outpatient; SA: self-administered; SN: specialist nurse; GN: General nurse.

† Age and gender details relate only to those patients who were clinically evaluable or provided questionnaire data.

For peer review only

SUPPLEMENTARY TABLES

TABLE S2 Safety associated with outpatient parenteral antibiotic treatment

ID	Author (country)	Study design	OPAT Model(s)	OPAT group(s)†	Comparator(s)†	Findings	Notes
**COMPARATOR STUDIES**							
20	Bedi et al (UK)	Cohort	SA	Home therapy (domiciliary IV); 52 patients (median age 61); 58% male<br>Home therapy (early supported discharge); 23 patients (median age 65); 35% male	Inpatient therapy; 36 patients (median age 71); 36% male	Thirty-day re-admission rates were similar across groups (inpatient 13.8%; ESD 12.5%; domiciliary IV 14.2%).<br>Antibiotic side effects developed in four inpatients (5%), two ESD patients (6.3%) and four domiciliary IV patients (4.7%).<br>There were no IV access-related complications in the inpatient group, two (6.3%) in the ESD group, and three (3.6%) in the domiciliary IV group.<br>No deaths were recorded in any group.	Reasons for admission were: exacerbation of bronchiectasis (all).<br>Access-related complications were: line blockage (3), line fell out (2), and line sepsis (1).
30	Corwin et al (New Zealand)	RCT	GN	Home therapy; 98 patients (mean age 55); 62% male	Inpatient therapy; 96 patients (mean age 48); 73% male	Eleven of 98 patients in the Home group (12%) were admitted to hospital.<br>Three of 96 hospital patients (3%) required re-admission within one month.	Reasons for admission were: unsatisfactory clinical improvement (4), surgical drainage (1), PICC line insertion (2), ischaemic toe (1), rash (1), nausea/vomiting on starting post-discharge oral antibiotics (1), and not coping at home (1).
32	Dall et al (USA)	Before and after	SN	Home therapy; 92 pneumonia patients (97% aged >50 years); 44% male / 64 cellulitis patients (mean age 51); 70% male	Inpatient therapy; 10728 pneumonia patients (83% aged >50 years); 51% male / cellulitis patients (no numbers provided)	Two of 92 patients with pneumonia were re-admitted (rate over 30-day period, 2%). The re-admission rate for comparator patients was 7.4% (69/933 patients).<br>Six cellulitis patients were hospitalised briefly (9.4%). One treatment failure resulted in re-admission (for MRSA).<br>There were no deaths among OPAT patients.	
39	Escalante et al (USA)	Clinical trial (unspecified)	Not specified	IV therapy; 43 patients (median age 39); 42% male	Oral therapy; 40 patients (median age 52); 50% male	Three of 40 patients receiving oral treatment had an adverse drug reaction.<br>Six patients receiving oral treatment (15%) were admitted to	Adverse drug reactions were: renal toxicity (3).<br>Reasons for admission were: renal toxicity (3), and persistent fever (3).

SUPPLEMENTARY TABLES

ID	Author (country)	Study design	OPAT Model(s)	OPAT group(s)†	Comparator(s)†	Findings	Notes
						hospital; there were no admissions in patients receiving IV treatment.<br>There were no infection-related complications or deaths.	
40	Esmond et al (UK)	Controlled trial	SA	Home therapy; 15 patients (mean age 27); 40% male	Inpatient therapy; 15 patients (mean age 23); 60% male	There were no drug reactions, IV line problems or sepsis reported in either group.	
44	Fernandez-Aviles et al (Spain)	Case-control	SN	Home therapy; 50 patients (mean age 47); 62% male	Inpatient therapy; 50 patients (median age 50); 54% male	There was no significant difference in mucositis (Home 24% vs. 34%), diarrhoea (35% vs. 39%) or bacteraemia between the two groups.<br>In 40% of Home patients' day unit consultations, physician intervention was required.<br>Four Home patients (8%) were re-admitted. The median number of admitted days was 8 days for Home patients and 25 days for Hospital patients (p<.00001).<br>No patients in either group died.	Reasons for intervention were: fever (75%), catheter-related complication (10%), and mucositis, bleeding, diarrhoea, or chemotherapy related exanthema (15%).<br>Reasons for re-admission were: fever with hemodynamic instability (3), and pneumonia (1).
58	Keller et al (USA)	Before and after	Home agency; skilled nursing facility; other (not specified)	Pre-infectious disease transition service; 215 patients (mean age 56); 60% male<br>Standard OPAT (pre);70 patients (mean age 58); 69% male	Post-IDTS; 147 patients (mean age 57); 54% male<br>Standard OPAT (post); 56 patients (mean age 55); 68% male	Overall, 199 patients (40.8%) experienced either a re-admission and/or ED visit within 60 days of discharge (IDTS arm: pre n=82, 38.1%; post n=41, 27.9%; standard OPAT arm: pre n=35, 50.0%; post n=16, 28.6%).<br>There was no relationship between the presence of the IDTS and re-admission and/or ED visit at 60 days (adjusted OR 0.48; 95% C, 0.13-1.79). Similar results were observed for 7-day and 30-day re-admissions and/or ED visits.<br>The IDTS was associated with decreased antimicrobial prescribing errors (OR 0.062; 95% CI, 0.015–0.262), increased receipt of laboratory test results (OR 27.85; 95% CI, 12.93–59.99), and increased follow-up (OR 2.44; 95%	

SUPPLEMENTARY TABLES

ID	Author (country)	Study design	OPAT Model(s)	OPAT group(s)†	Comparator(s)†	Findings	Notes
						CI, 1.50–3.97).<br>During the 60-day period after discharge, drug-related adverse events, catheter complications, and infection relapse were common.	
61	Lacroix et al (France)	Observational (retrospective)	Not specified	OPAT; 18 patients (mean age 60); 61% male	Inpatient therapy; 21 patients (mean age 68); 67% male	During OPAT, three patients (16.7%) presented with severe adverse events requiring re-hospitalization; none were attributed to OPAT.<br>Following OPAT, three patients (16.7%) presented with severe adverse events, including one death. This compares with seven re-admitted patients (33.3%) and one death (4.8%) in the hospital group.	Reasons for re-admission were: haemorrhagic stroke with subdural haematoma (1); fever due to beta-lactams requiring re-hospitalization for a switch to vancomycin (1); cardiac failure (1).<br>Adverse events were: death from pneumonia with severe hypoxemia (1), relapse of IE (1), and progressive mitral regurgitation requiring mitral surgery after IE cure (1).
75	Martone et al (USA)	Observational (retrospective)	Not specified	OPAT; 539 patients (57% aged >50 years); 56% male	Inpatient therapy; 410 patients (63% aged >50 years); 45% male	A total of 216 adverse events were reported in 50/539 OPAT patients and 81/410 hospital patients (9.3% vs. 19.8%; p<0.0001).<br>A total of 89 adverse events possibly related to therapy were experienced by 31 OPAT and 34 hospital patients (5.8% vs. 8.3%; p=0.12). Significantly more hospital patients had diarrhoea (2.4% vs. 0.2%; p=0.001).	
76	Matthews et al (UK)	Cohort (retrospective)	SA; GN; Community hospital staff	Self-administered OPAT; 513 episodes (mean patient age 46)	Professional-administered OPAT; 1621 episodes (mean patient age 61)	Drug associated complications were experienced in 189/1536 H-OPAT episodes (12%) and 59/473 S-OPAT episodes (12%).<br>Complications related to vascular access affected eight (0.5%) H-OPAT patients and five (1%) S-OPAT patients (p=0.2).<br>The overall complication rate was 23% (353/1536 episodes) for H-OPAT patients and 24% (112/473 episodes) for S-OPAT (p=0.7).<br>Re-admission occurred for 193 H-OPAT and 50 S-OPAT patients (rates of 12.6% and 10.5%	Drug related complications included: GI side-effects (58), drug rash (52), drug fever (51), unclassified drug reaction (45), neutropenia (41), anaphylaxis (4), renal impairment (1), and C-diff diarrhoea (1).<br>Access-related complications were: line infection (3), leaking line (3), mechanical phlebitis (2), and thrombosis (6).

SUPPLEMENTARY TABLES

ID	Author (country)	Study design	OPAT Model(s)	OPAT group(s)†	Comparator(s)†	Findings	Notes
						respectively; p=0.3). Two H-OPAT patients died (unrelated cardiac causes).	
78	Mazo et al (Spain)	Case-control (retrospective)	Hospital in the Home (HHU)	HHU therapy; 150 patients (mean age 67); 47% male	Inpatient therapy; 150 patients (mean age 63); 51% male	HHU decreased the number of complications or re-admissions to the surgical or emergency unit at 45 days (HHU rate 0.67% vs. hospital rate 3.5%).	
81	Montalto & Dunt (Australia)	Observational (retrospective)	SN (HHU)	HHU therapy; 55 cellulitis patients (mean age 52); 60% male / 14 pyelonephritis patients (mean age 30); 0% male	Inpatient therapy; 22 cellulitis patients (mean age 51); 73% male / 10 pyelonephritis patients (mean age 35); 30% male	Incidents were recorded for 3/69 HHU patients (4%) and 10/32 Hospital patients (31%). Four Hospital patients (three with cellulitis, one with pyelonephritis) were re-admitted within four weeks.	HHU incidents were: drainage of abscess (2), and vasovagal episode at administration of medication (1). Hospital group incidents were: unexpected non-urgent operation (2), drug reaction (1), unexpected returned to hospital within four weeks (4), anaphylaxis (1), re-admission for related condition (1), and unexpected gastroscopy (1).
98	Pond et al (UK)	Case-control (retrospective)	SA	Home therapy; 25 patients (mean age 21); 56% male	Inpatient therapy; 25 patients (mean age 22); 56% male	There were 2/25 (8%) adverse reactions in each group. All consisted of mild skin rashes that subsided with withdrawal of the relevant antibiotic.	
101	Rapoport et al (Multinational)	RCT	OP	Outpatient therapy; 40 patients (median age 45); 52% male	Inpatient therapy; 44 patients (median age 48); 32% male	Incidence of adverse events was similar for OPAT patients (6/40; 15%) and inpatients (8/44; 18%). Three events (one OPAT, two inpatients) were considered to be potentially drug related; two events were severe (one in each group). There was one death in the OPAT group.	
102	Rehm et al (USA)	RCT	SA; SN; (also skilled nursing facility; assisted-living; nursing home; rehab centre)	Home therapy; 103 patients (median age 50); 58% male	Inpatient therapy; 97 patients (median age 54); 61% male	Fewer patients in the OPAT group (48/103; 46.6%) experienced a serious adverse event compared with hospital patients (52/97; 53.6%). A total of 53 hospital patients (54.6%) discontinued therapy compared with 10 OPAT patients (9.7%). This was most commonly	Reasons for OPAT discontinuation were: skin problems (4), renal failure (2), diabetic gastroparesis (1), and blood creatine phosphokinase increased (1). Reasons for inpatient discontinuation were: cardiovascular problem (4), skin

SUPPLEMENTARY TABLES

ID	Author (country)	Study design	OPAT Model(s)	OPAT group(s)†	Comparator(s)†	Findings	Notes
						related to adverse events (80% of OPAT; 47% of hospital).<br>Eighteen of 103 OPAT patients (17.5%) required re-admission.<br>Fewer deaths occurred in the OPAT group (n=4; 3.9%) than in the hospital group (n=18; 18.6%) (p=0.001).	problem (4), infection (3), renal failure (3), blood creatine phosphokinase increased (2), fever (2), sepsis (2), anaphylactic reaction (1), hypoxia (1), red man syndrome (1), thrombocytopenia (1), and vomiting (1).<br>Reasons for OPAT re-admission were: other medical condition (11), related to initial infection (4), and problems related to treatment provision in post-acute care setting (3).
103	Richards et al (New Zealand)	RCT	GN	Home therapy; 24 patients (mean age 50); 54% male	Inpatient therapy; 25 patients (mean age 50); 52% male	Two patients in each group reported nausea and candidiasis.<br>There were five extra-pulmonary infections in Home patients (21%) and four in Hospital patients (16%), and two pulmonary complications in the Home group (8.3%) and one (4%) in the Hospital group.<br>Two of 24 patients were transferred from the Home group to hospital (one with pulmonary infection). One Hospital patient was re-admitted (clinical deterioration).<br>There were no deaths in either group.	Reasons for home to hospital transfer were: development of empyema in legionella infection (1); development of bullous myringitis (1).<br>Extra-pulmonary infection in the Home group included three IV site infections.
109	Seaton et al (UK)	Before and after	SN	Standard OPAT; 230 patients (median age 49); 52% male	Nurse-led OPAT; 112 patients (median age 50); 61% male	Medical review was required for 21/112 patients (19%) post-introduction of a nurse-led protocol.<br>Re-admission (6% vs. 7%), drug reaction (4% vs. 7%) and change in therapy rates (5% vs. 4%) were similar for pre- and post-protocol patients.	Reasons for review were: other medical problem (5), rash (8), incision of abscess required (3), and therapy change or admission for lack of improvement (5).
111	Sebban et al (France)	RCT	OP	IV therapy; 47 patients (mean age 52); 40% male	Oral therapy; 49 patients (mean age 52); 31% male	There were five adverse events in the IV group (5/46) and three in the Oral group (3/48). There were no statistical differences between the two groups for toxicity.<br>Nine IV patients (19.6%) and five Oral patients (10.4%) were re-admitted. There were no	Reasons for re-admission were: persistent temperature (9), chose to be re-admitted (2), severe dental infection (1), positive initial blood cultures (1), and allergic reaction to ceftriaxone (1).

SUPPLEMENTARY TABLES

ID	Author (country)	Study design	OPAT Model(s)	OPAT group(s)†	Comparator(s)†	Findings	Notes
						significant differences between groups in all-cause mortality, clinical deterioration, relapse of infection, new infection, modification of therapy, or re-admission.	
116	Stein et al (USA)	RCT	OP	IV therapy; 10 patients (mean age 54); 60% male	Oral therapy; 10 patients (mean age 53); 40% male	There were no side-effects in the OPAT group. Two of 10 patients in the Oral group (20%) experienced adverse events (one discontinued treatment). Four of 10 OPAT patients (40%) were hospitalised due to lack of improvement.	Adverse events were: skin and tongue pruritus (1), and diarrhoea (1)
131	Wolter et al (Australia)	RCT	SA	Home care; 13 admissions (median age 22); 28% male	Inpatient therapy; 18 admissions (median age 22); 39% male	There were no adverse drug reactions, short-term re-admissions, events or deaths attributable to the drugs used. One patient had a pneumothorax associated with central line insertion. There were no differences between groups in IV complication rates (p=0.57) or numbers of line changes required (p=0.5).	There was no significant difference in time to next admission between the two groups (p=0.68).
132	Wolter et al (Australia)	RCT	Not specified	Home care; 44 admissions (median patient age 43); 45% male	Inpatient therapy; 38 admissions (median patient age 49); 34% male	There were 23 adverse events reported in the Home group (52%) and 31 in the Hospital group (82%). Five were considered serious. Five Hospital and three Home group events were considered to be directly/possibly related to place of therapy. There were seven re-admissions in the Home group and four in the Hospital group 30 days after discharge. Three patients receiving home therapy had unplanned re-admission during treatment.	Serious events were: CVA (1), pneumothorax (1), line elated sepsis (1), and PICC vein thrombosis (2). Location related events were: Hospital patient with vancomycin resistant enterococci (4) or MRSA (1), Home patient with peripheral IV infection (1) or presented to another hospital for re-admission (1) or PICC line fracture (1).

SUPPLEMENTARY TABLES

ID	Author (country)	Study design	OPAT Model(s)	OPAT group(s)†	Comparator(s)†	Findings	Notes
136	Yang et al (Canada)	Case-control	Not specified	OPAT; 21 patients (mean age 59); 57% male	Previous standard care; 21 patients (mean age 59); 57% male	Incidence of 30-day re-admission was lower in the OPAT group than in the previous care group (14.3% vs. 28.6%, p=0.51), and mean length of hospital stay was shorter by 3.2 days (10.7 vs. 13.9, p=0.36).<br>The total incidence of adverse drug reactions (n=5, 23.8%) and VAD-related complications (n=2, 9.5%) were the same for each group. Three patients in the previous care group (60%) required a change or discontinuation of therapy compared with one (20%) in the OPAT group (p=0.62).<br>There were no re-admissions for adverse events in the OPAT group, and two in the previous care group (9.5%).	Adverse drug events included: allergy (3), haematological (1), renal/liver toxicity (2), GI-related (1), neurological (1), and abnormal drug level (2).
137	Yong et al (Singapore)	Case-control (retrospective)	OP; SA	OPAT; 69 patients (mean age 53); 53% male	Inpatient therapy; 93 patients (mean age 56); 51% male	Complications occurred in 18/72 OPAT episodes (25%), eight of which required re-admission.<br>At the end of 4-months follow-up, there was no difference in the number of OPAT (13/72; 18.1%) and hospital patients (18/93; 19.4%) re-admitted due to relapse (p=0.991).<br>There was one patient death in each group.	Complications were: PICC line related (8), adverse drug reaction (2), admission for underlying medical condition (7), and admission for adverse drug reaction (1).
**OPAT ONLY STUDIES**							
11	Al Ansari et al (Bahrain)	Case-series (retrospective)	OP	101 patients; 57% male	—	Two patients (2%) were re-admitted to hospital.	
12	Allison et al (USA)	Cohort (retrospective)	SN	782 patients (mean age 58); 57% male	—	Twenty-six percent (207/782) of patients were re-admitted within 30 days.<br>Nearly half of 16 patients prescribed aminoglycosides at discharge, (6) were re-admitted for medication side effects<br>Regression analysis showed that	Reasons for re-admission were non-ID related (63), worsening infection (62), new infection (48), adverse drug reaction (30), line complication (20), and diarrhoea (2).<br>Medication side-effects were: acute renal insufficiency (4), rash

SUPPLEMENTARY TABLES

ID	Author (country)	Study design	OPAT Model(s)	OPAT group(s)†	Comparator(s)†	Findings	Notes
						risk of re-admission was related to age (OR, 1.09 per decade; 95% CI 0.99-1.21; p=0.10), aminoglycoside use (OR, 2.33; 95% CI 1.17-4.57; p=0.01), drug resistant organisms (OR, 1.57; 95% CI 1.03-2.36; p=0.03), and number of prior hospital discharges without IV antibiotics in the past 12 months (OR, 1.20 per prior admission; 95% CI 1.09-1.32; p<0.001).	(1), rash and neutropenia (1).
13	Amodeo et al (New Zealand)	Observational (retrospective)	SA; GN	100 treatment courses (mean patient age 65); 75% male	—	Adverse events occurred in 27/100 episodes; 17 minor events managed in clinic, and 10 re-admissions.<br>There were two deaths in the 12-month follow-up period.<br>Five patients were re-admitted with further episodes of IE; mean number of days between completion of OPAT and re-admission was 220 (range 109-344d).	Minor adverse events were: ototoxicity (1), drug induced hepatitis (1), itch (1), PICC line occlusion (6), line migration (2), phlebitis (2), cellulitis (1), and high residual infusate unrelated to PICC line patency (3).<br>Reasons for re-admission were fever and rash (3), drug fever (2), diarrhoea and vomiting (1), hepatitis (1), worsening congestive heart failure (1), flank pain secondary to renal sub-capsular bleed (1), and recurrence of angina (1).
14	Anand et al (USA)	Case-series	Home IV therapy (unspecified)	52 patients	—	Seven of 52 patients (13%) had minor complications requiring a change of antibiotic or therapy duration.	Complications included: rash (1), increased LFTs (1), transient neutropenia / septicaemia (1), and bleeding at PICC site (1).
15	Angel (USA)	RCT	SA	62 patients (mean age 71); 53% male	—	Of 14 reported events, seven were judged unrelated to therapy.<br>There were no deaths during the study period.	Related adverse events were: phlebitis (2), rash (2), diarrhoea (1), nausea (1), and fever (1).
17	Barr et al (UK)	Observational (retrospective)	OP; SA	854 treatment courses; 55% male	—	Twenty of 854 patients had diagnosed line infections, 2.3% of all indwelling line episodes, and an incidence of 0.79 per 1,000 line use days (95% CI 0.68-0.91).<br>Incidence of other line events was 125/854 (14.6%), 4.9 per 1,000 indwelling line use days (95% CI 4.68-5.21).	There was a lower rate of line infection in Self compared to Clinic OPAT, but this was not statistically significance (OR 0.6846; 95% CI 0.2805-1.671; p=0.362).<br>There was a higher risk of other line events with Self compared to Clinic OPAT (OR 1.62; 95% CI 1.06-2.46; p=0.032). This was

SUPPLEMENTARY TABLES

ID	Author (country)	Study design	OPAT Model(s)	OPAT group(s)†	Comparator(s)†	Findings	Notes
							no longer significant in multivariate analysis (p=0.22).
18	Barr et al (UK)	Observational (retrospective)	OP; SA; SN; GN	2233 patients (median age 51); 58% male	—	Crude admission rate from OPAT was 11.7% (262/2233 patients). Unplanned re-admission was recorded for 204 patients (9.1%) (CR of 6.3 events per 1000 OPAT days).<br>A total of 219/2233 episodes (9.8%) were associated with adverse drug reaction (CR 6.7 events per 1000d).<br>Line infections occurred in 14/2233 patients (0.6%) (CR 0.4 events per 1000 days). Other line events occurred in 92 patients (4.1%) (CR 2.8 events per 1000d).<br>Eight patients died (0.4%).	Unplanned admission included: deterioration in infection (76), new medical event not infection related (63), adverse drug reaction (28), surgery (12), line complication (7), and logistic reasons (7).<br>Drug reactions were: rash (89), severe GI upset (38), chills or fever (28), leukopenia (22), thrombocytopenia or anaemia (21), nephrotoxicity (13), and hepatotoxicity (6).<br>Other line events included: line obstructed or leaking, 'chemical' or 'mechanical' phlebitis, patient default of follow-up with line in situ, and line fall out.
19	Barr et al (UK)	Observational (retrospective)	Not specified	780 patients (median age 52); 58% male	—	During or up to 90 days following OPAT, 34 patients (4.4%) were investigated for suspected symptomatic venous thromboembolism (VTE); two DVTs were diagnosed (incidence 2/780, 0.26% (95% CI: 0.03–0.92%)).<br>Up to 1 year following OPAT, there were a total of five VTEs (0.64%). The rate of VTE appeared constant in the year following OPAT.	
21	Berman & Johnson (USA)	Case-series (retrospective)	SA	221 patients (median age 41); 66% male	—	Side-effects were documented in 74/302 episodes (25%). In addition, 18% of patients (55 episodes) complained of constitutional symptoms.<br>Three patients (1.4%) were hospitalised for side-effects.	Side-effects included: renal dysfunction (21), drug rash (18), anaemia (13), diarrhoea (13), vestibular dysfunction (11), fever (9) thrombocytopenia (8), neutropenia (7), and hepatitis (3).<br>Constitutional symptoms were: fatigue (20), headache (12), nausea (19), anorexia (5), weakness (2), palpitations (1), sleepiness (1), and insomnia (1).

SUPPLEMENTARY TABLES

ID	Author (country)	Study design	OPAT Model(s)	OPAT group(s)†	Comparator(s)†	Findings	Notes
22	Bernard et al (France)	Observational (?)	SA; SN;	39 patients (mean age 44); 64% male	—	Four of 39 patients reported adverse events (10%). Four of 39 patients were re-admitted for medical reasons (10%). There were no haematological or renal complications.	Adverse events were: rash (2), and nausea and vomiting (2). Reasons for re-admission were: deep venous thrombophlebitis (3), and allergic reaction (1).
24	Cervera et al (Spain)	Observational	SA; SN	73 patients (mean age 60); 75% male	—	Twelve of 73 patients (16.4%) had complications requiring re-admission. Three patients had fatal complications.	Non-fatal events were: heart failure (2), catheter-related sepsis (1), variceal haemorrhage (1), abdominal pain (1), dizziness (1), lower-back pain (1), fever (1), and hypersensitivity reaction (1). Fatal complications were: health-care related pneumonia (1), cerebral haemorrhage (1), and pulmonary oedema (1).
25	Chambers et al (New Zealand)	Case-series	SA; SN	153 patients (median age 55); 58% male	—	Complications of treatment developed in 31/153 (20%) patients. All catheter related infections (n=3) resolved with removal of the devices. Fifteen patients (10%) were re-admitted within one month of discharge from hospital.	Complications were: phlebitis (9), line occlusion (12), line leakage or breakage (6), infection (3), thrombosis (1), and pump failure (4). Reasons for re-admission were: washout of infected prosthesis (2), slow resolution of cellulitis (3), jugular vein thrombosis (1) line sepsis (1), uncontrolled pain (3), discharged to inappropriate level of care (1), and unrelated condition (4).
27	Chan et al (Singapore)	Observational	Not specified	109 patients (mean age 57); 66% male	—	Nine patients were re-admitted from OPAT. There were no deaths or relapses at 30 days post cessation of antibiotics.	Reasons for re-admission were: surgical drainage of abscess after initial drainage failure (3), worsening of underlying non-infectious comorbidity (5), and infectious condition unrelated to KLA (1).
28	Chapman et al (UK)	Observational (retrospective)	SA; Infusion centre	334 treatment courses (mean patient age 46); 59% male	—	Twenty-one of 334 treatment episodes (6.3%) ended in re-admission. In 11 episodes OPAT was terminated because of a change of plan. Two patients developed C-diff	Reasons for re-admission were: unrelated to OPAT (6), inappropriate referral (6), non-resolving soft tissue sepsis (3), deep sepsis with inadequate clinical response (1), symptom control (1), alternative IV therapy

SUPPLEMENTARY TABLES

ID	Author (country)	Study design	OPAT Model(s)	OPAT group(s)†	Comparator(s)†	Findings	Notes
						diarrhoea during IV antibiotic therapy; a further patient had diarrhoea on starting OPAT, and was found to have C. difficile toxin.	following antibiotic reaction (1), line infection (1), fractured humerus after fall travelling to OPAT unit (1), cut off end of PICC self-administering antibiotics (1).
29	Cheong et al (Australia)	Case-series	GN	714 treatment courses (mean patient age 53); 42% male	—	Adverse drug reactions occurred in 38/714 courses of treatment (5.3%).<br>Drug reactions in five patients were ranked as serious or life-threatening (all were re-admitted).	Adverse events included: skin reaction (18), abdominal symptoms (8), vascular symptoms (5), renal function changes (4), hypotension (2), shortness of breath (1), and severe cutaneous symptoms (1).
31	Cox et al (USA)	Observational (retrospective)	SA	205 patients (mean age 59); 99% male	—	Adverse events occurred in 99/231 (43%) home IV courses.<br>Venous access complications were frequent (n=57; 25%) but rarely serious. Catheter or infusion problems resulted in admission in five treatment courses.<br>All other events related to adverse drug reactions (n=42; 18%).	Venous access complications were: catheter pulled out (22), occluded catheter (18), irritation from dressing (6), site infection (4), leakage (3), bacteraemia (3), and phlebitis (1).<br>Adverse drug reactions were: nephrotoxicity (10); eosinophilia (9), rash (8), leukopenia (7), anaemia (5), and thrombocytopenia (3).
33	Dalovisio et al (USA)	Observational (retrospective)	SA; SN	62 patients (mean age 69)	—	Five of 66 treatment courses (7.6%) resulted in re-admission within 30 days; none were directly related to the infectious diagnosis.<br>Twelve of 62 patients (19.4%) experienced line complications.<br>No patients died while receiving IV treatment.	Reasons for re-admission included: pneumonia, chest pain, surgical wound infection, hepatic failure, and tumour fever.<br>Line complications included: line breakage, leakage, infection, and infiltration.
34	Dargan et al (Canada)	Case-series (retrospective)	OP; Home therapy (unspecified)	66 patients (mean age 57); 50% male	—	Nineteen of 66 patients (29%) experienced a total of 25 complications. Most (n=15) were attributed to IV access, and the remainder to adverse drug reactions.<br>Five patients (all treatment failures) were re-admitted to hospital for reasons attributable to the infection.	Access related events were: interstitial of IV line (6), occluded line (3), phlebitis (3), bleeding (1), leakage of PICC line (1), and thrombosis (1).<br>Drug related events were: nausea (3), acute renal failure (1), allergic reaction (1), hypokalaemia (1), nephrotoxicity (1), neutropenia (1), vomiting (1), and yeast infection (1).

SUPPLEMENTARY TABLES

ID	Author (country)	Study design	OPAT Model(s)	OPAT group(s)†	Comparator(s)†	Findings	Notes
35	Dobson et al (Australia)	Case-series	SA; SN; GN	770 patients (mean age 48); 66% male	—	Allergic reactions were experienced by 28/770 patients (3.6%). No patient had anaphylaxis.<br>Mean time from treatment start to reaction onset was 19.6 days (range 1-39d).	Allergic reactions included: rash (13), rash with pruritus (4), fever (3), and perioral angioedema (3).<br>No reactions were experienced by patients who received drugs via bolus or intermittent administration alone (although this did not reach significance (OR 0; 95% CI 0-1.7)).
36	Donald et al (Australia)	Case-series (retrospective)	SN	124 patients (aged 16-97); 60% male	—	One patient developed self-limiting diarrhoea. There were no other complications attributable to therapy.<br>Nineteen of 124 patients (15%) were re-admitted.	Reasons for re-admission were: failure of cellulitis to resolve (13), abscess requiring surgery (4), analgesia needed (1), and admission for central venous access (1).
37	Duncan et al (UK)	Observational (retrospective)	Not specified	1377 treatment courses	—	A total of 51 adverse reactions were observed in 1377 episodes (3.7%), a prevalence rate of 37.0 per 1,000 new patient episodes.	Adverse reactions were: rash (20), hepatic enzyme abnormality (10), leukopenia, thrombocytopenia or anaemia (6), GI symptoms (5); anaphylaxis (4), chills or fever (3), and acute kidney injury (3).
38	Duncan et al (UK)	Observational (retrospective)	Not specified	55 completed OPAT patients (median age 59); 80% male / 25 failed OPAT patients (median age 67); 60% male	—	Twenty-one episodes resulted in re-admission (26.3%), seven in an adverse drug event (8.8%), three in line complications (3.8%), and two in drug resistance (2.5%).	Reasons for re-admission included: suspected endocarditis decompensation (7), and non-endocarditis related (4),<br>Reasons for OPAT failure included: hyperkalaemia (1), vomiting (1), acute renal dysfunction (1), presyncope during administration (1) and resistance to oral component of the OPAT regimen (1).
42	Esposito et al (Italy)	Observational (retrospective)	OP; SA; Visit by doctor or nurse (also care facility)	239 patients (aged 11-80+); 62% male	—	Adverse drug reactions, usually mild, were detected 27/239 patients (11%), and were more frequently seen in patients who received combination therapy (74.1% vs. 25.9%).	Adverse reactions were: rash (12), fever (3), hepatic impairment (3), nausea/vomiting (2) renal impairment (2), anaphylactic reaction (1), diarrhoea (1), itching (1), leukopenia (1), and urticaria (1).
43	Esposito et al (Italy)	Observational	OP; SA; Visit by doctor or nurse (also care facility)	176 patients (aged >65 years); 47% male	—	Adverse drug reactions, usually mild, were detected in 22/176 patients (13%).	Adverse reactions were: rash (7), fever (3), itching (3), renal impairment (2), hepatic impairment (1), diarrhoea (1), anaphylactic reaction (1),

SUPPLEMENTARY TABLES

ID	Author (country)	Study design	OPAT Model(s)	OPAT group(s)†	Comparator(s)†	Findings	Notes
							eosinophilia (1), dizziness (1), urticaria (1), and dyspnoea and palpitations (1).
45	Goodwin et al (Canada)	Case-series	SN; GN	2405 patients (mean age 46); 55% male	—	Thirty-three of 1642 patients (2%) discontinued therapy before prescribed completion (treatment related in 25 cases).<br>Fifty-two of 1642 patients (3.2%) were admitted to hospital.<br>Fifty-four of 1642 patients (3.3%) had unplanned interruptions to therapy; only two were unrelated to treatment (comorbidity).	Reasons for discontinuation were: adverse effect of therapy (9), inadequate venous access (5), unscheduled admission due to worsening infection (4), early resolution of infection (5), change of therapy (4), change to oral therapy (9), comorbidity (5), and patient decision (3).<br>Reasons for admission were: comorbidity (36), treatment failure (12), and inability to manage self-therapy (4).<br>Reasons for interruption were: venous access complication (15), temporary loss of venous access (26), device problem (n=18).
46	Gourdeau et al (Canada)	Case-series (retrospective)	OP; SA	124 patients (mean age 41); 56% male	—	Two of 124 patients (1.6%) experienced adverse reactions requiring treatment modification.<br>There were no cases of catheter-related infection.<br>One patient was re-admitted (due to lack of improvement).	Reasons for treatment modification were: rash (1), and recurrent phlebitis (1).
47	Graninger et al (Austria)	Case-series	OP	54 patients	—	Adverse events were seen in nine of 54 patients (17%).	Adverse events were: thrombocytopenia (3), rash (3), nausea (1), C-diff colitis (1), fever (1), transient hearing impairment (1), and leukopenia (1).
48	Grayson et al (Australia)	Case-series	GN	20 patients (mean age 58)	—	No major infective complications related to IV antibiotic administration were noted.<br>Two complications were seen (2/20; 10%).	Complications were: localised soft-tissue infection at catheter exit site (1), and progressive renal dysfunction (1).
49	Gross et al (USA)	Case-series (retrospective)	Home IV therapy (unspecified)	13 patients (mean age 38); 100% male	—	Eleven patients (85%) experienced 22 adverse events (one patient had four, two had three, four had two and four had one).<br>There were no deaths.	Adverse events were hypoglycaemic (8), nephrotoxicity (6), hypotension (5), hyperkalaemia (1), hypercalcaemia (1), and elevated

SUPPLEMENTARY TABLES

ID	Author (country)	Study design	OPAT Model(s)	OPAT group(s)†	Comparator(s)†	Findings	Notes
							pancreatic enzymes.
50	Heintz et al (USA)	Observational	OP; SA; SN; Infusion centre (also long-term care facility; interim-care facility; prison)	494 treatment courses	—	Infection and/or OPAT-related complications occurred in 35/494 cases (7.1%).<br>Intervention by the ID pharmacist post discharge occurred in 26/494 cases (5.3%).	Complications included: re-admission and/or emergency department visit within 30 days of discharge (28), 30-day infection-related mortality (2), and drug-induced toxicity (11).<br>Drug-induced adverse effects were: rash/hives (5), nephrotoxicity (3), bone-marrow suppression (2), and intractable nausea and vomiting (1).
51	Hindes et al (USA)	Case-series	SA	48 patients (mean age 65)	—	Complications were seen in four of 48 patients (8%).<br>Three patients required re-admission (6%),	Complications were: serum sickness reaction (3), and subclavian vein thrombosis (1).<br>Reasons for re-admission were: serum sickness (1), new site infection (1), and seizure unrelated to therapy (1).
52	Hitchcock et al (UK)	Case-series	OP; SA; GN; Private home healthcare company	273 patients (mean age 60); 54% male	—	Twenty three of 303 courses (8%) were followed by re-admission during therapy or within 28 days of completion.<br>Thirteen admissions were related to the original diagnosis or to OPAT drug treatment; 10 were unrelated.	Reasons for OPAT re-admission were: surgery after treatment failure (3), line problem (2), adverse drug effect (2), treatment failure at end of original course (3), super infection (1), and worsening clinical condition (2).
53	Ho et al (Singapore)	Case-series	OP	29 patients (median age 41); 90% male	—	Six of 29 patients (21%) required re-admission, five during OPAT and one during the 30 day follow-up period.<br>There were no deaths or significant misadventures.	Hospital re-admissions were: PICC infections (2), complications related to endocarditis (2), bacteraemia due to a different organism (2).<br>Incidence of re-admission and PICC infection was similar to that found in non-IVDU endocarditis patients treated in OPAT.
54	Htin et al (Australia)	Case-series (retrospective)	SN	68 patients (median age 68); 87% male	—	Three patients (4%) developed complications and required re-admission.<br>There were two deaths, both	Reasons for re-admission were: line-related bloodstream infection (1), fever due to flare of gouty arthritis (1), worsening anaemia in IHD, and Crohn's disease (1).

SUPPLEMENTARY TABLES

ID	Author (country)	Study design	OPAT Model(s)	OPAT group(s)†	Comparator(s)†	Findings	Notes
						unrelated to OPAT. Of the remaining 66 patients, 65 were still alive and 1 patient was lost to follow-up (1-year survival 96% (65/68)).	
55	Huminer et al (Israel)	Case-series	SA; SN (also nursing home; kibbutz)	37 patients (mean age 64); 57% male	—	Eight of 37 patients (22%) experienced adverse drug reactions.<br>Ten of 37 patients had local complications (27%).<br>Six patients were re-admitted before completing OPAT.	Adverse drug reactions were: fever (5), rash (2), and syncope (1).<br>Local complications were: occluded line (10), thrombophlebitis (6), and intravascular infection (1)<br>Reasons for admission were: fever (5), and PSV tachycardia (1).
56	Johansson et al (Sweden)	Case-series	SA	11 patients (mean age 51); 73% male	—	One patient telephoned the hospital on one occasion for help with CVAD handling.<br>One patient developed a CVAD exit-site infection.<br>All other patients recovered without re-admission and without antibiotic changed.	
57	Kayley et al (UK)	Case-series	SA; GN; Doctor administered	67 patients	—	Complications of home therapy were minimal (17/67; 25%).<br>Line infections (n=3) were all seen in HIV positive patients who were treated for >6 months continuously.	Complications were: line infection (3), broken line clamp (5), blocked line (2), dislodged line (3), drug allergy (2), and C-diff diarrhoea (2).
59	Kieran et al (Ireland)	Case-series (retrospective)	SA; Nurses attached to commercial company	56 patients (median age 50); 57% male	—	Adverse events occurred in 12/60 episodes (20%).<br>Five of 56 patients (7%) had a line-related complication (rate of 3.9 per 1,000 outpatient central line days).<br>Drug-related adverse events occurred in 4/60 episodes (6.7%).<br>In seven of 60 episodes (12%), patients required re-admission related to an adverse event; in five cases (8.3%) this was therapy related.	Line complications were: line infection (2), line blockage (2), and dislodged line (1).<br>Drug-related events were: rash (2), acute hepatitis (1), and neutropenia (1).<br>Reasons for re-admission were: acute hepatitis (1), neutropenia (1), failure to manage OPAT (1), anxiety (1), and line infection (1).

SUPPLEMENTARY TABLES

ID	Author (country)	Study design	OPAT Model(s)	OPAT group(s)†	Comparator(s)†	Findings	Notes
62	Lai et al (USA)	Case-series (retrospective)	SA; SN	333 patients (mean age 62); 98% male	—	Complications occurred in 96/393 OPAT courses (24.4%). The most common was re-admission (n=49, 12.5%).<br>Adverse drug events were present in 40 of the 393 courses (10.2%). Shortening of OPAT (including switches to oral antimicrobial therapy) occurred in 29 of the 40 cases (72.5%; 7.4% of all).<br>The overall line-related complication rate was 6.4% (25/393) over the period (PICC line, n=22; peripheral IV line, n=3).<br>Rates of PICC complications decreased across the period (8.4% to 4.5%), although this was not statistically significant.	Reasons for re-admission included: failure to improve on OPAT (15), reason unrelated to OPAT (13), PICC-related (9), and adverse drug event (8).<br>Adverse drug events were: acute kidney injury (11), pruritus/rash (10), leukopenia (7), GI problems, including diarrhoea (6), and C-diff associated diarrhoea (2).<br>Access-related complications included: occlusion/accidental displacement (9), erythema or tenderness (6), and bloodstream infection (6).
63	Lane et al (USA)	Cross-sectional	OP; SA	555 adult infectious diseases physicians	—	OPAT-associated complications were perceived as being rare (≤5% of patients).<br>The most commonly reported in greater numbers than this were line occlusion/ (80% of respondents), rash (39%), and nephrotoxicity (39%).<br>Patients commonly required line exchange or removal or change in antibiotic therapy because of complications; hospitalization was less common.	
64	Larioza et al (USA)	Case-series (retrospective)	Home infusion company	43 patients (56% aged >50 years); 67% male	—	Ten of 43 patients (23%) were re-admitted to hospital during antibiotic treatment.	Reasons for re-admission were: antibiotic-associated diarrhoea (1), PICC-related issues (6), interstitial nephritis (1), and drug rash (2).
65	Larioza et al (USA)	Case-series (retrospective)	Home infusion company	33 patients (76% aged ≥50 years); 61% male	—	Four of 33 patients (12.1%) developed complications.<br>There were no deaths.	Complications were: line infection (2), deep vein thrombophlebitis (1), and C-diff diarrhoea (1).

SUPPLEMENTARY TABLES

ID	Author (country)	Study design	OPAT Model(s)	OPAT group(s)†	Comparator(s)†	Findings	Notes
66	Laupland et al (Canada)	Observational	OP; SA; SN	2405 patients (mean age 46); 55% male	—	Overall, 139 of 3145 patients (4%) were admitted to hospital within seven days of starting home therapy.<br>Another 82 patients (3%) had interruptions to therapy.	Reasons for interruption were: venous access related (45), infusion delivery system related (30), comorbidity (3), and unspecified (4).
69	Lillie et al (UK)	Case-series	SN	98 patients (mean age 55); 61% male	—	Of 64 patients attending 4-week follow-up, two (3.1%) had a relapse of infection; one patient required admission for treatment.	
70	Lin et al (USA)	Case-series (retrospective)	Not specified	177 patients	—	Antibiotic complications occurred at a rate of 16% (28/177). Most were minor in nature.<br>PICC line related complications occurred in four patients (2%).<br>There were no permanent complications or deaths.	Antibiotic complications were: rash (11), diarrhoea (6), transient neutropenia (3), itchiness (2), fatigue (2), flushing (1), fever (1), black tongue (1), and elevated LFTs (1).<br>PICC related complications were: line thrombosis (3), and septicaemia (1).
71	Lopardo (Argentina)	Case-series (retrospective)	OP; SA; SN	48 patients (median age 55); 62% male	—	Complications occurred in 7/48 patients (15%; only one occurred while the patient was still receiving antibiotic treatment (heart failure).	Complications were: heart failure (3), and clinically significant emboli (4).
72	Mackintosh et al (UK)	Case-series (retrospective)	OP; SA; SN	198 patients (aged <40-89); 64% male	—	Twenty of 198 patients (10.1%) suffered an adverse reaction to initial IV therapy.	Adverse reactions were: rash (9), leukopenia or thrombocytopenia (4), vomiting (3), fever (2), tinnitus (1) and acute kidney injury (1).
77	Mauceri (USA)	RCT	SA	27 patients	—	Ten treatment-related adverse effects were reported in 27 patients, (two were considered severe).<br>Three patients were withdrawn from the study due to adverse events.	Adverse events were: rash (3), gastrointestinal symptoms (3), growth resistant organisms (3), and fever (1)
79	McMahon et al (Australia)	Observational	SN	40 patients (mean age 59 (M), 49 (F)); 75% male	—	Nine of 40 patients (23%) experienced a total of 11 adverse events. All were considered to be related to the antibiotic or to complications of venous access.<br>Three patients required hospital re-admission.	Adverse events were: rash (2), neutropenia (2), catheter-related (2), pseudomonas bacteraemia (1), fever (1), diarrhoea (1), nephrotoxicity (1), and chest pain during infusion (1).<br>Reasons for treatment failure re-admission were: dyspnoea and

SUPPLEMENTARY TABLES

ID	Author (country)	Study design	OPAT Model(s)	OPAT group(s)†	Comparator(s)†	Findings	Notes
						Three patients who were treatment failures were re-admitted.	pericardial effusion (2), and cellulitis around a cardiac surgery site (1).
80	Mohammadi et al (USA)	Case-series (retrospective)	SA; SN	190 patients (mean age 63); 98% male	—	Adverse events occurred in 12/90 patients (6.3%) during the review period. No emergence of a drug-resistant organism or C. difficile was observed<br>There were five peripherally inserted central catheter line adverse events, including three admissions (1.6%).<br>Eight deaths during OPAT were related to malignancies.	Adverse drug events included: neutropenia, diarrhoea, drug allergy rash, and nephrotoxicity.<br>Access-related events included line infection (4), re-admission (3), and emergency department visits for line-related events (2).
83	Montalto (Australia)	Case-series (retrospective)	SN (HHU)	133 patients (mean age 46); 52% male	—	Twenty-eight telephone calls were received from 22/133 patients (17%; range 1-3 calls). Thirteen were initiated by the patient or carer either anxious or asking about the condition or its management. Most required reassurance and follow-up.<br>Thirteen calls resulted in unscheduled staff callouts. One patient was re-admitted.<br>Five of 133 patients (3.8%) were returned to hospital during their HHU admission.	Reasons for calls were: nausea or vomiting (6), pain (3), allergic reaction (1), diarrhoea (1), fever (1), problems with venous cannula (2), and perception of worsening condition (2).<br>Results of callouts were: IV maxolon given (5), management of anaphylaxis (1), vasovagal episode during drug administration (1), discussion with GP/unit director and action planned (5), and re-admission (1).<br>Reasons for re-admission were: carer withdrawal (1), exploration of possible abscess (1), exploration of possible palmar space collection (1), worsening respiratory state in COPD (1), and non-resolving pneumonia (1).
84	Montalto et al (Australia)	Case-series (retrospective)	SN (HHU)	3423 treatment courses (patient age 0-80+); 55% male	—	Unexpected telephone calls were made by 607/3423 patients (18%).<br>There were 177 unexpected staff callouts (5%). Patients receiving antibiotics were more likely to have a staff call out than patients	Reasons for callout included: pump malfunction (46), venous access problems (30), pain (19), vomiting (17), fever (14), and anxiety (13). Patients who called were more likely to return to hospital than those who did not

SUPPLEMENTARY TABLES

ID	Author (country)	Study design	OPAT Model(s)	OPAT group(s)†	Comparator(s)†	Findings	Notes
						receiving non-antibiotic therapy (p<0.001).<br>A total of 143 episodes of care (4%) involved an unplanned return to hospital. Patients who received antibiotic therapy were more likely to require an unplanned return to hospital (p=0.001).<br>There were five patient deaths; two during HHU stay and three after return to hospital.	(14.5% v. 2.2%, p<0.001).<br>Reasons for re-admission included: lack of improvement (40), cardiac problem (22), and fever (19). No patient had an anaphylactoid drug reaction; four patients were returned for other drug reactions.
85	Morales & von Behren (Puerto Rico)	Case-series	SA	22 patients (mean age 33-41); 82% male	—	Six of 22 patients (27%) reported nine adverse events (all mild or moderate).<br>Four patients were withdrawn due to adverse events.<br>There were no deaths during the study period.	Unrelated adverse events were: headache (1), urethral disorder (1), and diarrhoea (1).<br>Reasons for withdrawal were: urticaria (1), probable allergic reaction (1), flushing (1), and fever, rash, diarrhoea, and urethral disorder (1).
86	Muldoon et al (Ireland)	Cross-sectional	Not specified	55 consultant physicians (15% clinical microbiologists)	—	61% of those reporting on re-admission estimated the 30-day re-admission rate for OPAT patients as being less than 10%, while the remainder estimated it at between 10 and 25%.	
87	Muldoon et al (USA)	Cross-sectional	Not specified	316 adult and paediatric infectious diseases physicians	—	Most respondents had witnessed at least one complication in the last year (243/274, 89%).<br>Adverse drug reaction was the most commonly reported (69%), followed by thrombosis (58%), C-Diff related disease (53%), line-related bacteraemia / exit site infection (51%), re-admission (43%), phlebitis (40%) and death (2%).	
89	Nathwani et al (UK)	Case-series	OP; SA	101 patients	—	Six of 101 patients experienced an adverse drug reaction (6%).<br>Eight patients had an unscheduled re-admission (8%).<br>Twelve patients (12%) had PICC line complications.	

SUPPLEMENTARY TABLES

ID	Author (country)	Study design	OPAT Model(s)	OPAT group(s)†	Comparator(s)†	Findings	Notes
90	Nathwani (UK)	Case-series	OP; SA; SN	125 patients	—	There were no complications associated with vascular devices.<br>One patient was re-admitted for OPAT failure (due to various logistical reasons)	
92	Pajaron et al (Spain)	Case-series (retrospective)	SA	45 patients (mean age 63); 76% male	—	Eight patients returned to hospital with complications and six were admitted (12.5%).<br>An additional 11 patients had less serious complications (24.4%).<br>There were three recurrences in 2/45 patients during the year following the initial episode (one patient had two recurrences).<br>No patients died in the course of treatment at home. Five patients (13%) died during the first year following discharge from hospital (two related to endocarditis and three to pre-existing advanced cancer).	Reasons for re-admission were: cardiac insufficiency (2), acute renal insufficiency with hypokalaemia (1), sepsis (1), medication-related anaphylactic reaction to allopurinol and acute secondary renal insufficiency (1), and hepatic-renal syndrome secondary to hepatocarcinoma (1).<br>Adverse events were: congestive cardiac insufficiency (4), angina (1), acute episode of previous chronic renal insufficiency (1), drug-side effect (4), and catheter related infection (3).
93	Parker et al (UK)	Observational	OP; SA	29 patients; 38 GP practices	—	Eight of 29 patients (28%) reported a problem.	Adverse events were: due to speed of injection or discomfort caused by IV access (6), and hypersensitivity type reaction (2).
94	Partridge et al (UK)	Case-series (retrospective)	OP; SA	34 patients (mean age 55); 79% male	—	Adverse events occurred in 12/36 episodes (33%), inc. one patient death (unrelated to OPAT).<br>Two patients with line infection required re-admission.<br>Three patients were re-admitted for non-OPAT-associated medical problems.	Adverse events included: line infection (4), split Hickman line (1), PICC line dressing allergy (1), pain in PICC arm (1), ototoxicity (1), renal toxicity (1), diarrhoea (1), and mycotic aneurysm of the spleen (1).
95	Patanwala et al (USA)	Decision tree analysis	SN	41 patients (mean age 53); 44% male	—	Nine of 41 patients (22%; all treatment failures) were re-admitted.	
96	Perez-Lopez et al (Spain)	Observational	SN	145 patients (mean age 68); 48% male	—	There were 23 adverse events; most common was phlebitis (n=21; 15%).<br>A total of 11/145 patients (8%) required hospitalisation.	There were no significant differences in the number of adverse events between older (n=14; 15%) and younger (n=9, 16%) patients (p=0.65).

SUPPLEMENTARY TABLES

ID	Author (country)	Study design	OPAT Model(s)	OPAT group(s)†	Comparator(s)†	Findings	Notes
						No patients died.<br>A further 22 patients were re-admitted during the 3-month follow-up period (four died while in hospital).	
99	Poretz (USA)	RCT	SA	238 patients (mean age 45-52); 57% male†	—	Seventy-two of 238 patients (30%) reported adverse experiences considered related to therapy (seven classified as severe, 25 moderate, 65 mild).	Adverse events included: phlebitis (13), rash (12), diarrhoea (8), maculopapular rash (4), hypoxia (3), allergic reaction (3), fever (3), headache (3), and nausea (3).
100	Poretz (USA)	RCT	SA	130 patients (mean age 45); 59% male†	—	Thirty-one of 130 patients (24%), reported adverse events (27 events were related to the study drug and four to the IV line).	Adverse events included: rash (7), diarrhoea (4), phlebitis (4), allergic reaction (3), and maculopapular rash (3).
104	Rodriguez-Cerrillo et al (Spain)	Case-series	SN (HHU)	24 patients (mean age 73)	—	Three of 24 patients contacted the Unit for minor symptoms or doubts about treatment.<br>One patient required an unscheduled visit by HHU staff (vomiting).<br>No patient had an unexpected return to hospital.	
105	Rodriguez-Cerrillo et al (Spain)	Case-series	SN (HHU)	25 patients (mean age 59); 48% male	—	One of 25 patients (4%) made an unexpected telephone call for diarrhoea (after starting oral antibiotics).<br>There were no urgent visits by HHU staff, hospital re-admissions during HHU treatment, or admissions in the month following discharge.	
106	Seaton & MacConnachie (UK)	Case-series (retrospective)	SA; Other (unspecified)	19 patients (mean age 55); 53% male	—	Eighteen patients (95%) survived >6-months after hospital discharge.<br>There were two adverse events and one patient death. No further events were noted in the OPAT setting with therapy up to 128 days.<br>There were no unplanned re-admissions.	Adverse events were: above knee amputation for osteomyelitis/septic arthritis (1), and reversible myotoxicity (1).

SUPPLEMENTARY TABLES

ID	Author (country)	Study design	OPAT Model(s)	OPAT group(s)†	Comparator(s)†	Findings	Notes
110	Seaton et al (UK)	Observational (retrospective)	OP	963 patients (median age 48); 59% male	—	Significant adverse events were observed in 68/963 patients (7.1%). Admission or re-admission occurred in 58/963 cases (6%).	Adverse events included: drug-related rash requiring a change in therapy (25), GI side effects, particularly diarrhoea (16), and abnormal liver function (11). Three patients had severe allergy or anaphylaxis. Reasons for admission included: treatment failure with progression of infection or lack of response (19), and other medical complication (15).
112	Seetoh et al (Singapore)	Cohort	OP; SA; SN	2,229 patients (median age 56); 64% male	—	Re-admission was needed in 281 episodes (12.6%), and 74 patients (3.3%) ended treatment early. Re-admission rate due to clinical deterioration was 9.0%, and occurred a median of 11 days (IQR 5-23 days) after beginning treatment. SN OPAT episodes were more than twice as likely to deteriorate as OP episodes (20% vs. 7.9%; aHR 2.5, 95% CI, 1.7-3.8; p<0.001). There was no significant difference compared with self-administration (20% vs. 9.6% aHR 0.9, 95% CI, 0.6-1.3; p=0.572). Of those who did not complete treatment as planned (i.e. those re-admitted or ceasing early without re-admission): 60 (2.7%) had adverse drug reactions, 16 (0.7%) had PICC line complications, four (0.2%) absconded, and two (0.1%) died suddenly.	Reasons for re-admission included: elective procedures (42), and clinical deterioration (201).
113	Sims et al (UK)	Case-series (retrospective)	OP	14 patients (mean age 63); 71% male	—	Four patients (29%) required re-admission, two for medical conditions unrelated to OPAT (UTI and hematemesis), and two for deterioration in the clinical status of their infected arthroplasty. There were no line complications. There was one unrelated patient	

SUPPLEMENTARY TABLES

ID	Author (country)	Study design	OPAT Model(s)	OPAT group(s)†	Comparator(s)†	Findings	Notes
						death.	
114	Smego et al (Pakistan)	Case-series	SA	316 patients (mean age 45); 53% male	—	Medication-related adverse effects were few (14/316), generally mild, and resolved with drug dosage adjustment or discontinuation of use.<br>No unscheduled clinic or emergency room visits.	Side effects were: renal impairment (4), skin rash (3), diarrhoea (3), leukopenia (2), hepatitis (1), and drug fever (1).<br>Minor thrombophlebitis was the only catheter-related effect (3).
115	South (UK)	Case-series (retrospective)	OP; SA; Nurse (unspecified)	57 patients (mean age 41); 77% male	—	There were two adverse events among the 69 episodes treated (2.9%).	Adverse events were: shaking following IV bolus administration (1), and truncal rash (1).
117	Subedi et al (Australia)	Case-series	SA; SN	144 patients (median age 55); 74% male	—	There were 11 (7%) drug-related adverse events, one resulting in re-admission (6.9%). The rest were managed with symptomatic treatment, earlier cessation, or a change of antibiotic.<br>Line-related events occurred in five patients (3%) or 1.4/ 1000 catheter-days.<br>OPAT related re-admission occurred in nine patients (6%) within 28 days of cessation of IV antimicrobials.	Reasons for re-admission were: drug fever (1), re-insertion of PICC line (1), clinical deterioration requiring source control or surgical debridement (6), and change of antimicrobial therapy (1),<br>Adverse drug events included: rash (6), biliary lithiasis (1), drug fever (1), and acute kidney injury (2)<br>Access-related events were: infection (2), lymphatic leakage (2), and line fell out (1).
118	Talcott et al (USA)	Observational (pilot)	SA; SN	30 patients (median age 38); 43% male	—	Five patients were re-admitted for recurrent or prolonged fever (17%). Four patients were re-admitted for medical complications (13%).<br>There were no lasting complications or deaths.	None of the 27 medically eligible patients who did not enter the study had complications. The complication rate for all medically eligible patients was 4/57 (7%).
121	Theocharis et al (Greece)	Case-series (retrospective)	SN	91 patients (mean age 85); 31% male	—	Mortality was 27.5% (25/91; (those who were not cured).<br>Thirteen patients were admitted to hospital (14%); three died.	

SUPPLEMENTARY TABLES

ID	Author (country)	Study design	OPAT Model(s)	OPAT group(s)†	Comparator(s)†	Findings	Notes
123	Tice (USA)	Case-series	OP; SA; SN	538 patients (mean age 45); 52% male	—	Medication was changed due to an adverse effect in 16 cases. Forty-two of 538 patients (7.8%) were hospitalised after starting OPAT. Only one patient (drug rash) was hospitalised due to the OPAT program or medications. One death occurred during treatment (patient with advanced AIDS). Three patients were removed from the program because of failure to comply.	Adverse events were: rash (11), leukopenia (1), neuromuscular disturbance (1), renal toxicity (1), laryngeal oedema (1), and intractable 'red man' syndrome (1). Reasons for admission were: surgery unable to take place at outpatient centre (20), poor clinical response (5), inadequate home care (3), neurological problem (3), cardiac disease (2), bleeding (2), chest disease (2), vomiting (2), leukopenia and fever (1), reimbursement problem (1), and drug rash (1).
124	Tice (USA)	Observational (retrospective)	OP; SA; SN; Infusion centre.	500 patients	—	A minority of patients (12%) discontinued treatment prematurely, most commonly for adverse drug reaction (5.2%). Discontinuation related to clinical failure in around 1% of cases, and to death in 0.4%.	
125	Tice et al (USA)	Observational (retrospective)	OP; SA; Office based; Pharmacy based	971 patients	—	One hundred and nineteen of 1053 (11%) episodes were complicated by adverse events. Early discontinuation of therapy occurred in 6.4% of episodes (most commonly for rash and fever). One hundred and forty-six venous access device-related adverse events were observed.	Adverse events were: rash, renal toxic reaction, fever, nausea and vomiting, urticaria, diarrhoea, anaphylactic reaction, leukopenia, and vestibular toxic reaction. Rash was common (3%); as renal toxicity (1.5%) and fever (1.3%). Access events included: phlebitis (42), thrombosis (21), local infection (17), leakage (12), and bacteraemia (9).
126	Upton et al (New Zealand)	Case-series	SA	100 patients (mean age 51); 59% male	—	Thirty-five of 100 patients (35%) experienced complications during treatment. Twenty-two patients had central line complications (incidence of 5.3 per 1000 central line days). Twelve occurred after discharge, and five resulted in re-admission. Twenty two patients had adverse	Line complications were: line blockage (8), infection (5), DVT (4), phlebitis (3), failed line insertion (1), and line intolerance (1). Adverse drug reactions were: rash (8), GI symptoms (7), chest discomfort (6), abnormal LFTs (5), leukopenia (4), and nephrotoxicity (3).

SUPPLEMENTARY TABLES

ID	Author (country)	Study design	OPAT Model(s)	OPAT group(s)†	Comparator(s)†	Findings	Notes
						drug reactions; three resulted in re-admission. One patient died two days after completing treatment (ruptured iliac aneurysm).	Reasons for re-admission: PICC line infection (3), PICC line re-insertion (2), anxiety (1), nausea (1), and allergic reaction (1).
127	Walton et al (Australia)	Case-series	SN	35 patients (mean age 50); 71% male	—	Four patients stopped treatment prematurely. No patients developed penicillin associated neutropenia. Four patients had problems with PICC venous access; three required replacement.	Reasons for discontinuation included: probable drug related febrile hypersensitivity reaction (1), PICC-associated septicaemic shock (1), and weakness and focal seizures (1). Reasons for PICC replacement were: catheter blockage (1), and exit site infection (2).
128	White et al (Australia)	Case-series (retrospective)	OP; SN; GN	55 patients (mean age 50); 65% male	—	Seven of 55 patients (13%) had adverse drug events, with IV antibiotics responsible for events in three patients. There were no complications related to IV access.	Antibiotic events were: rash (1), thrombocytopenia (1), and deranged liver enzymes (1).
129	White et al (UK)	Case-series (retrospective)	SA	72 patients (mean age 42); 42% male	—	A total of 39 drug reactions were documented in 29/72 patients (40%). Therapy was discontinued in 10 patients (14%). One patient was admitted to hospital (line infection with sepsis).	Drug reactions were: neutropenia (13), mild liver function derangement (8), line infection (3), rash (3), allergic reaction (2), diarrhoea (4), headache (1), nausea (1), fatigue (1), oral thrush (1), shingles (1) and dyspepsia (1). Reasons for discontinuation were: neutropenia (4), drug rash (3), allergic reaction (1), and line infection with severe sepsis (1).
130	Williams (USA)	Observational (retrospective)	SA	1045 patients (mean age 38); 58% male	—	Drug related side-effects were relatively infrequent (2-5%). Venous access issues were common (375/1500; 25%) Rate of re-admission was 5-6% (75-90 patients). No deaths were directly attributable to home therapy, and overall morbidity was low.	Drug side-effects were: nephrotoxicity (75), skin rash (75), and leukopenia (30). Access issues included: infiltration of IV, and phlebitis of a peripheral cannula. Reasons for re-admission included: antibiotic failure, therapy change, and surgery.

SUPPLEMENTARY TABLES

ID	Author (country)	Study design	OPAT Model(s)	OPAT group(s)†	Comparator(s)†	Findings	Notes
133	Wynn et al (USA)	Observational (retrospective)	Physician-based program; Home health organisation; Hospital-based program (no other details)	1252 patients (mean age 52); 65% male	—	A total of 66 adverse drug reactions were reported in 1,515 patients (4%).<br>There were 106 vascular access complications in 1,232 patients (9%).	Adverse drug reactions were: rash (26), diarrhoea (8), anaphylaxis (6), leukopenia (6), renal toxicity (6), fever (5), nausea/vomiting (5), and unspecified (4).<br>Access complications included: phlebitis (31), leakage (19), thrombosis (10), local reaction (9), and bacteraemia (4).
134	Yadlapalli et al (USA)	Case-series (retrospective)	Not specified	58 patients (mean age 60); 83% male	—	During 12-months follow-up, 10 patients (17.2%) required re-admission for further treatment.	
135	Yan et al (UK)	Case-series (retrospective)	OP; GN	140 patients (aged 17-89); 64% male	—	Five of 140 patients (3.6%) required hospital admission due to an inadequate response.<br>Three patients (2.1%) developed a rash.<br>There were no reported complications related to use of IV catheters (some patients inadvertently removed their cannula at home).	Three of the patients admitted to hospital required referral to the orthopaedic/surgical team due to abscess formation and tracking of cellulitis.

OP: outpatient; SA: self-administered; SN: specialist nurse; GN: General nurse.

† Age and gender details relate only to those patients who were clinically evaluable or provided questionnaire data.

For peer review only - http://bmjopen.bmj.com/site/about/guidelines.xhtml

TABLE S3 Cost-effectiveness of outpatient parenteral antibiotic treatment

ID	Author (country)	Study design	OPAT Model(s)	OPAT group(s)	Comparator(s)	Findings
60	Krauth et al (Worldwide)	Literature review	Not specified	11 studies	Inpatient therapy	Studies predominantly concluded that home IV therapy would lead to significant cost reductions, from the societal perspective as well as the third-party payer perspective. In five studies, inpatient therapy was 1.8 to 3.3 times as expensive as home care; three studies that considered incremental costs revealed potentially significant cost savings with home therapy. Two of the remaining three studies reported divergent results, primarily related to pharmaceutical pricing and duration of therapy.<br>Only one study included loss of working days and its monetary valuation, and found that the indirect costs per course of inpatient treatment totalled DM868 compared with DM476 for home treatment.
95	Patanwala et al (USA)	Decision tree analysis	SN	41 patients (mean age 53); 44% male	Switch therapy (IV-oral)<br>Oral therapy	Oral treatment during hospitalisation and after discharge had superior effectiveness and lower cost than the Switch and OPAT scenarios in the base-case, incremental cost-effectiveness analysis ($8923, 0.87 vs. $11479, 0.78 vs. $12481, 0.71, respectively). The cost variable that was expected to have the largest impact on the model was cost per day in hospital. Varying the baseline cost per day in hospital (range $463-$2500) did not affect the dominance of the Oral option.<br>The Switch option would be the cost-effective choice if the length of hospitalisation was <6 days or if the probability of cure with the Oral option was ≤0.72.
119	Teufel et al (Canada)	Decision tree analysis	SN	77 patients	Early discharge with oral therapy<br>Inpatient therapy<br>Oral outpatient therapy	Oral outpatient treatment yielded an average QAFNE of 0.65, inferior to OPAT (0.72) and Early discharge (0.66), but superior to hospital treatment (0.62). Oral outpatient treatment was cost saving, but less effective than outpatient IV (ICER of $10186 per QAFNE); Early discharge and Hospital treatment were dominated strategies.<br>Early discharge ($9265) and inpatient therapy ($21866) were less cost effective than either Oral or IV outpatient therapies (OPAT $5810; Oral $5338). At a WTP threshold of $4000 per QAFNE, Oral treatment was cost-effective in 54% of the simulations, and OPAT in 38%. Early discharge was cost effective in 8% and traditional hospital management in less than 1%. Beyond certain cost thresholds, dominance changed from the Oral to OPAT strategy, but there was no constellation when Early discharge or inpatient treatment became superior.

SUPPLEMENTARY TABLES

ID	Author (country)	Study design	OPAT Model(s)	OPAT group(s)	Comparator(s)	Findings
122	Thornton et al (UK)	Observational (retrospective)	Not specified	Home therapy; 47 patients (mean age 26); 36% male	Combined therapy; 18 patients (mean age 25); 61% male<br>Inpatient therapy; 51 patients (mean age 26); 59% male	When effective treatment was classed as ≤0% decline in FEV_1, mean ICER was £46,098 (-£374,044 to £362,472); when ≤2% was used, mean ICER was £73,885 (£1236 to £269,023). These are the amounts that must be spent to obtain one more year of effective treatment with hospital-based care for one patient. The cost-effectiveness planes indicated increased effectiveness and increased cost for Hospital treatment compared with Home treatment. There was a slight possibility that Hospital treatment may be less effective and more expensive than Home treatment at ≤0% decline; when ≤2% decline is used this was not the case.<br>The cost-effectiveness acceptability curve showed that if a decision maker was willing to pay up to £262,500 for one extra patient with an FEV1 decline of ≤2% over 1 year, there was a 95% probability that hospital care would be cost effective. If a decline of ≤0% was used, the probability that hospital care would be cost effective never reached 95% (even if the decision maker was willing to pay £10 million for one extra patient).
138	You et al (China)	Decision tree analysis	SA; SN	All patients (no numbers provided)	Oral therapy (early discharge)<br>Inpatient therapy	The base-case analysis showed that OPAT ($14,470 per patient) was the least costly alternative, followed by Oral ($17,877) and inpatient treatment ($19,980). Results were sensitive to variation of the success rates of the IV and oral drugs: 1) if the IV success rate was less than 55%, Oral treatment would become the least costly option; if the success rate was higher than 80%, both OPAT and hospital IV treatment would be less costly; 2) if the Oral treatment success rate was higher than 80%, this would be the least costly option; if the success rate was less than 55%, this would be the most costly. Throughout the ranges of all variables, OPAT remained less costly than hospital treatment.<br>OPAT was less costly than Oral and hospital treatment in 64% and 100% of the simulations, with mean savings of $2,313 (95% CI 2,188–2,438) and $4,881 (95% CI 4,869–4,893) per patient, respectively.

OP: outpatient; SA: self-administered; SN: specialist nurse; GN: General nurse; ICER: incremental cost-effectiveness ratio; QAFNE: quality adjusted febrile neutropenia episode

† Age and gender details relate only to those patients who were clinically evaluable or provided questionnaire data.

SUPPLEMENTARY TABLES

TABLE S4 Patient acceptability of outpatient parenteral antibiotic treatment

ID	Author (country)	Method	OPAT Model(s)	OPAT group(s)	Comparator(s)	Findings
**COMPARATOR STUDIES**						
30	Corwin et al (New Zealand)	Questionnaire survey	GN	Home therapy; 98 patients (mean age 55); 62% male	Inpatient therapy; 96 patients (mean age 48); 73% male	Most patients in both treatment groups were satisfied with the care received (Home 96%; Hospital 96%). Only 5% of Home patients would prefer hospital treatment, while 35% of Hospital patients would prefer home treatment (p<0.001). Nine percent of the Home group reported no preference for location compared with 34% of the Hospital group.
32	Dall et al (USA)	Telephone survey	SN	Home therapy; 92 pneumonia patients (97% aged >50 years); 44% male / 64 cellulitis patients (mean age 51); 70% male	Inpatient therapy; 10728 pneumonia patients (83% aged >50 years); 51% male / cellulitis patients (no numbers provided)	Overall satisfaction with the program was extremely high, with >90% of patients responding positively to the telephone survey. The ability to have medical care at home was consistently cited as a benefit.
44	Fernandez-Aviles et al (Spain)	Questionnaire survey	SN	Home therapy; 50 patients (mean age 47); 62% male	Inpatient therapy; 50 patients (median age 50); 54% male	All 30 of the patients and caregivers surveyed felt safe at home. Most patients (97%) indicated that they would choose to receive treatment at home again, and that they would recommend the procedure to other patients. The main advantages reported were quiet and increased home comfort (67%), familiar environment (27%), free choice of activity (27%), free choice of food (20%), and increased privacy (13%). Reported disadvantages (10 patients) including anxiety and fatigue (20%), cost of local hotel or apartment (7%), and caregiver anxiety (7%).
103	Richards et al (New Zealand)	Questionnaire survey	GN	Home therapy; 24 patients (mean age 50); 54% male	Inpatient therapy; 25 patients (mean age 50); 52% male	All Home group patients were *very happy* with their care, compared with 60% of Hospital patients (p=0.001). Similarly, most patients were happy with the location of their care, but the Home group were happier; 92% were *very happy* with the location, compared with 32% in the Hospital group (p<0.001).
**OPAT ONLY STUDIES**						
11	Al Ansari et al (Bahrain)	Questionnaire survey	OP	101 patients; 57% male	—	Survey response was high (96.9%). Patients were highly satisfied with the OPAT service (mean rating 4.41, SD 0.31).
16	Bamford et al (UK)	Interviews	Not specified	12 interviewees (mean age 62-63); 58% male	—	Most interviewees initially preferred oral administration. The most frequently cited disadvantages of IV administration were pain or discomfort and the inconvenience related to movement, followed by problems finding or maintaining the cannula and fear of needles.<br>Most thought the main advantage was that it would work quicker; other advantages mentioned were that it would be "*more effective*", "*less hassle*", "*it's correct and you don't forget*"

SUPPLEMENTARY TABLES

ID	Author (country)	Method	OPAT Model(s)	OPAT group(s)	Comparator(s)	Findings
						and "*it's probably gentler on the side effects on the stomach*". When specifically asked about IV antibiotics at home, four interviewees were clear that they would not be happy with the idea, three because of delivery issues, "*I just don't believe a district nurse could do it*", and one preferred oral therapy. Four others were happy with the idea, or had concerns about equipment/sterility but would be happy if management of the IV could be delivered at home, "*if there was no fear and it could be done sterilely*". When asked about management of future infections, 75% of those who gave an opinion preferred oral antibiotics. Of the two patients who preferred the IV route, one could not swallow tablets, one thought IV antibiotics were better.
22	Bernard et al (France)	Not specified	SA; SN;	39 patients (mean age 44); 64% male	—	None of the patients complained about having to return frequently for consultation with their attending physician, and they accepted this inconvenience in order to remain outside the hospital.
26	Chambers et al (New Zealand)	Questionnaire survey	SA	100 eligible patients (mean age 63)	—	Home IV therapy was rated as *good* or *very good* by 97% of respondents. Two patients (2%) reported that they would not have it again. When patients were asked to comment freely, 91% of the replies were extremely positive (the most common reason being a preference for home over hospital). Most patients rated the experience of having a PICC or midline inserted as very good (60%) or good (35%); 28% reported some problem with the IV catheter (leakage, blockage, discomfort, inconvenience, arm swelling, skin reaction to dressing, connector becoming undone). Most respondents with a pump device rated this as *good* or *very good* (88%); 8% reported discomfort or restricted movement, and 3% reported kinking. Twelve patients (14%) self-administered antibiotics and all would do so again. Of those who did not, 27% would have liked to have done so, the main reason given being freedom from appointments with nurses.
28	Chapman et al (UK)	Questionnaire survey	SA; Infusion centre	334 treatment courses (mean patient age 46); 59% male	—	Of 276 patients completing questionnaires since the OPAT service was established, 98.6% rated the service as *very good* or *excellent*; 99.6% reported that they would choose the OPAT service again.
31	Cox et al (USA)	Unscheduled calls or visits	SA	205 patients (mean age 59); 99% male	—	The younger group was significantly more likely than the older group to perform infusions unaided (41% vs 20%; P<0.001). Older patients were significantly more likely than younger patients to have an urgent care visit with a problem or question about the catheter or infusion (31.4 visits/1000 home IV days vs. 14.3 visits/1000 home IV days; p<0.001). Similarly, there

SUPPLEMENTARY TABLES

ID	Author (country)	Method	OPAT Model(s)	OPAT group(s)	Comparator(s)	Findings
						were more phone calls to the ID pharmacist from the older group (14.3/1000 home IV days vs. 8.57/1000 home IV days; p=0.04).
42	Esposito et al (Italy)	Not specified	OP; SA; Visit by doctor or nurse (also care facility)	239 patients (aged 11-80+); 62% male	—	Satisfaction with the OPAT regimen was high, with 91% of patients giving favourable feedback.
45	Goodwin et al (Canada)	Questionnaire survey	SN; GN	2405 patients (mean age 46); 55% male	—	Most of the 424 responding patients were *very satisfied* or *completely satisfied* with home therapy (61%); 6% were *not very satisfied*, and 5% were *totally dissatisfied*. Most of the satisfaction related to resuming daily activities such as work or school, and most dissatisfaction to costs incurred.
48	Grayson et al (Australia)	Not specified	GN	20 patients (mean age 58)	—	All patients reported a strong preference for home IV treatment over inpatient therapy, with many describing a sense of improved self-esteem, "ownership" of their illness, and involvement on therapy.
51	Hindes et al (USA)	Questionnaire survey; personal communication	SA	48 patients (mean age 65)	—	Surveys and personal communications demonstrated a high degree of satisfaction with the care provided, and strong endorsement of the home therapy provided as an alternative to inpatient care (no other details provided).
52	Hitchcock et al (UK)	Questionnaire survey	OP; SA; GN; Private home healthcare company	273 patients (mean age 60); 54% male	—	Most of the 84 survey respondents felt that the service met their expectations and would be happy to receive this form of treatment again should the situation arise (96%). All were happy with the support they received from the OPAT team during treatment; 95% felt that their quality of life during the period of infection had been improved by outpatient management (two of the patients who did not think this suffered an adverse drug event).<br>Most respondents (83%) thought that the service could not be improved. The most frequent comments from those who felt improvements could be made related to patient transport problems transport (6/14); two patients thought that communication between consultants, other staff and patients could be improved, three that they had waited too long, and one patient suggested referrals could be made directly by their GP.
55	Huminer et al (Israel)	Not specified	SA; SN (also nursing home; kibbutz)	37 patients (mean age 64); 57% male	—	Most patients (95%) reported a high level of satisfaction with this type of therapy (no other details provided).

SUPPLEMENTARY TABLES

ID	Author (country)	Method	OPAT Model(s)	OPAT group(s)	Comparator(s)	Findings
56	Johansson et al (Sweden)	Questionnaire survey; VAS	SA	11 patients (mean age 51); 73% male	—	All patients reported that OPAT was of great value (median VAS rating 97mm). Prior expectations of OPAT included peace and quiet, greater freedom and more time to do other things, the home environment being important for well-being, and to being an everyday part of the family. The 10 patients who responded after OPAT said that their expectations had been met.<br>All patients said they would prefer to have OPAT again during any subsequent IV antibiotic therapy, and all agreed there were many advantages to having treatment at home. Nine out of ten patients never felt worried during administration of the drug (although they sometimes worried about the direction of their underlying condition). Nine out of ten patients were fully satisfied with the education (inc. potential complications).<br>Specific advantages reported by patients included not having to go to hospital every day for therapy, more freedom, more time to do household duties and for leisure, the feeling of having control, freedom and flexibility, making it possible to be a part of daily issues at home, and being at home with your family. No examples of disadvantages were given.
57	Kayley et al (UK)	Not specified	SA; GN; Doctor administered	67 patients	—	Patients said they were happy to receive their treatment at home (anecdotal only – no formal survey)
59	Kieran et al (Ireland)	Telephone survey	SA; Nurses attached to commercial company	56 patients (median age 50); 57% male	—	All 12 patients surveyed reported that they were very happy to complete their course of antibiotics at home rather than remaining in hospital. Reasons given were preference of home to hospital (44%), the ability to resume activities of daily living (11%) or both (44%).<br>All patients were satisfied with the service received, none felt that they had inadequate training, and none felt that their infection took longer to be treated because they received OPAT. All stated that they would prefer to be treated at home if a similar situation arose in the future. Almost three quarters (70%) would be happy to be followed up by phone or internet with less frequent outpatient reviews, providing that care of the IV access device could be done in the community.
67	Lehoux (Canada)	Interviews	SA	6 interviewees (mean age 64); 50% male	—	User acceptance of home IV treatment was shaped by different forms of anxiety, such as protecting the catheter site to avoid potential infection or the possibility of the catheter becoming dislodged. The pump alarm system could go off too easily, and false alarms frequently disturbed sleep and were initially perceived as very stressful (over time they became a "normal" disturbance).

For peer review only - http://bmjopen.bmj.com/site/about/guidelines.xhtml

SUPPLEMENTARY TABLES

ID	Author (country)	Method	OPAT Model(s)	OPAT group(s)	Comparator(s)	Findings
						Professional and social life was slightly limited, and patients were generally passive or submissive about this, "*you're always a slave to it, having to carry it everywhere*". Carers sometimes curtailed social activities because they felt needed by the patient.<br>User-acceptance was closely linked to competence. Older patients did not feel comfortable with the electronic components of the pump, and the manual dexterity required to properly manipulate it could be problematic for older people, "*if my eyes were okay, I'd have been able to do it. But I was frightened of not doing it properly, of not seeing the needle, which is so tiny*" (IV carer). Direct observation showed patients who were unable to read messages on the digital screen due to poor eyesight, limited English, or illiteracy. They relied on memory or made informed guesses.
73	Marra et al (Canada)	Questionnaire survey	SA; SN	91 patients (mean age 56); 69% male	—	The majority of the 91 enrolled patients (96%) indicated a treatment location preference; 89% preferred home, while the remainder preferred hospital. Most patients (82%) gave an interpretable response regarding willingness to pay (WTP) for treatment in their preferred location. Of these patients, 90% preferred home with a median WTP of $490 CDN, and the remainder preferred hospital with a median WTP of $500 CDN (not statistically significant). Total WTP for the patients who preferred hospital treatment was $7,859 versus $60,712 for the patients who preferred home treatment. Those who stated preferences were willing to pay more than those who did not (p<0.0001).
74	Martel (France)	Questionnaire survey	SA	116 patients (mean age 36 (M), 41 (F)); 76% male	—	Patients who received Home treatment (n=33) had a significantly higher internal locus of control, while patients who preferred to continue their treatment in hospital (n=17) had a higher external locus of control. The main reasons for accepting Home treatment were socio-familial, wanting to carry on normal activities and to have higher autonomy or freedom. The hospital group had low confidence in their own ability.<br>The majority of Home group patients (89%) would use home treatment again if the cost was paid by a third party; 32% agreed to participate again even if they were responsible for the cost of antibiotics.
82	Montalto (Australia)	Telephone interviews	SN (HHU)	67 patients (aged 16-70+); 49% male / 65 carers (aged 16-70+); 29% male	—	Preference for the convenience and comfort of home was the most commonly cited reason for agreeing to enter the HHU (67%). More than a quarter of patients (28%) mentioned avoidance of hospitalisation, and a smaller number (13%) felt that it saved a bed for another person; three patients believed that they were not ill enough to warrant a bed. Seven patients (10%) felt that their choice was constrained as no inpatient beds

SUPPLEMENTARY TABLES

ID	Author (country)	Method	OPAT Model(s)	OPAT group(s)	Comparator(s)	Findings
						were available at the time. Almost all (97%) would use the service again if the opportunity arose. Benefits of a home environment were the most commonly perceived advantages of HHU care (62%), with patients reporting feeling happier, more comfortable at home, greater personal freedom, companionship, and less disruption for themselves and their family. Positive characteristics of the HHU were mentioned by 60% of patients; only two mentioned disadvantages of HHU care. Seven patients recalled feeling worried during their stay, but when asked whether they felt confident during their time in the HHU, all 67 patients, including those who felt worried, responded positively. The most common reason given for this was the 24-hour contact numbers for emergency backup.
89	Nathwani et al (UK)	Questionnaire survey	OP; SA	101 patients	—	Most patients (79%) were happy with all aspects of the care received; 96% reported that the service improved their quality of life, and 89% that this form of treatment met or exceeded their expectations. For 93% of patients it was preferable to inpatient care, and 93% would prefer this model in the future if the need arose. Most patients' family/carers were satisfied with the service (93%).
90	Nathwani (UK)	Not specified	OP; SA; SN	125 patients	—	Patient satisfaction with OPAT was very high (no other details provided.)
93	Parker et al (UK)	Questionnaire survey; focus group; call to practices	OP; SA	29 patients; 38 GP practices	—	Most patients (79%) reported the freedom of being at home as the main reason for taking part. Other reasons included less family disruption (31%), and increased social contact (21%). Of 26 patients who completed the end of study questionnaire, 92% were very much in favour of non-inpatient treatment and would repeat this form of therapy. Questionnaire results were supported by the focus group; all patients would repeat this form of therapy and felt that treatment outside of hospital improved their quality of life.
97	Pilling & Walley (UK)	Interviews	SA; Commercial home care organisation	11 CF patients (aged 17-30) / 14 parents (children aged 6-16)	—	Most of the respondents (92%) had considerable experience of home therapy, having received home IV antibiotics for ≥1 year. All preferred to be treatment at home rather than in hospital provided they were reasonably well. Most (96%) were satisfied with the quality and comprehensiveness of training, although one respondent felt that more information should have been provided on potential adverse reactions, and that the quality of homecare could be improved by the provision of refresher courses on the management of infusion devices. Support arrangements were thought to be good, and all knew how to

SUPPLEMENTARY TABLES

ID	Author (country)	Method	OPAT Model(s)	OPAT group(s)	Comparator(s)	Findings
						obtain advice if needed. Fifteen respondents (60%) had experienced one or more problems with their therapy (drug effervescence, painful administration, line blockage, failure of ambulatory infusion device, storage of drugs or equipment, getting rid of used equipment etc.). Most were minor, but some such as line blockages required a visit to hospital or a home visit from the nurse. Patients who self-constituted their antibiotics reported difficulties in obtaining adequate supplies of ancillary items (arbitrary quantities were provided and further supplies were difficult to obtain). They also reported problems with storage of drugs and equipment, and disposal of used equipment, particularly needles. With this exception however, all respondents were satisfied with the support they received from hospital and had no suggestions for improvement.
104	Rodriguez-Cerrillo et al (Spain)	Questionnaire survey	SN (HHU)	24 patients (mean age 73)	—	95% of patients treated expressed their satisfaction with this type of treatment.
105	Rodriguez-Cerrillo et al (Spain)	Questionnaire survey	SN (HHU)	25 patients (mean age 59); 48% male	—	All patients expressed their satisfaction with treatment at home (no other details provided).
108	Seaton et al (UK)	Questionnaire survey	Not specified	205 eligible patients (mean age 49); 51% male†	—	Most of the 183 responding patients (84%) thought that OPAT would be an acceptable alternative to inpatient therapy. Only three of the 29 patients who disagreed gave explanations (severe arthritis, fear of the IV device and disagreement in principle). This group was significantly older than those who were willing to have IV therapy at home ($\bar{x}$ 64y vs. 46y; $p<0.001$). Acceptability was not influenced by gender. All groups of patients (by condition) were equally agreeable to the prospect of outpatient therapy. Of the 95 patients who received IV antibiotic therapy, 87% thought that OPAT was an acceptable alternative to inpatient therapy, and 72% had a caregiver who would be willing to administer the antibiotic at home.
113	Sims et al (UK)	Telephone survey	OP	14 patients (mean age 63); 71% male	—	One patient died of unrelated causes prior to questionnaire completion. All of the 13 patients contacted were satisfied with the OPAT service, thought that it was more convenient than an inpatient stay, and should the same problem recur would undergo OPAT again. All reported they would recommend the service to a friend under similar circumstances.

SUPPLEMENTARY TABLES

ID	Author (country)	Method	OPAT Model(s)	OPAT group(s)	Comparator(s)	Findings
118	Talcott et al (USA)	Likert scales	SA; SN	30 patients (median age 38); 43% male	—	Before home therapy, all 30 patients overwhelmingly preferred home to hospital therapy, and had little fear of isolation from their physician if a serious problem occurred. These convictions were unchanged after completion of home therapy.<br>The report of family comfort with home care increased after treatment: support for the statement, "My family would rather have me near them than be in the hospital when something happens like this (developing fever and neutropenia)" increased from 42% agreement to 70% agreement.<br>There was some evidence that patients felt slightly more isolated at home than in the hospital: agreement with the statement "I feel uneasy about calling my doctor from home with a complaint that worries me a little, for fear that I will be disturbing him or her with such a minor problem" increased from 10% to 24%.
120	Teufel et al (Canada)	Interviews; VAS	Not specified	78 participants (mean age 54); 41% male	—	Most respondents (75%) preferred some form of outpatient management. Home oral treatment was most commonly ranked first (36%), but 21% of respondents preferred Home IV ad 18% preferred Early discharge. All three outpatient strategies were associated with higher mean VAS scores than (5.3 vs. 5.7 for Early discharge, 6.1 for Home oral, and 6.2 for Home IV).<br>There was no significant difference between the outpatient strategies in relation to how much remaining life patients would be willing to give up to avoid inpatient care; $\bar{x}$ 9.1 weeks for Early discharge, 9.6 weeks for Home IV, and 9.3 weeks for Home oral. On average, patients would give up <1% of their remaining lifetime to avoid inpatient care.<br>Findings were similar for willingness to pay ($\bar{x}$ $282 for Early discharge, $327 for Home IV, and £255 for Home oral). The maximum WTP to receive IV treatment at home instead of in hospital was $4,500 (max. $2,000 for Early discharge and $1,500 for Home oral).
123	Tice (USA)	Questionnaire survey	OP; SA; SN	538 patients (mean age 45); 52% male	—	Most of the 294 responders (89%) reported that they *definitely would* have outpatient therapy instead of being hospitalised if the need arose again, and 10% said they *probably would* have outpatient therapy again. Of the four remaining patients, two said they *probably would not*, and two said they *definitely would not*.
124	Tice (USA)	Questionnaire survey	OP; SA; SN; Infusion centre.	500 patients	—	Almost all of the respondents (99%) reported that, should they require parenteral antibiotic therapy again, they would *definitely* or *probably* choose to be treated at home; <1% said they would prefer hospitalisation.

SUPPLEMENTARY TABLES

ID	Author (country)	Method	OPAT Model(s)	OPAT group(s)	Comparator(s)	Findings
135	Yan et al (UK)	Questionnaire survey	OP; GN	140 patients (aged 17-89); 64% male	—	The survey was completed following an OPAT pilot scheme; 88% of patients (no numbers given) were pleased with the service as it enabled earlier discharge, even if this meant daily trips back to hospital.

OP: outpatient; SA: self-administered; SN: specialist nurse; GN: General nurse.

† Age and gender details relate only to those patients who were clinically evaluable or provided questionnaire data.

For peer review only

SUPPLEMENTARY TABLES

TABLE S5 Provider acceptability of outpatient parenteral antibiotic treatment

Ref.	Author (location)	Method	OPAT Model(s)	OPAT group(s)	Comparator(s)	Findings
63	Lane et al (USA)	Questionnaire survey	OP; SA	555 adult infectious diseases physicians	—	Lack of a dedicated OPAT team was reported as the most common barrier to providing safe OPAT services (median rank 2), followed by the large number of locations at which patients received OPAT, communication issues, and volume of lab results (median rank 3).
68	Lehoux et al (Canada)	Questionnaire survey	SA	51 nurses	—	Respondents strongly agreed that home IV therapy gave patients greater autonomy ($\bar{x}$ score 5.2; SD 1.0), and made it easier to pursue a normal life ($\bar{x}$ 4.4; SD 1.0). They viewed the technology as simple to use ($\bar{x}$ 4.5; SD 1.1), but believed it triggered some patient anxiety ($\bar{x}$ 3.3; SD 1.3).<br>Nursing teams perceived that IV therapy provided some challenges for providers in relation to the technical complexity of devices ($\bar{x}$ 3.5; SD 1.3), number of devices used by their hospital ($\bar{x}$ 3.3; SD 1.4), pace of product renewal ($\bar{x}$ 3.2; SD 1.4) and fit with the home setting ($\bar{x}$ 3.1; SD 1.4).<br>Dealing with patients' psychological ($\bar{x}$ 3.0; SD 1.0) and cognitive limitations ($\bar{x}$ 3.0; SD 1.1) was viewed as slightly more demanding than dealing with physical limitations ($\bar{x}$ 2.9; SD 1.1). There was a perceived increase in effort related to teaching patients how use the technology ($\bar{x}$ 3.1; SD 1.1), about associated risks (mean 3.0; SD 1.1), how to manage their anxiety ($\bar{x}$ 3.0; SD 1.1) and how to manage impact on their life ($\bar{x}$ 2.8; SD 1.0).<br>The two features reported as being most important for improvement, were ease of use for patients ($\bar{x}$ 3.9; SD 1.2) and integrating the technology into the patient's life ($\bar{x}$ 4.0; SD 1.3).
86	Muldoon et al (Ireland)	Questionnaire survey	Not specified	55 consultant physicians (15% clinical microbiologists)	—	Forty-one respondents (74%) had discharged patients with IV antibiotics, but almost half (n=26, 47%) did not have a designated OPAT service available to them; all felt that there was a need for a local OPAT service. Of respondents answering the question, 63% (12/19) reported that they had experienced difficulty obtaining funding for OPAT.<br>Forty-five percent (13/18) reported that they thought >75% of their patients were satisfied with OPAT services; only one consultant (6%) felt that <50% of patients were satisfied.
87	Muldoon et al (USA)	Questionnaire survey	Not specified	316 adult and paediatric infectious diseases physicians	—	Thirty-four respondents rarely or never participated in OPAT: 48% cited logistic reasons or lack of an OPAT program, 15% that another practitioner had responsibility for OPAT, 15% reported lack of control of patient management, 12% lack of reimbursement, and 10% low patient volume.<br>In the 'Comments' section, many respondents reported

SUPPLEMENTARY TABLES

Ref.	Author (location)	Method	OPAT Model(s)	OPAT group(s)	Comparator(s)	Findings
						frustration about lack of institutional and financial support for OPAT, while also expressing a significant liability burden.
93	Parker et al (UK)	Questionnaire survey; focus group; call to practices	OP; SA	29 patients; 38 GP practices	—	Most GP practices (76%) saw no advantage for themselves in home IV treatment. Those that did mentioned admissions saved and freed up hospital beds, that the GP maintained responsibility for the patient, and that the patient could be treated at home. Most practices (70%) saw a substantial disadvantage in increased workload, and a small number were concerned about the safety of IV access (18%). However, almost all (94%) thought patients would benefit from being in their own environment, although many saw distance from hospital and lack of support and nursing care as a disadvantage (68%).
107	Seaton & Nathwani (UK)	Questionnaire survey	Not specified	157 infection specialists (92% clinical microbiologists)	—	Most of the 157 respondents had experience of OPAT, but only 21% had an established program within their institution. Of those with no program, 61% thought there was a definite need, while 14% thought there was no need for a formal program. Only 2% thought IV therapy should always be given in hospital.<br>Of the 124 specialist without a program, perceived barriers to development of OPAT were mainly organisational, including source of funding (n=43), lack of leadership (n=42), links between hospital and community (n=37), and identification and training of staff (n=21). Small numbers of patients or fragmentation of patient distribution within Trusts was also a concern (n=34). Concerns over lack of experience (n=17) or patient safety and acceptability (particularly line care and antibiotic administration), were expressed by a few respondents (n=12).<br>There was no consensus regarding funding and clinical responsibility for patients; 40% thought secondary care should pay and specialists take day-to-day clinical responsibility, while 50% thought that care should be shared between hospital and community practitioners.

OP: outpatient; SA: self-administered; SN: specialist nurse; GN: General nurse.

† Age and gender details relate only to those patients who were clinically evaluable or provided questionnaire data.

CIVAS Systematic Review: Mitchell et al – PRISMA Checklist

Section / topic	Item no.	Checklist item	Reported on page no
**Title**			
Title	1	Identify the report as a systematic review, meta-analysis, or both	1
**Abstract**			
Structured summary	2	Provide a structured summary including, as applicable, background, objectives, data sources, study eligibility criteria, participants, interventions, study appraisal and synthesis methods, results, limitations, conclusions and implications of key findings, systematic review registration number	3
**Introduction**			
Rationale	3	Describe the rationale for the review in the context of what is already known	4
Objectives	4	Provide an explicit statement of questions being addressed with reference to participants, interventions, comparisons, outcomes, and study design (PICOS)	4 & 5
**Methods**			
Protocol and registration	5	Indicate if a review protocol exists, if and where it can be accessed (such as web address), and, if available, provide registration information including registration number	3
Eligibility criteria	6	Specify study characteristics (such as PICOS, length of follow-up) and report characteristics (such as years considered, language, publication status) used as criteria for eligibility, giving rationale	5
Information sources	7	Describe all information sources (such as databases with dates of coverage, contact with study authors to identify additional studies) in the search and date last searched	4-5
Search	8	Present full electronic search strategy for at least one database, including any limits used, such that it could be repeated	Appendix 1
Study selection	9	State the process for selecting studies (that is, screening, eligibility, included in systematic review, and, if applicable, included in the meta-analysis)	5
Data collection process	10	Describe method of data extraction from reports (such as piloted forms, independently, in duplicate) and any processes for obtaining and confirming data from investigators	6
Data items	11	List and define all variables for which data were sought (such as PICOS, funding sources) and any assumptions and simplifications made	6
Risk of bias in individual studies	12	Describe methods used for assessing risk of bias of individual studies (including specification of whether this was done at the study or outcome level), and how this information is to be used in any data synthesis	6-7; Appendix 2
Summary measures	13	State the principal summary measures (such as risk ratio, difference in means).	N/A
Synthesis of results	14	Describe the methods of handling data and combining results of studies, if done, including measures of consistency (such as I^2 statistic) for each meta-analysis	7
Risk of bias across studies	15	Specify any assessment of risk of bias that may affect the cumulative evidence (such as publication bias, selective reporting within studies)	8-10
Additional analyses	16	Describe methods of additional analyses (such as sensitivity or subgroup analyses, meta-regression), if done, indicating which were pre-specified	N/A
**Results**			
Study selection	17	Give numbers of studies screened, assessed for eligibility, and included in the review, with reasons for exclusions at each stage, ideally with a flow diagram	7; 27 (Figure 1); 28 (Table 1)

For peer review only

Study characteristics	18	For each study, present characteristics for which data were extracted (such as study size, PICOS, follow-up period) and provide the citations	7-8; 29-35 (Table 2); Tables S1-S5
Risk of bias within studies	19	Present data on risk of bias of each study and, if available, any outcome-level assessment (see item 12).	9-10
Results of individual studies	20	For all outcomes considered (benefits or harms), present for each study (a) simple summary data for each intervention group and (b) effect estimates and confidence intervals, ideally with a forest plot	36; (Table 3)
Synthesis of results	21	Present results of each meta-analysis done, including confidence intervals and measures of consistency	N/A
Risk of bias across studies	22	Present results of any assessment of risk of bias across studies (see item 15)	N/A
Additional analysis	23	Give results of additional analyses, if done (such as sensitivity or subgroup analyses, meta-regression) (see item 16)	N/A
**Discussion**			
Summary of evidence	24	Summarise the main findings including the strength of evidence for each main outcome; consider their relevance to key groups (such as health care providers, users, and policy makers)	14
Limitations	25	Discuss limitations at study and outcome level (such as risk of bias), and at review level (such as incomplete retrieval of identified research, reporting bias)	14-15
Conclusions	26	Provide a general interpretation of the results in the context of other evidence, and implications for future research	15-16
**Funding**			
Funding	27	Describe sources of funding for the systematic review and other support (such as supply of data) and role of funders for the systematic review	17

For peer review only

Clinical and cost-effectiveness, safety and acceptability of community intravenous antibiotic service models: CIVAS systematic review

Journal:	*BMJ Open*
Manuscript ID	bmjopen-2016-013560.R1
Article Type:	Research
Date Submitted by the Author:	24-Jan-2017
Complete List of Authors:	Mitchell, Elizabeth; University of Leeds, Leeds Institute of Health Sciences<br>Czoski Murray, Carolyn; University of Leeds, Leeds Institute of Health Sciences<br>Meads, David; University of Leeds, Leeds Institute of Health Sciences<br>Minton, Jane; Leeds Teaching Hospitals NHS Trust,<br>Wright, Judy; University of Leeds, Leeds Institute of Health Sciences<br>Twiddy, Maureen; University of Leeds, Leeds Institute of Health Sciences
**Primary Subject Heading**:	Health services research
Secondary Subject Heading:	Infectious diseases, Evidence based practice
Keywords:	Organisation of health services < HEALTH SERVICES ADMINISTRATION & MANAGEMENT, Quality in health care < HEALTH SERVICES ADMINISTRATION & MANAGEMENT, Infection control < INFECTIOUS DISEASES

SCHOLARONE™ Manuscripts

Clinical and cost-effectiveness, safety and acceptability of community intravenous antibiotic service models: CIVAS systematic review

ED Mitchell, C Czoski Murray, D Meads, J Minton, J Wright, M Twiddy

Centre for Health Services Research, Leeds Institute of Health Sciences, University of Leeds, Charles Thackrah Building, 101 Clarendon Road, Leeds UK LS2 9LJ

ED Mitchell, *Senior Research Fellow*; C Czoski Murray, *Senior Research Fellow*; M Twiddy, *Senior Research Fellow*

Academic Unit of Health Economics, Leeds Institute of Health Sciences, University of Leeds, Charles Thackrah Building, 101 Clarendon Road, Leeds UK LS2 9LJ

D Meads, *Associate Professor*; J Wright, *Senior Information Specialist*

Leeds Teaching Hospitals NHS Trust, Infection and Travel Medicine, St James's Hospital, Beckett Street, Leeds UK LS9 7TF Road, Leeds UK LS2 9LJ

J Minton, *Consultant in Infectious Diseases*

For peer review only

Correspondence to:

Dr Liz Mitchell, Leeds Institute of Health Sciences, University of Leeds, Charles Thackrah Building, 101 Clarendon Road, LEEDS UK LS2 9LJ; *Email*: e.d.mitchell@leeds.ac.uk

WORD COUNT: 5,102

For peer review only - http://bmjopen.bmj.com/site/about/guidelines.xhtml

ARTICLE SUMMARY

Strengths and limitations of this study

- This review provides the most comprehensive picture of OPAT-related research in recent years, bringing together available evidence on the clinical and cost-effectiveness, safety and acceptability of community-based antibiotic delivery
- Much of the work in this area is based around service evaluation and many studies provided only basic descriptive findings, making it difficult to grade the quality and robustness of the evidence
- Although many studies were identified and included in the review, the definitiveness of its conclusions related to the clinical benefit of OPAT over traditional inpatient treatment is limited by the lack of good quality, comparative studies
- Nonetheless, the synthesis provided by this review provides much needed evidence to inform the design and commissioning of OPAT services

For peer review only

ABSTRACT

Objective: Evaluate evidence of the efficacy, safety, acceptability and cost-effectiveness of outpatient parenteral antimicrobial therapy (OPAT) models.

Design: Systematic review.

Data sources: MEDLINE, EMBASE, CINAHL, Cochrane Library, NHS EED, RePEc, Tufts CEA Registry, Health Business Elite, HMIC, Web of Science Proceedings, International Pharmaceutical Abstracts, British Society for Antimicrobial Chemotherapy website. Searches were undertaken from 1993-2015.

Study selection: All studies, except case reports, considering adult patients or practitioners involved in the delivery of OPAT were included. Studies combining outcomes for adults and children or non-IV and IV antibiotic groups were excluded, as were those focused on process of delivery or clinical effectiveness of one antibiotic over another. Titles/abstracts were screened by one reviewer (20% verified). Two authors independently screened studies for inclusion.

Results: 128 studies involving >60,000 OPAT episodes were included. 22 studies (17%) did not indicate the OPAT model used; only 29 involved a comparator (23%). There was little difference in duration of OPAT treatment compared to inpatient therapy, and overall OPAT appeared to produce superior cure/improvement rates. However, when models were considered individually, outpatient delivery appeared to be less effective, and self-administration and specialist nurse delivery more effective. Drug side-effects, deaths and hospital re-admissions were similar to those for inpatient treatment, but there were more line-related complications. Patient satisfaction was high, with advantages seen in being able to resume daily activities and having greater freedom and control. However, most professionals perceived challenges in providing OPAT.

Conclusions: There were no systematic differences related to the impact of OPAT on treatment duration or adverse events. However, evidence of its clinical benefit compared with traditional inpatient treatment is lacking, primarily due to the dearth of good quality, comparative studies. There was high patient satisfaction with OPAT use but the few studies considering practitioner acceptability highlighted organisational and logistic barriers to its delivery.

For peer review only - http://bmjopen.bmj.com/site/about/guidelines.xhtml

INTRODUCTION

Delivery of intravenous (IV) antibiotics outside of the hospital setting (often termed outpatient parenteral antimicrobial therapy (OPAT)) is widely accepted as standard practice in many countries, including the USA and Australia.[1] In the USA alone, an estimated quarter of a million patients receive IV antibiotics on an outpatient basis each year,[2] and this method of delivery has been used to treat a wide variety of infections, among them skin and soft tissue infections, bone and joint infections, pneumonia, and endocarditis.

In recent years OPAT services have also been developed in the UK, both in the NHS and private sectors.[3] It has the potential to deliver cost efficiency savings and improved patient experience[4] through fewer in-patient bed days, antimicrobial agents that need less frequent administration and choice of treatment setting. Despite this, implementation of OPAT is patchy and there is significant geographical variation in both the setting and the health care professions involved. Four primary models of OPAT delivery are in use: outpatient attendance at a healthcare facility, patient (or carer) self-administration, administration by a visiting general nurse, and administration by a visiting specialist nurse.

The failure to realise the full potential of OPAT in the UK is due in no small part to the paucity of information related to optimal delivery, most notably evidence of effectiveness, associated risks, and patient preferences for this form of treatment. The purpose of this review was to evaluate existing evidence in relation to the efficacy, safety, acceptability and cost-effectiveness of different models of OPAT delivery.

METHODS

Identification of studies

A comprehensive search strategy was developed by an experienced information specialist (JW) and a range of bibliographic sources were searched for the period 1993 to March 2015: Medline, Medline in process, EMBASE, CINAHL, International Pharmaceutical Abstracts, the Cochrane Library, the NHS Economic Evaluation Database (EED), Research Papers in Economics (RePEc), Tufts Cost-Effectiveness Analysis (CEA) Registry, and Health Business Elite (HBE). This timeframe was selected to take account of the substantial changes in the organisation of the NHS and the shift towards more community involvement that have taken place following publication of National Health Service and Community Care Act 1990.[5] Two separate searches were run. The first identified studies of IV antibiotics and known models of delivery, while the second identified reviews of antibiotic use in cellulitis or cystic fibrosis (infections where OPAT is frequently used) to allow for the identification of delivery models that

For peer review only - http://bmjopen.bmj.com/site/about/guidelines.xhtml

were unknown to us and subsequently not considered when identifying terms for the first search.

Supplementary searches of Web of Science Proceedings, the Health Information Management Consortium (HMIC), and the website of the British Society for Antimicrobial Chemotherapy were conducted to identify relevant unpublished work. In addition, the reference lists of included studies were reviewed for potentially relevant papers (see Appendix 1 for sample search strategies and list of databases searched).

Criteria for inclusion

Studies evaluating the clinical effectiveness or cost-effectiveness of an OPAT model, describing or evaluating patient safety issues associated with OPAT, or considering the acceptability of OPAT were included. The population included adult patients treated for any condition (and/or their carer in the case of acceptability studies) or practitioners involved in the delivery of OPAT. Any form of intravenous antibiotic drug delivery system (e.g. infusion or bolus) was included. Studies of any research design were considered (with the exception of single case reports), and no language restrictions were applied.

Studies were excluded if they considered the costs related to a model of delivery but did not consider patient benefit alongside these, or if they made reference to costs and benefits but did not report specific cost-effectiveness data (such as cost per Quality Adjusted Life Year (QALY)). Similarly, studies that made reference to clinical effectiveness without reporting specific patient outcomes were also excluded. Studies that included children or that involved multiple routes of antibiotic delivery were reviewed but excluded if they did not differentiate between outcomes for adult patients or for patients receiving IV treatment, and those of other participants. Studies that focused only on the method or process of delivery or on the clinical effectiveness of a single treatment or of one class of antibiotic over another were excluded, as were abstract only, descriptive or commentary pieces and guidance documents.

Selection of studies

Titles and abstracts of all identified studies were screened for eligibility by one reviewer (EDM) with a random selection (20%) independently screened by a second. Full text versions of papers not excluded at this stage were obtained for detailed review and independently assessed by two reviewers (EDM with CCM, DM or MT) to determine whether they met the inclusion criteria. Differences of opinion were discussed until a consensus was reached, with validation sought from a third reviewer where necessary.

For peer review only

For peer review only - http://bmjopen.bmj.com/site/about/guidelines.xhtml

Data extraction

Data extraction was carried out by one experienced reviewer (EDM) using a standardised proforma. Data for a sample of studies were extracted independently by a second reviewer (CCM) in order to validate the items being collected. Information was collected on study purpose and design (including factors related to quality assessment), setting, duration, population and clinical characteristics, models of delivery (outpatient, self-administration, general nurse, specialist nurse), outcome area (clinical effectiveness, cost-effectiveness, safety, acceptability), antibiotic parameters (type, delivery route, treatment dose), outcome measures, follow-up and key findings.

Assessment of bias

Studies were assessed for bias by two reviewers (EDM and CCM), where possible using previously developed scoring systems. The Cochrane Risk of Bias assessment tool was used for experimental studies (randomised controlled trials, clinical trials, controlled before and after studies), and the Newcastle-Ottawa scales for cohort and case-control studies.[6-7]

A method of assessing the strength of evidence of other observational studies – developed for previous reviews on early diagnosis of cancer[8-10] – was modified for this topic area and applied to relevant studies. In this system, papers were evaluated on three key areas: Population, Methodology and Analysis (Appendix 2). Population assesses the method used to ensure that a study is appropriately powered/produces generalizable results, with use of a sample size calculation or inclusion of all possible patients or providers rated more highly than selective recruitment. Methodology assesses procedures for obtaining study data, with use of a rigorous approach designed to reduce systematic differences between groups (selection, characteristics, treatment etc.) rated more highly than other methods. Analysis assesses use of analytic techniques, with reporting of relevant significant comparisons or differences (or use of appropriate analytic techniques if qualitative) rated more highly than non-statistical comparisons or descriptive data.

Four categories were then used to the weight the evidence: *STRONG+* (study graded strong in all three areas); *STRONG–* (study graded strong in two areas and moderate in the third); *MODERATE* (study graded strong in one area and moderate in two, or moderate in all three areas); *INSUFFICIENT* (study used a selective study population and/or an inappropriate method to ascertain data, or did not provide enough information to be able to determine a grading).

For peer review only - http://bmjopen.bmj.com/site/about/guidelines.xhtml

Many of the papers included in this review used methodologies that did not lend themselves to the scoring systems outlined. Many studies included all patients in receipt of OPAT since its establishment at a particular institution or over a specified time period, and simply reported conditions treated and therapies used, along with limited outcomes data. Case-series such as these, which were to all intents and purposes service evaluations that included little or no analytic content, were not subject to formal quality assessment.

In keeping with accepted good practice, studies at risk of bias were not excluded from the review, but an appraisal of the strength of existing evidence has been reported, and findings interpreted in light of this.

Data synthesis

The main characteristics of included studies and findings relating to clinical effectiveness, cost-effectiveness, patient safety, acceptability, and study quality have been summarised in narrative and tabular form. Substantial heterogeneity in clinical characteristics – condition treated, treatment duration, definition of a successful outcome (cure, improvement, deterioration etc.) – and methodology precluded pooling of study data for meta-analysis.

Patient involvement

This review formed one work stream of a larger NIHR-funded study that included qualitative interviews and economic modelling. The research team included a lay co-applicant who commented on study design prior to submission of the funding proposal, including the focus of the systematic review and the outcomes to be included. In addition, a Patient Advisory Group (PAG) was established to input at key phases throughout the project including the design of patient recruitment materials, interview topic guides and health economics aspects of the study. An Expert Panel was convened to discuss emerging findings from each work stream, and a representative of the PAG was a member of the panel. A project dissemination event was held in April 2016 to which both patients and professionals were invited.

RESULTS

The search strategy identified 7,214 articles of which 589 met the inclusion criteria for detailed review (Figure 1). The full-text of an additional 17 papers, identified from the reference lists of previous reviews and identified references, was also obtained giving a total of 606 potentially relevant papers. In a change to the initial protocol, non-English language papers were identified but were not assessed for inclusion (n=69), and we were unable to obtain one article from the library document supply service. One hundred and twenty-eight papers involving more than 60,000 OPAT episodes were included in the final analysis.[11-138]

For peer review only - http://bmjopen.bmj.com/site/about/guidelines.xhtml

Population and setting

Three-quarters of studies were undertaken in Europe (n=53; 41%) and North America (n=45; 35%). Almost two-thirds of the European studies were UK based. Two studies involved centres in more than one country,[41, 101] and one was a systematic review of world literature.[60] Studies were comparatively small in size , involving between 6 and 11,427 participants or episodes of care (mean 476; median 100); almost two-thirds (63%) had fewer than 150 participants. It was not possible to determine total participant numbers in two studies.[32, 138] The period under study ranged from 6 weeks to 15 years. In general, those studies with the largest numbers of subjects (>1000) either analysed all cases included in an OPAT registry and/or reviewed cases over a more substantial time period.[18, 32, 37, 41, 45, 66, 76, 84, 112, 130, 133]

The most commonly reported reason for treatment was osteomyelitis (n=68, 53%), followed by endocarditis (n=53, 41%), skin and soft tissue infection (n=41, 32%), cellulitis (n=32, 25%), and septic arthritis (n=29, 23%). Most studies involved multiple conditions (n=72, 56%); seven did not specify indications for treatment. These included two qualitative studies and one survey of patient acceptability,[16, 26, 67] three surveys of practitioner acceptability,[63, 68, 107] and one secondary analysis of data from an OPAT database.[17]

Outcomes and OPAT models studied

Of the five outcome areas evaluated in the review, the most commonly considered were patient safety (n=109; 85%) and clinical effectiveness (n=89; 70%). Just over a quarter of studies involved some aspect of patient acceptability (n=37; 29%), but few determined cost-effectiveness (n=5) or practitioner acceptability (n=6). Most investigated multiple areas (70%).

Twenty two of the 128 studies (17%) either did not indicate the type of OPAT delivery model used, or reported that home treatment was used without providing any additional detail. In the remainder, the most frequently reported model across studies was self (or carer) administration (n=66; 52%), followed by visits from a specialist nurse (n=44; 34%), outpatient treatment (n=35; 27%), and visits from a general nurse (n=14; 11%). Just over half of these studies evaluated a single model of delivery (n=59; 55%). Other, less common delivery methods and locations included infusion centres, home infusion or home care companies, Hospital in the Home Units (HHU), prison, and doctor visits (Tables S1–S5).

Quality assessment

Few studies employed an experimental or quasi-experimental design (Tables S1–S5). Most (67%) were observational, primarily case-series, and many involved retrospective data

For peer review only - http://bmjopen.bmj.com/site/about/guidelines.xhtml

collection (n=57; 45%). Of the 12 included randomised controlled trials, five reported on subgroup analyses from the main study and all failed to provide details of the trial methodology.[15, 77, 99-100, 102] One of two included clinical trials did not specify the delivery model under evaluation.[39]

Many studies analysed data obtained from medical records or prospectively held OPAT databases (42%); a small number of others carried out secondary analysis of national or international OPAT Registries. Satisfaction surveys, either by questionnaire or telephone completion, were frequently used, while interviews (face-to-face, telephone, focus group) and visual analogue scales were also well represented. Less common methods of data collection included clinic or ward diaries, direct observation, and data from previous studies or the published literature. It was often unclear who collected data, and few studies reported on how this was carried out. A substantial number provided little or no detail on the methods used. Twenty one of the 128 (16%) studies received full or partial funding by pharmaceutical companies, 72 others (56%) did not report the funding source.

Risk of bias within studies

Three of the 14 included trials were assessed as having a low risk of bias,[30, 103, 131] and one a high risk of bias (pilot study where patients self-selected hospital or home treatment and were recruited consecutively to each arm after this decision was made).[40] In the remaining ten studies, the level of potential bias was unclear. In five cases, it was uncertain whether randomisation or controlling had taken place. Four of these studies reported on subgroup analyses from a single open label trial, and none provided details of the original study methodology.[15, 77, 99-100] The fifth reported on two related trials comparing IV with oral treatment for neutropenia in cancer patients, and again reported no methodological details of the parent studies.[39] In the remaining five trials, details of randomisation, allocation concealment and blinding (especially in relation to assessment of outcome measures) were poorly reported.[101-102, 111, 116, 132] In addition, one the trials closed early due to poor recruitment,[87] whilst population bias due to age and treatment duration differences could not be excluded from another.[132]

All five included case-control studies (three of them retrospective) rated low for potential bias (median 8/9; range 6-9).[44, 78, 98, 136-137] Those studies scoring lowest did not provide details on the methods used to determine outcomes.[44, 78] Similarly, four cohort studies (two retrospective) all had low potential for bias (median 8/9; range 7-9)[12, 20, 76, 112]; lower scores related to possible failure to control for potential differences.[20, 76]

For peer review only - http://bmjopen.bmj.com/site/about/guidelines.xhtml

In the majority of observational studies, the data analysed were derived from OPAT databases or medical records review. Many studies also involved questionnaire surveys. Only five included a comparator (inpatient care),[23, 61, 75, 81, 122] with many simply including all OPAT patients over a selected time period. Most studies reported descriptive results only, with no statistical testing of differences.

Impact on clinical outcomes

Only 21 of the 89 studies evaluating the clinical effectiveness of OPAT included a comparator, which with few exceptions, was inpatient treatment (Table 1; Table S1). Five of the studies did not specify the OPAT delivery model used,[39, 75, 122, 132, 136] while two others reported combined results for multiple models.[102, 137]

Regardless of the particular OPAT model used, most studies assessing duration of treatment found little difference compared to inpatient therapy[30, 32, 40, 44, 81, 98, 101, 103, 131] (Table 1). Two studies (one using specialist nurses[18] the other self-administration[74]) found that the average length of OPAT treatment was shorter. A third study evaluating a Hospital at Home Unit (HHU) found a significant reduction in treatment duration for patients with cellulitis (median 6 days vs. 8.6 days; 95% CI 0.24-4.85) but not for patients with pyelonephritis (4.6 days vs. 4 days; 95% CI -1.86-0.72).[81]

Evidence of the effect of OPAT on cure rate however, was less conclusive. When findings from all delivery models were considered, OPAT appeared to produce superior results to those seen for inpatient treatment. This may reflect heterogeneity in the population groups, with inpatients being thus because they required more intensive care. However, it is also undoubtedly influenced by the inclusion of positive studies that reported on multiple or unspecified OPAT treatment models.[75, 102, 137] When these were removed and specific models considered individually, outpatient delivery appeared to have a lower rate of cure or improvement than inpatient treatment (89% vs. 95%)[101] and self-administration[20] and OPAT by specialist nurse a higher rate.[44, 81]. One study found that duration of neutropenia-related fever in cancer patients was significantly reduced with specialist nurse delivered OPAT (2 days vs. 5 days; $p<0.001$),[44] and another that rates of recovery for pyelonephritis were higher (93% vs. 90%)[62], although this was not the case for cellulitis (93% vs. 100%).[81] OPAT delivered by general nurse however, had little or no impact on rates of recovery. One study of treatment for community-acquired pneumonia[103] found no significant difference in time to resolution of fever, tachycardia and tachypnoea, while another found no significant difference in days to no advancement of cellulitis[30] (mean 1.50 vs. 1.49; 95% CI -0.3 to 0.28; p=0.90). Results from studies assessing

For peer review only - http://bmjopen.bmj.com/site/about/guidelines.xhtml

the impact of OPAT specifically on lung function in patients with cystic fibrosis were either inconclusive or showed no differential impact.[23, 40, 98, 131]

In the remaining studies that considered clinical effectiveness for OPAT models only, rates of cure or improvement ranged from 61.1-100% (mean 89.6%; median 92.5%). When the various OPAT models were considered individually, the highest mean cure/improvement rate was found for self-administration (91.3%), followed by specialist nurse (90.6%), general nurse (90.0%) and outpatient treatment (88.3%). Few studies reported on bacterial eradication, but those that did saw rates of between 57.1-100% (mean 86.2%; median 90.0%) (Table S1).

Patient safety and adverse events

Only 24 of the 109 studies evaluating OPAT-related safety included a comparator, which in the majority of cases was inpatient treatment (Table 1; Table S2). Five studies did not specify the OPAT model being used[39, 61, 75, 132, 136] and three others reported combined results for multiple models.[76, 102, 137]

There was little evidence of impact on either drug-related side effects or number of deaths in OPAT patients compared to patients receiving treatment in hospital[20, 32, 40, 44, 76, 81, 98, 101, 103, 109, 131] (Table 1). One study evaluating OPAT via outpatient treatment[101] reported a higher death rate (1 patient vs. 0 patients), but this was a small study and the overall rate of side-effects was lower in the OPAT group (15% vs. 18%). There also appeared to be no conclusive evidence of benefit either in relation to hospital re-admissions overall or in relation to those who self-administered therapy.[20, 131] There were conflicting results for OPAT delivered by nurses, with specialist nurses[32, 78, 81, 109] appearing to have superior results to those of general nurses.[30, 103] Perhaps unsurprisingly, overall there appeared to be more line-related complications in IV therapy administered outside of hospital.

Across all studies, the most commonly reported adverse events were rash, fever, nausea/vomiting, diarrhoea, allergic reaction or anaphylaxis, phlebitis, leucopenia, and line complications (including line infection, occlusion, breakage, and dislodgement) (Table S2).

Cost-effectiveness of OPAT

While many of the identified studies reported on the cost of OPAT, only five considered cost-effectiveness (Table S3). Three studies applied decision tree models to OPAT provided by specialist nurses,[95, 119, 138] with one also determining the cost-effectiveness of self-administration.[138] The remaining studies (one literature review, one retrospective observational study) did not specify the OPAT model(s) used.[60, 122]

For peer review only - http://bmjopen.bmj.com/site/about/guidelines.xhtml

In two of the three decision tree analyses, IV OPAT was found to be more cost-effective than IV inpatient therapy.[119, 138] In one case, it was also more cost-effective than early discharge with oral therapy and oral outpatient therapy.[119] In the other, its dominance was only maintained when the IV success rate was greater than 55%.[138] Conversely, in the third study IV OPAT was found to be less cost-effective than both IV to oral switch therapy, and oral treatment both during and after hospitalisation (which was the most cost-effective option).[95] However, the probability estimates used in two of the three scenarios in this study were derived from different data sources (published research and institutional data). In addition, there were no published data related to the baseline probability for cure in the third scenario, and so this was assumed to be at some point between the estimates used in the other two.

Studies included in the literature review predominantly concluded that home IV antibacterial therapy would lead to significant cost reductions, both from a societal and third-party payer perspective.[60] In five of the 11 studies, inpatient therapy was 2-3 times as expensive as home care therapy. However, there was considerable variation in the way in which costs were determined and calculated across studies (e.g. incremental costs, costs for selected components only etc.), while the review itself lacked considerable detail on the methodology used and the criteria for study inclusion and exclusion.

Findings from the observational study (based on 435 courses of IV antibiotic treatment for respiratory exacerbations in 116 adult patients with cystic fibrosis) indicated that for both one course and one year of treatment, IV antibiotic treatment administered mostly in hospital was more effective but more costly than treatment administered mostly at home.[122] This improved clinical effectiveness could only be achieved with the input of considerable additional resources (between £46 and £73k per patient at 2002 prices). However, when the strictest definition of effectiveness was applied (≤0% decline in lung function), hospital treatment was unlikely to ever be cost-effective.

Acceptability of OPAT

Patient preference

Only four of the 36 studies considering patients' acceptability of OPAT involved a comparison of inpatient and outpatient therapy (Table S4), two where OPAT delivery was by general nurses[30, 103] and two where delivery was by specialist nurses.[32, 44] In each case, satisfaction was high, with home treatment seen as beneficial. One of the two general nurse studies found that only 5% of home group patients would have preferred hospital treatment, compared with 35% of hospital patients who would have preferred treatment at home ($p<0.001$).[30] The other found

For peer review only - http://bmjopen.bmj.com/site/about/guidelines.xhtml

that patients in the home group were significantly happier with the location of their care than those receiving inpatient therapy ($p<0.001$).[103] Similarly, in one of the studies where patients received home care by specialist nurses, almost all (97%) indicated that they would chose to receive at-home therapy in future and would recommend it to others.[44] The main reasons given for this were quiet and increased home comfort, familiar environment, and free choice of activity. However, some patients in this study did report disadvantages to receiving therapy at home, primarily related to patient and caregiver anxiety.[44]

In the remaining studies that considered acceptability in OPAT patients only, most involved multiple OPAT models (and did not differentiate between them in their findings), or did not specify the model(s) under study. For the most part, satisfaction with treatment was very high,[11, 26, 28, 42, 45, 48, 51-52, 55-57, 59, 74, 89-90, 104-105, 108, 113, 120, 123-124] including where patients had to have frequent attendance at hospital.[22, 135] Commonly perceived advantages of OPAT included the ability to resume daily activities,[45, 56, 59, 74] feelings of improved self-esteem or greater freedom and control,[48, 56, 74, 82, 93] and not having to remain in or attend hospital.[11, 56, 59, 82, 118] The main disadvantages were most commonly related to infusion equipment, and included anxiety about the device and its sterility,[16, 67, 97] Two studies found that younger patients were better able to use infusion devices, and required less support to do so than older patients.[31, 67]

Two studies determined patients' willingness to pay to have treatment in their preferred location, and although differences did not reach statistical significance, patients reported that they would pay more for home than hospital-based treatment, including giving up slightly more of their remaining life to ensure this.[73, 120]

Provider preference

Only six studies included assessment of practitioner acceptability; one involving GPs, one involving nurses, and four involving infection specialists (Table S5). In most cases, professionals saw advantages (or a need) for patients receiving IV antibiotic therapy outside of hospital.[68, 86, 93, 107] However, there were also negative perceptions in relation to practitioner involvement. Most GPs saw no advantage to themselves in home treatment, and many thought distance from hospital was an issue for patients.[93] Similarly, nurses perceived that there were challenges in providing this model of care, mainly around the technical nature of the devices used, and dealing with patients' understanding of the technology and its related risks.[68] Finally, many specialists saw organisational barriers to the use of OPAT, in terms of funding, leadership, and the links between primary and secondary care.[63, 86-87, 107] In addition, there was no consensus regarding who should assume the cost or take clinical responsibility for patients.[87, 107]

For peer review only - http://bmjopen.bmj.com/site/about/guidelines.xhtml

DISCUSSION

Principal findings

Rates of cure from the published literature, when aggregated, show that there are no systematic differences in relation to the impact of OPAT on duration of therapy, or on adverse events associated with IV antibiotic treatment. In addition, OPAT is, on the whole, more cost-effective than inpatient care. However, conclusive evidence as to the clinical benefit (or otherwise) of this mode of therapy compared with traditional inpatient IV treatment is lacking. Acceptability of OPAT appears to be high among patients, who appreciate the greater freedom that it provides, particularly in relation to being able to resume daily activities (such as going to work or school), having greater control over their illness, and not having to attend hospital but being able to stay at home with family. Disadvantages identified most commonly related to the use of infusion equipment. Few studies considered practitioner acceptability, but those that did found some concerns related to the logistics involved in providing an OPAT service, including cost and who would assume clinical responsibility for patients.

The results of this review should be used by clinicians in the context of their patient populations and the types of infection being treated. Results from ongoing research studies, such as work being done by Scarborough and colleagues to investigate the possibility of early switch from inpatient IV to oral antibiotics[139] are likely to change the way in which patient care is planned and implemented. However, the results of this review (within the constraints of the literature) have demonstrated that OPAT is safe, effective and cost-effective, and as such, patients are likely to continue to be treated using OPAT models. In addition, the evidence shows that patients prefer OPAT, so it is likely that there will be continued development of community-based services.

Limitations of the study

Although many studies were identified and included in this review, its conclusions are limited by the lack of studies involving a usual care comparison, or comparison with other models of OPAT delivery. In addition, few studies employed a rigorous study design. Much of the work in this area appears to be based around service evaluation, and as such many of the studies provided only basic descriptive findings, with no estimates of variance and limited data related to patient outcomes. As a result, it is difficult to grade the quality and robustness of the evidence, even in the few randomised controlled trials that have been conducted. Similarly, substantial clinical and methodological heterogeneity precluded pooling of data for meta-analysis. The majority of studies included a varied case-mix and did not differentiate their results between conditions treated. In addition, there was variation across studies in relation to

For peer review only - http://bmjopen.bmj.com/site/about/guidelines.xhtml

what constituted a successful outcome (cure, improvement, deterioration etc.) as well as a lack of consistent treatment duration. Although this undoubtedly reflects real-world practise, it meant that it was not possible to pool the results of individual studies to provide estimates of true effect size when using OPAT for different patient groups, or even for comparing OPAT as a whole with inpatient treatment. Although identified as part of the search strategy, time constraints meant that Non-English language papers were not assessed beyond the abstract screening stage, and it is possible that this may have resulted in some language bias (through exclusion of negative findings that may be more likely to have been published in non-international journals). However, given the lack of robust, conclusive studies that were included, this is unlikely to have significantly influenced the findings of the review.

For peer review only

Implications for clinicians and policymakers

It is likely that the increased use of OPAT in the UK in recent years is due, in no small part, to clinician's expectations that it should increase hospital inpatient capacity whilst delivering non-inferior patient care and improved patient experience. However, this review has demonstrated that the evidence related to care is not strong. Few studies reported on the different levels of service required to account for the complexity of patient cases encountered, including those with co-morbidity and those requiring 'one off' or longer term treatment. In addition, many studies provided aggregated results and it was not possible to disentangle these either for individual OPAT models or for the specific conditions treated. This, combined with a lack of detail on the delivery models actually used, makes it difficult for clinicians and policymakers to be able to replicate existing practice (and consequently potential outcomes) even from positive studies. OPAT services have the potential to deliver significant costs savings and increased patient satisfaction for the NHS, and it is crucial that this information is reported in future studies in order to identify best practice and help support decision making at a local level. We would recommend that as a minimum, authors fully describe the specific method of OPAT delivery studied (including defining who delivers 'specialist' nurse care), include and report on a comparator when determining the impact of OPAT on clinical outcomes (only 21/89 studies in this review did so), report all numerators and denominators, avoid reporting pooled data (where possible), report on case complexity and average duration of treatment to achieve cure, and adhere to CONSORT guidelines when reporting trials.

Future research

Commissioning healthcare services in England is increasingly complex and has been subject to much change in recent years.[140] The NHS England service specification for Infectious Diseases services[141] includes a requirement to provide OPAT, but at present there is no clear

For peer review only - http://bmjopen.bmj.com/site/about/guidelines.xhtml

commissioning mechanism to achieve this. This review has established that there is currently a dearth of information around key aspects of OPAT, and it is therefore clear that where Clinical Commissioning Groups have opted to commission OPAT services, they are working without the support of a robust evidence-base. It is also clear that there is little evidence upon which recommendations for best practice can be based. Indeed, the main guidance currently in use is the British Society for Antimicrobial Chemotherapy OPAT standards document,[142] which is a consensus statement largely derived from expert opinion.

There is a need for further research to address significant gaps in knowledge about the cost-effectiveness of different IV antibiotic services, and to ascertain which services patients prefer and which aspects of the services are most important to them. This would help identify the most appropriate configuration of services in terms of value for money and patient preference, as well as future research priorities. Since OPAT services are now established in many areas of the UK, conducting a definite randomised controlled trial of different models of care is unlikely to be feasible. Individual centres would not be able to provide all of the necessary comparator services for randomisation, and there would be difficulties related to the heterogeneous nature of the patient population, and consequent decisions about the most relevant outcome measure. Other, more pragmatic, study designs should therefore be considered, including large-scale, prospective observational studies designed to collect robust data on outcomes, adverse events, and the costs of OPAT (including mixed models of care). This, together with high standards of reporting to ensure that delivery models and their results are fully described and reported individually, would go some way to bridging the current information gap.

For peer review only - http://bmjopen.bmj.com/site/about/guidelines.xhtml

Acknowledgements: The authors would like to thank Thomas Veale for help in obtaining papers, Jill Edwards for help with screening abstracts, and Kate Hill for assistance with data extraction. We would also like to thank the members of the CIVAS Patient Advisory Group and Expert Panel.

Contributors: The review was designed by CCM, JM, JW and MT as part of a larger study. JW developed and ran the electronic searches. Data extraction tools were designed by EDM. EDM, CCM, DM and MT were involved in record screening and study selection. EDM and CCM undertook data abstraction and carried out assessment of risk of bias. EDM synthesized the data. The article was drafted by EDM, and all authors contributed to subsequent drafts. EDM is guarantor.

Funding: This work was supported by NIHR Health Services Research and Delivery (HSDR), Project reference 11/2003/60. NIHR had no role in design, conduct, analysis, interpretation of data, or the decision to submit for publication.

Competing interests: All authors have completed the Unified Competing Interest form at www.icmje.org/coi_disclosure.pdf (available on request from the corresponding author) and declare: this work was funded by The National Institute for Health Research; no financial relationships with any organization that might have an interest in the submitted work in the previous three years; no other relationships or activities that could appear to have influenced the submitted work.

Ethical approval: Not required: systematic review.

Data sharing: No additional data are available.

Review registration: This study is registered as PROSPERO CRD42013006374 (available at www.crd.york.ac.uk/PROSPERO/).

For peer review only

REFERENCES

1. Paladino J, Portez D. Outpatient parenteral antimicrobial therapy today. Clinical Infectious Diseases 2010; 51: S198-208.

2. Chary A, Tice AD, Martinelli LP, Liedtke LA, Plantenga MS, Strausbaugh LJ. Experience of infectious diseases consultants with outpatient parenteral antimicrobial therapy: results of an emerging infections network survey. Clinical Infectious Diseases 2006; 43(10):1290-95.

3. Torok M, Chapman A, Lessing M, Sanderson F, Seaton R. Outpatient parenteral antimicrobial therapy: Recent developments and future prospects. Current Opinion in Investigational Drugs 2010; 11: 929-39.

4. Chapman ALN. Outpatient parenteral antimicrobial therapy. BMJ 2013; 346: f1585.

5. Department of Health. National Health Service and Community Care Act 1990. The Stationary Office: London, 1990.

6. Higgins JPT, Green S (eds.). Cochrane handbook for systematic reviews of interventions. Version 5.1.0. Cochrane Collaboration, 2008. Available at: http://handbook.cochrane.org (accessed 11 July 2016).

7. Wells GA, Shea B, O'Connell D, et al. The Newcastle-Ottawa Scale (NOS) for assessing the quality of nonrandomised studies in meta-analyses. Available at: http://www.ohri.ca/programs/clinical_epidemiology/oxford.htm (accessed 11 July 2016).

8. Mitchell ED, Pickwell-Smith B, Macleod U. Risk factors for emergency presentation with lung and colorectal cancers: a systematic review. BMJ Open 2015; 5: 4 e006965.

9. Macleod U, Mitchell ED, Burgess C, Macdonald S, Ramirez AJ. Risk factors for delayed presentation and referral of symptomatic cancer: evidence for common cancers. Br J Cancer 2009; 101: S92-S101.

10. Mitchell E, Macdonald S, Campbell NC, Weller D, Macleod U. Influences on pre-hospital delay in the diagnosis of colorectal cancer: a systematic review. Br J Cancer 2008; 98: 60-70.

11. Al Ansari A, Al Alawi S, Al Qahtani M, Darwish A. Outpatient Parenteral Antimicrobial Therapy (OPAT) in the Kingdom of Bahrain: efficacy, patient satisfaction and cost effectiveness. The Open Infectious Diseases Journal 2013; 7(1): 90-95.

12. Allison GM, Muldoon EG, Kent DM, et al. Prediction model for 30-day hospital readmissions among patients discharged receiving outpatient parenteral antibiotic therapy. Clinical Infectious Diseases 2014; 58(6): 812-19.

13. Amodeo MR, Clulow T, Lainchbury J, et al. Outpatient intravenous treatment for infective endocarditis: safety, effectiveness and one-year outcomes. Journal of Infection 2009; 59(6): 387-93.

14. Anand V, Levine H, Friedman M, et al. Intravenous antibiotics for refractory rhinosinusitis in nonsurgical patients: preliminary findings of a prospective study. American Journal of Rhinology 2003; 17(6): 363-68.

15. Angel JV. Outpatient antibiotic therapy for elderly patients. HIAT Study Group. American Journal of Medicine 1994; 97(2A): 43-49.

16. Bamford KB, Desai M, Aruede MJ, Lawson W, Jacklin A, Franklin BD. Patients' views and experience of intravenous and oral antimicrobial therapy: room for change. Injury 2011; 42(Suppl 5): S24-27.

17. Barr DA, Semple L, Seaton RA. Self-administration of outpatient parenteral antibiotic therapy and risk of catheter-related adverse events: a retrospective cohort study. European Journal of Clinical Microbiology & Infectious Diseases 2012; 31(10): 2611-19.

18. Barr DA, Semple L, Seaton RA. Outpatient parenteral antimicrobial therapy (OPAT) in a teaching hospital-based practice: a retrospective cohort study describing experience and evolution over 10 years. International Journal of Antimicrobial Agents 2012; 39(5): 407-13.

19. Barr DA, Irvine S, Ritchie NS, McCutcheon J, Seaton RA. Risk of venous thromboembolism in patients treated for bacterial infection in the community with outpatient parenteral antimicrobial therapy. QJM 2014; 107(3): 207-11.

For peer review only - http://bmjopen.bmj.com/site/about/guidelines.xhtml

20. Bedi P, Sidhu MK, Donaldson LS, et al. A prospective cohort study of the use of domiciliary intravenous antibiotics in bronchiectasis. Primary Care Respiratory Medicine 2014; 24: 14090.

21. Berman SJ, Johnson EW. Out-patient parenteral antibiotic therapy (OPAT): clinical outcomes and adverse events. Hawaii Medical Journal 2001; 60(2): 31-33.

22. Bernard L, El H, Pron B, et al. Outpatient parenteral antimicrobial therapy (OPAT) for the treatment of osteomyelitis: evaluation of efficacy, tolerance and cost. Journal of Clinical Pharmacy & Therapeutics 2001; 26(6): 445-51.

23. Bradley JM, Wallace ES, Elborn JS, Howard JL, McCoy MP. An audit of the effect of intravenous antibiotic treatment on spirometric measures of pulmonary function in cystic fibrosis. Irish Journal of Medical Science 1999; 168(1): 25-28.

24. Cervera C, Del Rio A, Garcia L, et al. Efficacy and safety of outpatient parenteral antibiotic therapy for infective endocarditis: a ten-year prospective study. Enfermedades Infecciosas y Microbiologia Clinica 2011; 29(8): 587-92.

25. Chambers S, Gallagher K, Metcalf S, Pithie A. Home intravenous antimicrobial service--twelve months experience in Christchurch. New Zealand Medical Journal 2002; 115: 216-18.

26. Chambers S, Gallagher K, Pithie A. Patient acceptability of home intravenous antibiotic therapy. New Zealand Medical Journal 2004; 117: U865.

27. Chan DSG, Archuleta S, Llorin RM, Lye DC, Fisher D. Standardized outpatient management of Klebsiella pneumoniae liver abscesses. International Journal of Infectious Diseases 2013; 17(3): e185-e188.

28. Chapman ALN, Dixon S, Andrews D, Lillie PJ, Bazaz R, Patchett JD. Clinical efficacy and cost-effectiveness of outpatient parenteral antibiotic therapy (OPAT): a UK perspective. Journal of Antimicrobial Chemotherapy 2009; 64(6): 1316-24.

29. Cheong EA, Katelaris CH, Sisson CM, Anderson EA, Byth K. Adverse drug reactions associated with home parenteral therapy. Journal of Pharmacy Practice and Research 2008; 38(4): 267-70.

30. Corwin P, Toop L, McGeoch G, et al. Randomised controlled trial of intravenous antibiotic treatment for cellulitis at home compared with hospital. BMJ 2005; 330: 129-34..

31. Cox AM, Malani PN, Wiseman SW, Kauffman CA. Home intravenous antimicrobial infusion therapy: a viable option in older adults. Journal of the American Geriatrics Society 2007; 55(5): 645-50.

32. Dall L, Peddicord T, Peterson S, Simmons T, Dall T. Hospitalist treatment of CAP and cellulitis using objective criteria to select patients. Infections in Medicine 2003; 20(8): 379-90.

33. Dalovisio JR, Juneau J, Baumgarten K, Kateiva J. Financial impact of a home intravenous antibiotic program on a medicare managed care program. Clinical Infectious Diseases 2000; 30(4): 639-42.

34. Dargan S, Zvonar RK, Saginur R. A review of outpatient parenteral antimicrobial therapy practices and experience at the Ottawa Hospital. Canadian Journal of Hospital Pharmacy 2007; 60(3): 177-83.

35. Dobson PM, Boyle M, Loewenthal M. Home intravenous antibiotic therapy and allergic drug reactions: is there a case for routine supply of anaphylaxis kits? Journal of Infusion Nursing 2004; 27(6): 425-30.

36. Donald M, Marlow N, Swinburn E, Wu M. Emergency department management of home intravenous antibiotic therapy for cellulitis. Emergency Medicine Journal 2005; 22(10): 715-17.

37. Duncan CJA, Barr DA, Seaton RA. Outpatient parenteral antimicrobial therapy with ceftriaxone, a review. International Journal of Clinical Pharmacy 2012; 34(3): 410-17.

38. Duncan CJA, Barr DA, Ho A, Sharp E, Semple L, Seaton RA. Risk factors for failure of outpatient parenteral antibiotic therapy (OPAT) in infective endocarditis. Journal of Antimicrobial Chemotherapy 2013; 68(7): 1650-54.

39. Escalante CP, Rubenstein EB, Rolston KV. Outpatient antibiotic therapy for febrile episodes in low-risk neutropenic patients with cancer. Cancer Investigation 1997; 15(3): 237-42.

40. Esmond G, Butler M, McCormack AM. Comparison of hospital and home intravenous antibiotic therapy in adults with cystic fibrosis. Journal of Clinical Nursing 2006; 15(1): 52-60.

For peer review only - http://bmjopen.bmj.com/site/about/guidelines.xhtml

41. Esposito S, Noviello S, Leone S, et al. Outpatient parenteral antibiotic therapy (OPAT) in different countries: a comparison. International Journal of Antimicrobial Agents 2004; 24(5): 473-78.

42. Esposito S, Leone S, Noviello S, et al. Outpatient parenteral antibiotic therapy for bone and joint infections: an Italian multicenter study. Journal of Chemotherapy 2007; 19(4): 417-22.

43. Esposito S, Leone S, Noviello S, et al. Outpatient parenteral antibiotic therapy in the elderly: an Italian observational multicenter study. Journal of Chemotherapy 2009; 21(2): 193-98.

44. Fernandez-Aviles F, Carreras E, Urbano-Ispizua A, et al. Case-control comparison of at-home to total hospital care for autologous stem-cell transplantation for hematologic malignancies. Journal of Clinical Oncology 2006; 24(30): 4855-61.

45. Goodwin DD, Hanson JC, Berry CP. The changing face of Canadian home parenteral therapy. Journal of Infusion Nursing 2002; 25(6): 372-78.

46. Gourdeau M, Deschenes L, Caron M, Desmarais M. Home iv antibiotic therapy through a medical day care unit. Canadian Journal of Infectious Diseases 1993; 4(3): 158-62.

47. Graninger W, Presterl E, Wenisch C, Schwameis E, Breyer S, Vukovich T. Management of serious staphylococcal infections in the outpatient setting. Drugs 1997; 54 (Suppl 6): 21-28.

48. Grayson ML, Silvers J, Turnidge J. Home intravenous antibiotic therapy. A safe and effective alternative to inpatient care. Medical Journal of Australia 1995; 162(5): 249-53.

49. Gross R, Graziani AL, Laufer D, Turner JL, Ondercin JP, Macgregor RR. Adverse effects of the use of intravenous pentamidine in the home. Infectious Diseases in Clinical Practice 1996; 5(7): 456-58.

50. Heintz BH, Halilovic J, Christensen CL. Impact of a multidisciplinary team review of potential outpatient parenteral antimicrobial therapy prior to discharge from an academic medical center. Annals of Pharmacotherapy 2011; 45(11): 1329-37.

51. Hindes R, Winkler C, Kane P, Kunkel M, Poretz DM. Outpatient intravenous antibiotic therapy in Medicare patients: Cost-savings analysis. Infectious Diseases in Clinical Practice 1995; 4(3): 211-18.

52. Hitchcock J, Jepson AP, Main J, Wickens HJ. Establishment of an outpatient and home parenteral antimicrobial therapy service at a London teaching hospital: a case series. Journal of Antimicrobial Chemotherapy 2009; 64(3): 630-34.

53. Ho J, Archuleta S, Sulaiman Z, Fisher D. Safe and successful treatment of intravenous drug users with a peripherally inserted central catheter in an outpatient parenteral antibiotic treatment service. Journal of Antimicrobial Chemotherapy 2010; 65(12): 2641-44.

54. Htin KF, Friedman ND, Hughes A, et al. Outpatient parenteral antimicrobial therapy is safe and effective for the treatment of infective endocarditis: a retrospective cohort study. Internal Medicine Journal 2013; 43(6): 700-05.

55. Huminer D, Bishara J, Pitlik S. Home intravenous antibiotic therapy for patients with infective endocarditis. European Journal of Clinical Microbiology & Infectious Diseases 1999; 18(5): 330-34.

56. Johansson E, Bjorkholm M, Wredling R, Kalin M, Engervall P. Outpatient parenteral antibiotic therapy in patients with haematological malignancies. A pilot study of an early discharge strategy. Supportive Care in Cancer 2001; 9(8): 619-24.

57. Kayley J, Berendt AR, Snelling MJ, et al. Safe intravenous antibiotic therapy at home: experience of a UK based programme. Journal of Antimicrobial Chemotherapy 1996; 37(5): 1023-29.

58. Keller SC, Ciuffetelli D, Bilker W, et al. The impact of an Infectious Diseases Transition Service on the care of outpatients on parenteral antimicrobial therapy. Journal of Pharmacy Technology 2013; 29(5): 205-14.

59. Kieran J, O'Reilly A, Parker J, Clarke S, Bergin C. Self-administered outpatient parenteral antimicrobial therapy: a report of three year's experience in the Irish healthcare setting. European Journal of Clinical Microbiology & Infectious Diseases 2009; 28(11): 1369-74.

60. Krauth C, Jalilvand N, Welte T, Busse R. Cystic fibrosis: cost of illness and considerations for the economic evaluation of potential therapies. Pharmacoeconomics 2003; 21(14): 1001-24.

61. Lacroix A, Revesta M, Patrat-Delon S, et al. Outpatient parenteral antimicrobial therapy for infective endocarditis: A cost-effective strategy. Medecine et Maladies Infectieuses 2014; 44(7): 327-30.

For peer review only

For peer review only - http://bmjopen.bmj.com/site/about/guidelines.xhtml

62. Lai A, Tran T, Nguyen HM, Fleischmann J, Beenhouwer DO, Graber CJ. Outpatient parenteral antimicrobial therapy at large Veterans Administration medical center. The American Journal of Managed Care 2013; 19(9): e317-e324.

63. Lane MA, Marschall J, Beekmann SE, et al. Outpatient parenteral antimicrobial therapy practices among adult infectious disease physicians. Infection Control and Hospital Epidemiology 2014; 35(7): 839-44.

64. Larioza J, Heung L, Girard A, Brown RB. Management of infective endocarditis in outpatients: clinical experience with outpatient parenteral antibiotic therapy. Southern Medical Journal 2009; 102(6): 575-79.

65. Larioza J, Girard A, Brown RB. Clinical experience with daptomycin for outpatient parenteral antibiotic therapy. American Journal of the Medical Sciences 2011; 342(6): 486-88.

66. Laupland KB, Gill MJ, Schenk L, Goodwin D, Davies HD. Outpatient parenteral antibiotic therapy: evolution of the Calgary adult home parenteral therapy program. Clinical & Investigative Medicine - Medecine Clinique et Experimentale 2002; 25(5): 185-90.

67. Lehoux P. Patients' perspectives on high-tech home care: a qualitative inquiry into the user-friendliness of four technologies. BMC Health Services Research 2004; 4(1): 28.

68. Lehoux P, Richard L, Pineault R, Saint-Arnaud J. Delivery of high-tech home care by hospital-based nursing units in Quebec: clinical and technical challenges. Nursing leadership (Toronto, Ont.) 2006; 19(1): 44-55.

69. Lillie PJ, Andrews D, Eaves K, Darton TC, Chapman ALN. Baseline factors predicting the duration of intravenous antibiotic therapy for cellulitis in an outpatient setting. European Journal of Clinical Microbiology & Infectious Diseases 2010; 29(3): 347-49.

70. Lin JW, Kacker A, Anand VK, Levine H. Catheter- and antibiotic-related complications of ambulatory intravenous antibiotic therapy for chronic refractory rhinosinusitis. American Journal of Rhinology 2005; 19(4): 365-69.

71. Lopardo G. Management of endocarditis: outpatient parenteral antibiotic treatment in Argentina. Chemotherapy 2001; 47(Suppl 1): 24-32.

72. Mackintosh CL, White HA, Seaton RA. Outpatient parenteral antibiotic therapy (OPAT) for bone and joint infections: experience from a UK teaching hospital-based service. Journal of Antimicrobial Chemotherapy 2011; 66(2): 408-15.

73. Marra CA, Frighetto L, Goodfellow AF, et al. Willingness to pay to assess patient preferences for therapy in a Canadian setting. BMC Health Services Research 2005; 5: 43.

74. Martel AY. Home intravenous self-injection of antibiotic therapy. Canadian Journal of Infectious Diseases 1994; 5(Suppl. C): 51C-55C.

75. Martone WJ, Lindfield KC, Katz DE. Outpatient parenteral antibiotic therapy with daptomycin: insights from a patient registry. International Journal of Clinical Practice 2008; 62(8): 1183-87.

76. Matthews PC, Conlon CP, Berendt AR, et al. Outpatient parenteral antimicrobial therapy (OPAT): is it safe for selected patients to self-administer at home? A retrospective analysis of a large cohort over 13 years. Journal of Antimicrobial Chemotherapy 2007; 60(2): 356-62.

77. Mauceri AA. Treatment of bone and joint infections utilizing a third-generation cephalosporin with an outpatient drug delivery device. HIAT Study Group. American Journal of Medicine 1994; 97(2A): 14-22.

78. Mazo S, Emparan C, Vallejo M, Soriano P. Hospital-in-the-home treatment of surgical infectious diseases: an economic analysis. Surgical Infections 2007; 8(6): 567-74.

79. McMahon JH, O'Keeffe JM, Victorian Hith Outcomes Study G, Grayson ML. Is hospital-in-the-home (HITH) treatment of bacterial endocarditis safe and effective? Scandinavian Journal of Infectious Diseases 2008; 40(1): 40-43.

80. Mohammadi S, MacKay K, Ward TT, Forrest GN. Clinical outcomes of a Veterans Affairs outpatient antimicrobial treatment program. Southern Medical Journal 2013; 106(6): 345-49.

81. Montalto M, Dunt D. Home and hospital intravenous therapy for two acute infections: an early study. Australian & New Zealand Journal of Medicine 1997; 27(1): 19-23.

For peer review only - http://bmjopen.bmj.com/site/about/guidelines.xhtml

82. Montalto M. Patients' and carers' satisfaction with hospital-in-the-home care. International Journal for Quality in Health Care 1996; 8(3): 243-51.

83. Montalto M. An audit of patients admitted for home intravenous therapy directly from the emergency department. International Journal of Clinical Practice 1997; 51(7): 433-37.

84. Montalto M, Lui B, Mullins A, Woodmason K. Medically-managed Hospital in the Home: 7 year study of mortality and unplanned interruption. Australian Health Review 2010; 34(3): 269-75.

85. Morales JO, von Behren L. Secondary bacterial infections in HIV-infected patients: an alternative ambulatory outpatient treatment utilizing intravenous cefotaxime. American Journal of Medicine 1994; 97(2A): 9-13.

86. Muldoon EG, Allison GM, Gallagher D, Snydman DR, Bergin C. Outpatient parenteral antimicrobial therapy (OPAT) in the republic of Ireland: results of a national survey. European Journal of Clinical Microbiology & Infectious Diseases 2013; 32(11): 1465-70.

87. Muldoon EG, Switkowski K, Tice A, Snydman DR, Allison GM. A national survey of infectious disease practitioners on their use of outpatient parenteral antimicrobial therapy (OPAT). Infectious Diseases 2015; 47(1): 39-45.

88. Murray H, Stiell I, Wells G. Treatment failure in emergency department patients with cellulitis. Canadian Journal of Emergency Medicine 2005; 7(4): 228-34.

89. Nathwani D, Morrison J, Seaton RA, France AJ, Davey P. Out-patient and home-parental antibiotic therapy (OHPAT): Evaluation of the impact of one year's experience in Tayside. Health Bulletin 1999; 57(5): 332-37.

90. Nathwani D. The management of skin and soft tissue infections: outpatient parenteral antibiotic therapy in the United Kingdom. Chemotherapy 2001; 47(Suppl 1): 17-23.

91. Nathwani D, Barlow GD, Ajdukiewicz K, et al. Cost-minimization analysis and audit of antibiotic management of bone and joint infections with ambulatory teicoplanin, in-patient care or outpatient oral linezolid therapy. Journal of Antimicrobial Chemotherapy 2003; 51(2): 391-96.

92. Pajarón M, Fernández-Miera MF, Allende I, et al. Self-administered outpatient parenteral antimicrobial therapy (S-OPAT) for infective endocarditis: A safe and effective model. European Journal of Internal Medicine 2015; 26(2): 131-36.

93. Parker SE, Nathwani D, O'Reilly D, Parkinson S, Davey PG. Evaluation of the impact of non-inpatient IV antibiotic treatment for acute infections on the hospital, primary care services and the patient. Journal of Antimicrobial Chemotherapy 1998; 42(3): 373-80.

94. Partridge DG, O'Brien E, Chapman ALN. Outpatient parenteral antibiotic therapy for infective endocarditis: a review of 4 years' experience at a UK centre. Postgraduate Medical Journal 2012; 88: 377-81.

95. Patanwala AE, Erstad BL, Nix DE. Cost-effectiveness of linezolid and vancomycin in the treatment of surgical site infections. Current Medical Research and Opinion 2007; 23(1): 185-93.

96. Perez-Lopez J, San Jose Laporte A, Pardos-Gea J, et al. Safety and efficacy of home intravenous antimicrobial infusion therapy in older patients: a comparative study with younger patients. International Journal of Clinical Practice 2008; 62(8): 1188-92.

97. Pilling M, Walley T. Parenteral antibiotics at home in cystic fibrosis: experiences and attitudes of recipients. Health & Social Care in the Community 1997; 5(3): 209-12.

98. Pond MN, Newport M, Joanes D, Conway SP. Home versus hospital intravenous antibiotic therapy in the treatment of young adults with cystic fibrosis. European Respiratory Journal 1994; 7(9): 1640-44.

99. Poretz DM. Treatment of serious infections with cefotaxime utilizing an outpatient drug delivery device: global analysis of a large-scale, multicenter trial. HIAT Study Group. American Journal of Medicine 1994; 97(2A): 34-42.

100. Poretz DM. Treatment of skin and soft-tissue infections utilizing an outpatient parenteral drug delivery device: a multicenter trial. HIAT Study Group. American Journal of Medicine 1994; 97(2A): 23-27.

For peer review only - http://bmjopen.bmj.com/site/about/guidelines.xhtml

101. Rapoport BL, Sussmann O, Herrera MV, et al. Ceftriaxone plus once daily aminoglycoside with filgrastim for treatment of febrile neutropenia: Early hospital discharge vs. standard in-patient care. Chemotherapy 1999; 45(6): 466-76.

102. Rehm S, Campion M, Katz DE, Russo R, Boucher HW. Community-based outpatient parenteral antimicrobial therapy (CoPAT) for Staphylococcus aureus bacteraemia with or without infective endocarditis: analysis of the randomized trial comparing daptomycin with standard therapy. Journal of Antimicrobial Chemotherapy 2009; 63(5): 1034-42.

103. Richards DA, Toop LJ, Epton MJ, et al. Home management of mild to moderately severe community-acquired pneumonia: a randomised controlled trial. Medical Journal of Australia 2005; 183(5): 235-38.

104. Rodriguez-Cerrillo M, Poza-Montoro A, Fernandez-Diaz E, Romero AI. Patients with uncomplicated diverticulitis and comorbidity can be treated at home. European Journal of Internal Medicine 2010; 21(6): 553-54.

105. Rodriguez-Cerrillo M, Poza-Montoro A, Fernandez-Diaz E, Inurrieta-Romero A, Matesanz-David M. Home treatment of patients with acute cholecystitis. European Journal of Internal Medicine 2012; 23(1): e10-e13.

106. Seaton RA, MacConnachie AA. Experience with daptomycin in an infectious diseases service over 1 year: utility in an outpatient parenteral antibiotic programme. International Journal of Antimicrobial Agents 2008; 31(5): 492-97.

107. Seaton RA, Nathwani D. Outpatient and home parenteral antibiotic therapy (OHPAT) in the UK: survey of infection specialists' experience and views. Clinical Microbiology & Infection 2000; 6(7): 387-90.

108. Seaton RA, Nathwani D, Williams FL, Boyter AC. Feasibility of an outpatient and home parenteral antibiotic therapy (OHPAT) programme in Tayside, Scotland. Journal of Infection 1999; 39(2): 129-33.

109. Seaton RA, Bell E, Gourlay Y, Semple L. Nurse-led management of uncomplicated cellulitis in the community: evaluation of a protocol incorporating intravenous ceftriaxone. Journal of Antimicrobial Chemotherapy 2005; 55(5): 764-67.

110. Seaton RA, Sharp E, Bezlyak V, Weir CJ. Factors associated with outcome and duration of therapy in outpatient parenteral antibiotic therapy (OPAT) patients with skin and soft-tissue infections. International Journal of Antimicrobial Agents 2011; 38(3): 243-48.

111. Sebban C, Dussart S, Fuhrmann C, et al. Oral moxifloxacin or intravenous ceftriaxone for the treatment of low-risk neutropenic fever in cancer patients suitable for early hospital discharge. Supportive Care in Cancer 2008; 16(9): 1017-23.

112. Seetoh T, Lye DC, Cook AR, et al. An outcomes analysis of outpatient parenteral antibiotic therapy (OPAT) in a large Asian cohort. International Journal of Antimicrobial Agents 2013; 41(6): 569-73.

113. Sims AL, Baker P, Bellamy R, McMurtry IA. Outpatient parenteral antibiotic therapy in primary hip and knee arthroplasty infection managed with debridement and retention of prosthesis: a retrospective cohort study. Surgical Infections 2013; 14(3): 293-96.

114. Smego RA, Khan MA, Khowaja K, Rafique R, Datoo F. A university-sponsored Home Health Nursing Program in Karachi, Pakistan. Home Healthcare Nurse 2005; 23(11): 710-16.

115. South R. Retrospective study of teicoplanin as home continuation of hospital-initiated therapy. International Journal of Antimicrobial Agents 1998; 9(4): 219-25.

116. Stein GE, Schooley SL, Havlichek DH, Nix DE. Outpatient intravenous antibiotic therapy compared with oral linezolid in patients with skin and soft tissue infections: A pharmacoeconomic analysis. Infectious Diseases in Clinical Practice 2008; 16(4): 235-39.

117. Subedi S, Looke DFM, McDougall DA, Sehu MM, Playford EG. Supervised self-administration of outpatient parenteral antibiotic therapy: a report from a large tertiary hospital in Australia. International Journal of Infectious Diseases 2015; 30: 161-65.

118. Talcott JA, Whalen A, Clark J, Rieker PP, Finberg R. Home antibiotic therapy for low-risk cancer patients with fever and neutropenia: a pilot study of 30 patients based on a validated prediction rule. Journal of Clinical Oncology 1994; 12(1): 107-14.

For peer review only - http://bmjopen.bmj.com/site/about/guidelines.xhtml

119. Teuffel O, Amir E, Alibhai S, Beyene J, Sung L. Cost effectiveness of outpatient treatment for febrile neutropaenia in adult cancer patients. British Journal of Cancer 2011; 104(9): 1377-83.

120. Teuffel O, Cheng S, Ethier MC, et al. Health-related quality of life anticipated with different management strategies for febrile neutropenia in adult cancer patients. Supportive Care in Cancer 2012; 20(11): 2755-64.

121. Theocharis G, Rafailidis PI, Rodis D, Kontopidis I, Barbas SG, Falagas ME. Outpatient parenteral antibiotic therapy (OPAT) at home in Attica, Greece. European Journal of Clinical Microbiology & Infectious Diseases 2012; 31(11): 2957-61.

122. Thornton J, Elliott RA, Tully MP, Dodd M, Webb AK. Clinical and economic choices in the treatment of respiratory infections in cystic fibrosis: comparing hospital and home care. Journal of Cystic Fibrosis 2005; 4(4): 239-47.

123. Tice AD. Experience with a physician-directed, clinic-based program for outpatient parenteral antibiotic therapy in the USA. European Journal of Clinical Microbiology & Infectious Diseases 1995; 14(7): 655-61.

124. Tice A. The use of outpatient parenteral antimicrobial therapy in the management of osteomyelitis: data from the Outpatient Parenteral Antimicrobial Therapy Outcomes Registries. Chemotherapy 2001; 47(Suppl 1): 5-16.

125. Tice AD, Hoaglund PA, Nolet B, Mckinnon PS, Mozaffari E. Cost perspectives for outpatient intravenous antimicrobial therapy. Pharmacotherapy: The Journal of Human Pharmacology & Drug Therapy 2002; 22(2 Pt 2): 63S-70S.

126. Upton A, Ellis-Pegler RB, Woodhouse AF. Outpatient parenteral antimicrobial therapy (OPAT): A review of experience at Auckland Hospital. New Zealand Medical Journal 2004; 117: 1200.

127. Walton AL, Howden BP, Grayson LM, Korman TM. Continuous-infusion penicillin home-based therapy for serious infections due to penicillin-susceptible pathogens. International Journal of Antimicrobial Agents 2007; 29(5): 544-48.

128. White HA, Davis JS, Kittler P, Currie BJ. Outpatient parenteral antimicrobial therapy-treated bone and joint infections in a tropical setting. Internal Medicine Journal 2011; 41(9): 668-73.

129. White B, Seaton RA, Evans TJ. Management of suspected lyme borreliosis: Experience from an outpatient parenteral antibiotic therapy service. QJM 2013; 106(2): 133-38.

130. Williams DN. Home intravenous antibiotic therapy (HIVAT): Indications, patients and antimicrobial agents. International Journal of Antimicrobial Agents 1995; 5(1): 3-8.

131. Wolter JM, Bowler SD, Nolan PJ, McCormack JG. Home intravenous therapy in cystic fibrosis: a prospective randomized trial examining clinical, quality of life and cost aspects. European Respiratory Journal 1997; 10(4): 896-900.

132. Wolter JM, Cagney RA, McCormack JG. A randomized trial of home vs hospital intravenous antibiotic therapy in adults with infectious diseases. Journal of Infection 2004; 48(3): 263-68.

133. Wynn M, Dalovisio JR, Tice AD, Jiang X. Evaluation of the efficacy and safety of outpatient parenteral antimicrobial therapy for infections with methicillin-sensitive Staphylococcus aureus. Southern Medical Journal 2005; 98(6): 590-95.

134. Yadlapalli NG, Vaishnav A, Sheehan P. Conservative management of diabetic foot ulcers complicated by osteomyelitis. Wounds: A Compendium of Clinical Research & Practice 2002; 14(1): 31-35.

135. Yan YM, Singh M, Tonks K, Kavi J, Langford NJ. Delivering outpatient antibiotic therapy (OPAT) in an Acute Medical Unit. Acute Medicine 2011; 10(1): 22-25.

136. Yang A, Fung R, Brunton J, Dresser L. Outpatient parenteral antimicrobial therapy for surgery patients: A comparison with previous standard of care. The Canadian Journal of Infectious Diseases & Medical Microbiology 2014; 24(2): 74-78.

137. Yong C, Fisher DA, Sklar GE, Li S-C. A cost analysis of Outpatient Parenteral Antibiotic Therapy (OPAT): an Asian perspective. International Journal of Antimicrobial Agents 2009; 33(1): 46-51.

138. You JHS, Lee GCH, So RKH, Cheung KW, Hui M. Linezolid versus vancomycin for prosthetic joint infections: a cost analysis. Infection 2007; 35(4): 265-70.

For peer review only

For peer review only - http://bmjopen.bmj.com/site/about/guidelines.xhtml

139. Scarborough M, Bejon P, Thwaites G et al. The OVIVA Trials (oral versus intravenous antibiotics for bone and joint infection. Available at: https://www.situ.ox.ac.uk/surgical-trials/oviva-oral-versus-intravenous-antibiotics-for-bone-and-joint-infection (accessed 18 January, 2017).

140. Wye L, Brangan E, Cameron A, Gabbay J, Klein J, Pope C. Knowledge exchange in health-care commissioning: case studies of the use of commercial, not-for-profit and public sector agencies, 2011-14. Health Serv Deliv Res 2015; 3(19).

141. NHS England. NHS standard contract for specialised services for infectious diseases (adult), 2013/14. Available at: https://www.england.nhs.uk/wp-content/uploads/2013/06/b07-infec-diseases-adu.pdf (accessed 13 July 2016).

142. Chapman ALN, Seaton RA, Cooper MA et al. Good practice recommendations for outpatient parenteral antibiotic therapy (OPAT) in adults in the UK: a consensus statement. J Antimicrob Chemother 2012; 67(5): 1053-1062.

For peer review only

For peer review only - http://bmjopen.bmj.com/site/about/guidelines.xhtml

Figure 1 Flow of studies into the review

For peer review only

For peer review only - http://bmjopen.bmj.com/site/about/guidelines.xhtml

TABLE 1 Effect of OPAT on clinical success and safety

OPAT model(s) [Study ID]	Inferior	Superior	No difference	Inconclusive
**Duration of treatment (all models)**			⦿	
Outpatient attendance vs. inpatient treatment [101]			●	
Self-administration vs. inpatient treatment [40, 98, 131]			●	
General nurse vs. inpatient treatment [30, 103]			●	
Specialist nurse vs. inpatient treatment [32, 44, 81]			⦿	
**Rate of cure or improvement (all models)**		⦿		
Outpatient attendance vs. inpatient treatment [101]	●			
Self-administration vs. inpatient treatment [20]		●		
General nurse vs. inpatient treatment [30,103]			●	
Specialist nurse vs. inpatient treatment [44, 81]		⦿		
**Improved lung function in CF (all models)**				○
Self-administration vs. inpatient treatment for FEV_1 [23, 40, 98, 131]			●	
Self-administration vs. inpatient treatment for FVC [23, 40, 98, 131]				○
Self-administration vs. inpatient treatment for PEFR [23, 98]				○
**Drug-related side effects (all models)**			⦿	
Outpatient attendance vs. inpatient treatment [101]		●		
Self-administration vs. inpatient treatment [20, 40, 98, 131]			●	
General nurse vs. inpatient treatment [103]			●	
Specialist nurse vs. inpatient treatment [44, 78, 81, 109]				○
**Venous access complications (all models)**	⦿			
Self-administration vs. inpatient treatment [20, 40, 131]			⦿	
General nurse vs. inpatient treatment [103]	●			
**Hospital admission (all models)**				○
Self-administration vs. inpatient treatment [20, 131]			●	
General nurse vs. inpatient treatment [30, 103]	●			
Specialist nurse vs. inpatient treatment [32, 78, 81, 109]		⦿		
**Deaths (all models)**			●	
Outpatient attendance vs. inpatient treatment [101]	●			
Self-administration vs. inpatient treatment [20, 131]			●	
General nurse vs. inpatient treatment [103]			●	
Specialist nurse vs. inpatient treatment [32, 44]			●	

(dark shading) Reports the overall effect on an outcome for all OPAT models combined. This includes findings from studies where the OPAT model was not specified or where results for multiple models were not reported separately.

(light shading) Reports the effect only for studies that specify the model under study and report on individual findings.

Key: ● found in ≥75% studies considering outcome; ⦿ found in ≥50% studies considering outcome; ○ evidence of effect supported by <50% studies considering outcome.

For peer review only - http://bmjopen.bmj.com/site/about/guidelines.xhtml

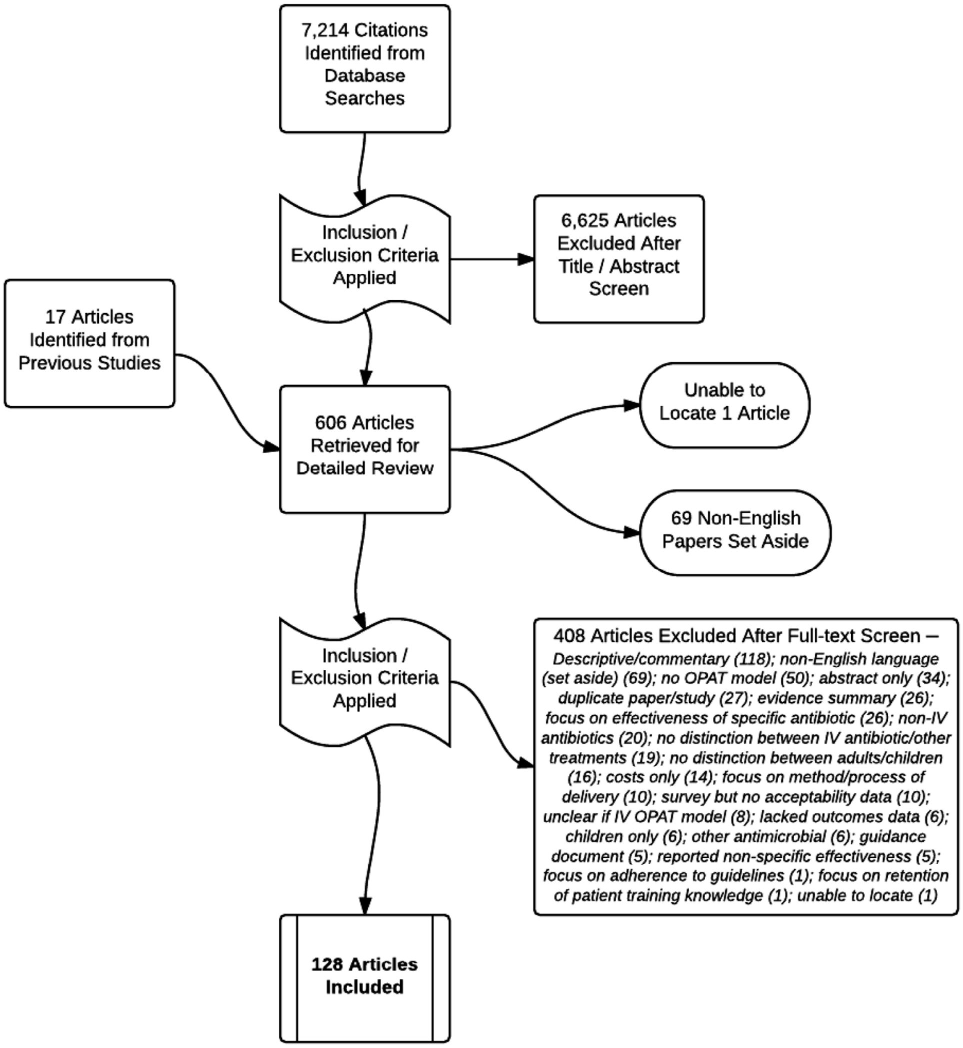

Figure 1 Flow of studies into the review
Figure 1 Flow of studies into
187x203mm (300 x 300 DPI)

APPENDIX 1

CIVAS SYSTEMATIC REVIEW:
DATABASES SEARCHED AND SAMPLE SEARCHES

DATABASE	DATES
British Society for Antimicrobial Chemotherapy	Accessed March 2015 http://bsac.org.uk/
CINAHL (EBSCOHost)	1981 to March 2015
The Cochrane Library (Wiley)	Accessed March 25, 2015
Embase Classic + Embase (Ovid)	1947 to March 25, 2015
Health Business Elite (HDAS, NHS Evidence)	1922 to March 25, 2015
Health Management Information Consortium – HMIC (Ovid)	1983 to March 25, 2015
International Pharmaceutical Abstracts (Ovid)	1970 to March 2015
Ovid MEDLINE(R)	1946 to March Week 4, 2015
Ovid MEDLINE(R) In-Process & Other Non-Indexed Citations	March 25, 2015
Research Papers in Economics – RePEc	Accessed March 2015 http://ideas.repec.org/
CEA Registry	Accessed March 2015 https://research.tufts-nemc.org/cear4/Default.aspx
Web of Science Conference Proceedings Citation Index – Science (Thomson Reuters)	1990 to March 2015

For peer review only

APPENDIX 1

Sample Search A: Studies of IV antibiotics and known models of care

Ovid MEDLINE(R) <1946 to March Week 4, 2015>

----

1 exp Anti-Bacterial Agents/ (497300)

2 Anti-Infective Agents/ (36221)

3 exp Anti-infective agents, Urinary/ (28162)

4 exp antifungal agents/ (126501)

5 or/1-4 (612854)

6 Administration, Intravenous/ (151)

7 infusions, intravenous/ (45941)

8 injections, intravenous/ (75646)

9 Home infusion therapy/ (579)

10 or/6-9 (120138)

11 ((parenteral or intravenous or IV or inject* or infusion*) adj5 (antibiotic* or anti-biotic* or antimicrobial* or anti-microbial* or antifungal* or anti-fungal* or anti-infective* or antiinfective* or antibiotherap*or anti-biotherap*)).tw. (10477)

12 (5 and 10) or 11 (20711)

13 exp Ambulatory Care Facilities/ (41613)

14 exp Delivery of healthcare/ (739029)

15 Critical Pathways/ (4202)

16 Ambulatory Care/ (34170)

17 Emergency Service, Hospital/ (39312)

18 outpatients/ (7955)

19 inpatients/ (11488)

20 day care/ (4526)

21 inpatients/ (11488)

22 hospitalization/ (66091)

23 community health services/ (25779)

24 community health nursing/ (17982)

25 home care services/ (26544)

26 home care services, hospital-based/ (1552)

27 home nursing/ (7802)

28 home infusion therapy/ (579)

29 or/13-28 (936219)

30 12 and 29 (1550)

31 ("emergency ward*" or "emergency room*" or "accident and emergency" or ED or "emergency department*" or A&E).tw. (77291)

32 outpatient*.tw. (95608)

For peer review only

APPENDIX 1

33 (home or homes).tw. (141950)

34 (self adj3 (treat* or care or regime*)).tw. (14552)

35 ((clinic or clinics) adj3 (treat* or care or regime*)).tw. (11443)

36 (community adj3 (treat* or care or regime*)).tw. (15994)

37 (ambulatory adj3 (treat* or care or regime*)).tw. (11257)

38 "district nurs*".tw. (1351)

39 ("specialist nurs*" or "nurse specialist*").tw. (3498)

40 "community nurs*".tw. (2437)

41 ((hospital* or ward or clinic) adj2 (patient or patients)).tw. (58637)

42 inpatient*.tw. (54156)

43 ("care pathway*" or "care model*" or "model* of care").tw. (6275)

44 or/31-43 (438938)

45 44 and 12 (2102)

46 "outpatient parenteral antibiotic* therapy".tw. (103)

47 "outpatient antibiotic* therapy".tw. (64)

48 (ohpat or opat).tw. (116)

49 or/45-48 (2140)

50 49 or 30 (2678)

Sample Search B: Reviews of IV antibiotic use in cellulitis or cystic fibrosis

Ovid MEDLINE(R) <1946 to March Week 4, 2015>

----

1 exp *Anti-Bacterial Agents/ or *Anti-Infective Agents/ or exp *antifungal agents/ or exp *Anti-infective agents, Urinary/ (380181)

2 *Administration, Intravenous/ or *infusions, intravenous/ or *injections, intravenous/ or *Home infusion therapy/ (5005)

3 1 and 2 (324)

4 ((parenteral or intravenous or IV or inject* or infusion*) adj5 (antibiotic* or anti-biotic* or antimicrobial* or anti-microbial* or antifungal* or anti-fungal* or anti-infective* or antiinfective* or antibiotherap*or anti-biotherap*)).ti. (1464)

5 3 or 4 (1649)

6 exp Anti-Bacterial Agents/ (498780)

7 Anti-Infective Agents/ (36330)

8 exp Anti-infective agents, Urinary/ (28211)

9 exp antifungal agents/ (126949)

10 or/6-9 (614703)

11 Administration, Intravenous/ (177)

12 infusions, intravenous/ (46068)

For peer review only

APPENDIX 1

13 injections, intravenous/ (75786)

14 Home infusion therapy/ (580)

15 or/11-14 (120429)

16 ((parenteral or intravenous or IV or inject* or infusion*) adj5 (antibiotic* or anti-biotic* or antimicrobial* or anti-microbial* or antifungal* or anti-fungal* or anti-infective* or antiinfective* or antibiotherap*or anti-biotherap*)).tw. (10513)

17 (10 and 15) or 16 (20758)

18 "outpatient* parenteral".tw. [finds more] (189)

19 "outpatient parenteral antibiotic* therapy".tw. (103)

20 "outpatient antibiotic* therapy".tw. (64)

21 (ohpat or opat).tw. (116)

22 Cellulitis/ (6046)

23 cellulitis.tw. (5439)

24 Cystic Fibrosis/ (27005)

25 (cystic adj fibrosis).tw. (29491)

26 "fibrocystic disease*".tw. (807)

27 17 or 19 or 20 or 21 (20796)

28 or/22-26 (43980)

29 (5 or (18 or 19 or 20 or 21)) and 28 [major headings or title occurence of IV Antibiotic terms AND MINOR Cell/CF terms] (125)

30 17 and 28 [most sensitive search] (1037)

31 limit 30 to "review articles" (150)

32 29 or 31 (254)

For peer review only

APPENDIX 2

CIVAS SYSTEMATIC REVIEW:
QUALITY ASSESSMENT – OBSERVATIONAL STUDIES

**Population** (tick one box)	**PAPER ID:**	
1. Sample size:		
a) Strong – power calculation provided	✪	☐
b) Strong – included all OPAT patients	✪	☐
c) Strong – appropriate size for qualitative study	✪	☐
d) Insufficient – no power calculation or selective/small study		☐
e) Unclear – not enough information provided		☐
**Methodology:** (tick one box)		
2. Method to ascertain data:		
a) Strong – not open to selection bias*	✪	☐
b) Strong – appropriate qualitative methodology	✪	☐
c) Moderate – open to some bias but appropriate method used	○	☐
d) Insufficient – inappropriate method used to ascertain data		☐
e) Unclear – not enough information provided		☐
**Analysis:** (tick one box)		
3. Data analysis:		
a) Strong – reports statistically significant differences in outcomes	✪	☐
b) Strong – relevant qualitative analytic techniques	✪	☐
c) Moderate – used only comparative analysis with no statistical testing of differences	○	☐
d) Moderate – reported only descriptive statistics	○	☐

* Systematic differences between comparison groups in terms of selection, prognosis or treatment; differences in group characteristics.

ASSESSMENT OF THE RISK OF BIAS:

**Potential bias:**	Selection		Analysis	
	Method		**ASSESSMENT**	

For peer review only

TABLE S1: Clinical effectiveness of outpatient parenteral antibiotic treatment

ID	Author (country)	Study design	OPAT Model(s)	OPAT group(s)†	Comparator(s)†	Findings
**COMPARATOR STUDIES**						
20	Bedi et al (UK)	Cohort	SA	Home therapy (domiciliary IV); 52 patients (median age 61); 58% male<br>Home therapy (early supported discharge); 23 patients (median age 65); 35% male	Inpatient therapy; 36 patients (median age 71); 36% male	There was resolution of infection in 76% of the inpatient group, 80% of the ESD group and 80% of the domiciliary IV group.<br>Within group comparisons showed significant ($p<0.05$) improvements in FEV1, FVC, incremental shuttle walking test, 24-h sputum volume, sputum bacterial clearance, and parameters of inflammation in all groups.
23	Bradley et al (UK)	Observational (retrospective)	SA	Home therapy; 8 patients (mean age 26); 50% male	Combined therapy; 14 patients (mean age 24); 57% male<br>Inpatient therapy; 29 patients (mean age 24); 83% male	On completion of therapy, there was no significant difference between the groups in FEV_1 (51 vs. 66 vs. 60), FVC (63 vs. 73 vs. 71) or FEF_{25-75} (32 vs. 43 vs. 29).<br>PEFR was significantly greater in the OPAT and Combined groups than in the Hospital group (84 vs. 73 vs. 67; $p<0.05$).<br>When change was expressed as a percentage of the baseline value, FEF_{25-75} showed a large improvement in each group (approx. 15-48%). There was a significantly greater improvement in FVC, FEV_1 and FEF^{25-75} in the Hospital group than in the other two groups.
30	Corwin et al (New Zealand)	RCT	GN	Home therapy; 98 patients (mean age 55); 62% male	Inpatient therapy; 96 patients (mean age 48); 73% male	There was no significant difference in mean days to no advancement of cellulitis between Home and Hospital groups (1.50 days vs. 1.49 days; 95% CI -0.3 to 0.28)).<br>This was also the case for days on IV antibiotics (HR 0.84, 95% CI 0.63-1.12; p=0.23), and days to discharge (HR 0.93, 95% CI 0.70-1.23; p=0.60).
32	Dall et al (USA)	Before and after	SN	Home therapy; 92 pneumonia patients (97% aged >50 years); 44% male / 64 cellulitis patients (mean age 51); 70% male	Inpatient therapy; 10728 pneumonia patients (83% aged >50 years); 51% male / cellulitis patients (no numbers provided)	Most OPAT cellulitis patients (45/64; 70%) required only two day's treatment (range 2-6d). Among pneumonia patients, 58% (53/92) required ≤3 days of treatment (range 2-9d).<br>Median length of stay was shorter for OPAT pneumonia patients than for historical comparators (0.07-1.07 days vs. 2-4 days vs. 6-9 days; $p<0.05$). This was also the case for SSTI patients ((0.1 day vs. 1.3 days; $p<.001$).
39	Escalante et al (USA)	Clinical trial (unspecified)	Not specified	IV therapy; 43 patients (median age 39); 42% male	Oral therapy; 40 patients (median age 52); 50% male	There was a response in 41/43 (95%) patients receiving IV therapy compared with 35/40 (88%) patients receiving oral treatment (p=0.19).<br>Preliminary results of a second trial (altered oral regime) showed a response rate of 87% (78/90 patients) in the IV arm and 90% (80/89 patients) in the oral arm.

SUPPLEMENTARY TABLES

ID	Author (country)	Study design	OPAT Model(s)	OPAT group(s)†	Comparator(s)†	Findings
40	Esmond et al (UK)	Controlled trial	SA	Home therapy; 15 patients (mean age 27); 40% male	Inpatient therapy; 15 patients (mean age 23); 60% male	There was no significant difference between groups in mean duration of treatment: Home group 14 days (range 10-18d), Hospital group 15 days (range 10-25d).<br>Improved lung function was significantly greater in the Hospital group than in the Home group (FEV_1 2.0 vs. 5.2; p=0.05 and FVC 2.1 vs. 12.3; p=0.01). When change in the groups was directly compared, only the difference in FVC was significant (p=0.01).
44	Fernandez-Aviles et al (Spain)	Case-control	SN	Home therapy; 50 patients (mean age 47); 62% male	Inpatient therapy; 50 patients (median age 50); 54% male	There was no significant difference between Home and Hospital patients in relation to median duration of treatment (8 days vs. 9 days).<br>Median duration of fever was significantly shorter in Home patients (2 days vs. 5 days; p=0.00003).
75	Martone et al (USA)	Observational (retrospective)	Not specified	OPAT; 539 patients (57% aged >50 years); 56% male	Inpatient therapy; 410 patients (63% aged >50 years); 45% male	Median duration of OPAT only treatment was 17 days (range 3-144d). Median duration of hospital treatment was 7 days (range 1-153d).<br>Success rates were higher in OPAT (510/539) than in hospital (354/41) patients (94.6% vs. 86.3%; p<0.001).
81	Montalto & Dunt (Australia)	Observational (retrospective)	SN (HHU)	HHU therapy; 55 cellulitis patients (mean age 52); 60% male / 14 pyelonephritis patients (mean age 30); 0% male	Inpatient therapy; 22 cellulitis patients (mean age 51); 73% male / 10 pyelonephritis patients (mean age 35); 30% male	Mean duration of stay was lower for HHU cellulitis patients (6.00 days vs. 8.55 days; 95% CI 0.24-4.85) but not for pyelonephritis patients (4.57 days vs. 4.00 days; 95% CI -1.86-0.72).<br>Full recovery was expected in 51/55 HHU cellulitis patients (93%); the remaining four showed recovery back to a stable pre-existent condition. Recovery was expected in all 22 Hospital patients.<br>Recovery was expected in 13/14 HHU pyelonephritis patients (93%), and in 9/10 Hospital patients (90%). The remaining patient in each group was expected to return to a stable pre-existent condition.<br>Mean time to febrifuge was lower for HHU cellulitis (1.96 days vs. 2.00 days) and pyelonephritis patients (1.79 days vs. 2.40 days), although the difference was not statistically significant.
98	Pond et al (UK)	Case-control (retrospective)	SA	Home therapy; 25 patients (mean age 21); 56% male	Inpatient therapy; 25 patients (mean age 22); 56% male	Mean duration of treatment was similar in the two groups (OPAT 14.1 days vs. Hospital 16.7 days).<br>There was no difference between the OPAT and Hospital groups in relation to adjusted mean improvement in FVC (0.567 vs. 0.644), FEV1 (0.456 vs. 0.403), PEFR (66.1 vs. 57.5) or other outcome variables. The only variable to show a significant difference was total white cell count (-3.64 vs. -4.72; p<0.05).

SUPPLEMENTARY TABLES

ID	Author (country)	Study design	OPAT Model(s)	OPAT group(s)†	Comparator(s)†	Findings
101	Rapoport et al (Multinational)	RCT	OP	Outpatient therapy; 40 patients (median age 45); 52% male	Inpatient therapy; 44 patients (median age 48); 32% male	Median duration of treatment was similar for both OPAT and inpatients groups (6.0 days vs. 6.3 days).<br>Treatment was successful in 34/38 OPAT patients (89%) and 40/42 inpatients (95%).
102	Rehm et al (USA)	RCT	SA; SN; (also skilled nursing facility; assisted-living; nursing home; rehab centre)	Home therapy; 103 patients (median age 50); 58% male	Inpatient therapy; 97 patients (median age 54); 61% male	OPAT patients received longer courses of antibiotics than hospital patients (mean 25.4 days vs. 13.5 days; p<0.001). OPAT patients received a mean of 14.9 days of therapy outside the hospital setting.<br>There was a higher rate of clinical success among OPAT patients (89/103; 86.4%) than hospital patients (54/97; 55.7%) (p<0.001).
103	Richards et al (New Zealand)	RCT	GN	Home therapy; 24 patients (mean age 50); 54% male	Inpatient therapy; 25 patients (mean age 50); 52% male	Median time to discharge was 4 days (range 1-14d) in the Home group and 2 days (range 0-10d) in the Hospital group (p=0.004).<br>There was no difference in the number of days on IV antibiotics (3 days vs. 2 days; p=0.22).<br>At 2 weeks, there was no difference in patient-rated symptoms. There was a significant difference in sleep disturbance (Home median “occasional”; Hospital median “never”; p<0.01), but this did not persist at 6 weeks. There was no difference in time to resolution of fever, tachycardia or tachypnoea.
109	Seaton et al (UK)	Before and after	SN	Standard OPAT; 230 patients (median age 49); 52% male	Nurse-led OPAT; 112 patients (median age 50); 61% male	Total median duration of IV therapy was reduced from 5 days (range 1-37d) to 4 days (range 1-23d) (p=0.01)<br>Median duration of outpatient therapy was reduced from 4 days (range 1-37d) to 3 days (range 1-22d) (p=0.02).<br>Cure or improvement was similar for pre (225/228) and post-protocol (106/108) patients (99% vs. 97%).
111	Sebban et al (France)	RCT	OP	IV therapy; 47 patients (mean age 52); 40% male	Oral therapy; 49 patients (mean age 52); 31% male	Success was observed for 34/46 (73.9%) patients in the IV group and 38/48 (79.2%) patients in the Oral group (calculated risk difference was 5.3%; 95% CI 11.9 to 22.4%).
116	Stein et al (USA)	RCT	OP	IV therapy; 10 patients (mean age 54); 60% male	Oral therapy; 10 patients (mean age 53); 40% male	Average number of OPAT doses was eight (range 1-30). Average number of Oral doses was 19 (range 9-40).<br>Cure and improvement rates were higher for Oral group patients (10/10; 100%) than for OPAT patients (6/10; 60%), although this was not statistically significant (p=0.09).
122	Thornton et al (UK)	Observational (retrospective)	Not specified	Home therapy; 47 patients (mean age 26); 36% male	Combined therapy; 18 patients (mean age 25); 61% male<br>Inpatient therapy; 51 patients (mean age 26); 59% male	Mean treatment duration was 63 days for Home patients 54 days for Hospital patients and 66 days for Combined patients.<br>Mean FEV_1 improvement from baseline ‘‘best’’ was significantly higher for Hospital patients (mean diff. 4.6%, 95% CI 1.8-7.4; p=0.001).

SUPPLEMENTARY TABLES

ID	Author (country)	Study design	OPAT Model(s)	OPAT group(s)†	Comparator(s)†	Findings
						More Hospital (41/236; 17%) than Home courses (18/199; 9%) were classified as effective (p=0.001).<br>At the ≤0% level, there was a mean decline in FEV_1 for Home patients after 1 year, but an improvement for Hospital patients (mean diff. 10.1%, 95% CI 2.9-17.2; p=0.003). The number of patients in whom treatment was classified as effective was higher in the Hospital (n=30; 59%) than Home group (n=20; 43%). When effectiveness was defined as an FEV_1 decline of ≤2%, the difference became statistically significant (43% vs. 63%; p=0.045).
131	Wolter et al (Australia)	RCT	SA	Home care; 13 admissions (median age 22); 28% male	Inpatient therapy; 18 admissions (median age 22); 39% male	There were no significant differences in the duration of treatment or use of antibiotics. Median duration of treatment was 12 days (range 10-24d) in the Home group and 11 days (range 7-26d) in the Hospital group (p=0.2).<br>Home patients had fewer investigations performed than inpatients (p=0.002).<br>There was no significant difference between groups in improved lung function (FEV_1 p=0.27; FVC p=0.30).<br>There was no significant difference in time to next admission between the groups (p=0.68).
132	Wolter et al (Australia)	RCT	Not specified	Home care; 44 admissions (median patient age 43); 45% male	Inpatient therapy; 38 admissions (median patient age 49); 34% male	Median duration of treatment was similar between the groups (Home 11.5 days, range 3-57d; Hospital 11.0 days, range 4-126d; p=0.002).<br>Median number of investigations was significantly lower in the Home group (12.5 vs. 19.0; p<0.001).<br>There was no difference in numbers with an "Improved" outcome following treatment (Home 84.1% vs. Hospital 92.1%; p=0.32).<br>Average time to the next admission was longer in the Hospital group (12 days vs. 32 days; p=0.006).
136	Yang et al (Canada)	Case-control	Not specified	OPAT; 21 patients (mean age 59); 57% male	Previous standard care; 21 patients (mean age 59); 57% male	There was a better overall cure rate in the OPAT group (61.9% vs. 57.1%, p=0.1),<br>For a composite end point of clinical success (i.e. combined cures and controlled/improved cases) the results were 95% in the OPAT group and 86% in the previous care group (p=0.62). There were three (14.3%) treatment failures in the control group and one (4.8%) in the OPAT group
137	Yong et al (Singapore)	Case-control (retrospective)	OP; SA	OPAT ; 69 patients (mean age 53); 53% male	Inpatient therapy; 93 patients (mean age 56); 51% male	Average length of OPAT only care was 24.3 days (range 4-93d). Mean duration of treatment for OPAT patients was 42.5 days (range 4-145d), significantly longer than for hospital patients (mean 19 days; range 4-183d) (p<0.001).<br>At the end of the 4-month follow-up period, 59/72 OPAT

SUPPLEMENTARY TABLES

ID	Author (country)	Study design	OPAT Model(s)	OPAT group(s)†	Comparator(s)†	Findings
						episodes (81.9%) were considered cured compared with 75/93 hospital patients (80.6%).
**OPAT ONLY STUDIES**						
11	Al Ansari et al (Bahrain)	Case-series (retrospective)	OP	101 patients; 57% male	—	Average duration of treatment ranged from three to 10 days. Total cure was achieved in 97 (96%) treated patients. Two patients were lost to follow-up with the OPAT clinic.
14	Anand et al (USA)	Case-series	Home IV therapy (unspecified)	52 patients	—	Patients showed a significant improvement in all symptoms post-treatment (p<0.001).
15	Angel (USA)	RCT	SA	62 patients (mean age 71); 53% male	—	Mean length of inpatient treatment was 3.6 days less than expected from DRGs. Documented cure was seen in 35/50 evaluable patients (70%), while 14 (28%) had improved. There was one clinical failure. Bacteriologic eradication was seen in 16/28 (57%) evaluable patients; 10 others (36%) were clinically improved (no culture could be obtained). Two patients had persistent pathogens.
18	Barr et al (UK)	Observational (retrospective)	OP; SA; SN; GN	2233 patients (median age 51); 58% male	—	Median duration of treatment was 5 days (range <1-328d). Most patients were cured (1,501/2233; 67%) or improved (n=562; 25%). Small numbers showed no change (n=52; 2%) or deteriorated (n=91; 4%).
21	Berman & Johnson (USA)	Case-series (retrospective)	SA	221 patients (median age 41); 66% male	—	Median duration of treatment was 18 days (range 3-307d). OPAT was successful in 283/302 episodes (94%). Treatment failed in 19 episodes (6%) of which 16 (84%) required hospitalisation.
22	Bernard et al (France)	Observational (?)	SA; SN;	39 patients (mean age 44); 64% male	—	Mean duration of treatment was four months (range 1.5 to 12m). Twenty-eight of 30 patients (93%) available for follow-up were considered cured with a mean delay of 24 months after completion of therapy. There was one clinical failure and one relapse within one month after treatment.
27	Chan et al (Singapore)	Observational	Not specified	109 patients (mean age 57); 66% male	—	Average duration of IV therapy was 32 days (range 11-98d). Mean length of OPAT was 16 days (range 2-54d). Eighty of 109 patients (74%) achieved a clinical response at four weeks. All patients achieved cure.

SUPPLEMENTARY TABLES

ID	Author (country)	Study design	OPAT Model(s)	OPAT group(s)†	Comparator(s)†	Findings
28	Chapman et al (UK)	Observational (retrospective)	SA; Infusion centre	334 treatment courses (mean patient age 46); 59% male	—	Mean duration of treatment for SSTI (n=198) was 4.9 days (range 1-46d). Mean duration of treatment for other infections (n=136) was 22.5 days (range 1-163d).<br>A total of 291/334 patients (87%) were cured or improved on completion of therapy; 11 were recorded as "no change".<br>Sixty two records of SSTI patients were selected at random for follow-up data. Mean duration of follow-up was 27.8 days (range 4-122d); 61 (98.4%) were cured or improved.
31	Cox et al (USA)	Observational (retrospective)	SA	205 patients (mean age 59); 99% male	—	Median duration of treatment was 22 days (range 2-105d) for older adults (≥60 years) and 29.5 days (range 4-450d) for younger adults.<br>A total of 54/231 (23%) courses resulted in cure at the end of home IV treatment, while 159 courses (69%) were deemed to have stable or improved infection. In 16 courses (7%) treatment was considered a failure (in all cases attributed to a difficult-to-treat infection rather than adverse effects).
33	Dalovisio et al (USA)	Observational (retrospective)	SA; SN	62 patients (mean age 69)	—	Average duration of treatment was 23.4 days.<br>Average number of home care visits per course was 18.8.<br>The majority of treatment courses (60/66; 91%) met the goals of therapy.
34	Dargan et al (Canada)	Case-series (retrospective)	OP; Home therapy (unspecified)	66 patients (mean age 57); 50% male	—	Mean total duration of therapy was 32.8 days (range 3-169d). Mean duration of OPAT treatment was 27.3 days (range 2-169d).<br>Most patients (56/66; 85%) had a successful outcome; 10 patients experienced treatment failure.
36	Donald et al (Australia)	Case-series (retrospective)	SN	124 patients (aged 16-97); 60% male	—	Mean duration of treatment was 6.24 days.<br>The majority of patients were treated successfully (105/124; 85%).
38	Duncan et al (UK)	Observational (retrospective)	Not specified	55 completed OPAT patients (median age 59); 80% male / 25 failed OPAT patients (median age 67); 60% male	—	Median duration of OPAT was 28 days (IQR 20-38 days); all patients had a prior period of inpatient management (median 22 days, IQR 14–30 days).<br>OPAT failure occurred in 25/80 (31.3%) episodes. Increased probability of failure was associated with co-existing cardiac or renal failure (OR 7.29, 95% CI 1.84-29.66, p=0.005), specialist referral (OR 0.25, 95% CI 2.01-37.47, p=0.00), and use of Teicoplanin (OR 8.69, 95% CI 0.06-1.11, p=0.068).
41	Esposito et al (Multinational)	Observational (retrospective)	OP; SA; SN; GN; Emergency room; Infusion centre	9826 US registry patients (aged 1-80+); 57% male / 981 UK registry patients (aged 11-80+); 63% male / 620 Italian registry	—	Mean duration of treatment was longer in Italy (56.0 days) compared with the USA (22.5 days) and UK (19.9 days).<br>Most patients (10629/11427; 93%) achieved clinical cure or improvement (USA 93%; UK 97%; Italy 95%).

SUPPLEMENTARY TABLES

ID	Author (country)	Study design	OPAT Model(s)	OPAT group(s)†	Comparator(s)†	Findings
				patients (aged 1-80+); 57% male		
42	Esposito et al (Italy)	Observational (retrospective)	OP; SA; Visit by doctor or nurse (also care facility)	239 patients (aged 11-80+); 62% male	—	Mean duration of treatment was 71.2 days (SD 39.3d).<br>Clinical results were satisfactory, with only 20 failures observed at the end of therapy (8.4%).<br>Of 120 patients available for follow-up (30 days after end of therapy), 72 (60%) were cured and 35 (29%) were improved.
43	Esposito et al (Italy)	Observational	OP; SA; Visit by doctor or nurse (also care facility)	176 patients (aged >65 years); 47% male	—	At the end of therapy, almost 90% of cases were as cured or improved (156/176).<br>Seventy one of 176 patients were available for follow-up (30 days after the end of therapy); cure or improvement was recorded in 81% (56/71).
45	Goodwin et al (Canada)	Case-series	SN; GN	2405 patients (mean age 46); 55% male	—	Duration of treatment ranged from one to >42 days (longest was 209 days).<br>Five patients had a resolution of their infection earlier than expected
47	Graninger et al (Austria)	Case-series	OP	54 patients	—	Mean duration of treatment for osteomyelitis was 62 days (range 28-150d).<br>Cure was achieved in 18 (41%) and improvement in 19 (43%) of 44 patients. Seven patients failed to respond even after the minimum treatment period of 1 month (range 25-84d).<br>Average duration of treatment for endocarditis was 49 days (range 28-88d).<br>Eight of 10 patients were cured (80%), and there were two treatment failures.
48	Grayson et al (Australia)	Case-series	GN	20 patients (mean age 58)	—	Mean duration of treatment was 26 days (range 11-44d).<br>Eighteen of 20 patients (90%) were clinically and microbiologically cured after therapy.
49	Gross et al (USA)	Case-series (retrospective)	Home IV therapy (unspecified)	13 patients (mean age 38); 100% male	—	There were no treatment failures.
51	Hindes et al (USA)	Case-series	SA	48 patients (mean age 65)	—	Mean duration of therapy was 22 days.<br>All patients (48/48) had eradication or arrest of their infection.

SUPPLEMENTARY TABLES

ID	Author (country)	Study design	OPAT Model(s)	OPAT group(s)†	Comparator(s)†	Findings
52	Hitchcock et al (UK)	Case-series	OP; SA; GN; Private home healthcare company	273 patients (mean age 60); 54% male	—	Mean length of treatment was 24 days (range 1-165d). In 278/303 episodes (92%), patients were successfully treated as per plan.
53	Ho et al (Singapore)	Case-series	OP	29 patients (median age 41); 90% male	—	Median length of hospital stay prior to OPAT was 15 days (range 2-48d) Median duration of treatment was 18 days (range 1-85d). All but one patient (28/29; 96.6%) completed the intended duration of OPAT therapy.
54	Htin et al (Australia)	Case-series (retrospective)	SN	68 patients (median age 68); 87% male	—	Median duration of antimicrobial therapy was 24 days (range 4 to 42 days). Treatment success rate was 94% (64/68). There were two relapses.
55	Huminer et al (Israel)	Case-series	SA; SN (also nursing home; kibbutz)	37 patients (mean age 64); 57% male	—	Mean duration of treatment was 26.2 days (range 14-49d). Cure after a first course of treatment was documented in 34/37 patients (92%).
56	Johansson et al (Sweden)	Case-series	SA	11 patients (mean age 51); 73% male	—	Median duration of OPAT was 4 days (range 1-12 days). Patients received therapy during 16 episodes of neutropenic fever. None of the patients developed recurrent fever.
59	Kieran et al (Ireland)	Case-series (retrospective)	SA; Nurses attached to commercial company	56 patients (median age 50); 57% male	—	Median duration of treatment was 16 days (range 2-84d). Clinical cure was documented in 52/56 patients (93%). There was no significant difference in outcomes between self-administered courses (n=48) and those given by a nurse (n=12); (p=0.61).
60	Krauth et al (Worldwide)	Literature review	Not specified	11 studies	—	Only one of seven studies evaluating efficacy of home IV therapy for CF found significant differences between home and inpatient care with regard to short-term outcomes such as VC% and FEV_1% etc. The only randomised study supported the (short-term) equivalence of home care and inpatient therapy. One trial performed under everyday conditions found that home care therapy produced significantly poorer VC and FEV_1 results.
62	Lai et al (USA)	Case-series (retrospective)	SA; SN	333 patients (mean age 62); 98% male	—	Mean duration of treatment was 21.1 days (range, 0-88 days; interquartile ratio, 9-30 days). Overall completion rate was 79.6% (313/393 courses). Factors associated with non-completion were bacteraemia (OR 1.82; p=0.040), concomitant CHF (OR 1.64; p=0.051), and concomitant end stage renal disease (ESRD) (OR 2.59; p=0.015). Only ESRD remained significant when the three variables were combined in a multivariable model (OR 2.20;

SUPPLEMENTARY TABLES

ID	Author (country)	Study design	OPAT Model(s)	OPAT group(s)†	Comparator(s)†	Findings
						p=0.047).
64	Larioza et al (USA)	Case-series (retrospective)	Home infusion company	43 patients (56% aged >50 years); 67% male	—	Patients were treated for 4-8 weeks; duration of OPAT was 70% for patients with surgery and 80% for patients without (p=0.0561).<br>All patients survived for one year following therapy without infective endocarditis relapse (43/43).
65	Larioza et al (USA)	Case-series (retrospective)	Home infusion company	33 patients (76% aged ≥50 years); 61% male	—	Mean duration of treatment was 29 days.<br>Thirty one of 33 patients (94%) were successfully treated.
69	Lillie et al (UK)	Case-series	SN	98 patients (mean age 55); 61% male	—	Average duration of treatment was 6.3 days.<br>Elevated baseline CRP (p=0.002), male sex (p=0.004), prolonged duration of symptoms prior to OPAT (p=0.001) and higher antibiotic dose (p=0.006) were associated with prolonged treatment via OPAT.
71	Lopardo (Argentina)	Case-series (retrospective)	OP; SA; SN	48 patients (median age 55); 62% male	—	Clinical and microbiologic cure was achieved in 100% of cases (48/48).
72	Mackintosh et al (UK)	Case-series (retrospective)	OP; SA; SN	198 patients (aged <40-89); 64% male	—	Success rate following initial OPAT was 86.4% (171/198), ranging from 71.8% for diabetic foot or stump infection to 100% for metalwork-related infection.<br>Median duration of follow-up was 60 weeks (range 6-104w). A total of 59/198 patients (29.8%) failed initial treatment, re-presented with a recurrence or relapse of their infection, or died during the follow-up period. Of those who failed, 55 (93%) did so within 12 months.
77	Mauceri (USA)	RCT	SA	27 patients	—	Mean duration of OPAT treatment was 30.50 days. Mean duration of prior hospitalisation was 12.17 days (1.77 days longer than allotted by DRGs).<br>Cure was achieved in 11/18 evaluable patients (61%) and improvement in four patients (22%).<br>Bacteriologic success rate was 79% (11/14 patients).
79	McMahon et al (Australia)	Observational	SN	40 patients (mean age 59 (M), 49 (F)); 75% male	—	Mean duration of treatment was 47.7 days, with the mean inpatient treatment duration being 23.5 days (range 8-75d), and the mean HITH treatment duration being 24.2 days (range 2-63d).<br>Cure was achieved in 37/40 (93%) patients, with the three remaining patients considered as failures.

SUPPLEMENTARY TABLES

ID	Author (country)	Study design	OPAT Model(s)	OPAT group(s)†	Comparator(s)†	Findings
80	Mohammadi et al (USA)	Case-series (retrospective)	SA; SN	190 patients (mean age 63); 98% male	—	Median duration of OPAT was 30 days (range 5-56 days). The overall cure rate for all infections treated was 78% at end of treatment, decreasing to 58% at 90 days post-OPAT (p<0.001).
83	Montalto (Australia)	Case-series (retrospective)	SN (HHU)	133 patients (mean age 46); 52% male	—	Mean duration of HHU stay was 4.8 days (range 1-28d). Full recovery was expected in 132/133 patients (99%).
85	Morales & von Behren (Puerto Rico)	Case-series	SA	22 patients (mean age 33-41); 82% male	—	Mean length of inpatient treatment was 4.5-6.5 days shorter than allotted by DRGs. On completion of treatment, 19/20 evaluable patients (95%) were cured or improved. Bacteriologic eradication was seen in 15/17 (88%) evaluable patients. There were no super infections or reinfections.
88	Murray et al (Canada)	Observational	Emergency department	75 patients (mean age 48); 57% male	—	Two of 29 patients in the oral group failed treatment (6.8%), compared with 12/46 patients in the IV group (26%).
89	Nathwani et al (UK)	Case-series	OP; SA	101 patients	—	Most patients were cured or improved (95/101; 94%), while two patients were unchanged (4%), and the remaining four were worse (4%).
90	Nathwani (UK)	Case-series	OP; SA; SN	125 patients	—	Mean duration of treatment was 5.32 days. The majority of patients (123/125; 98%) were cured or improved. Two patients were worse and required surgery.
91	Nathwani et al (UK)	Observational (retrospective)	OP; SA	50 patients	—	Mean duration treatment was 37.7 days. All patients 50/50 were deemed improved or cured at the end of treatment.
93	Parker et al (UK)	Observational	OP; SA	29 patients; 38 GP practices	—	Of the 29 enrolled patients, 23 (79%) had complete resolution of their symptoms. The remaining six were indeterminate.
94	Partridge et al (UK)	Case-series (retrospective)	OP; SA	34 patients (mean age 55); 79% male	—	Median duration of treatment was 27 days (range 7-65d). Most episodes (34/36; 94%) were treated successfully, with no evidence of recurrence at a median of 30 months following completion of OPAT (range 6-57m).

SUPPLEMENTARY TABLES

ID	Author (country)	Study design	OPAT Model(s)	OPAT group(s)†	Comparator(s)†	Findings
95	Patanwala et al (USA)	Decision tree analysis	SN	41 patients (mean age 53); 44% male	—	Cure was documented in 18/41 patients (44%) and presumed in 11/41 patients (27%). Twelve patients were treatment failures.
96	Perez-Lopez et al (Spain)	Observational	SN	145 patients (mean age 68); 48% male	—	Mean duration of treatment was 8.8 days (range 2-60d).<br>Definitive discharge was given to 83/90 (92%) elderly group patients (≥70 years) due to satisfactory clinical evolution (no data reported for younger patients).
99	Poretz (USA)	RCT	SA	238 patients (mean age 45-52); 57% male†	—	Of 211 evaluable patients, 158 (75%) were cured and 43 (20%) were improvement. Response was unsatisfactory in the remaining 10 patients (5%).<br>Bacteriologic eradication was seen in 125/134 (93%) evaluable patients. There was persistence in 8 patients (6%).
100	Poretz (USA)	RCT	SA	130 patients (mean age 45); 59% male†	—	For patients with initial inpatient treatment, length of stay was 2.4 days shorter than allotted by DRGs. Mean duration of treatment completed entirely at home was 10.4 days.<br>Clinical cure or improvement was seen in 115/118 (98%) evaluable patients.<br>Bacteriologic eradication was noted in 78 (94%) of the 83 evaluable patients.
104	Rodriguez-Cerrillo et al (Spain)	Case-series	SN (HHU)	24 patients (mean age 73)	—	Mean duration of Hospital at Home Unit (HHU) stay was 9 days.<br>All patients had a favourable clinical outcome (24/24).
105	Rodriguez-Cerrillo et al (Spain)	Case-series	SN (HHU)	25 patients (mean age 59); 48% male	—	Mean duration of HHU stay was 11 days.<br>All patients (25/25) had a favourable clinical outcome.
106	Seaton & MacConnachie (UK)	Case-series (retrospective)	SA; Other (unspecified)	19 patients (mean age 55); 53% male	—	Average duration of OPAT was 34 days (range 3-128d).<br>Twelve of 19 patients (63%) were cured, while four (21%) were improved).
110	Seaton et al (UK)	Observational (retrospective)	OP	963 patients (median age 48); 59% male	—	Median duration of treatment was 3 days (IQR 2-5d).<br>The success rate (no progression of infection, no re-admission and no significant adverse events) was 87.1% (839/963 patients).<br>Progression of infection was seen in 27/963 patients (2.8%).

ID	Author (country)	Study design	OPAT Model(s)	OPAT group(s)†	Comparator(s)†	Findings
112	Seetoh et al (Singapore)	Cohort	OP; SA; SN	2,229 patients (median age 56); 64% male	—	Treatment was completed as planned in 1,874 (84.1%) of 2,229 episodes.<br>Median time to treatment completion was 16 days (IQR 8-27 days).
113	Sims et al (UK)	Case-series (retrospective)	OP	14 patients (mean age 63); 71% male	—	Average duration of OPAT treatment was 58 days.<br>Overall, 11/14 joints (79%) were salvaged. Of the three failures, one patient improved initially but had a recurrence 5-months later (different organism identified recurrence), one had an excellent initial response but infection recurred 18-months later, and one developed infection with an atypical organism 3-years after primary surgery.
115	South (UK)	Case-series (retrospective)	OP; SA; Nurse (unspecified)	57 patients (mean age 41); 77% male	—	Median duration of non-inpatient treatment was 5 days (range 1-14d) for haematological malignancies, and 24 days (range 9-78d) for bone and joint infections.<br>Cure or improvement was achieved in 60/64 evaluable episodes (94.8%; 95% CI 88.2-99.8). There were two clinical failures (both Hickman line infections).<br>Bacteriological success was achieved in 33/40 evaluable episodes (82.5%; 95% CI 71.4-94.6).
117	Subedi et al (Australia)	Case-series	SA; SN	144 patients (median age 55); 74% male	—	Median duration of OPAT treatment was 22 days (range 4-106 days).<br>Overall cure rate was 93% (140 episodes).
118	Talcott et al (USA)	Observational (pilot)	SA; SN	30 patients (median age 38); 43% male	—	Median duration of treatment was 3.5 days (range 1-24d).<br>Twenty-one of 30 patients (70%) completed home therapy without complication or readmission. Medically eligible patients who did not enrol in the trial had a shorter duration of neutropenia (median 4 days vs. 6 days; p<0.005) than enrolled patients.
121	Theocharis et al (Greece)	Case-series (retrospective)	SN	91 patients (mean age 85); 31% male	—	Cure rate was 72.5% (66/91).<br>Mean duration of treatment was 4.7 days (range 1-18d).
123	Tice (USA)	Case-series	OP; SA; SN	538 patients (mean age 45); 52% male	—	Clinical improvement was recorded in 484/491 patients who could be evaluated, (98.6%); failure was recorded in seven patients.<br>Eradication was reported in 244/265 cases (92%) where bacteriological outcome could be assessed. There was persistence in 17 cases (6.4%) and superinfection in four (1.5%).

ID	Author (country)	Study design	OPAT Model(s)	OPAT group(s)†	Comparator(s)†	Findings
124	Tice (USA)	Observational (retrospective)	OP; SA; SN; Infusion centre.	500 patients	—	Most patients (269/424; 56%) were treated for at least four weeks, although treatment duration varied from <1 week to 11 weeks.<br>Most patients assessed for clinical outcome were improved (259/266; 97%). There were failures in three patients and no change in four others. Most failures occurred in the first few months (65% in ≤3m); the majority (95%) in the first year.
126	Upton et al (New Zealand)	Case-series	SA	100 patients (mean age 51); 59% male	—	Median duration of treatment was 22 days (range 3-160d).<br>Cure was achieved in 94/107 treatment courses in 88 patients (88%). Ten patients relapsed, one did not respond, and one died.
127	Walton et al (Australia)	Case-series	SN	35 patients (mean age 50); 71% male	—	Mean duration of treatment was 24 days (range 6-47d).<br>Of 31 patients who were assessable after treatment completion, all were clinically and bacteriologically cured (31/31).<br>Twenty of 31 patients (65%) were reviewed ≥2 months after completion of treatment (median 6.5m; range 2-21m), and all remained free of relapse for the duration of follow-up.
128	White et al (Australia)	Case-series (retrospective)	OP; SN; GN	55 patients (mean age 50); 65% male	—	Median duration of IV therapy, inc. time spent as an inpatient, was 42 days (range 4-79d).<br>Median duration of treatment was 22 days (range 3-56d).<br>Forty six of 55 patients (84%) were considered to have had a successful outcome at completion of IV OPAT.
129	White et al (UK)	Case-series (retrospective)	SA	72 patients (mean age 42); 42% male	—	Median duration of treatment was 21 days (range 1-43d).<br>Definite improvement was seen in 20/72 patients (28%), modest/slight/transient improvement in 24 patients (33%), and no improvement was seen in 26 patients (36%).
133	Wynn et al (USA)	Observational (retrospective)	Physician-based program; Home health organisation; Hospital-based program (no other details)	1252 patients (mean age 52); 65% male	—	A total of 1202/1252 patients (96%) were treated successfully for their condition.
134	Yadlapalli et al (USA)	Case-series (retrospective)	Not specified	58 patients (mean age 60); 83% male	—	Mean duration of antibiotic therapy was 40.3 days (range 19-90d).<br>At the end of 12-months follow-up, 46/58 patients (79.3%) were cured (mean healing time 15.4 weeks). Three of the 12 patients (5.2%) who failed to heal had an amputation, and nine (15.5%) had persistence of ulceration after a year.

SUPPLEMENTARY TABLES

ID	Author (country)	Study design	OPAT Model(s)	OPAT group(s)†	Comparator(s)†	Findings
135	Yan et al (UK)	Case-series (retrospective)	OP; GN	140 patients (aged 17-89); 64% male	—	Mean duration of treatment for patients with cellulitis (n=128) was 4.4 days. Duration of treatment ranged from 0 to >15 days. Around 60% of patients received IV antibiotics for ≤3 days. The failure/complication rate among patients was low (8/140; 5.7%).

OP: outpatient; SA: self-administered; SN: specialist nurse; GN: General nurse.

† Age and gender details relate only to those patients who were clinically evaluable or provided questionnaire data.

For peer review only

TABLE S2 Safety associated with outpatient parenteral antibiotic treatment

ID	Author (country)	Study design	OPAT Model(s)	OPAT group(s)†	Comparator(s)†	Findings	Notes
**COMPARATOR STUDIES**							
20	Bedi et al (UK)	Cohort	SA	Home therapy (domiciliary IV); 52 patients (median age 61); 58% male<br>Home therapy (early supported discharge); 23 patients (median age 65); 35% male	Inpatient therapy; 36 patients (median age 71); 36% male	Thirty-day re-admission rates were similar across groups (inpatient 13.8%; ESD 12.5%; domiciliary IV 14.2%).<br>Antibiotic side effects developed in four inpatients (5%), two ESD patients (6.3%) and four domiciliary IV patients (4.7%).<br>There were no IV access-related complications in the inpatient group, two (6.3%) in the ESD group, and three (3.6%) in the domiciliary IV group.<br>No deaths were recorded in any group.	Reasons for admission were: exacerbation of bronchiectasis (all).<br>Access-related complications were: line blockage (3), line fell out (2), and line sepsis (1).
30	Corwin et al (New Zealand)	RCT	GN	Home therapy; 98 patients (mean age 55); 62% male	Inpatient therapy; 96 patients (mean age 48); 73% male	Eleven of 98 patients in the Home group (12%) were admitted to hospital.<br>Three of 96 hospital patients (3%) required re-admission within one month.	Reasons for admission were: unsatisfactory clinical improvement (4), surgical drainage (1), PICC line insertion (2), ischaemic toe (1), rash (1), nausea/vomiting on starting post-discharge oral antibiotics (1), and not coping at home (1).
32	Dall et al (USA)	Before and after	SN	Home therapy; 92 pneumonia patients (97% aged >50 years); 44% male / 64 cellulitis patients (mean age 51); 70% male	Inpatient therapy; 10728 pneumonia patients (83% aged >50 years); 51% male / cellulitis patients (no numbers provided)	Two of 92 patients with pneumonia were re-admitted (rate over 30-day period, 2%). The re-admission rate for comparator patients was 7.4% (69/933 patients).<br>Six cellulitis patients were hospitalised briefly (9.4%). One treatment failure resulted in re-admission (for MRSA).<br>There were no deaths among OPAT patients.	
39	Escalante et al (USA)	Clinical trial (unspecified)	Not specified	IV therapy; 43 patients (median age 39); 42% male	Oral therapy; 40 patients (median age 52); 50% male	Three of 40 patients receiving oral treatment had an adverse drug reaction.<br>Six patients receiving oral treatment (15%) were admitted to	Adverse drug reactions were: renal toxicity (3).<br>Reasons for admission were: renal toxicity (3), and persistent fever (3).

SUPPLEMENTARY TABLES

ID	Author (country)	Study design	OPAT Model(s)	OPAT group(s)†	Comparator(s)†	Findings	Notes
						hospital; there were no admissions in patients receiving IV treatment. There were no infection-related complications or deaths.	
40	Esmond et al (UK)	Controlled trial	SA	Home therapy; 15 patients (mean age 27); 40% male	Inpatient therapy; 15 patients (mean age 23); 60% male	There were no drug reactions, IV line problems or sepsis reported in either group.	
44	Fernandez-Aviles et al (Spain)	Case-control	SN	Home therapy; 50 patients (mean age 47); 62% male	Inpatient therapy; 50 patients (median age 50); 54% male	There was no significant difference in mucositis (Home 24% vs. 34%), diarrhoea (35% vs. 39%) or bacteraemia between the two groups. In 40% of Home patients' day unit consultations, physician intervention was required. Four Home patients (8%) were re-admitted. The median number of admitted days was 8 days for Home patients and 25 days for Hospital patients (p<.00001). No patients in either group died.	Reasons for intervention were: fever (75%), catheter-related complication (10%), and mucositis, bleeding, diarrhoea, or chemotherapy related exanthema (15%). Reasons for re-admission were: fever with hemodynamic instability (3), and pneumonia (1).
58	Keller et al (USA)	Before and after	Home agency; skilled nursing facility; other (not specified)	Pre-infectious disease transition service; 215 patients (mean age 56); 60% male Standard OPAT (pre);70 patients (mean age 58); 69% male	Post-IDTS; 147 patients (mean age 57); 54% male Standard OPAT (post); 56 patients (mean age 55); 68% male	Overall, 199 patients (40.8%) experienced either a re-admission and/or ED visit within 60 days of discharge (IDTS arm: pre n=82, 38.1%; post n=41, 27.9%; standard OPAT arm: pre n=35, 50.0%; post n=16, 28.6%). There was no relationship between the presence of the IDTS and re-admission and/or ED visit at 60 days (adjusted OR 0.48; 95% C, 0.13-1.79). Similar results were observed for 7-day and 30-day re-admissions and/or ED visits. The IDTS was associated with decreased antimicrobial prescribing errors (OR 0.062; 95% CI, 0.015–0.262), increased receipt of laboratory test results (OR 27.85; 95% CI, 12.93–59.99), and increased follow-up (OR 2.44; 95%	

SUPPLEMENTARY TABLES

ID	Author (country)	Study design	OPAT Model(s)	OPAT group(s)†	Comparator(s)†	Findings	Notes
						CI, 1.50–3.97). During the 60-day period after discharge, drug-related adverse events, catheter complications, and infection relapse were common.	
61	Lacroix et al (France)	Observational (retrospective)	Not specified	OPAT; 18 patients (mean age 60); 61% male	Inpatient therapy; 21 patients (mean age 68); 67% male	During OPAT, three patients (16.7%) presented with severe adverse events requiring re-hospitalization; none were attributed to OPAT. Following OPAT, three patients (16.7%) presented with severe adverse events, including one death. This compares with seven re-admitted patients (33.3%) and one death (4.8%) in the hospital group.	Reasons for re-admission were: haemorrhagic stroke with subdural haematoma (1); fever due to beta-lactams requiring re-hospitalization for a switch to vancomycin (1); cardiac failure (1). Adverse events were: death from pneumonia with severe hypoxemia (1), relapse of IE (1), and progressive mitral regurgitation requiring mitral surgery after IE cure (1).
75	Martone et al (USA)	Observational (retrospective)	Not specified	OPAT; 539 patients (57% aged >50 years); 56% male	Inpatient therapy; 410 patients (63% aged >50 years); 45% male	A total of 216 adverse events were reported in 50/539 OPAT patients and 81/410 hospital patients (9.3% vs. 19.8%; p<0.0001). A total of 89 adverse events possibly related to therapy were experienced by 31 OPAT and 34 hospital patients (5.8% vs. 8.3%; p=0.12). Significantly more hospital patients had diarrhoea (2.4% vs. 0.2%; p=0.001).	
76	Matthews et al (UK)	Cohort (retrospective)	SA; GN; Community hospital staff	Self-administered OPAT; 513 episodes (mean patient age 46)	Professional-administered OPAT; 1621 episodes (mean patient age 61)	Drug associated complications were experienced in 189/1536 H-OPAT episodes (12%) and 59/473 S-OPAT episodes (12%). Complications related to vascular access affected eight (0.5%) H-OPAT patients and five (1%) S-OPAT patients (p=0.2). The overall complication rate was 23% (353/1536 episodes) for H-OPAT patients and 24% (112/473 episodes) for S-OPAT (p=0.7). Re-admission occurred for 193 H-OPAT and 50 S-OPAT patients (rates of 12.6% and 10.5%	Drug related complications included: GI side-effects (58), drug rash (52), drug fever (51), unclassified drug reaction (45), neutropenia (41), anaphylaxis (4), renal impairment (1), and C-diff diarrhoea (1). Access-related complications were: line infection (3), leaking line (3), mechanical phlebitis (2), and thrombosis (6).

SUPPLEMENTARY TABLES

ID	Author (country)	Study design	OPAT Model(s)	OPAT group(s)†	Comparator(s)†	Findings	Notes
						respectively; p=0.3). Two H-OPAT patients died (unrelated cardiac causes).	
78	Mazo et al (Spain)	Case-control (retrospective)	Hospital in the Home (HHU)	HHU therapy; 150 patients (mean age 67); 47% male	Inpatient therapy; 150 patients (mean age 63); 51% male	HHU decreased the number of complications or re-admissions to the surgical or emergency unit at 45 days (HHU rate 0.67% vs. hospital rate 3.5%).	
81	Montalto & Dunt (Australia)	Observational (retrospective)	SN (HHU)	HHU therapy; 55 cellulitis patients (mean age 52); 60% male / 14 pyelonephritis patients (mean age 30); 0% male	Inpatient therapy; 22 cellulitis patients (mean age 51); 73% male / 10 pyelonephritis patients (mean age 35); 30% male	Incidents were recorded for 3/69 HHU patients (4%) and 10/32 Hospital patients (31%). Four Hospital patients (three with cellulitis, one with pyelonephritis) were re-admitted within four weeks.	HHU incidents were: drainage of abscess (2), and vasovagal episode at administration of medication (1). Hospital group incidents were: unexpected non-urgent operation (2), drug reaction (1), unexpected returned to hospital within four weeks (4), anaphylaxis (1), re-admission for related condition (1), and unexpected gastroscopy (1).
98	Pond et al (UK)	Case-control (retrospective)	SA	Home therapy; 25 patients (mean age 21); 56% male	Inpatient therapy; 25 patients (mean age 22); 56% male	There were 2/25 (8%) adverse reactions in each group. All consisted of mild skin rashes that subsided with withdrawal of the relevant antibiotic.	
101	Rapoport et al (Multinational)	RCT	OP	Outpatient therapy; 40 patients (median age 45); 52% male	Inpatient therapy; 44 patients (median age 48); 32% male	Incidence of adverse events was similar for OPAT patients (6/40; 15%) and inpatients (8/44; 18%). Three events (one OPAT, two inpatients) were considered to be potentially drug related; two events were severe (one in each group). There was one death in the OPAT group.	
102	Rehm et al (USA)	RCT	SA; SN; (also skilled nursing facility; assisted-living; nursing home; rehab centre)	Home therapy; 103 patients (median age 50); 58% male	Inpatient therapy; 97 patients (median age 54); 61% male	Fewer patients in the OPAT group (48/103; 46.6%) experienced a serious adverse event compared with hospital patients (52/97; 53.6%). A total of 53 hospital patients (54.6%) discontinued therapy compared with 10 OPAT patients (9.7%). This was most commonly	Reasons for OPAT discontinuation were: skin problems (4), renal failure (2), diabetic gastroparesis (1), and blood creatine phosphokinase increased (1). Reasons for inpatient discontinuation were: cardiovascular problem (4), skin

SUPPLEMENTARY TABLES

ID	Author (country)	Study design	OPAT Model(s)	OPAT group(s)†	Comparator(s)†	Findings	Notes
						related to adverse events (80% of OPAT; 47% of hospital).<br>Eighteen of 103 OPAT patients (17.5%) required re-admission.<br>Fewer deaths occurred in the OPAT group (n=4; 3.9%) than in the hospital group (n=18; 18.6%) (p=0.001).	problem (4), infection (3), renal failure (3), blood creatine phosphokinase increased (2), fever (2), sepsis (2), anaphylactic reaction (1), hypoxia (1), red man syndrome (1), thrombocytopenia (1), and vomiting (1).<br>Reasons for OPAT re-admission were: other medical condition (11), related to initial infection (4), and problems related to treatment provision in post-acute care setting (3).
103	Richards et al (New Zealand)	RCT	GN	Home therapy; 24 patients (mean age 50); 54% male	Inpatient therapy; 25 patients (mean age 50); 52% male	Two patients in each group reported nausea and candidiasis.<br>There were five extra-pulmonary infections in Home patients (21%) and four in Hospital patients (16%), and two pulmonary complications in the Home group (8.3%) and one (4%) in the Hospital group.<br>Two of 24 patients were transferred from the Home group to hospital (one with pulmonary infection). One Hospital patient was re-admitted (clinical deterioration).<br>There were no deaths in either group.	Reasons for home to hospital transfer were: development of empyema in legionella infection (1); development of bullous myringitis (1).<br>Extra-pulmonary infection in the Home group included three IV site infections.
109	Seaton et al (UK)	Before and after	SN	Standard OPAT; 230 patients (median age 49); 52% male	Nurse-led OPAT; 112 patients (median age 50); 61% male	Medical review was required for 21/112 patients (19%) post-introduction of a nurse-led protocol.<br>Re-admission (6% vs. 7%), drug reaction (4% vs. 7%) and change in therapy rates (5% vs. 4%) were similar for pre- and post-protocol patients.	Reasons for review were: other medical problem (5), rash (8), incision of abscess required (3), and therapy change or admission for lack of improvement (5).
111	Sebban et al (France)	RCT	OP	IV therapy; 47 patients (mean age 52); 40% male	Oral therapy; 49 patients (mean age 52); 31% male	There were five adverse events in the IV group (5/46) and three in the Oral group (3/48). There were no statistical differences between the two groups for toxicity.<br>Nine IV patients (19.6%) and five Oral patients (10.4%) were re-admitted. There were no	Reasons for re-admission were: persistent temperature (9), chose to be re-admitted (2), severe dental infection (1), positive initial blood cultures (1), and allergic reaction to ceftriaxone (1).

SUPPLEMENTARY TABLES

ID	Author (country)	Study design	OPAT Model(s)	OPAT group(s)†	Comparator(s)†	Findings	Notes
						significant differences between groups in all-cause mortality, clinical deterioration, relapse of infection, new infection, modification of therapy, or re-admission.	
116	Stein et al (USA)	RCT	OP	IV therapy; 10 patients (mean age 54); 60% male	Oral therapy; 10 patients (mean age 53); 40% male	There were no side-effects in the OPAT group. Two of 10 patients in the Oral group (20%) experienced adverse events (one discontinued treatment).<br>Four of 10 OPAT patients (40%) were hospitalised due to lack of improvement.	Adverse events were: skin and tongue pruritus (1), and diarrhoea (1)
131	Wolter et al (Australia)	RCT	SA	Home care; 13 admissions (median age 22); 28% male	Inpatient therapy; 18 admissions (median age 22); 39% male	There were no adverse drug reactions, short-term re-admissions, events or deaths attributable to the drugs used.<br>One patient had a pneumothorax associated with central line insertion.<br>There were no differences between groups in IV complication rates (p=0.57) or numbers of line changes required (p=0.5).	There was no significant difference in time to next admission between the two groups (p=0.68).
132	Wolter et al (Australia)	RCT	Not specified	Home care; 44 admissions (median patient age 43); 45% male	Inpatient therapy; 38 admissions (median patient age 49); 34% male	There were 23 adverse events reported in the Home group (52%) and 31 in the Hospital group (82%). Five were considered serious.<br>Five Hospital and three Home group events were considered to be directly/possibly related to place of therapy.<br>There were seven re-admissions in the Home group and four in the Hospital group 30 days after discharge. Three patients receiving home therapy had unplanned re-admission during treatment.	Serious events were: CVA (1), pneumothorax (1), line elated sepsis (1), and PICC vein thrombosis (2).<br>Location related events were: Hospital patient with vancomycin resistant enterococci (4) or MRSA (1), Home patient with peripheral IV infection (1) or presented to another hospital for re-admission (1) or PICC line fracture (1).

SUPPLEMENTARY TABLES

ID	Author (country)	Study design	OPAT Model(s)	OPAT group(s)†	Comparator(s)†	Findings	Notes
136	Yang et al (Canada)	Case-control	Not specified	OPAT; 21 patients (mean age 59); 57% male	Previous standard care; 21 patients (mean age 59); 57% male	Incidence of 30-day re-admission was lower in the OPAT group than in the previous care group (14.3% vs. 28.6%, p=0.51), and mean length of hospital stay was shorter by 3.2 days (10.7 vs. 13.9, p=0.36).<br>The total incidence of adverse drug reactions (n=5, 23.8%) and VAD-related complications (n=2, 9.5%) were the same for each group. Three patients in the previous care group (60%) required a change or discontinuation of therapy compared with one (20%) in the OPAT group (p=0.62).<br>There were no re-admissions for adverse events in the OPAT group, and two in the previous care group (9.5%).	Adverse drug events included: allergy (3), haematological (1), renal/liver toxicity (2), GI-related (1), neurological (1), and abnormal drug level (2).
137	Yong et al (Singapore)	Case-control (retrospective)	OP; SA	OPAT; 69 patients (mean age 53); 53% male	Inpatient therapy; 93 patients (mean age 56); 51% male	Complications occurred in 18/72 OPAT episodes (25%), eight of which required re-admission.<br>At the end of 4-months follow-up, there was no difference in the number of OPAT (13/72; 18.1%) and hospital patients (18/93; 19.4%) re-admitted due to relapse (p=0.991).<br>There was one patient death in each group.	Complications were: PICC line related (8), adverse drug reaction (2), admission for underlying medical condition (7), and admission for adverse drug reaction (1).
**OPAT ONLY STUDIES**							
11	Al Ansari et al (Bahrain)	Case-series (retrospective)	OP	101 patients; 57% male	—	Two patients (2%) were re-admitted to hospital.	
12	Allison et al (USA)	Cohort (retrospective)	SN	782 patients (mean age 58); 57% male	—	Twenty-six percent (207/782) of patients were re-admitted within 30 days.<br>Nearly half of 16 patients prescribed aminoglycosides at discharge, (6) were re-admitted for medication side effects<br>Regression analysis showed that	Reasons for re-admission were non-ID related (63), worsening infection (62), new infection (48), adverse drug reaction (30), line complication (20), and diarrhoea (2).<br>Medication side-effects were: acute renal insufficiency (4), rash

SUPPLEMENTARY TABLES

ID	Author (country)	Study design	OPAT Model(s)	OPAT group(s)†	Comparator(s)†	Findings	Notes
						risk of re-admission was related to age (OR, 1.09 per decade; 95% CI 0.99-1.21; p=0.10), aminoglycoside use (OR, 2.33; 95% CI 1.17-4.57; p=0.01), drug resistant organisms (OR, 1.57; 95% CI 1.03-2.36; p=0.03), and number of prior hospital discharges without IV antibiotics in the past 12 months (OR, 1.20 per prior admission; 95% CI 1.09-1.32; p<0.001).	(1), rash and neutropenia (1).
13	Amodeo et al (New Zealand)	Observational (retrospective)	SA; GN	100 treatment courses (mean patient age 65); 75% male	—	Adverse events occurred in 27/100 episodes; 17 minor events managed in clinic, and 10 re-admissions.<br>There were two deaths in the 12-month follow-up period.<br>Five patients were re-admitted with further episodes of IE; mean number of days between completion of OPAT and re-admission was 220 (range 109-344d).	Minor adverse events were: ototoxicity (1), drug induced hepatitis (1), itch (1), PICC line occlusion (6), line migration (2), phlebitis (2), cellulitis (1), and high residual infusate unrelated to PICC line patency (3).<br>Reasons for re-admission were fever and rash (3), drug fever (2), diarrhoea and vomiting (1), hepatitis (1), worsening congestive heart failure (1), flank pain secondary to renal sub-capsular bleed (1), and recurrence of angina (1).
14	Anand et al (USA)	Case-series	Home IV therapy (unspecified)	52 patients	—	Seven of 52 patients (13%) had minor complications requiring a change of antibiotic or therapy duration.	Complications included: rash (1), increased LFTs (1), transient neutropenia / septicaemia (1), and bleeding at PICC site (1).
15	Angel (USA)	RCT	SA	62 patients (mean age 71); 53% male	—	Of 14 reported events, seven were judged unrelated to therapy.<br>There were no deaths during the study period.	Related adverse events were: phlebitis (2), rash (2), diarrhoea (1), nausea (1), and fever (1).
17	Barr et al (UK)	Observational (retrospective)	OP; SA	854 treatment courses; 55% male	—	Twenty of 854 patients had diagnosed line infections, 2.3% of all indwelling line episodes, and an incidence of 0.79 per 1,000 line use days (95% CI 0.68-0.91).<br>Incidence of other line events was 125/854 (14.6%), 4.9 per 1,000 indwelling line use days (95% CI 4.68-5.21).	There was a lower rate of line infection in Self compared to Clinic OPAT, but this was not statistically significance (OR 0.6846; 95% CI 0.2805-1.671; p=0.362).<br>There was a higher risk of other line events with Self compared to Clinic OPAT (OR 1.62; 95% CI 1.06-2.46; p=0.032). This was

ID	Author (country)	Study design	OPAT Model(s)	OPAT group(s)†	Comparator(s)†	Findings	Notes
							no longer significant in multivariate analysis (p=0.22).
18	Barr et al (UK)	Observational (retrospective)	OP; SA; SN; GN	2233 patients (median age 51); 58% male	—	Crude admission rate from OPAT was 11.7% (262/2233 patients). Unplanned re-admission was recorded for 204 patients (9.1%) (CR of 6.3 events per 1000 OPAT days).<br>A total of 219/2233 episodes (9.8%) were associated with adverse drug reaction (CR 6.7 events per 1000d).<br>Line infections occurred in 14/2233 patients (0.6%) (CR 0.4 events per 1000 days). Other line events occurred in 92 patients (4.1%) (CR 2.8 events per 1000d).<br>Eight patients died (0.4%).	Unplanned admission included: deterioration in infection (76), new medical event not infection related (63), adverse drug reaction (28), surgery (12), line complication (7), and logistic reasons (7).<br>Drug reactions were: rash (89), severe GI upset (38), chills or fever (28), leukopenia (22), thrombocytopenia or anaemia (21), nephrotoxicity (13), and hepatotoxicity (6).<br>Other line events included: line obstructed or leaking, 'chemical' or 'mechanical' phlebitis, patient default of follow-up with line in situ, and line fall out.
19	Barr et al (UK)	Observational (retrospective)	Not specified	780 patients (median age 52); 58% male	—	During or up to 90 days following OPAT, 34 patients (4.4%) were investigated for suspected symptomatic venous thromboembolism (VTE); two DVTs were diagnosed (incidence 2/780, 0.26% (95% CI: 0.03–0.92%)).<br>Up to 1 year following OPAT, there were a total of five VTEs (0.64%). The rate of VTE appeared constant in the year following OPAT.	
21	Berman & Johnson (USA)	Case-series (retrospective)	SA	221 patients (median age 41); 66% male	—	Side-effects were documented in 74/302 episodes (25%). In addition, 18% of patients (55 episodes) complained of constitutional symptoms.<br>Three patients (1.4%) were hospitalised for side-effects.	Side-effects included: renal dysfunction (21), drug rash (18), anaemia (13), diarrhoea (13), vestibular dysfunction (11), fever (9) thrombocytopenia (8), neutropenia (7), and hepatitis (3).<br>Constitutional symptoms were: fatigue (20), headache (12), nausea (19), anorexia (5), weakness (2), palpitations (1), sleepiness (1), and insomnia (1).

SUPPLEMENTARY TABLES

ID	Author (country)	Study design	OPAT Model(s)	OPAT group(s)†	Comparator(s)†	Findings	Notes
22	Bernard et al (France)	Observational (?)	SA; SN;	39 patients (mean age 44); 64% male	—	Four of 39 patients reported adverse events (10%). Four of 39 patients were re-admitted for medical reasons (10%). There were no haematological or renal complications.	Adverse events were: rash (2), and nausea and vomiting (2). Reasons for re-admission were: deep venous thrombophlebitis (3), and allergic reaction (1).
24	Cervera et al (Spain)	Observational	SA; SN	73 patients (mean age 60); 75% male	—	Twelve of 73 patients (16.4%) had complications requiring re-admission. Three patients had fatal complications.	Non-fatal events were: heart failure (2), catheter-related sepsis (1), variceal haemorrhage (1), abdominal pain (1), dizziness (1), lower-back pain (1), fever (1), and hypersensitivity reaction (1). Fatal complications were: health-care related pneumonia (1), cerebral haemorrhage (1), and pulmonary oedema (1).
25	Chambers et al (New Zealand)	Case-series	SA; SN	153 patients (median age 55); 58% male	—	Complications of treatment developed in 31/153 (20%) patients. All catheter related infections (n=3) resolved with removal of the devices. Fifteen patients (10%) were re-admitted within one month of discharge from hospital.	Complications were: phlebitis (9), line occlusion (12), line leakage or breakage (6), infection (3), thrombosis (1), and pump failure (4). Reasons for re-admission were: washout of infected prosthesis (2), slow resolution of cellulitis (3), jugular vein thrombosis (1) line sepsis (1), uncontrolled pain (3), discharged to inappropriate level of care (1), and unrelated condition (4).
27	Chan et al (Singapore)	Observational	Not specified	109 patients (mean age 57); 66% male	—	Nine patients were re-admitted from OPAT. There were no deaths or relapses at 30 days post cessation of antibiotics.	Reasons for re-admission were: surgical drainage of abscess after initial drainage failure (3), worsening of underlying non-infectious comorbidity (5), and infectious condition unrelated to KLA (1).
28	Chapman et al (UK)	Observational (retrospective)	SA; Infusion centre	334 treatment courses (mean patient age 46); 59% male	—	Twenty-one of 334 treatment episodes (6.3%) ended in re-admission. In 11 episodes OPAT was terminated because of a change of plan. Two patients developed C-diff	Reasons for re-admission were: unrelated to OPAT (6), inappropriate referral (6), non-resolving soft tissue sepsis (3), deep sepsis with inadequate clinical response (1), symptom control (1), alternative IV therapy

SUPPLEMENTARY TABLES

ID	Author (country)	Study design	OPAT Model(s)	OPAT group(s)†	Comparator(s)†	Findings	Notes
						diarrhoea during IV antibiotic therapy; a further patient had diarrhoea on starting OPAT, and was found to have C. difficile toxin.	following antibiotic reaction (1), line infection (1), fractured humerus after fall travelling to OPAT unit (1), cut off end of PICC self-administering antibiotics (1).
29	Cheong et al (Australia)	Case-series	GN	714 treatment courses (mean patient age 53); 42% male	—	Adverse drug reactions occurred in 38/714 courses of treatment (5.3%).<br>Drug reactions in five patients were ranked as serious or life-threatening (all were re-admitted).	Adverse events included: skin reaction (18), abdominal symptoms (8), vascular symptoms (5), renal function changes (4), hypotension (2), shortness of breath (1), and severe cutaneous symptoms (1).
31	Cox et al (USA)	Observational (retrospective)	SA	205 patients (mean age 59); 99% male	—	Adverse events occurred in 99/231 (43%) home IV courses.<br>Venous access complications were frequent (n=57; 25%) but rarely serious. Catheter or infusion problems resulted in admission in five treatment courses.<br>All other events related to adverse drug reactions (n=42; 18%).	Venous access complications were: catheter pulled out (22), occluded catheter (18), irritation from dressing (6), site infection (4), leakage (3), bacteraemia (3), and phlebitis (1).<br>Adverse drug reactions were: nephrotoxicity (10); eosinophilia (9), rash (8), leukopenia (7), anaemia (5), and thrombocytopenia (3).
33	Dalovisio et al (USA)	Observational (retrospective)	SA; SN	62 patients (mean age 69)	—	Five of 66 treatment courses (7.6%) resulted in re-admission within 30 days; none were directly related to the infectious diagnosis.<br>Twelve of 62 patients (19.4%) experienced line complications.<br>No patients died while receiving IV treatment.	Reasons for re-admission included: pneumonia, chest pain, surgical wound infection, hepatic failure, and tumour fever.<br>Line complications included: line breakage, leakage, infection, and infiltration.
34	Dargan et al (Canada)	Case-series (retrospective)	OP; Home therapy (unspecified)	66 patients (mean age 57); 50% male	—	Nineteen of 66 patients (29%) experienced a total of 25 complications. Most (n=15) were attributed to IV access, and the remainder to adverse drug reactions.<br>Five patients (all treatment failures) were re-admitted to hospital for reasons attributable to the infection.	Access related events were: interstitial of IV line (6), occluded line (3), phlebitis (3), bleeding (1), leakage of PICC line (1), and thrombosis (1).<br>Drug related events were: nausea (3), acute renal failure (1), allergic reaction (1), hypokalaemia (1), nephrotoxicity (1), neutropenia (1), vomiting (1), and yeast infection (1).

SUPPLEMENTARY TABLES

ID	Author (country)	Study design	OPAT Model(s)	OPAT group(s)†	Comparator(s)†	Findings	Notes
35	Dobson et al (Australia)	Case-series	SA; SN; GN	770 patients (mean age 48); 66% male	—	Allergic reactions were experienced by 28/770 patients (3.6%). No patient had anaphylaxis.<br>Mean time from treatment start to reaction onset was 19.6 days (range 1-39d).	Allergic reactions included: rash (13), rash with pruritus (4), fever (3), and perioral angioedema (3).<br>No reactions were experienced by patients who received drugs via bolus or intermittent administration alone (although this did not reach significance (OR 0; 95% CI 0-1.7)).
36	Donald et al (Australia)	Case-series (retrospective)	SN	124 patients (aged 16-97); 60% male	—	One patient developed self-limiting diarrhoea. There were no other complications attributable to therapy.<br>Nineteen of 124 patients (15%) were re-admitted.	Reasons for re-admission were: failure of cellulitis to resolve (13), abscess requiring surgery (4), analgesia needed (1), and admission for central venous access (1).
37	Duncan et al (UK)	Observational (retrospective)	Not specified	1377 treatment courses	—	A total of 51 adverse reactions were observed in 1377 episodes (3.7%), a prevalence rate of 37.0 per 1,000 new patient episodes.	Adverse reactions were: rash (20), hepatic enzyme abnormality (10), leukopenia, thrombocytopenia or anaemia (6), GI symptoms (5); anaphylaxis (4), chills or fever (3), and acute kidney injury (3).
38	Duncan et al (UK)	Observational (retrospective)	Not specified	55 completed OPAT patients (median age 59); 80% male / 25 failed OPAT patients (median age 67); 60% male	—	Twenty-one episodes resulted in re-admission (26.3%), seven in an adverse drug event (8.8%), three in line complications (3.8%), and two in drug resistance (2.5%).	Reasons for re-admission included: suspected endocarditis decompensation (7), and non-endocarditis related (4),<br>Reasons for OPAT failure included: hyperkalaemia (1), vomiting (1), acute renal dysfunction (1), presyncope during administration (1) and resistance to oral component of the OPAT regimen (1).
42	Esposito et al (Italy)	Observational (retrospective)	OP; SA; Visit by doctor or nurse (also care facility)	239 patients (aged 11-80+); 62% male	—	Adverse drug reactions, usually mild, were detected 27/239 patients (11%), and were more frequently seen in patients who received combination therapy (74.1% vs. 25.9%).	Adverse reactions were: rash (12), fever (3), hepatic impairment (3), nausea/vomiting (2) renal impairment (2), anaphylactic reaction (1), diarrhoea (1), itching (1), leukopenia (1), and urticaria (1).
43	Esposito et al (Italy)	Observational	OP; SA; Visit by doctor or nurse (also care facility)	176 patients (aged >65 years); 47% male	—	Adverse drug reactions, usually mild, were detected in 22/176 patients (13%).	Adverse reactions were: rash (7), fever (3), itching (3), renal impairment (2), hepatic impairment (1), diarrhoea (1), anaphylactic reaction (1),

SUPPLEMENTARY TABLES

ID	Author (country)	Study design	OPAT Model(s)	OPAT group(s)†	Comparator(s)†	Findings	Notes
							eosinophilia (1), dizziness (1), urticaria (1), and dyspnoea and palpitations (1).
45	Goodwin et al (Canada)	Case-series	SN; GN	2405 patients (mean age 46); 55% male	—	Thirty-three of 1642 patients (2%) discontinued therapy before prescribed completion (treatment related in 25 cases).<br>Fifty-two of 1642 patients (3.2%) were admitted to hospital.<br>Fifty-four of 1642 patients (3.3%) had unplanned interruptions to therapy; only two were unrelated to treatment (comorbidity).	Reasons for discontinuation were: adverse effect of therapy (9), inadequate venous access (5), unscheduled admission due to worsening infection (4), early resolution of infection (5), change of therapy (4), change to oral therapy (9), comorbidity (5), and patient decision (3).<br>Reasons for admission were: comorbidity (36), treatment failure (12), and inability to manage self-therapy (4).<br>Reasons for interruption were: venous access complication (15), temporary loss of venous access (26), device problem (n=18).
46	Gourdeau et al (Canada)	Case-series (retrospective)	OP; SA	124 patients (mean age 41); 56% male	—	Two of 124 patients (1.6%) experienced adverse reactions requiring treatment modification.<br>There were no cases of catheter-related infection.<br>One patient was re-admitted (due to lack of improvement).	Reasons for treatment modification were: rash (1), and recurrent phlebitis (1).
47	Graninger et al (Austria)	Case-series	OP	54 patients	—	Adverse events were seen in nine of 54 patients (17%).	Adverse events were: thrombocytopenia (3), rash (3), nausea (1), C-diff colitis (1), fever (1), transient hearing impairment (1), and leukopenia (1).
48	Grayson et al (Australia)	Case-series	GN	20 patients (mean age 58)	—	No major infective complications related to IV antibiotic administration were noted.<br>Two complications were seen (2/20; 10%).	Complications were: localised soft-tissue infection at catheter exit site (1), and progressive renal dysfunction (1).
49	Gross et al (USA)	Case-series (retrospective)	Home IV therapy (unspecified)	13 patients (mean age 38); 100% male	—	Eleven patients (85%) experienced 22 adverse events (one patient had four, two had three, four had two and four had one).<br>There were no deaths.	Adverse events were hypoglycaemic (8), nephrotoxicity (6), hypotension (5), hyperkalaemia (1), hypercalcaemia (1), and elevated

SUPPLEMENTARY TABLES

ID	Author (country)	Study design	OPAT Model(s)	OPAT group(s)†	Comparator(s)†	Findings	Notes
							pancreatic enzymes.
50	Heintz et al (USA)	Observational	OP; SA; SN; Infusion centre (also long-term care facility; interim-care facility; prison)	494 treatment courses	—	Infection and/or OPAT-related complications occurred in 35/494 cases (7.1%).<br>Intervention by the ID pharmacist post discharge occurred in 26/494 cases (5.3%).	Complications included: re-admission and/or emergency department visit within 30 days of discharge (28), 30-day infection-related mortality (2), and drug-induced toxicity (11).<br>Drug-induced adverse effects were: rash/hives (5), nephrotoxicity (3), bone-marrow suppression (2), and intractable nausea and vomiting (1).
51	Hindes et al (USA)	Case-series	SA	48 patients (mean age 65)	—	Complications were seen in four of 48 patients (8%).<br>Three patients required re-admission (6%),	Complications were: serum sickness reaction (3), and subclavian vein thrombosis (1).<br>Reasons for re-admission were: serum sickness (1), new site infection (1), and seizure unrelated to therapy (1).
52	Hitchcock et al (UK)	Case-series	OP; SA; GN; Private home healthcare company	273 patients (mean age 60); 54% male	—	Twenty three of 303 courses (8%) were followed by re-admission during therapy or within 28 days of completion.<br>Thirteen admissions were related to the original diagnosis or to OPAT drug treatment; 10 were unrelated.	Reasons for OPAT re-admission were: surgery after treatment failure (3), line problem (2), adverse drug effect (2), treatment failure at end of original course (3), super infection (1), and worsening clinical condition (2).
53	Ho et al (Singapore)	Case-series	OP	29 patients (median age 41); 90% male	—	Six of 29 patients (21%) required re-admission, five during OPAT and one during the 30 day follow-up period.<br>There were no deaths or significant misadventures.	Hospital re-admissions were: PICC infections (2), complications related to endocarditis (2), bacteraemia due to a different organism (2).<br>Incidence of re-admission and PICC infection was similar to that found in non-IVDU endocarditis patients treated in OPAT.
54	Htin et al (Australia)	Case-series (retrospective)	SN	68 patients (median age 68); 87% male	—	Three patients (4%) developed complications and required re-admission.<br>There were two deaths, both	Reasons for re-admission were: line-related bloodstream infection (1), fever due to flare of gouty arthritis (1), worsening anaemia in IHD, and Crohn's disease (1).

SUPPLEMENTARY TABLES

ID	Author (country)	Study design	OPAT Model(s)	OPAT group(s)†	Comparator(s)†	Findings	Notes
						unrelated to OPAT. Of the remaining 66 patients, 65 were still alive and 1 patient was lost to follow-up (1-year survival 96% (65/68)).	
55	Huminer et al (Israel)	Case-series	SA; SN (also nursing home; kibbutz)	37 patients (mean age 64); 57% male	—	Eight of 37 patients (22%) experienced adverse drug reactions.<br>Ten of 37 patients had local complications (27%).<br>Six patients were re-admitted before completing OPAT.	Adverse drug reactions were: fever (5), rash (2), and syncope (1).<br>Local complications were: occluded line (10), thrombophlebitis (6), and intravascular infection (1)<br>Reasons for admission were: fever (5), and PSV tachycardia (1).
56	Johansson et al (Sweden)	Case-series	SA	11 patients (mean age 51); 73% male	—	One patient telephoned the hospital on one occasion for help with CVAD handling.<br>One patient developed a CVAD exit-site infection.<br>All other patients recovered without re-admission and without antibiotic changed.	
57	Kayley et al (UK)	Case-series	SA; GN; Doctor administered	67 patients	—	Complications of home therapy were minimal (17/67; 25%).<br>Line infections (n=3) were all seen in HIV positive patients who were treated for >6 months continuously.	Complications were: line infection (3), broken line clamp (5), blocked line (2), dislodged line (3), drug allergy (2), and C-diff diarrhoea (2).
59	Kieran et al (Ireland)	Case-series (retrospective)	SA; Nurses attached to commercial company	56 patients (median age 50); 57% male	—	Adverse events occurred in 12/60 episodes (20%).<br>Five of 56 patients (7%) had a line-related complication (rate of 3.9 per 1,000 outpatient central line days).<br>Drug-related adverse events occurred in 4/60 episodes (6.7%).<br>In seven of 60 episodes (12%), patients required re-admission related to an adverse event; in five cases (8.3%) this was therapy related.	Line complications were: line infection (2), line blockage (2), and dislodged line (1).<br>Drug-related events were: rash (2), acute hepatitis (1), and neutropenia (1).<br>Reasons for re-admission were: acute hepatitis (1), neutropenia (1), failure to manage OPAT (1), anxiety (1), and line infection (1).

SUPPLEMENTARY TABLES

ID	Author (country)	Study design	OPAT Model(s)	OPAT group(s)†	Comparator(s)†	Findings	Notes
62	Lai et al (USA)	Case-series (retrospective)	SA; SN	333 patients (mean age 62); 98% male	—	Complications occurred in 96/393 OPAT courses (24.4%). The most common was re-admission (n=49, 12.5%).<br>Adverse drug events were present in 40 of the 393 courses (10.2%). Shortening of OPAT (including switches to oral antimicrobial therapy) occurred in 29 of the 40 cases (72.5%; 7.4% of all).<br>The overall line-related complication rate was 6.4% (25/393) over the period (PICC line, n=22; peripheral IV line, n=3).<br>Rates of PICC complications decreased across the period (8.4% to 4.5%), although this was not statistically significant.	Reasons for re-admission included: failure to improve on OPAT (15), reason unrelated to OPAT (13), PICC-related (9), and adverse drug event (8).<br>Adverse drug events were: acute kidney injury (11), pruritus/rash (10), leukopenia (7), GI problems, including diarrhoea (6), and C-diff associated diarrhoea (2).<br>Access-related complications included: occlusion/accidental displacement (9), erythema or tenderness (6), and bloodstream infection (6).
63	Lane et al (USA)	Cross-sectional	OP; SA	555 adult infectious diseases physicians	—	OPAT-associated complications were perceived as being rare (≤5% of patients).<br>The most commonly reported in greater numbers than this were line occlusion/ (80% of respondents), rash (39%), and nephrotoxicity (39%).<br>Patients commonly required line exchange or removal or change in antibiotic therapy because of complications; hospitalization was less common.	
64	Larioza et al (USA)	Case-series (retrospective)	Home infusion company	43 patients (56% aged >50 years); 67% male	—	Ten of 43 patients (23%) were re-admitted to hospital during antibiotic treatment.	Reasons for re-admission were: antibiotic-associated diarrhoea (1), PICC-related issues (6), interstitial nephritis (1), and drug rash (2).
65	Larioza et al (USA)	Case-series (retrospective)	Home infusion company	33 patients (76% aged ≥50 years); 61% male	—	Four of 33 patients (12.1%) developed complications.<br>There were no deaths.	Complications were: line infection (2), deep vein thrombophlebitis (1), and C-diff diarrhoea (1).

SUPPLEMENTARY TABLES

ID	Author (country)	Study design	OPAT Model(s)	OPAT group(s)†	Comparator(s)†	Findings	Notes
66	Laupland et al (Canada)	Observational	OP; SA; SN	2405 patients (mean age 46); 55% male	—	Overall, 139 of 3145 patients (4%) were admitted to hospital within seven days of starting home therapy. Another 82 patients (3%) had interruptions to therapy.	Reasons for interruption were: venous access related (45), infusion delivery system related (30), comorbidity (3), and unspecified (4).
69	Lillie et al (UK)	Case-series	SN	98 patients (mean age 55); 61% male	—	Of 64 patients attending 4-week follow-up, two (3.1%) had a relapse of infection; one patient required admission for treatment.	
70	Lin et al (USA)	Case-series (retrospective)	Not specified	177 patients	—	Antibiotic complications occurred at a rate of 16% (28/177). Most were minor in nature. PICC line related complications occurred in four patients (2%). There were no permanent complications or deaths.	Antibiotic complications were: rash (11), diarrhoea (6), transient neutropenia (3), itchiness (2), fatigue (2), flushing (1), fever (1), black tongue (1), and elevated LFTs (1). PICC related complications were: line thrombosis (3), and septicaemia (1).
71	Lopardo (Argentina)	Case-series (retrospective)	OP; SA; SN	48 patients (median age 55); 62% male	—	Complications occurred in 7/48 patients (15%; only one occurred while the patient was still receiving antibiotic treatment (heart failure).	Complications were: heart failure (3), and clinically significant emboli (4).
72	Mackintosh et al (UK)	Case-series (retrospective)	OP; SA; SN	198 patients (aged <40-89); 64% male	—	Twenty of 198 patients (10.1%) suffered an adverse reaction to initial IV therapy.	Adverse reactions were: rash (9), leukopenia or thrombocytopenia (4), vomiting (3), fever (2), tinnitus (1) and acute kidney injury (1).
77	Mauceri (USA)	RCT	SA	27 patients	—	Ten treatment-related adverse effects were reported in 27 patients, (two were considered severe). Three patients were withdrawn from the study due to adverse events.	Adverse events were: rash (3), gastrointestinal symptoms (3), growth resistant organisms (3), and fever (1)
79	McMahon et al (Australia)	Observational	SN	40 patients (mean age 59 (M), 49 (F)); 75% male	—	Nine of 40 patients (23%) experienced a total of 11 adverse events. All were considered to be related to the antibiotic or to complications of venous access. Three patients required hospital re-admission.	Adverse events were: rash (2), neutropenia (2), catheter-related (2), pseudomonas bacteraemia (1), fever (1), diarrhoea (1), nephrotoxicity (1), and chest pain during infusion (1). Reasons for treatment failure re-admission were: dyspnoea and

SUPPLEMENTARY TABLES

ID	Author (country)	Study design	OPAT Model(s)	OPAT group(s)†	Comparator(s)†	Findings	Notes
						Three patients who were treatment failures were re-admitted.	pericardial effusion (2), and cellulitis around a cardiac surgery site (1).
80	Mohammadi et al (USA)	Case-series (retrospective)	SA; SN	190 patients (mean age 63); 98% male	—	Adverse events occurred in 12/90 patients (6.3%) during the review period. No emergence of a drug-resistant organism or C. difficile was observed<br>There were five peripherally inserted central catheter line adverse events, including three admissions (1.6%).<br>Eight deaths during OPAT were related to malignancies.	Adverse drug events included: neutropenia, diarrhoea, drug allergy rash, and nephrotoxicity.<br>Access-related events included line infection (4), re-admission (3), and emergency department visits for line-related events (2).
83	Montalto (Australia)	Case-series (retrospective)	SN (HHU)	133 patients (mean age 46); 52% male	—	Twenty-eight telephone calls were received from 22/133 patients (17%; range 1-3 calls). Thirteen were initiated by the patient or carer either anxious or asking about the condition or its management. Most required reassurance and follow-up.<br>Thirteen calls resulted in unscheduled staff callouts. One patient was re-admitted.<br>Five of 133 patients (3.8%) were returned to hospital during their HHU admission.	Reasons for calls were: nausea or vomiting (6), pain (3), allergic reaction (1), diarrhoea (1), fever (1), problems with venous cannula (2), and perception of worsening condition (2).<br>Results of callouts were: IV maxolon given (5), management of anaphylaxis (1), vasovagal episode during drug administration (1), discussion with GP/unit director and action planned (5), and re-admission (1).<br>Reasons for re-admission were: carer withdrawal (1), exploration of possible abscess (1), exploration of possible palmar space collection (1), worsening respiratory state in COPD (1), and non-resolving pneumonia (1).
84	Montalto et al (Australia)	Case-series (retrospective)	SN (HHU)	3423 treatment courses (patient age 0-80+); 55% male	—	Unexpected telephone calls were made by 607/3423 patients (18%).<br>There were 177 unexpected staff callouts (5%). Patients receiving antibiotics were more likely to have a staff call out than patients	Reasons for callout included: pump malfunction (46), venous access problems (30), pain (19), vomiting (17), fever (14), and anxiety (13). Patients who called were more likely to return to hospital than those who did not

SUPPLEMENTARY TABLES

ID	Author (country)	Study design	OPAT Model(s)	OPAT group(s)†	Comparator(s)†	Findings	Notes
						receiving non-antibiotic therapy (p<0.001).<br>A total of 143 episodes of care (4%) involved an unplanned return to hospital. Patients who received antibiotic therapy were more likely to require an unplanned return to hospital (p=0.001).<br>There were five patient deaths; two during HHU stay and three after return to hospital.	(14.5% v. 2.2%, p<0.001).<br>Reasons for re-admission included: lack of improvement (40), cardiac problem (22), and fever (19). No patient had an anaphylactoid drug reaction; four patients were returned for other drug reactions.
85	Morales & von Behren (Puerto Rico)	Case-series	SA	22 patients (mean age 33-41); 82% male	—	Six of 22 patients (27%) reported nine adverse events (all mild or moderate).<br>Four patients were withdrawn due to adverse events.<br>There were no deaths during the study period.	Unrelated adverse events were: headache (1), urethral disorder (1), and diarrhoea (1).<br>Reasons for withdrawal were: urticaria (1), probable allergic reaction (1), flushing (1), and fever, rash, diarrhoea, and urethral disorder (1).
86	Muldoon et al (Ireland)	Cross-sectional	Not specified	55 consultant physicians (15% clinical microbiologists)	—	61% of those reporting on re-admission estimated the 30-day re-admission rate for OPAT patients as being less than 10%, while the remainder estimated it at between 10 and 25%.	
87	Muldoon et al (USA)	Cross-sectional	Not specified	316 adult and paediatric infectious diseases physicians	—	Most respondents had witnessed at least one complication in the last year (243/274, 89%).<br>Adverse drug reaction was the most commonly reported (69%), followed by thrombosis (58%), C-Diff related disease (53%), line-related bacteraemia / exit site infection (51%), re-admission (43%), phlebitis (40%) and death (2%).	
89	Nathwani et al (UK)	Case-series	OP; SA	101 patients	—	Six of 101 patients experienced an adverse drug reaction (6%).<br>Eight patients had an unscheduled re-admission (8%).<br>Twelve patients (12%) had PICC line complications.	

SUPPLEMENTARY TABLES

ID	Author (country)	Study design	OPAT Model(s)	OPAT group(s)†	Comparator(s)†	Findings	Notes
90	Nathwani (UK)	Case-series	OP; SA; SN	125 patients	—	There were no complications associated with vascular devices.<br>One patient was re-admitted for OPAT failure (due to various logistical reasons)	
92	Pajaron et al (Spain)	Case-series (retrospective)	SA	45 patients (mean age 63); 76% male	—	Eight patients returned to hospital with complications and six were admitted (12.5%).<br>An additional 11 patients had less serious complications (24.4%).<br>There were three recurrences in 2/45 patients during the year following the initial episode (one patient had two recurrences).<br>No patients died in the course of treatment at home. Five patients (13%) died during the first year following discharge from hospital (two related to endocarditis and three to pre-existing advanced cancer).	Reasons for re-admission were: cardiac insufficiency (2), acute renal insufficiency with hypokalaemia (1), sepsis (1), medication-related anaphylactic reaction to allopurinol and acute secondary renal insufficiency (1), and hepatic-renal syndrome secondary to hepatocarcinoma (1).<br>Adverse events were: congestive cardiac insufficiency (4), angina (1), acute episode of previous chronic renal insufficiency (1), drug-side effect (4), and catheter related infection (3).
93	Parker et al (UK)	Observational	OP; SA	29 patients; 38 GP practices	—	Eight of 29 patients (28%) reported a problem.	Adverse events were: due to speed of injection or discomfort caused by IV access (6), and hypersensitivity type reaction (2).
94	Partridge et al (UK)	Case-series (retrospective)	OP; SA	34 patients (mean age 55); 79% male	—	Adverse events occurred in 12/36 episodes (33%), inc. one patient death (unrelated to OPAT).<br>Two patients with line infection required re-admission.<br>Three patients were re-admitted for non-OPAT-associated medical problems.	Adverse events included: line infection (4), split Hickman line (1), PICC line dressing allergy (1), pain in PICC arm (1), ototoxicity (1), renal toxicity (1), diarrhoea (1), and mycotic aneurysm of the spleen (1).
95	Patanwala et al (USA)	Decision tree analysis	SN	41 patients (mean age 53); 44% male	—	Nine of 41 patients (22%; all treatment failures) were re-admitted.	
96	Perez-Lopez et al (Spain)	Observational	SN	145 patients (mean age 68); 48% male	—	There were 23 adverse events; most common was phlebitis (n=21; 15%).<br>A total of 11/145 patients (8%) required hospitalisation.	There were no significant differences in the number of adverse events between older (n=14; 15%) and younger (n=9, 16%) patients (p=0.65).

SUPPLEMENTARY TABLES

ID	Author (country)	Study design	OPAT Model(s)	OPAT group(s)†	Comparator(s)†	Findings	Notes
						No patients died.<br>A further 22 patients were re-admitted during the 3-month follow-up period (four died while in hospital).	
99	Poretz (USA)	RCT	SA	238 patients (mean age 45-52); 57% male†	—	Seventy-two of 238 patients (30%) reported adverse experiences considered related to therapy (seven classified as severe, 25 moderate, 65 mild).	Adverse events included: phlebitis (13), rash (12), diarrhoea (8), maculopapular rash (4), hypoxia (3), allergic reaction (3), fever (3), headache (3), and nausea (3).
100	Poretz (USA)	RCT	SA	130 patients (mean age 45); 59% male†	—	Thirty-one of 130 patients (24%), reported adverse events (27 events were related to the study drug and four to the IV line).	Adverse events included: rash (7), diarrhoea (4), phlebitis (4), allergic reaction (3), and maculopapular rash (3).
104	Rodriguez-Cerrillo et al (Spain)	Case-series	SN (HHU)	24 patients (mean age 73)	—	Three of 24 patients contacted the Unit for minor symptoms or doubts about treatment.<br>One patient required an unscheduled visit by HHU staff (vomiting).<br>No patient had an unexpected return to hospital.	
105	Rodriguez-Cerrillo et al (Spain)	Case-series	SN (HHU)	25 patients (mean age 59); 48% male	—	One of 25 patients (4%) made an unexpected telephone call for diarrhoea (after starting oral antibiotics).<br>There were no urgent visits by HHU staff, hospital re-admissions during HHU treatment, or admissions in the month following discharge.	
106	Seaton & MacConnachie (UK)	Case-series (retrospective)	SA; Other (unspecified)	19 patients (mean age 55); 53% male	—	Eighteen patients (95%) survived >6-months after hospital discharge.<br>There were two adverse events and one patient death. No further events were noted in the OPAT setting with therapy up to 128 days.<br>There were no unplanned re-admissions.	Adverse events were: above knee amputation for osteomyelitis/septic arthritis (1), and reversible myotoxicity (1).

SUPPLEMENTARY TABLES

ID	Author (country)	Study design	OPAT Model(s)	OPAT group(s)†	Comparator(s)†	Findings	Notes
110	Seaton et al (UK)	Observational (retrospective)	OP	963 patients (median age 48); 59% male	—	Significant adverse events were observed in 68/963 patients (7.1%). Admission or re-admission occurred in 58/963 cases (6%).	Adverse events included: drug-related rash requiring a change in therapy (25), GI side effects, particularly diarrhoea (16), and abnormal liver function (11). Three patients had severe allergy or anaphylaxis. Reasons for admission included: treatment failure with progression of infection or lack of response (19), and other medical complication (15).
112	Seetoh et al (Singapore)	Cohort	OP; SA; SN	2,229 patients (median age 56); 64% male	—	Re-admission was needed in 281 episodes (12.6%), and 74 patients (3.3%) ended treatment early. Re-admission rate due to clinical deterioration was 9.0%, and occurred a median of 11 days (IQR 5-23 days) after beginning treatment. SN OPAT episodes were more than twice as likely to deteriorate as OP episodes (20% vs. 7.9%; aHR 2.5, 95% CI, 1.7-3.8; p<0.001). There was no significant difference compared with self-administration (20% vs. 9.6% aHR 0.9, 95% CI, 0.6-1.3; p=0.572). Of those who did not complete treatment as planned (i.e. those re-admitted or ceasing early without re-admission): 60 (2.7%) had adverse drug reactions, 16 (0.7%) had PICC line complications, four (0.2%) absconded, and two (0.1%) died suddenly.	Reasons for re-admission included: elective procedures (42), and clinical deterioration (201).
113	Sims et al (UK)	Case-series (retrospective)	OP	14 patients (mean age 63); 71% male	—	Four patients (29%) required re-admission, two for medical conditions unrelated to OPAT (UTI and hematemesis), and two for deterioration in the clinical status of their infected arthroplasty. There were no line complications. There was one unrelated patient	

SUPPLEMENTARY TABLES

ID	Author (country)	Study design	OPAT Model(s)	OPAT group(s)†	Comparator(s)†	Findings	Notes
						death.	
114	Smego et al (Pakistan)	Case-series	SA	316 patients (mean age 45); 53% male	—	Medication-related adverse effects were few (14/316), generally mild, and resolved with drug dosage adjustment or discontinuation of use.<br>No unscheduled clinic or emergency room visits.	Side effects were: renal impairment (4), skin rash (3), diarrhoea (3), leukopenia (2), hepatitis (1), and drug fever (1).<br>Minor thrombophlebitis was the only catheter-related effect (3).
115	South (UK)	Case-series (retrospective)	OP; SA; Nurse (unspecified)	57 patients (mean age 41); 77% male	—	There were two adverse events among the 69 episodes treated (2.9%).	Adverse events were: shaking following IV bolus administration (1), and truncal rash (1).
117	Subedi et al (Australia)	Case-series	SA; SN	144 patients (median age 55); 74% male	—	There were 11 (7%) drug-related adverse events, one resulting in re-admission (6.9%). The rest were managed with symptomatic treatment, earlier cessation, or a change of antibiotic.<br>Line-related events occurred in five patients (3%) or 1.4/ 1000 catheter-days.<br>OPAT related re-admission occurred in nine patients (6%) within 28 days of cessation of IV antimicrobials.	Reasons for re-admission were: drug fever (1), re-insertion of PICC line (1), clinical deterioration requiring source control or surgical debridement (6), and change of antimicrobial therapy (1),<br>Adverse drug events included: rash (6), biliary lithiasis (1), drug fever (1), and acute kidney injury (2)<br>Access-related events were: infection (2), lymphatic leakage (2), and line fell out (1).
118	Talcott et al (USA)	Observational (pilot)	SA; SN	30 patients (median age 38); 43% male	—	Five patients were re-admitted for recurrent or prolonged fever (17%). Four patients were re-admitted for medical complications (13%).<br>There were no lasting complications or deaths.	None of the 27 medically eligible patients who did not enter the study had complications. The complication rate for all medically eligible patients was 4/57 (7%).
121	Theocharis et al (Greece)	Case-series (retrospective)	SN	91 patients (mean age 85); 31% male	—	Mortality was 27.5% (25/91; (those who were not cured).<br>Thirteen patients were admitted to hospital (14%); three died.	

SUPPLEMENTARY TABLES

ID	Author (country)	Study design	OPAT Model(s)	OPAT group(s)†	Comparator(s)†	Findings	Notes
123	Tice (USA)	Case-series	OP; SA; SN	538 patients (mean age 45); 52% male	—	Medication was changed due to an adverse effect in 16 cases.<br>Forty-two of 538 patients (7.8%) were hospitalised after starting OPAT. Only one patient (drug rash) was hospitalised due to the OPAT program or medications.<br>One death occurred during treatment (patient with advanced AIDS).<br>Three patients were removed from the program because of failure to comply.	Adverse events were: rash (11), leukopenia (1), neuromuscular disturbance (1), renal toxicity (1), laryngeal oedema (1), and intractable 'red man' syndrome (1).<br>Reasons for admission were: surgery unable to take place at outpatient centre (20), poor clinical response (5), inadequate home care (3), neurological problem (3), cardiac disease (2), bleeding (2), chest disease (2), vomiting (2), leukopenia and fever (1), reimbursement problem (1), and drug rash (1).
124	Tice (USA)	Observational (retrospective)	OP; SA; SN; Infusion centre.	500 patients	—	A minority of patients (12%) discontinued treatment prematurely, most commonly for adverse drug reaction (5.2%). Discontinuation related to clinical failure in around 1% of cases, and to death in 0.4%.	
125	Tice et al (USA)	Observational (retrospective)	OP; SA; Office based; Pharmacy based	971 patients	—	One hundred and nineteen of 1053 (11%) episodes were complicated by adverse events<br>Early discontinuation of therapy occurred in 6.4% of episodes (most commonly for rash and fever).<br>One hundred and forty-six venous access device-related adverse events were observed.	Adverse events were: rash, renal toxic reaction, fever, nausea and vomiting, urticaria, diarrhoea, anaphylactic reaction, leukopenia, and vestibular toxic reaction. Rash was common (3%); as renal toxicity (1.5%) and fever (1.3%).<br>Access events included: phlebitis (42), thrombosis (21), local infection (17), leakage (12), and bacteraemia (9).
126	Upton et al (New Zealand)	Case-series	SA	100 patients (mean age 51); 59% male	—	Thirty-five of 100 patients (35%) experienced complications during treatment.<br>Twenty-two patients had central line complications (incidence of 5.3 per 1000 central line days). Twelve occurred after discharge, and five resulted in re-admission.<br>Twenty two patients had adverse	Line complications were: line blockage (8), infection (5), DVT (4), phlebitis (3), failed line insertion (1), and line intolerance (1).<br>Adverse drug reactions were: rash (8), GI symptoms (7), chest discomfort (6), abnormal LFTs (5), leukopenia (4), and nephrotoxicity (3).

SUPPLEMENTARY TABLES

ID	Author (country)	Study design	OPAT Model(s)	OPAT group(s)†	Comparator(s)†	Findings	Notes
						drug reactions; three resulted in re-admission.<br>One patient died two days after completing treatment (ruptured iliac aneurysm).	Reasons for re-admission: PICC line infection (3), PICC line re-insertion (2), anxiety (1), nausea (1), and allergic reaction (1).
127	Walton et al (Australia)	Case-series	SN	35 patients (mean age 50); 71% male	—	Four patients stopped treatment prematurely.<br>No patients developed penicillin associated neutropenia.<br>Four patients had problems with PICC venous access; three required replacement.	Reasons for discontinuation included: probable drug related febrile hypersensitivity reaction (1), PICC-associated septicaemic shock (1), and weakness and focal seizures (1).<br>Reasons for PICC replacement were: catheter blockage (1), and exit site infection (2).
128	White et al (Australia)	Case-series (retrospective)	OP; SN; GN	55 patients (mean age 50); 65% male	—	Seven of 55 patients (13%) had adverse drug events, with IV antibiotics responsible for events in three patients.<br>There were no complications related to IV access.	Antibiotic events were: rash (1), thrombocytopenia (1), and deranged liver enzymes (1).
129	White et al (UK)	Case-series (retrospective)	SA	72 patients (mean age 42); 42% male	—	A total of 39 drug reactions were documented in 29/72 patients (40%). Therapy was discontinued in 10 patients (14%).<br>One patient was admitted to hospital (line infection with sepsis).	Drug reactions were: neutropenia (13), mild liver function derangement (8), line infection (3), rash (3), allergic reaction (2), diarrhoea (4), headache (1), nausea (1), fatigue (1), oral thrush (1), shingles (1) and dyspepsia (1).<br>Reasons for discontinuation were: neutropenia (4), drug rash (3), allergic reaction (1), and line infection with severe sepsis (1).
130	Williams (USA)	Observational (retrospective)	SA	1045 patients (mean age 38); 58% male	—	Drug related side-effects were relatively infrequent (2-5%).<br>Venous access issues were common (375/1500; 25%)<br>Rate of re-admission was 5-6% (75-90 patients).<br>No deaths were directly attributable to home therapy, and overall morbidity was low.	Drug side-effects were: nephrotoxicity (75), skin rash (75), and leukopenia (30).<br>Access issues included: infiltration of IV, and phlebitis of a peripheral cannula.<br>Reasons for re-admission included: antibiotic failure, therapy change, and surgery.

SUPPLEMENTARY TABLES

ID	Author (country)	Study design	OPAT Model(s)	OPAT group(s)†	Comparator(s)†	Findings	Notes
133	Wynn et al (USA)	Observational (retrospective)	Physician-based program; Home health organisation; Hospital-based program (no other details)	1252 patients (mean age 52); 65% male	—	A total of 66 adverse drug reactions were reported in 1,515 patients (4%).<br>There were 106 vascular access complications in 1,232 patients (9%).	Adverse drug reactions were: rash (26), diarrhoea (8), anaphylaxis (6), leukopenia (6), renal toxicity (6), fever (5), nausea/vomiting (5), and unspecified (4).<br>Access complications included: phlebitis (31), leakage (19), thrombosis (10), local reaction (9), and bacteraemia (4).
134	Yadlapalli et al (USA)	Case-series (retrospective)	Not specified	58 patients (mean age 60); 83% male	—	During 12-months follow-up, 10 patients (17.2%) required re-admission for further treatment.	
135	Yan et al (UK)	Case-series (retrospective)	OP; GN	140 patients (aged 17-89); 64% male	—	Five of 140 patients (3.6%) required hospital admission due to an inadequate response.<br>Three patients (2.1%) developed a rash.<br>There were no reported complications related to use of IV catheters (some patients inadvertently removed their cannula at home).	Three of the patients admitted to hospital required referral to the orthopaedic/surgical team due to abscess formation and tracking of cellulitis.

OP: outpatient; SA: self-administered; SN: specialist nurse; GN: General nurse.

† Age and gender details relate only to those patients who were clinically evaluable or provided questionnaire data.

For peer review only - http://bmjopen.bmj.com/site/about/guidelines.xhtml

TABLE S3 Cost-effectiveness of outpatient parenteral antibiotic treatment

ID	Author (country)	Study design	OPAT Model(s)	OPAT group(s)	Comparator(s)	Findings
60	Krauth et al (Worldwide)	Literature review	Not specified	11 studies	Inpatient therapy	Studies predominantly concluded that home IV therapy would lead to significant cost reductions, from the societal perspective as well as the third-party payer perspective. In five studies, inpatient therapy was 1.8 to 3.3 times as expensive as home care; three studies that considered incremental costs revealed potentially significant cost savings with home therapy. Two of the remaining three studies reported divergent results, primarily related to pharmaceutical pricing and duration of therapy.<br>Only one study included loss of working days and its monetary valuation, and found that the indirect costs per course of inpatient treatment totalled DM868 compared with DM476 for home treatment.
95	Patanwala et al (USA)	Decision tree analysis	SN	41 patients (mean age 53); 44% male	Switch therapy (IV-oral)<br>Oral therapy	Oral treatment during hospitalisation and after discharge had superior effectiveness and lower cost than the Switch and OPAT scenarios in the base-case, incremental cost-effectiveness analysis ($8923, 0.87 vs. $11479, 0.78 vs. $12481, 0.71, respectively). The cost variable that was expected to have the largest impact on the model was cost per day in hospital. Varying the baseline cost per day in hospital (range $463-$2500) did not affect the dominance of the Oral option.<br>The Switch option would be the cost-effective choice if the length of hospitalisation was <6 days or if the probability of cure with the Oral option was ≤0.72.
119	Teufel et al (Canada)	Decision tree analysis	SN	77 patients	Early discharge with oral therapy<br>Inpatient therapy<br>Oral outpatient therapy	Oral outpatient treatment yielded an average QAFNE of 0.65, inferior to OPAT (0.72) and Early discharge (0.66), but superior to hospital treatment (0.62). Oral outpatient treatment was cost saving, but less effective than outpatient IV (ICER of $10186 per QAFNE); Early discharge and Hospital treatment were dominated strategies.<br>Early discharge ($9265) and inpatient therapy ($21866) were less cost effective than either Oral or IV outpatient therapies (OPAT $5810; Oral $5338). At a WTP threshold of $4000 per QAFNE, Oral treatment was cost-effective in 54% of the simulations, and OPAT in 38%. Early discharge was cost effective in 8% and traditional hospital management in less than 1%. Beyond certain cost thresholds, dominance changed from the Oral to OPAT strategy, but there was no constellation when Early discharge or inpatient treatment became superior.

SUPPLEMENTARY TABLES

ID	Author (country)	Study design	OPAT Model(s)	OPAT group(s)	Comparator(s)	Findings
122	Thornton et al (UK)	Observational (retrospective)	Not specified	Home therapy; 47 patients (mean age 26); 36% male	Combined therapy; 18 patients (mean age 25); 61% male<br>Inpatient therapy; 51 patients (mean age 26); 59% male	When effective treatment was classed as ≤0% decline in FEV_1, mean ICER was £46,098 (-£374,044 to £362,472); when ≤2% was used, mean ICER was £73,885 (£1236 to £269,023). These are the amounts that must be spent to obtain one more year of effective treatment with hospital-based care for one patient. The cost-effectiveness planes indicated increased effectiveness and increased cost for Hospital treatment compared with Home treatment. There was a slight possibility that Hospital treatment may be less effective and more expensive than Home treatment at ≤0% decline; when ≤2% decline is used this was not the case.<br>The cost-effectiveness acceptability curve showed that if a decision maker was willing to pay up to £262,500 for one extra patient with an FEV1 decline of ≤2% over 1 year, there was a 95% probability that hospital care would be cost effective. If a decline of ≤0% was used, the probability that hospital care would be cost effective never reached 95% (even if the decision maker was willing to pay £10 million for one extra patient).
138	You et al (China)	Decision tree analysis	SA; SN	All patients (no numbers provided)	Oral therapy (early discharge)<br>Inpatient therapy	The base-case analysis showed that OPAT ($14,470 per patient) was the least costly alternative, followed by Oral ($17,877) and inpatient treatment ($19,980). Results were sensitive to variation of the success rates of the IV and oral drugs: 1) if the IV success rate was less than 55%, Oral treatment would become the least costly option; if the success rate was higher than 80%, both OPAT and hospital IV treatment would be less costly; 2) if the Oral treatment success rate was higher than 80%, this would be the least costly option; if the success rate was less than 55%, this would be the most costly. Throughout the ranges of all variables, OPAT remained less costly than hospital treatment.<br>OPAT was less costly than Oral and hospital treatment in 64% and 100% of the simulations, with mean savings of $2,313 (95% CI 2,188–2,438) and $4,881 (95% CI 4,869–4,893) per patient, respectively.

OP: outpatient; SA: self-administered; SN: specialist nurse; GN: General nurse; ICER: incremental cost-effectiveness ratio; QAFNE: quality adjusted febrile neutropenia episode

† Age and gender details relate only to those patients who were clinically evaluable or provided questionnaire data.

TABLE S4 Patient acceptability of outpatient parenteral antibiotic treatment

ID	Author (country)	Method	OPAT Model(s)	OPAT group(s)	Comparator(s)	Findings
**COMPARATOR STUDIES**						
30	Corwin et al (New Zealand)	Questionnaire survey	GN	Home therapy; 98 patients (mean age 55); 62% male	Inpatient therapy; 96 patients (mean age 48); 73% male	Most patients in both treatment groups were satisfied with the care received (Home 96%; Hospital 96%). Only 5% of Home patients would prefer hospital treatment, while 35% of Hospital patients would prefer home treatment (p<0.001). Nine percent of the Home group reported no preference for location compared with 34% of the Hospital group.
32	Dall et al (USA)	Telephone survey	SN	Home therapy; 92 pneumonia patients (97% aged >50 years); 44% male / 64 cellulitis patients (mean age 51); 70% male	Inpatient therapy; 10728 pneumonia patients (83% aged >50 years); 51% male / cellulitis patients (no numbers provided)	Overall satisfaction with the program was extremely high, with >90% of patients responding positively to the telephone survey. The ability to have medical care at home was consistently cited as a benefit.
44	Fernandez-Aviles et al (Spain)	Questionnaire survey	SN	Home therapy; 50 patients (mean age 47); 62% male	Inpatient therapy; 50 patients (median age 50); 54% male	All 30 of the patients and caregivers surveyed felt safe at home. Most patients (97%) indicated that they would choose to receive treatment at home again, and that they would recommend the procedure to other patients. The main advantages reported were quiet and increased home comfort (67%), familiar environment (27%), free choice of activity (27%), free choice of food (20%), and increased privacy (13%). Reported disadvantages (10 patients) including anxiety and fatigue (20%), cost of local hotel or apartment (7%), and caregiver anxiety (7%).
103	Richards et al (New Zealand)	Questionnaire survey	GN	Home therapy; 24 patients (mean age 50); 54% male	Inpatient therapy; 25 patients (mean age 50); 52% male	All Home group patients were *very happy* with their care, compared with 60% of Hospital patients (p=0.001). Similarly, most patients were happy with the location of their care, but the Home group were happier; 92% were *very happy* with the location, compared with 32% in the Hospital group (p<0.001).
**OPAT ONLY STUDIES**						
11	Al Ansari et al (Bahrain)	Questionnaire survey	OP	101 patients; 57% male	—	Survey response was high (96.9%). Patients were highly satisfied with the OPAT service (mean rating 4.41, SD 0.31).
16	Bamford et al (UK)	Interviews	Not specified	12 interviewees (mean age 62-63); 58% male	—	Most interviewees initially preferred oral administration. The most frequently cited disadvantages of IV administration were pain or discomfort and the inconvenience related to movement, followed by problems finding or maintaining the cannula and fear of needles.<br>Most thought the main advantage was that it would work quicker; other advantages mentioned were that it would be "*more effective*", "*less hassle*", "*it's correct and you don't forget*"

SUPPLEMENTARY TABLES

ID	Author (country)	Method	OPAT Model(s)	OPAT group(s)	Comparator(s)	Findings
						and "*it's probably gentler on the side effects on the stomach*". When specifically asked about IV antibiotics at home, four interviewees were clear that they would not be happy with the idea, three because of delivery issues, "*I just don't believe a district nurse could do it*", and one preferred oral therapy. Four others were happy with the idea, or had concerns about equipment/sterility but would be happy if management of the IV could be delivered at home, "*if there was no fear and it could be done sterilely*". When asked about management of future infections, 75% of those who gave an opinion preferred oral antibiotics. Of the two patients who preferred the IV route, one could not swallow tablets, one thought IV antibiotics were better.
22	Bernard et al (France)	Not specified	SA; SN;	39 patients (mean age 44); 64% male	—	None of the patients complained about having to return frequently for consultation with their attending physician, and they accepted this inconvenience in order to remain outside the hospital.
26	Chambers et al (New Zealand)	Questionnaire survey	SA	100 eligible patients (mean age 63)	—	Home IV therapy was rated as *good* or *very good* by 97% of respondents. Two patients (2%) reported that they would not have it again. When patients were asked to comment freely, 91% of the replies were extremely positive (the most common reason being a preference for home over hospital). Most patients rated the experience of having a PICC or midline inserted as very good (60%) or good (35%); 28% reported some problem with the IV catheter (leakage, blockage, discomfort, inconvenience, arm swelling, skin reaction to dressing, connector becoming undone). Most respondents with a pump device rated this as *good* or *very good* (88%); 8% reported discomfort or restricted movement, and 3% reported kinking. Twelve patients (14%) self-administered antibiotics and all would do so again. Of those who did not, 27% would have liked to have done so, the main reason given being freedom from appointments with nurses.
28	Chapman et al (UK)	Questionnaire survey	SA; Infusion centre	334 treatment courses (mean patient age 46); 59% male	—	Of 276 patients completing questionnaires since the OPAT service was established, 98.6% rated the service as *very good* or *excellent*; 99.6% reported that they would choose the OPAT service again.
31	Cox et al (USA)	Unscheduled calls or visits	SA	205 patients (mean age 59); 99% male	—	The younger group was significantly more likely than the older group to perform infusions unaided (41% vs 20%; P<0.001). Older patients were significantly more likely than younger patients to have an urgent care visit with a problem or question about the catheter or infusion (31.4 visits/1000 home IV days vs. 14.3 visits/1000 home IV days; p<0.001). Similarly, there

ID	Author (country)	Method	OPAT Model(s)	OPAT group(s)	Comparator(s)	Findings
						were more phone calls to the ID pharmacist from the older group (14.3/1000 home IV days vs. 8.57/1000 home IV days; p=0.04).
42	Esposito et al (Italy)	Not specified	OP; SA; Visit by doctor or nurse (also care facility)	239 patients (aged 11-80+); 62% male	—	Satisfaction with the OPAT regimen was high, with 91% of patients giving favourable feedback.
45	Goodwin et al (Canada)	Questionnaire survey	SN; GN	2405 patients (mean age 46); 55% male	—	Most of the 424 responding patients were *very satisfied* or *completely satisfied* with home therapy (61%); 6% were *not very satisfied*, and 5% were *totally dissatisfied*. Most of the satisfaction related to resuming daily activities such as work or school, and most dissatisfaction to costs incurred.
48	Grayson et al (Australia)	Not specified	GN	20 patients (mean age 58)	—	All patients reported a strong preference for home IV treatment over inpatient therapy, with many describing a sense of improved self-esteem, "ownership" of their illness, and involvement on therapy.
51	Hindes et al (USA)	Questionnaire survey; personal communication	SA	48 patients (mean age 65)	—	Surveys and personal communications demonstrated a high degree of satisfaction with the care provided, and strong endorsement of the home therapy provided as an alternative to inpatient care (no other details provided).
52	Hitchcock et al (UK)	Questionnaire survey	OP; SA; GN; Private home healthcare company	273 patients (mean age 60); 54% male	—	Most of the 84 survey respondents felt that the service met their expectations and would be happy to receive this form of treatment again should the situation arise (96%). All were happy with the support they received from the OPAT team during treatment; 95% felt that their quality of life during the period of infection had been improved by outpatient management (two of the patients who did not think this suffered an adverse drug event).<br>Most respondents (83%) thought that the service could not be improved. The most frequent comments from those who felt improvements could be made related to patient transport problems transport (6/14); two patients thought that communication between consultants, other staff and patients could be improved, three that they had waited too long, and one patient suggested referrals could be made directly by their GP.
55	Huminer et al (Israel)	Not specified	SA; SN (also nursing home; kibbutz)	37 patients (mean age 64); 57% male	—	Most patients (95%) reported a high level of satisfaction with this type of therapy (no other details provided).

SUPPLEMENTARY TABLES

ID	Author (country)	Method	OPAT Model(s)	OPAT group(s)	Comparator(s)	Findings
56	Johansson et al (Sweden)	Questionnaire survey; VAS	SA	11 patients (mean age 51); 73% male	—	All patients reported that OPAT was of great value (median VAS rating 97mm). Prior expectations of OPAT included peace and quiet, greater freedom and more time to do other things, the home environment being important for well-being, and to being an everyday part of the family. The 10 patients who responded after OPAT said that their expectations had been met.<br>All patients said they would prefer to have OPAT again during any subsequent IV antibiotic therapy, and all agreed there were many advantages to having treatment at home. Nine out of ten patients never felt worried during administration of the drug (although they sometimes worried about the direction of their underlying condition). Nine out of ten patients were fully satisfied with the education (inc. potential complications).<br>Specific advantages reported by patients included not having to go to hospital every day for therapy, more freedom, more time to do household duties and for leisure, the feeling of having control, freedom and flexibility, making it possible to be a part of daily issues at home, and being at home with your family. No examples of disadvantages were given.
57	Kayley et al (UK)	Not specified	SA; GN; Doctor administered	67 patients	—	Patients said they were happy to receive their treatment at home (anecdotal only – no formal survey)
59	Kieran et al (Ireland)	Telephone survey	SA; Nurses attached to commercial company	56 patients (median age 50); 57% male	—	All 12 patients surveyed reported that they were very happy to complete their course of antibiotics at home rather than remaining in hospital. Reasons given were preference of home to hospital (44%), the ability to resume activities of daily living (11%) or both (44%).<br>All patients were satisfied with the service received, none felt that they had inadequate training, and none felt that their infection took longer to be treated because they received OPAT. All stated that they would prefer to be treated at home if a similar situation arose in the future. Almost three quarters (70%) would be happy to be followed up by phone or internet with less frequent outpatient reviews, providing that care of the IV access device could be done in the community.
67	Lehoux (Canada)	Interviews	SA	6 interviewees (mean age 64); 50% male	—	User acceptance of home IV treatment was shaped by different forms of anxiety, such as protecting the catheter site to avoid potential infection or the possibility of the catheter becoming dislodged. The pump alarm system could go off too easily, and false alarms frequently disturbed sleep and were initially perceived as very stressful (over time they became a "normal" disturbance).

ID	Author (country)	Method	OPAT Model(s)	OPAT group(s)	Comparator(s)	Findings
						Professional and social life was slightly limited, and patients were generally passive or submissive about this, "*you're always a slave to it, having to carry it everywhere*". Carers sometimes curtailed social activities because they felt needed by the patient.<br>User-acceptance was closely linked to competence. Older patients did not feel comfortable with the electronic components of the pump, and the manual dexterity required to properly manipulate it could be problematic for older people, "*if my eyes were okay, I'd have been able to do it. But I was frightened of not doing it properly, of not seeing the needle, which is so tiny*" (IV carer). Direct observation showed patients who were unable to read messages on the digital screen due to poor eyesight, limited English, or illiteracy. They relied on memory or made informed guesses.
73	Marra et al (Canada)	Questionnaire survey	SA; SN	91 patients (mean age 56); 69% male	—	The majority of the 91 enrolled patients (96%) indicated a treatment location preference; 89% preferred home, while the remainder preferred hospital. Most patients (82%) gave an interpretable response regarding willingness to pay (WTP) for treatment in their preferred location. Of these patients, 90% preferred home with a median WTP of $490 CDN, and the remainder preferred hospital with a median WTP of $500 CDN (not statistically significant). Total WTP for the patients who preferred hospital treatment was $7,859 versus $60,712 for the patients who preferred home treatment. Those who stated preferences were willing to pay more than those who did not (p<0.0001).
74	Martel (France)	Questionnaire survey	SA	116 patients (mean age 36 (M), 41 (F)); 76% male	—	Patients who received Home treatment (n=33) had a significantly higher internal locus of control, while patients who preferred to continue their treatment in hospital (n=17) had a higher external locus of control. The main reasons for accepting Home treatment were socio-familial, wanting to carry on normal activities and to have higher autonomy or freedom. The hospital group had low confidence in their own ability.<br>The majority of Home group patients (89%) would use home treatment again if the cost was paid by a third party; 32% agreed to participate again even if they were responsible for the cost of antibiotics.
82	Montalto (Australia)	Telephone interviews	SN (HHU)	67 patients (aged 16-70+); 49% male / 65 carers (aged 16-70+); 29% male	—	Preference for the convenience and comfort of home was the most commonly cited reason for agreeing to enter the HHU (67%). More than a quarter of patients (28%) mentioned avoidance of hospitalisation, and a smaller number (13%) felt that it saved a bed for another person; three patients believed that they were not ill enough to warrant a bed. Seven patients (10%) felt that their choice was constrained as no inpatient beds

SUPPLEMENTARY TABLES

ID	Author (country)	Method	OPAT Model(s)	OPAT group(s)	Comparator(s)	Findings
						were available at the time. Almost all (97%) would use the service again if the opportunity arose. Benefits of a home environment were the most commonly perceived advantages of HHU care (62%), with patients reporting feeling happier, more comfortable at home, greater personal freedom, companionship, and less disruption for themselves and their family. Positive characteristics of the HHU were mentioned by 60% of patients; only two mentioned disadvantages of HHU care. Seven patients recalled feeling worried during their stay, but when asked whether they felt confident during their time in the HHU, all 67 patients, including those who felt worried, responded positively. The most common reason given for this was the 24-hour contact numbers for emergency backup.
89	Nathwani et al (UK)	Questionnaire survey	OP; SA	101 patients	—	Most patients (79%) were happy with all aspects of the care received; 96% reported that the service improved their quality of life, and 89% that this form of treatment met or exceeded their expectations. For 93% of patients it was preferable to inpatient care, and 93% would prefer this model in the future if the need arose. Most patients' family/carers were satisfied with the service (93%).
90	Nathwani (UK)	Not specified	OP; SA; SN	125 patients	—	Patient satisfaction with OPAT was very high (no other details provided.)
93	Parker et al (UK)	Questionnaire survey; focus group; call to practices	OP; SA	29 patients; 38 GP practices	—	Most patients (79%) reported the freedom of being at home as the main reason for taking part. Other reasons included less family disruption (31%), and increased social contact (21%). Of 26 patients who completed the end of study questionnaire, 92% were very much in favour of non-inpatient treatment and would repeat this form of therapy. Questionnaire results were supported by the focus group; all patients would repeat this form of therapy and felt that treatment outside of hospital improved their quality of life.
97	Pilling & Walley (UK)	Interviews	SA; Commercial home care organisation	11 CF patients (aged 17-30) / 14 parents (children aged 6-16)	—	Most of the respondents (92%) had considerable experience of home therapy, having received home IV antibiotics for ≥1 year. All preferred to be treatment at home rather than in hospital provided they were reasonably well. Most (96%) were satisfied with the quality and comprehensiveness of training, although one respondent felt that more information should have been provided on potential adverse reactions, and that the quality of homecare could be improved by the provision of refresher courses on the management of infusion devices. Support arrangements were thought to be good, and all knew how to

SUPPLEMENTARY TABLES

ID	Author (country)	Method	OPAT Model(s)	OPAT group(s)	Comparator(s)	Findings
						obtain advice if needed. Fifteen respondents (60%) had experienced one or more problems with their therapy (drug effervescence, painful administration, line blockage, failure of ambulatory infusion device, storage of drugs or equipment, getting rid of used equipment etc.). Most were minor, but some such as line blockages required a visit to hospital or a home visit from the nurse. Patients who self-constituted their antibiotics reported difficulties in obtaining adequate supplies of ancillary items (arbitrary quantities were provided and further supplies were difficult to obtain). They also reported problems with storage of drugs and equipment, and disposal of used equipment, particularly needles. With this exception however, all respondents were satisfied with the support they received from hospital and had no suggestions for improvement.
104	Rodriguez-Cerrillo et al (Spain)	Questionnaire survey	SN (HHU)	24 patients (mean age 73)	—	95% of patients treated expressed their satisfaction with this type of treatment.
105	Rodriguez-Cerrillo et al (Spain)	Questionnaire survey	SN (HHU)	25 patients (mean age 59); 48% male	—	All patients expressed their satisfaction with treatment at home (no other details provided).
108	Seaton et al (UK)	Questionnaire survey	Not specified	205 eligible patients (mean age 49); 51% male†	—	Most of the 183 responding patients (84%) thought that OPAT would be an acceptable alternative to inpatient therapy. Only three of the 29 patients who disagreed gave explanations (severe arthritis, fear of the IV device and disagreement in principle). This group was significantly older than those who were willing to have IV therapy at home ($\bar{x}$ 64y vs. 46y; p<0.001). Acceptability was not influenced by gender. All groups of patients (by condition) were equally agreeable to the prospect of outpatient therapy. Of the 95 patients who received IV antibiotic therapy, 87% thought that OPAT was an acceptable alternative to inpatient therapy, and 72% had a caregiver who would be willing to administer the antibiotic at home.
113	Sims et al (UK)	Telephone survey	OP	14 patients (mean age 63); 71% male	—	One patient died of unrelated causes prior to questionnaire completion. All of the 13 patients contacted were satisfied with the OPAT service, thought that it was more convenient than an inpatient stay, and should the same problem recur would undergo OPAT again. All reported they would recommend the service to a friend under similar circumstances.

SUPPLEMENTARY TABLES

ID	Author (country)	Method	OPAT Model(s)	OPAT group(s)	Comparator(s)	Findings
118	Talcott et al (USA)	Likert scales	SA; SN	30 patients (median age 38); 43% male	—	Before home therapy, all 30 patients overwhelmingly preferred home to hospital therapy, and had little fear of isolation from their physician if a serious problem occurred. These convictions were unchanged after completion of home therapy.<br>The report of family comfort with home care increased after treatment: support for the statement, "My family would rather have me near them than be in the hospital when something happens like this (developing fever and neutropenia)" increased from 42% agreement to 70% agreement.<br>There was some evidence that patients felt slightly more isolated at home than in the hospital: agreement with the statement "I feel uneasy about calling my doctor from home with a complaint that worries me a little, for fear that I will be disturbing him or her with such a minor problem" increased from 10% to 24%.
120	Teufel et al (Canada)	Interviews; VAS	Not specified	78 participants (mean age 54); 41% male	—	Most respondents (75%) preferred some form of outpatient management. Home oral treatment was most commonly ranked first (36%), but 21% of respondents preferred Home IV ad 18% preferred Early discharge. All three outpatient strategies were associated with higher mean VAS scores than (5.3 vs. 5.7 for Early discharge, 6.1 for Home oral, and 6.2 for Home IV).<br>There was no significant difference between the outpatient strategies in relation to how much remaining life patients would be willing to give up to avoid inpatient care; $\bar{x}$ 9.1 weeks for Early discharge, 9.6 weeks for Home IV, and 9.3 weeks for Home oral. On average, patients would give up <1% of their remaining lifetime to avoid inpatient care.<br>Findings were similar for willingness to pay ($\bar{x}$ \$282 for Early discharge, \$327 for Home IV, and £255 for Home oral). The maximum WTP to receive IV treatment at home instead of in hospital was \$4,500 (max. \$2,000 for Early discharge and \$1,500 for Home oral).
123	Tice (USA)	Questionnaire survey	OP; SA; SN	538 patients (mean age 45); 52% male	—	Most of the 294 responders (89%) reported that they *definitely would* have outpatient therapy instead of being hospitalised if the need arose again, and 10% said they *probably would* have outpatient therapy again. Of the four remaining patients, two said they *probably would not*, and two said they *definitely would not*.
124	Tice (USA)	Questionnaire survey	OP; SA; SN; Infusion centre.	500 patients	—	Almost all of the respondents (99%) reported that, should they require parenteral antibiotic therapy again, they would *definitely* or *probably* choose to be treated at home; <1% said they would prefer hospitalisation.

For peer review only - http://bmjopen.bmj.com/site/about/guidelines.xhtml

SUPPLEMENTARY TABLES

ID	Author (country)	Method	OPAT Model(s)	OPAT group(s)	Comparator(s)	Findings
135	Yan et al (UK)	Questionnaire survey	OP; GN	140 patients (aged 17-89); 64% male	—	The survey was completed following an OPAT pilot scheme; 88% of patients (no numbers given) were pleased with the service as it enabled earlier discharge, even if this meant daily trips back to hospital.

OP: outpatient; SA: self-administered; SN: specialist nurse; GN: General nurse.

† Age and gender details relate only to those patients who were clinically evaluable or provided questionnaire data.

For peer review only

TABLE S5 Provider acceptability of outpatient parenteral antibiotic treatment

Ref.	Author (location)	Method	OPAT Model(s)	OPAT group(s)	Comparator(s)	Findings
63	Lane et al (USA)	Questionnaire survey	OP; SA	555 adult infectious diseases physicians	—	Lack of a dedicated OPAT team was reported as the most common barrier to providing safe OPAT services (median rank 2), followed by the large number of locations at which patients received OPAT, communication issues, and volume of lab results (median rank 3).
68	Lehoux et al (Canada)	Questionnaire survey	SA	51 nurses	—	Respondents strongly agreed that home IV therapy gave patients greater autonomy ($\bar{x}$ score 5.2; SD 1.0), and made it easier to pursue a normal life ($\bar{x}$ 4.4; SD 1.0). They viewed the technology as simple to use ($\bar{x}$ 4.5; SD 1.1), but believed it triggered some patient anxiety ($\bar{x}$ 3.3; SD 1.3).<br>Nursing teams perceived that IV therapy provided some challenges for providers in relation to the technical complexity of devices ($\bar{x}$ 3.5; SD 1.3), number of devices used by their hospital ($\bar{x}$ 3.3; SD 1.4), pace of product renewal ($\bar{x}$ 3.2; SD 1.4) and fit with the home setting ($\bar{x}$ 3.1; SD 1.4).<br>Dealing with patients' psychological ($\bar{x}$ 3.0; SD 1.0) and cognitive limitations ($\bar{x}$ 3.0; SD 1.1) was viewed as slightly more demanding than dealing with physical limitations ($\bar{x}$ 2.9; SD 1.1). There was a perceived increase in effort related to teaching patients how use the technology ($\bar{x}$ 3.1; SD 1.1), about associated risks (mean 3.0; SD 1.1), how to manage their anxiety ($\bar{x}$ 3.0; SD 1.1) and how to manage impact on their life ($\bar{x}$ 2.8; SD 1.0).<br>The two features reported as being most important for improvement, were ease of use for patients ($\bar{x}$ 3.9; SD 1.2) and integrating the technology into the patient's life ($\bar{x}$ 4.0; SD 1.3).
86	Muldoon et al (Ireland)	Questionnaire survey	Not specified	55 consultant physicians (15% clinical microbiologists)	—	Forty-one respondents (74%) had discharged patients with IV antibiotics, but almost half (n=26, 47%) did not have a designated OPAT service available to them; all felt that there was a need for a local OPAT service. Of respondents answering the question, 63% (12/19) reported that they had experienced difficulty obtaining funding for OPAT.<br>Forty-five percent (13/18) reported that they thought >75% of their patients were satisfied with OPAT services; only one consultant (6%) felt that <50% of patients were satisfied.
87	Muldoon et al (USA)	Questionnaire survey	Not specified	316 adult and paediatric infectious diseases physicians	—	Thirty-four respondents rarely or never participated in OPAT: 48% cited logistic reasons or lack of an OPAT program, 15% that another practitioner had responsibility for OPAT, 15% reported lack of control of patient management, 12% lack of reimbursement, and 10% low patient volume.<br>In the 'Comments' section, many respondents reported

Ref.	Author (location)	Method	OPAT Model(s)	OPAT group(s)	Comparator(s)	Findings
						frustration about lack of institutional and financial support for OPAT, while also expressing a significant liability burden.
93	Parker et al (UK)	Questionnaire survey; focus group; call to practices	OP; SA	29 patients; 38 GP practices	—	Most GP practices (76%) saw no advantage for themselves in home IV treatment. Those that did mentioned admissions saved and freed up hospital beds, that the GP maintained responsibility for the patient, and that the patient could be treated at home. Most practices (70%) saw a substantial disadvantage in increased workload, and a small number were concerned about the safety of IV access (18%). However, almost all (94%) thought patients would benefit from being in their own environment, although many saw distance from hospital and lack of support and nursing care as a disadvantage (68%).
107	Seaton & Nathwani (UK)	Questionnaire survey	Not specified	157 infection specialists (92% clinical microbiologists)	—	Most of the 157 respondents had experience of OPAT, but only 21% had an established program within their institution. Of those with no program, 61% thought there was a definite need, while 14% thought there was no need for a formal program. Only 2% thought IV therapy should always be given in hospital.<br>Of the 124 specialist without a program, perceived barriers to development of OPAT were mainly organisational, including source of funding (n=43), lack of leadership (n=42), links between hospital and community (n=37), and identification and training of staff (n=21). Small numbers of patients or fragmentation of patient distribution within Trusts was also a concern (n=34). Concerns over lack of experience (n=17) or patient safety and acceptability (particularly line care and antibiotic administration), were expressed by a few respondents (n=12).<br>There was no consensus regarding funding and clinical responsibility for patients; 40% thought secondary care should pay and specialists take day-to-day clinical responsibility, while 50% thought that care should be shared between hospital and community practitioners.

OP: outpatient; SA: self-administered; SN: specialist nurse; GN: General nurse.

† Age and gender details relate only to those patients who were clinically evaluable or provided questionnaire data.

CIVAS Systematic Review: Mitchell et al – PRISMA Checklist

Section / topic	Item no.	Checklist item	Reported on page no
**Title**			
Title	1	Identify the report as a systematic review, meta-analysis, or both	1
**Abstract**			
Structured summary	2	Provide a structured summary including, as applicable, background, objectives, data sources, study eligibility criteria, participants, interventions, study appraisal and synthesis methods, results, limitations, conclusions and implications of key findings, systematic review registration number	3
**Introduction**			
Rationale	3	Describe the rationale for the review in the context of what is already known	4
Objectives	4	Provide an explicit statement of questions being addressed with reference to participants, interventions, comparisons, outcomes, and study design (PICOS)	4 & 5
**Methods**			
Protocol and registration	5	Indicate if a review protocol exists, if and where it can be accessed (such as web address), and, if available, provide registration information including registration number	3
Eligibility criteria	6	Specify study characteristics (such as PICOS, length of follow-up) and report characteristics (such as years considered, language, publication status) used as criteria for eligibility, giving rationale	5
Information sources	7	Describe all information sources (such as databases with dates of coverage, contact with study authors to identify additional studies) in the search and date last searched	4-5
Search	8	Present full electronic search strategy for at least one database, including any limits used, such that it could be repeated	Appendix 1
Study selection	9	State the process for selecting studies (that is, screening, eligibility, included in systematic review, and, if applicable, included in the meta-analysis)	5
Data collection process	10	Describe method of data extraction from reports (such as piloted forms, independently, in duplicate) and any processes for obtaining and confirming data from investigators	6
Data items	11	List and define all variables for which data were sought (such as PICOS, funding sources) and any assumptions and simplifications made	6
Risk of bias in individual studies	12	Describe methods used for assessing risk of bias of individual studies (including specification of whether this was done at the study or outcome level), and how this information is to be used in any data synthesis	6-7; Appendix 2
Summary measures	13	State the principal summary measures (such as risk ratio, difference in means).	N/A
Synthesis of results	14	Describe the methods of handling data and combining results of studies, if done, including measures of consistency (such as I^2 statistic) for each meta-analysis	7
Risk of bias across studies	15	Specify any assessment of risk of bias that may affect the cumulative evidence (such as publication bias, selective reporting within studies)	8-10
Additional analyses	16	Describe methods of additional analyses (such as sensitivity or subgroup analyses, meta-regression), if done, indicating which were pre-specified	N/A
**Results**			
Study selection	17	Give numbers of studies screened, assessed for eligibility, and included in the review, with reasons for exclusions at each stage, ideally with a flow diagram	7; 27 (Figure 1); 28 (Table 1)

For peer review only

Study characteristics	18	For each study, present characteristics for which data were extracted (such as study size, PICOS, follow-up period) and provide the citations	7-8; 29-35 (Table 2); Tables S1-S5
Risk of bias within studies	19	Present data on risk of bias of each study and, if available, any outcome-level assessment (see item 12).	9-10
Results of individual studies	20	For all outcomes considered (benefits or harms), present for each study (a) simple summary data for each intervention group and (b) effect estimates and confidence intervals, ideally with a forest plot	36; (Table 3)
Synthesis of results	21	Present results of each meta-analysis done, including confidence intervals and measures of consistency	N/A
Risk of bias across studies	22	Present results of any assessment of risk of bias across studies (see item 15)	N/A
Additional analysis	23	Give results of additional analyses, if done (such as sensitivity or subgroup analyses, meta-regression) (see item 16)	N/A
**Discussion**			
Summary of evidence	24	Summarise the main findings including the strength of evidence for each main outcome; consider their relevance to key groups (such as health care providers, users, and policy makers)	14
Limitations	25	Discuss limitations at study and outcome level (such as risk of bias), and at review level (such as incomplete retrieval of identified research, reporting bias)	14-15
Conclusions	26	Provide a general interpretation of the results in the context of other evidence, and implications for future research	15-16
**Funding**			
Funding	27	Describe sources of funding for the systematic review and other support (such as supply of data) and role of funders for the systematic review	17

For peer review only
